# Supplementary material for: CyVerse: Cyberinfrastructure for open science
Source: PLoS Comput Biol. 2024 Feb 7;20(2):e1011270. doi: 10.1371/journal.pcbi.1011270 (PMC10878509; doi:10.1371/journal.pcbi.1011270)
Supplement: S1 Text — Additional details about CyVerse featured platforms, core services, and cloud native services. Basemaps from Carto and OpenStreetMap CC-BY 4.0 license, (https://github.com/CartoDB/basemap-styles). (PDF) [file pcbi.1011270.s001.pdf]

# S1 Text

## Powered By

CyVerse services are used in over 40 external projects (S4 Table). "Powered By" projects rely upon components of the CyVerse SaaS, i.e., authentication, data storage, cloud services, etc.

Featured services under the **Powered By** framework, which can be accessed directly through the User Portal, include:

**BisQue (Bio-Image Semantic Query User Environment)** is a sophisticated web based image analysis platform to store, visualize, organize, and analyze data [91–94]. BisQue supports 2D, 3D, imagery, video, and tomography data from the visible, hyperspectral radiometric, magnetic resonance imaging (MRI), and X-Ray data from virtually all domains. BisQue is an imaging service that can capture, annotate, query, analyze, and train machine learning (ML) models [91]. BisQue is integrated with the DataStore, OAuth, and Cloud-Native Services for seamless scalability. A licensed version of BisQue software, called ViQi.org, continues to be developed with new features, including ML, in partnership with CyVerse. In its hosted BisQue service, CyVerse currently supports 5.57 million images, with 35 thousand new images added per month.

**CoGe (Comparative Genomics)** is designed to help make access to genomic information easy and to facilitate doing complex comparative genomics analyses [95–98]. Its suite of over 30 web-based analytical tools allow users to perform single gene, multiple gene, whole genome, and multiple genome analyses and share them, publicly with a unique URL, or privately with other CoGe users. CoGe leverages CyVerse's authentication, data store, and compute resources to power its services. CoGe currently supports over 6,700 registered users and more than 62,000 genomes from 21,000 organisms.

**DNA Subway** takes the metaphor of riding a subway line and applies it as a bioinformatics workspace that makes high-level genome analysis broadly available to students and educators [99]. DNA Subway is a complement to the DE, with a simplified interface that allows instructors to teach students about genomic analysis in a logical flow (as subway lines). DNA Subway leverages CyVerse's authentication, data store, and compute resources to power its services.

**SciApps** is a cloud-based platform for bioinformatics workflows which is run over CyVerse and the TACC Tapis API system [100]. Example projects which support interactive web-based workflows include MAIZECode [101] and BSAseq [102]. SciApps leverages CyVerse's authentication, data store, and HPC compute resources to power its services.

**PlantIT** (https://plantit.cyverse.org/) is one example of an open source "Powered By" project. PlantIT acts as a domain specific portal for the plant phenotyping community and leverages multiple components of CyVerse SaaS (S5 Table). PlantIT uses CyVerse authentication, data storage, and workflows enabled by Dockerized workflows and HPC scheduler under the hood. Projects such as PlantIT can be thought of as templates to gather communities around their computational tools by only changing the appearance of the system. That means if CyVerse is an apartment complex in the city of cyberinfrastructure, then PlantIT is the layout for an apartment that can be customized.

## Physical Resources

CyVerse operates approximately one hundred and sixty servers on premises at The University of Arizona (UArizona) location (S7 Table).

These servers manage its core infrastructure: iRODS Data Store, DE, and Cloud-Native Services (Main Text Fig 2). Servers at UArizona are connected across Internet2 [45, 103] to the NSF ACCESS-CI and to HTC via the Open Science Grid (OSG) and PaTH (Fig A). Strategically, CyVerse has worked to minimize the physical footprint of the hardware resources it operates. This reduces our overall operating cost and maximizes financial investments into software development, science outreach, and training. Through partnerships with other NSF cyberinfrastructure investments [104–107], CyVerse leverages public research hardware resources, maximizing the utilization of its own hardware while also disseminating its software to other hardware projects, e.g. Jetstream and Jetstream2 at Indiana University [74, 108, 109].

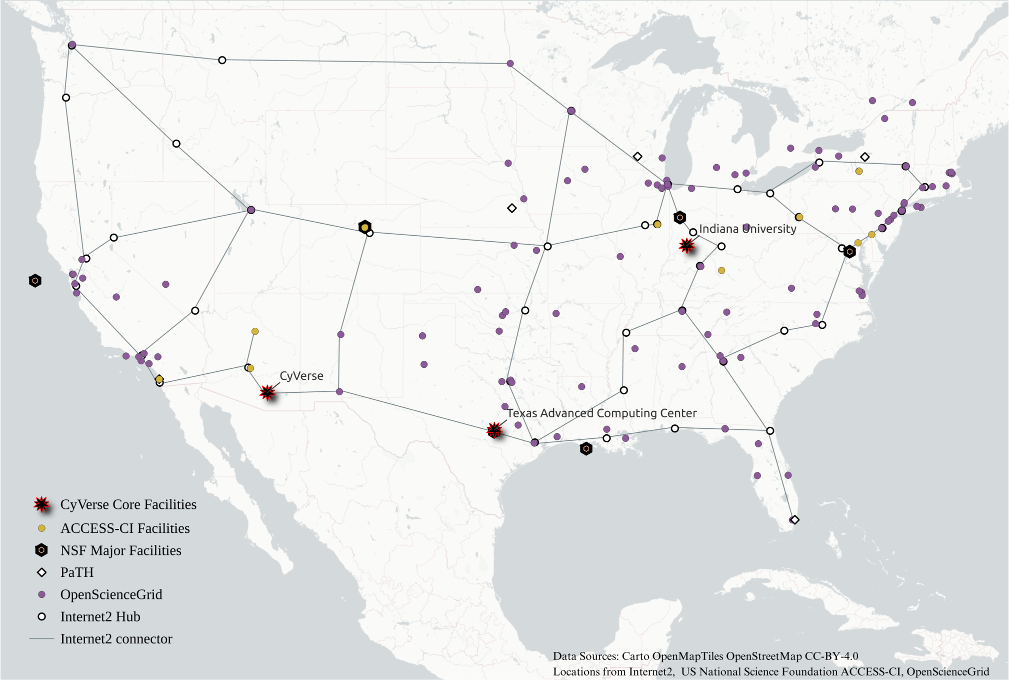


**Fig A. Where CyVerse US operates.** CyVerse US connects with the ACCESS-CI (triangles) to NSF Major Facilities (hexagons), and the OpenScienceGrid (diamonds) over a combination of Internet2 (open circles) and regional high speed networks. High Throughput Computing and High Performance Computing jobs run on CyVerse are distributed across the country based on resource availability and user's credentials when creating custom applications. All locations from public address records, base-map from Carto and OpenStreetMap CC-BY 4.0 license (https://github.com/CartoDB/basemap-styles).

## OpenStack Clouds

CyVerse operates OpenStack clouds [110–112] at UArizona for production services, development, and testing. The development clouds support larger-scale production clouds Jetstream2 at TACC, Indiana University, Arizona State University, Cornell, and University of Hawaii. The original Jetstream's web service and API are based on

CyVerse's foundational service Atmosphere. A hybrid cloud service is operated using HTCondor [46] and Kubernetes [43,113,114] for the DE's executable and interactive apps.

### High Performance Computing

CyVerse has partnered with multiple organizations within the NSF-supported XSEDE [104] (now) ACCESS-CI to connect users with HPC resources (Fig A). The DE framework allows researchers to seamlessly launch jobs on HPC. In practice, jobs are launched at TACC, the San Diego Supercomputer Center (SDSC), National Center for Supercomputing Applications (NCSA), and UArizona HPC. At TACC, CyVerse leverages the Tapis v3 [107,115] web-based API framework for securely managing computational workloads across infrastructure and institutions.

### High Throughput Computing

High Throughput Computing (HTC) describes multiple simultaneous processes [jobs] which run in parallel or sequentially across many computational processors [cores]. Examples of HTC workflows include genome assemblies and processing and reconstruction of signals and images. HTCondor integration with the OSG [46] in the DE allows researchers to launch jobs with tens to hundreds of simultaneous processes [jobs] across the entire OSG framework (Fig A). CyVerse hosts nodes as part of the OSG pool. CyVerse DE helps to reduce the complexity of using HTCondor by providing GUI-based tools and templates for users to design their own workflows which run on HTC. CyVerse resources can also connect to the new PaTH project.

### Data Storage

Data are stored across multiple resource servers at UArizona and at TACC which coordinate the management of over 8 PB of user contributed data in an Integrated Rule Oriented Data System (iRODS) [116] "Data Store". Data are replicated (mirrored) nightly between UArizona and TACC's Corral [117], a petascale storage and data management resource. OpenStack cloud services nodes and DE allocated processing nodes hold temporary (scratch) data while they are in use. During an analysis in the DE or on OpenStack, data can be moved anywhere across the internet or copied back to the Data Store when the analyses are completed.

### Databases

CyVerse operates multiple databases within its infrastructure. These include a list of all registered users and their host institutions, the Data Store iRODS database for user and community file storage, metadata required by the DE's applications and tools, as well as a PostgreSQL database [118,119] which makes file and folder metadata and contents queryable in the DE. CyVerse uses ElasticSearch [120] for indexing and searching of data in the Data Store.

## Foundational Services

CyVerse Foundational Services (Main Text Fig 2) provide the linkage between end-user platforms and hardware resources. These services are often referred to as 'middleware' and serve as the glue holding the rest of cyberinfrastructure together. CyVerse provides a federated authentication service for its users, a user portal where platforms and

services can be requested, an API for launching resources in the DE from 3rd party platforms, cloud services for managing virtual machines and clusters, and researcher support services called "Powered by CyVerse" which leverage one or more of these resources.

## Authentication and Security

CyVerse services use Central Authentication Service (CAS) [121] and KeyCloak [122] which operate on the OAuth2.0 standard internet protocol [123] (Fig B). After creating an account, users authenticate as a single sign-on service in their internet browser (Fig C). Users are encouraged to provide their academic, government, or organizational email address and ORCID (Open Researcher and Contributor IDentifier) [124] when creating profiles. User information is private, in accordance with European Union General Data Protection Regulations (EU-GDPR) [125]. Users can authenticate through KeyCloak using a CILogon [126], GitHub, Globus [127], or Google account. Once authenticated to CyVerse, only the authenticated user can access the secure Uniform Resource Locators (URL) (commonly known as 'web addresses') for the featured Platforms. Private URLs to running applications can be shared with other users through the DE interface after they have been started.

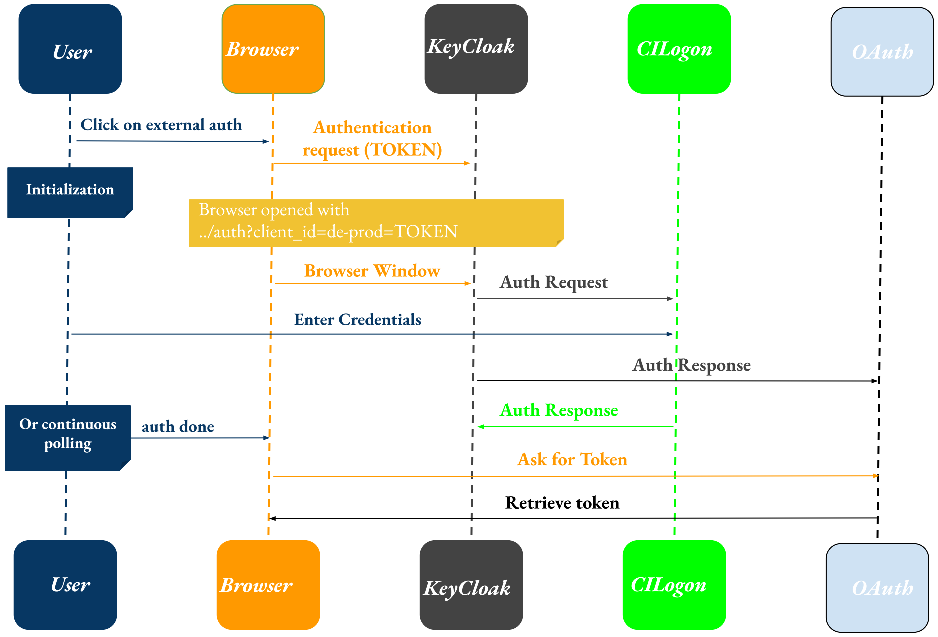


**Fig B. Authentication.** Users (navy blue) log in through a Web Browser (orange) where they submit their credentials through either KeyCloak (gray) and CILogon (green). These authentication requests are accepted by OAuth2.0 (black) and returned. UML template adapted from GitHub user JMBarbier (https://github.com/jmbarbier).

## User Portal

Through the User Portal (Fig D), new users can create and manage their account, request access to featured platforms, and request to schedule workshops. The User

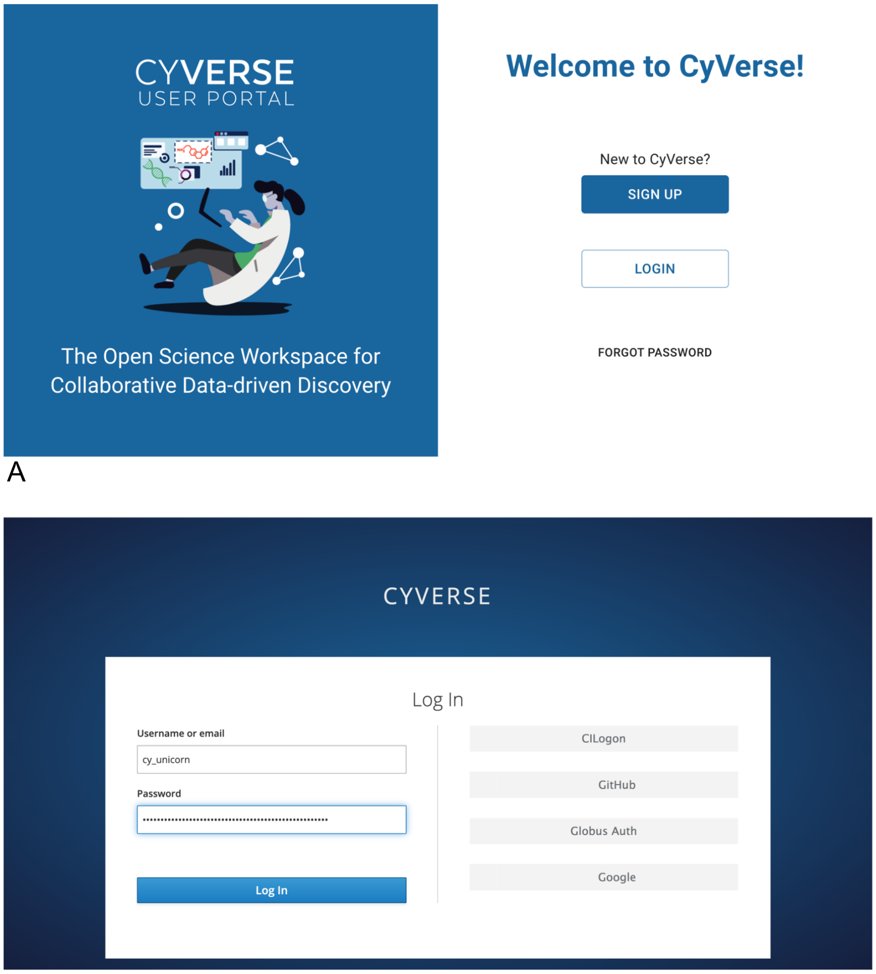

B

**Fig C. User login screen.** CyVerse authentication uses Keycloak with CILogon, GitHub, Globus Auth, or Google credentials. Users can log in with their unique CyVerse username or from their preferred single-sign on service.

Portal interface also provides hyperlinks to all the CyVerse platform's featured Services.

Requests for access to featured services, platforms, workshops, and community data released folders are reviewed by CyVerse staff. Requests for federated CyVerse services, 'Powered by CyVerse' as a 3rd party platform projects, or replicated CyVerse deployments, are sent through the ticketing system (Intercom.io). Requestors are contacted directly by CyVerse leadership to begin discussions and contracting agreements.

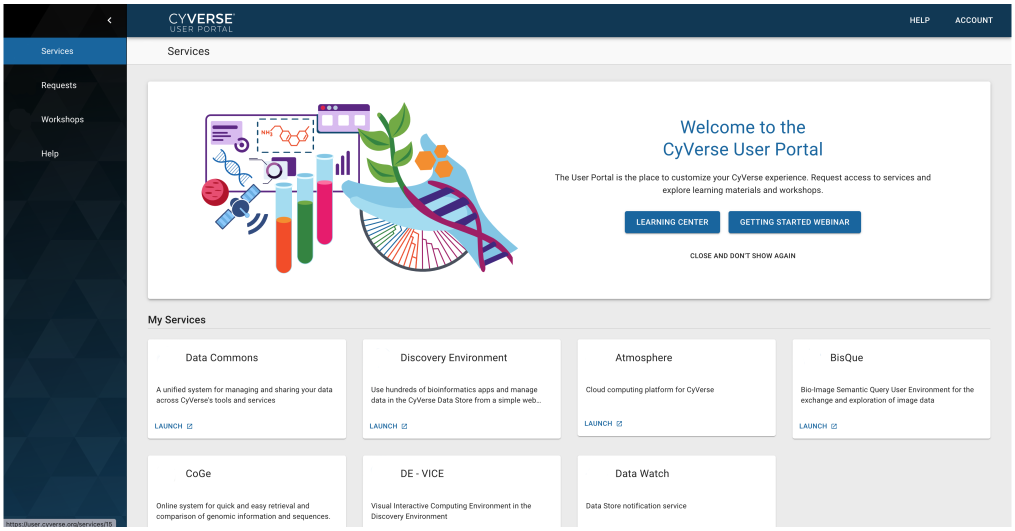


**Fig D. User Portal.** Provides access to all other CyVerse platforms and services, as well as requests for additional data storage, cloud resources, and workshops.

## Terrain API

The DE uses JSON [128] for managing its REST API service called "Terrain." Terrain API serves as the backbone to the DE and can be used outside of CyVerse's featured platforms over a Swagger RESTful API [128, 129]. This allows users to build or define tools and workflows to their own services. CyVerse software engineers connect 3rd party projects to the Terrain API as part of the Powered by CyVerse feature. At TACC, the Tapis web service APIs support HPC jobs requested by DE users by marshaling data to and from the CyVerse Data Store systems, while also resiliently managing job submission and lifecycle on XSEDE and other HPC providers.

## Cloud-Native Services

CyVerse has developed and maintained an OpenStack cloud service [111, 112] called 'Atmosphere' for the last 10 years [130]. The Atmosphere services were expanded and made available via 'Jetstream' (NSF award OAC 1445604) in 2016 [74] and again in 2021 with Jetstream2 (NSF award OAC 2005506 ). Atmosphere abstracts numerous complex operations required to manage and launch virtual machines in an OpenStack cloud, thus providing an easy to use interface for researchers through the browser. Featured base images of common Linux operating systems and Graphic User Interface (GUI) desktops provide users a workspace on which they can rapidly compile scientific software, run services, and analyze data.

As containers have become the dominant modality for software virtualization, the need for full deployment of virtual machines with base OS images and administrator access has changed. Users now bring their own precompiled containers of preferred

operating systems and scientific software stacks into the cloud. In the second generation of CyVerse, the Cloud-Native Services team has incorporated Kubernetes (K8s) [43], Lightweight Kubernetes (Rancher K3s) [131], and Argo workflows [42] for orchestrating containers in its platforms. Orchestration and job scheduling allows CyVerse to simultaneously manage hundreds of users running interactive environments (i.e., while thousands of jobs are run across multiple computational platforms (HTCondor, HTC, HPC).

# Platform Products

The CyVerse Data Store is part of the foundational services offered by the cyberinfrastructure. The DE enables users to run analyses and access the Data Store. CyVerse's focus on cloud has evolved from managing OpenStack instances via its Atmosphere client toward a continuous analysis platform which functions as Cloud-Native Services.

## Data Store

The Data Store utilizes iRODS [116] running across a distributed array of storage nodes located at UArizona. It is replicated weekly to the Corral storage resource at the TACC. As of mid-2023, the CyVerse iRODS store is holding 8 petabytes (PB) of user contributed data, which total 200 million individual objects (Fig E). Currently, CyVerse iRODS handles on average 100 terabytes (TB) of uploads and 400 TB of downloads per month (Fig F). Transfer (download and upload) speeds between CyVerse and TACC and between CyVerse and cloud services currently range between 140-220 MB/s for large files. See S6 Table for expected transfer duration by file size in seconds. Internal transfers between iRODS storage and computing nodes vary between 25-300 MB/s, depending on the storage type of the compute nodes (i.e., solid state drives [SSD] vs spinning disk hard drives). User data can be managed through CyVerse browser-based platforms including the DE and BisQue, or through terminal-based software such as iCommands and iRODS FUSE Lite. Data can also be uploaded or downloaded using third party software such as FileZilla, CyberDuck, and standard file browsers like Windows File Explorer.

The iRODS Data Store uses a conventional three-tiered Linux permission system (i.e., 'read', 'write', and 'own') for files and folders. Data is shared internally with iRODS by adding permissions to individual objects or collections with other CyVerse users' private names, or with 'teams' made up of grouped usernames administered by a project owner. Data can also be shared with the entire CyVerse user base by adding the 'public' username group, or the open internet by adding the 'anonymous' username group. The CyVerse metadata database is the primary repository for DE metadata storage. Based on AVU-triples (Attributes-Values-Units) foundation of iRODS metadata database, the CyVerse metadata database allows an unlimited number of AVU combinations. For example, users can use the same attribute with more than one value or more than one type of unit, significantly expanding the degree of metadata that can be stored for data analysis and retrieval. AVUs are exposed to web crawlers, like schema.org when they are shared with the 'anonymous' username group. These metadata allow both files and folders to become searchable using common search engines, i.e., Google or Bing.

Metadata templates for common metadata standards, e.g., the Dublin Core https://www.dublincore.org/ and DataCite [140, 141] provide pre-configured formats for users to apply standard AVUs to their data files and folders in the Discovery

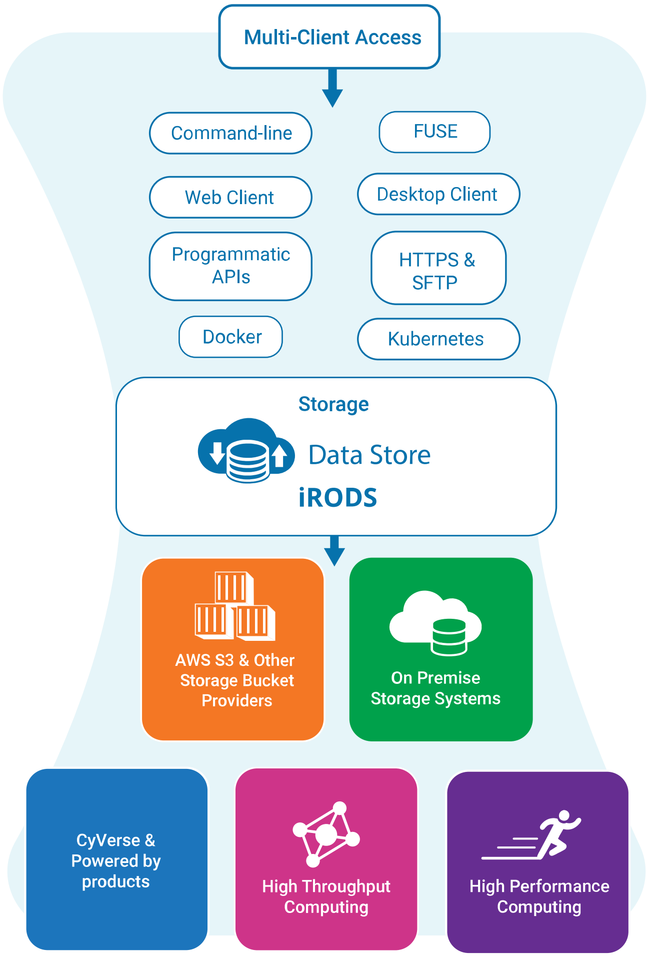


**Fig E. Data Store.** The iRODS data store is accessible from a variety of multi-client access end points. The resource servers that make-up the data store include on-premises storage servers at UArizona, as well as federated storage on commercial and public research clouds.

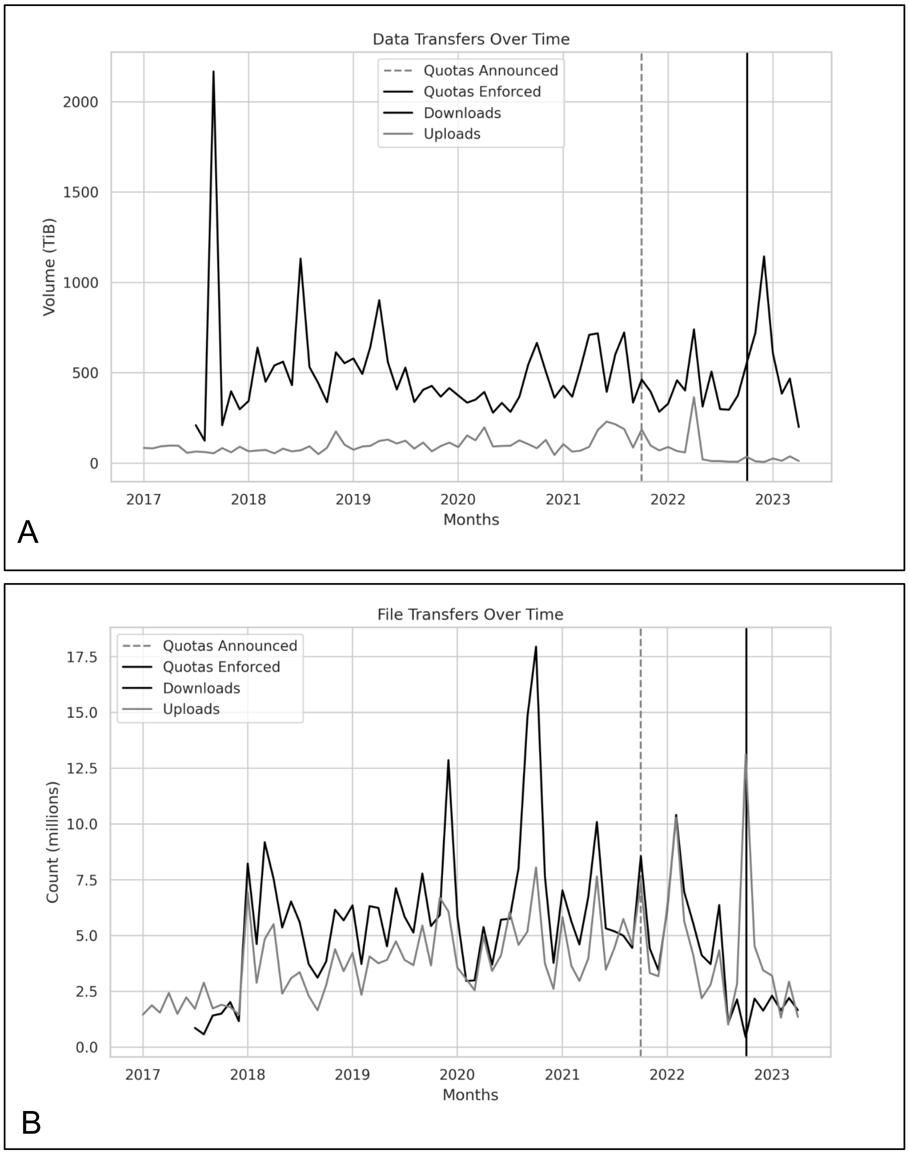


**Fig F. Data Store traffic.** Data Store transfers by CyVerse users total (TiB/month) data download and upload from the Data Store (Panel A), the number of files (million/month) downloaded and uploaded (Panel B).

Environment. The AVUs are visible publicly via the Data Commons website and programmatically through iRODS and its APIs.

**WebDAV** [132, 133] is an extension of the HTTP communication protocol that allows users to collaboratively manage files and folders stored remotely. The CyVerse WebDAV service provides a TLS encrypted WebDAV interface to the Data Store. It complies with the WebDAV Class 2 standard, meaning it supports standard file browser features like file reading/downloading, file writing/uploading, folder creation, etc.; plus it supports multi-user access features like file locking. A user may navigate the folder hierarchy and view data through a common web browser using this service. They may also use a common file browser like Windows File Explorer, MacOS X Finder, or any tool that understands WebDAV, to work with CyVerse files and folders as if they were local. Since WebDAV is a standard,open protocol with significant library support, a user may interact with the service programmatically. The service respects Data Store data access controls. For a user to access data that is not anonymously available, the user must authenticate using CyVerse credentials. Files downloaded through this service do not have the same tracking or internal analytics as files moved or downloaded using iRODS iCommands. However, downloading data sets consisting of many small files through this service can be many times faster than directly through iRODS due to caching. File caching is accomplished through an internal Varnish caching service [134]. For common use cases, the cache service has reduced data access times by 75%.

**DataWatch API** triggers code or runs workflows when specific "data events" take place. An example of this is when a specific file type is uploaded to a specific folder, or when an analysis completes, and its results are returned to the Data Store. DataWatch enables email notifications at pre-specified data events and will work in concert with event-driven webhooks to utilities or URLs.

**Data Commons** hosts both curated and community released data. The Data Commons can be used to publish (DataCite DOI) data for which there are no other existing canonical repositories or for which hosting on other research data services would not be feasible, such as very large datasets or data that need to be linked to analysis tools on CyVerse. Where appropriate, CyVerse encourages publication to canonical data repositories such as the National Center for Biotechnology Information (NCBI) [135–137]. CyVerse also provides tools for publishing sequence data directly to NCBI's Sequence Read Archive (SRA) [138, 139]. Curated data in the Data Commons are published via DataCite [140, 141] and receive a DOI. In addition to publication of static datasets with a DOI, shared data can exist as writable (editable) archives that can be modified by their owners in the Data Store's 'Community Released' projects. Community released data folders can be shared publicly as 'read-only' files over WebDav (https://data.cyverse.org) and the Data Commons (https://datacommons.cyverse.org) once shared with the 'anonymous' username group. Users can request DataCite DOI publication through the User Portal or by 'Publishing' their data in the Discovery Environment. Metadata are applied to community released and curated folders using the Discovery Environment's metadata template AVUs. The steps for requesting either a Community Released folder or a Curated folder are described in the user documentation (https://learning.cyverse.org/ds/doi/). Every Curated DOI requestor must complete the DataCite metadata template with required fields in the Discovery Environment. The template is submitted for review where CyVerse DOI experts communicates with the authors ensuring that all fields are complete and meet the DataCite standards. Once the DOI has been granted all write and own permissions are removed from the folder and it is transferred to the '/curated' folder space as 'read-only'.

**Federated Storage**, CyVerse allows its community to federate their own resource storage servers from their institutions or from within CyVerse facilities. The new

storage servers are added as 'resources' to the CyVerse iRODS zone, and can be kept accessible to only those community owners. These additional data storage devices can have their data replicated (mirrored) at UArizona and at TACC.

## Discovery Environment

The Discovery Environment (DE) is a multi-function 'data science workbench' with numerous applications designed for accessibility in the browser [142, 143]. The DE user interface features a table of contents on the left side which provides access to the user's data space and community data, to applications and their integration, and to running analyses, and training materials (Fig G). The DE allows users to upload or download data from the Data Store via their web browser over HTTP. Users can start scientific workflows via HTCondor, Tapis, OSG, and Kubernetes by selecting public applications, or integrate their own applications as private apps. The DE is Accessible Rich Internet Applications (ARIA) compliant for users with disabilities.

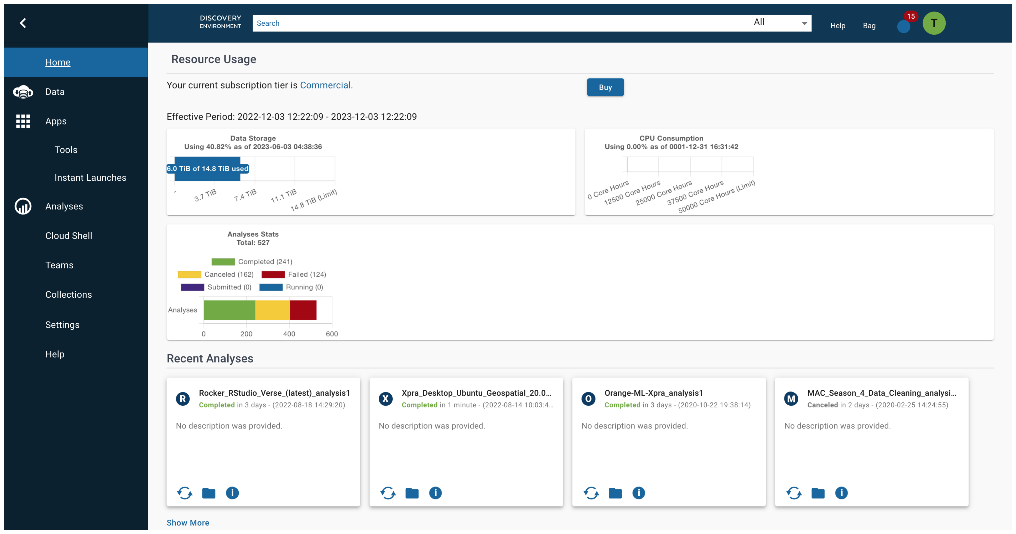


**Fig G. Discovery Environment User Interface.** The DE uses a table of contents menu (left side) with a collapsable hamburger menu. The Data Store, Apps, and Analyses can be viewed in the mainframe. Help, updates, and user profile features are visible in the upper right corner. Administrator accounts can approve access requests (VICE), edit public Apps and Tools, approve DOI Requests, and edit Reference Genomes in the table of contents (lower left).

The DE uses the concept of "Applications" for "Apps" which provide UI fields for input file paths, directories, or for abstracting command line interface (CLI) parameter fields. The "Tools" are for bringing-your-own-containers to the workbench. A "Tool" is a metadata template with information about a public Docker image: its metadata description, attribution, version, as well as cached public registry location and tag name. The Tool can (re)set the image's working directory, open ports, and change its entrypoint. Once these Tool parameters are established, an "App" can be created which will use a specific tool. Multiple types of FOSS programs can be integrated into the DE. These programs are defined as executable, interactive, high-throughput, or high-performance "Tools" [143] (S7 Table). Jobs are run on numerous different infrastructures, which are physically located at UArizona (CyVerse), at TACC, or on the Open Science Grid. Each application or "App" is managed by a different type of scheduler or job handler depending on its type.

Docker images from public container registries, i.e., DockerHub [152], GitHub Container Registry [153], QUAY.io [154], BioContainers.pro [155], NVIDIA GPU Cloud [156], can be integrated. When users wish to publish a DE App with the community, the Tool image is reviewed by CyVerse staff. Once approved, it is added to the Harbor [157] private container registry (Fig H). Public tools are cached on the DE's processing nodes and in a public/private Harbor registry maintained by CyVerse (Fig I). This enables dramatically faster launches, particularly of large containers typical of data science applications (e.g. JupyterLab with numerous Data Science Python libraries [48, 158], RStudio TidyVerse or Geospatial [47, 159, 160]) (Fig G). Tools that are integrated as high throughput Apps on OSG must be converted from Docker to Singularity [161]. These Singularity images are cached on the OSG's Cern Virtual File Management System (CVFMS) [162] scratch file system for rapid deployment across OSG's international network.

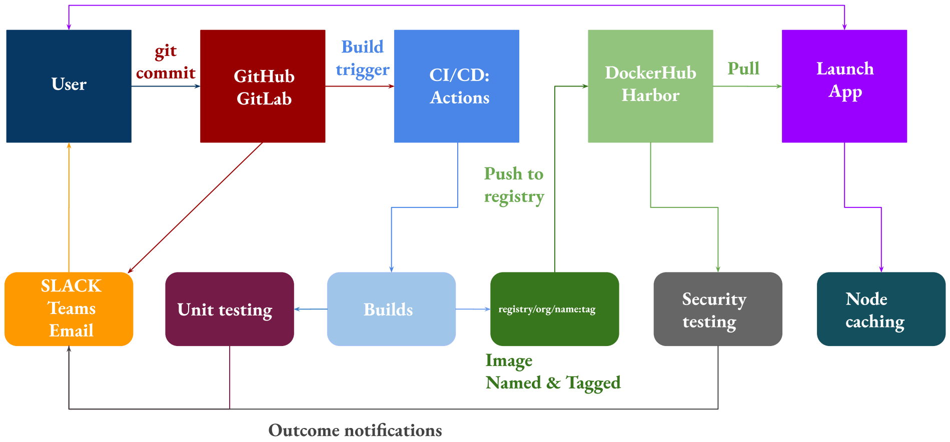


**Fig H. Featured container deployment.** CI/CD workflow for featured container applications in the Discovery Environment. Image recipes (Dockerfiles) are hosted on GitHub/GitLab and use build triggers with automation servers (GitHub Actions [163]) to build and tag images. Tested images are pushed to public and private registries on DockerHub and self-hosted Harbor. Images are cached on the DE production servers (nodes) for rapid deployment as containers at runtime.

**Executable Apps** are defined as non-interactive CLI applications that require an input, parameters, or flags, and defined output files and directory names. Example applications include the most common tools for genomic analyses [170], as well as applications written with scripted languages like Python and R. Executable jobs are managed by HTCondor and can be run individually, sequentially as a scientific workflow, or in parallel batches over a set of input files (Fig J).

**Interactive Apps** refer to any application which has its own GUI or IDE. Interactive Apps are deployed with Kubernetes, which provisions and launches the container on its own secure URL (Fig I). The **visual interactive compute environment (VICE)** component of the DE acts as a graphical interface for common IDE platforms such as RStudio [47], JupyterLab [146], Visual Studio Code [49], Remote Desktops (noVNC) [147]; browser based GUI such as R Shiny [164], Python Flask [165], Java [166], or JavaScript [167]. Community efforts to containerize browser-based RStudio-Server from the Rocker Project [159, 160] provide researchers with hundreds of libraries built by the global R community. Project Jupyter [48, 168] similarly supports a vast array of libraries written in Python, as well as other languages as add-in kernels. Remote Desktop applications (over HTTP) allow users to work in

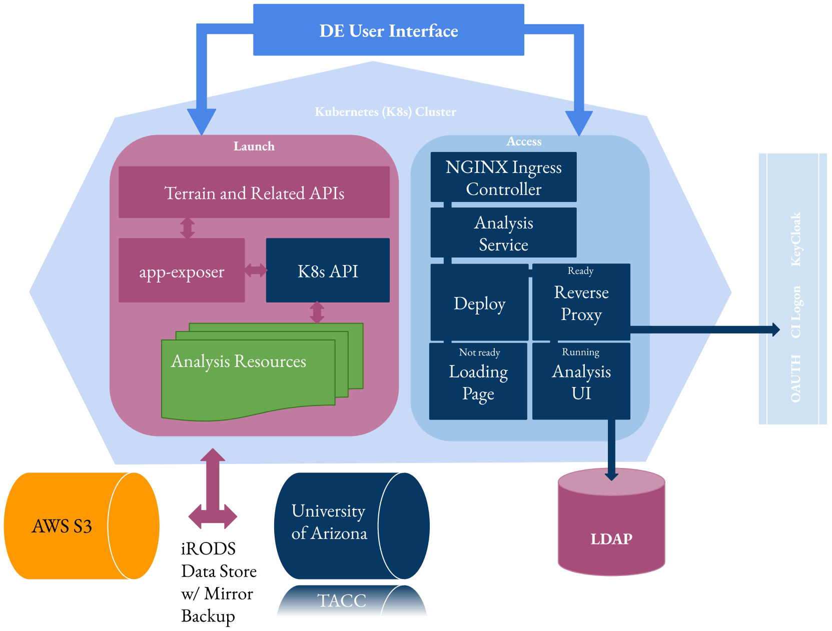


**Fig I. Discovery Environment Interactive.** Interactive jobs include GUI apps like RStudio and JupyterLab. The DE manages interactive jobs through Kubernetes (K8s) and its Terrain API. Access to apps are managed by an Ingress Controller (NGINX [169]). The analysis service shows whether the app is deployed, loading, or currently running and loads the UI for the analysis. Central authentication is managed by CAS. Users can load data from the iRODS datastore into their running containers. LDAP manages the user's secure authentication. Data Store is mirrored nightly at TACC from UArizona.

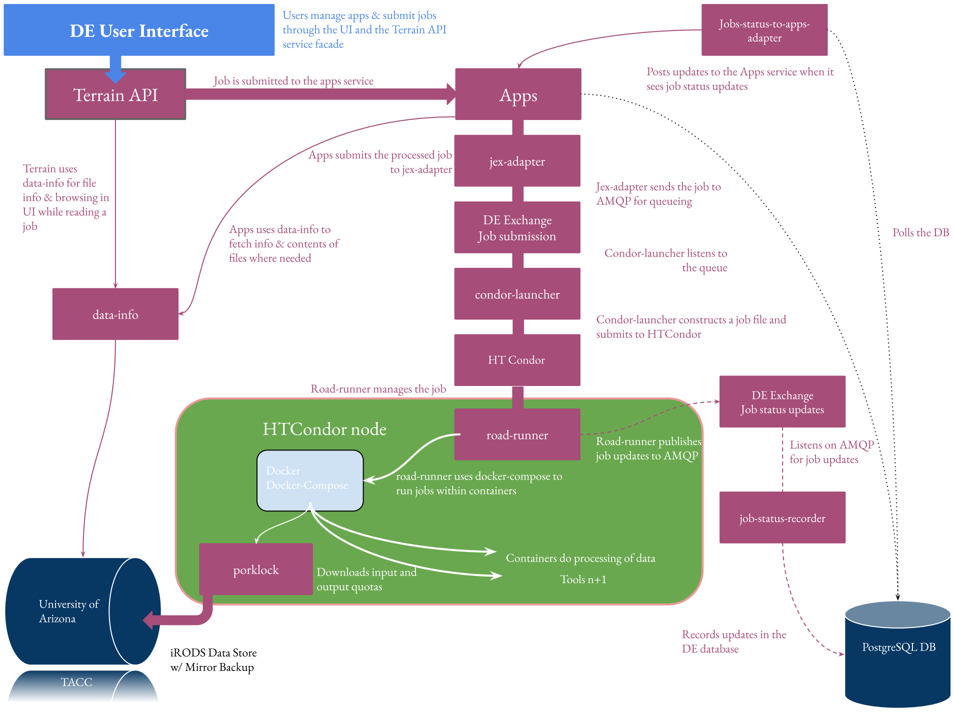


**Fig J. Discovery Environment Executable.** Executable (command line interface driven) Apps are managed by HTCondor and the Terrain API. Jobs are submitted through the DE user interface where they trigger a job submission service managed by HTCondor with Advanced Message Queueing Protocol (AMQP). Once the job runs, it is sent to a node where a program called RoadRunner uses Docker-Compose to manage the execution. Data are copied back to the iRODS data store when the app completes using a custom program called porklock (https://github.com/cyverse-de/porklock). A PostgreSQL database monitors all job status and outcomes.

their familiar desktop environments, and extended applications allow for the server-side hardware rendering of large 3D visualizations with GPU.

**High Performance Apps** are defined as jobs which require multiple nodes using message passing interface (MPI) [171] or Open Multi-Processing (OpenMP) [172]. High performance apps are managed by TACC's Tapis (formerly Agave API) [173, 174] and run at TACC relying on CyVerse Data Store mirror for faster data throughput. CyVerse has integration with the OSG via HTCondor, allowing users to deploy High Throughput Apps onto the OSG.

Three levels of metadata are operated by the DE: (1) Data Store object metadata are managed by iRODS, (2) PostgreSQL which manages User, Tool, and App metadata which is used by the featured search bar (Fig G), (3) Metadata Templates applied through the DE to Data Store objects, storing supplemental metadata in PostgreSQL, for application in the Data Commons.

The DE brokers data access to the iRODS Data Store. When users run an App, the DE transfers input data from the Data Store to the local Linux file system. The App can also access the remote input data as if they are mounted in the local file system without making manual data transfers. When the job (HTCondor or Kubernetes) completes, the output data are written back to the Data Store. By default, all analysis data are saved under the user's /analyses folder, into a folder defaulting to the name of the app with the date and time of the application launch. The user can modify the name of the analyses' output folder and change its location within the Data Store.

The DE uses two CyVerse-developed open-software packages, iRODS FUSE Lite and an iRODS CSI driver, to broker data access to the Data Store. iRODS FUSE Lite is a tool that mounts data stored in the iRODS data store (e.g., the Data Store) on the local Linux file system and provides on-demand data access. The iRODS CSI driver manages the mounts in Kubernetes and facilitates data access in Tools. The DE integrates the Data Store using the software.

### Publishing custom Tools and Apps

The only prerequisite to user's publishing their own private container apps is that the container image for the app must reside in a Docker configured to be trusted by the Discovery Environment, i.e., Harbor (https://harbor.cyverse.org). If the app publishing request container image is not in a trusted registry then the request is sent to Discovery Environment administrators as a notification. This process allows administrators to review the container image before allowing the app to be published. If the container used by the app already resides in one of the registries that are trusted by the Discovery Environment, then the app is made available immediately.

Forcing apps to be in a trusted registry provides a few important benefits. First, it allows administrators to inspect any container images that aren't already in one of the trusted registries for vulnerabilities. Second, it allows administrators to ensure that different image tags are used for different versions of the same container image, which helps to ensure reproducibility. Third, it can help to avoid errors caused by rate limiting from third party registries. Whether or not administrator intervention is required to publish the app, all newly published apps are tagged as Beta apps. Only administrators can remove this tag, so users of the app can request an inspection before using the app if they want.

## Cloud-Native Services

"Cloud-native" [12, 20, 175] has a narrower definition than "cloud-based" services. Cloud-native generally refers to leveraging existing cloud with container-based

environments which greatly reduce the time to set up or deploy, versus cloud-based services, which require provisioning and software stack installation.

CyVerse has successfully managed cloud-based services for over ten years. In the last five years, these have included an OpenStack web-based interface called "Atmosphere," which has become the Jetstream production research cloud. Currently, CyVerse is developing cloud-native applications for managing larger and more dynamic cloud deployments, including Kubernetes (K8s) [43], Lightweight Kurbernetes [176](Rancher K3s), Argo workflows [177], and Terraform [178,179]. These products are in support of the Jetstream2, both as backend services and a new web-based UI.

# How challenges are met

In 2009, grand challenges facing the Plant Sciences community included (1) assembling, visualizing and analyzing the Tree of Life (AVAToL) [158] with all 1.7 million species, (2) the exploration of genotype-to-phenotype, i.e., genotype crossed by environment equals phenotype (G×E=P), and (3) the curation with digital images of herbaria records estimated at the time to be 500 million. Critically, the iPlant Collaborative was not established to do any type of primary data collection, but rather to develop the cyberinfrastructure aspects of the challenge. In the early years of the iPlant Collaborative project, there were heated discussions amongst the community at large about the NSF's decision to support cyberinfrastructure without associated data collection. The outcome was that iPlant would focus on cyberinfrastructure: providing software and hardware solutions which link to community data which could lead to scientific discovery and transformative outcomes. Significant portions of the original iPlant Collaborative budget went to supporting travel and to developing synthesis collaborations amongst data curation and collection programs to the cyberinfrastructure (e.g., Taxonomic Name Resolution Service [TNRS] [159], Botanical Information and Ecology Network [BIEN] [160,161])[4]. Examples of big data projects still hosted in the CyVerse infrastructure include the USDA NIFA AG2PI Collaborative [162], NSF GenoPhenoEnvo [163], and PhytoOracle [164]. Notable examples of projects which do collect large-scale life science data that are leveraged within CyVerse include the National Ecological Observatory Network (NEON) [165], Genome to Fields (G2P) [166–169], TerraREF [170], National Phenology Network (NPN) [171], Long Term Agricultural Research (LTAR) [172], and Long Term Ecological Research (LTER) [173] networks. Many of these data are now supported by the Ecological Data Initiative (EDI) [174], which collaborates with CyVerse to make ecological data more FAIR.

### Migration to Commercial Cloud versus remaining On-Premise

When research data are hosted in separate geographic locations connected across the internet, they often require specialized data transfers [180,181]. Research data can be so large they require physically carrying or transporting the data storage devices to other computing facilities (so-called "walking networks" or "sneaker-net") so they can be analyzed. This can be because the overall bandwidth of the internet available to the researchers is too small [182,183] or the data formats are not optimized for cloud-native processing [12]. A growing number of research projects rely on more computational hardware than may be available at any one institution [184–186]. Local compute clusters have given way to high performance computing, grid computing, and cloud computing. Commercial cloud services have only existed for 15 years [187], while internet connected mobile phones have only existed for 14 years [188]. At-cost and small-loss pricing for cloud data hosting has become a lure for bringing publicly funded research and governmental data into commercial cloud. Profits are made on computing

and data egress from such services. As of 2021, cloud is the largest revenue producer for Microsoft (Azure) [189] and the most profitable sector for Amazon (Amazon Web Services) [190]. This massive shift toward cloud computing globally, along with the need for research objects, explains why 'cloud-native science' is part of our shared future, with the promise of reducing our time-to-science and increasing the overall pace of scientific discovery. However, it is not without risk or potentially high cost of ownership.

Other than CyVerse, large tech companies are the only entities with development teams large enough to create and operate middleware for managing cyberinfrastructure for research objects **??**. However, tech companies need to (eventually) make a profit for their shareholders and thus may change the availability of free services to paid. Without more and larger investments into public cyberinfrastructure from state and national funding entities for science and education, researchers will increasingly turn to commercial cloud to run their science experiments at scale, which will increase the divide between those with and without financial resources [191]. As researchers move toward commercial cloud, they move away from open-science and toward commercialized data access.

While the capability of commercial cloud is not in question, the price is. The cogent question is whether managing hardware on-premise is the most valuable use of financial resources for state- and national-scale research [80, 81, 83]. In the effort to address this question and remain agile, CyVerse's SaaS and IaC are designed to be run anywhere (cloud-agnostic). If, in the future, the consensus about where to operate research computing on commercial or on-premise hardware changes, CyVerse operations could be moved. However, for the present, the financial requirements of moving data and analyses fully onto commercial clouds are beyond CyVerse's research funding capacity.

### Generative AI revolution

With the emergence of generative AI and Large Language Models (LLMs) from OpenAI (ChatGPT), Google (Bard), Meta (LLaMA), and model repositories such as HuggingFace, anyone with internet access can now leverage AI-assisted programming and general work productivity. Early research reports suggest that AI assistants can improve programming and general productivity by over 50%. CyVerse already supports LLMs and integrates AI extensions into its workbench. The CyVerse Data Store can host trained models and training data, run popular applications (e.g., HuggingFace hosted Apps) in its workbench, or distribute larger model training processes to publicly available HPC/HTC and cloud platforms with GPU hardware at no cost to academic researchers (through the NSF's ACCESS-CI program).

## User Demographics continued

Distribution of registered users globally and in the United States (Fig K).

Of all users, 71% are from North America, 12% Europe, 10% Asia, 3% Africa, 2% South America, and 1% Oceana (Fig **??**). The majority of users self-identify as graduate and undergraduate students (Fig 4 in main text), which is not surprising given the workload distribution of modern research and CyVerse's focus on student training. By race, users identify as White, Hispanic or Latino (27.4%) and White including Arabic (17.5%), Asian or Pacific Islander (18.5%), African American or Black (6.0%), and American Indian, Alaskan Native, or Hawaiian Native (0.6%). Undefined categories included "Other" (6.6%), "Not Provided" (8.4%), and "Declined to Provide" (15.1%).

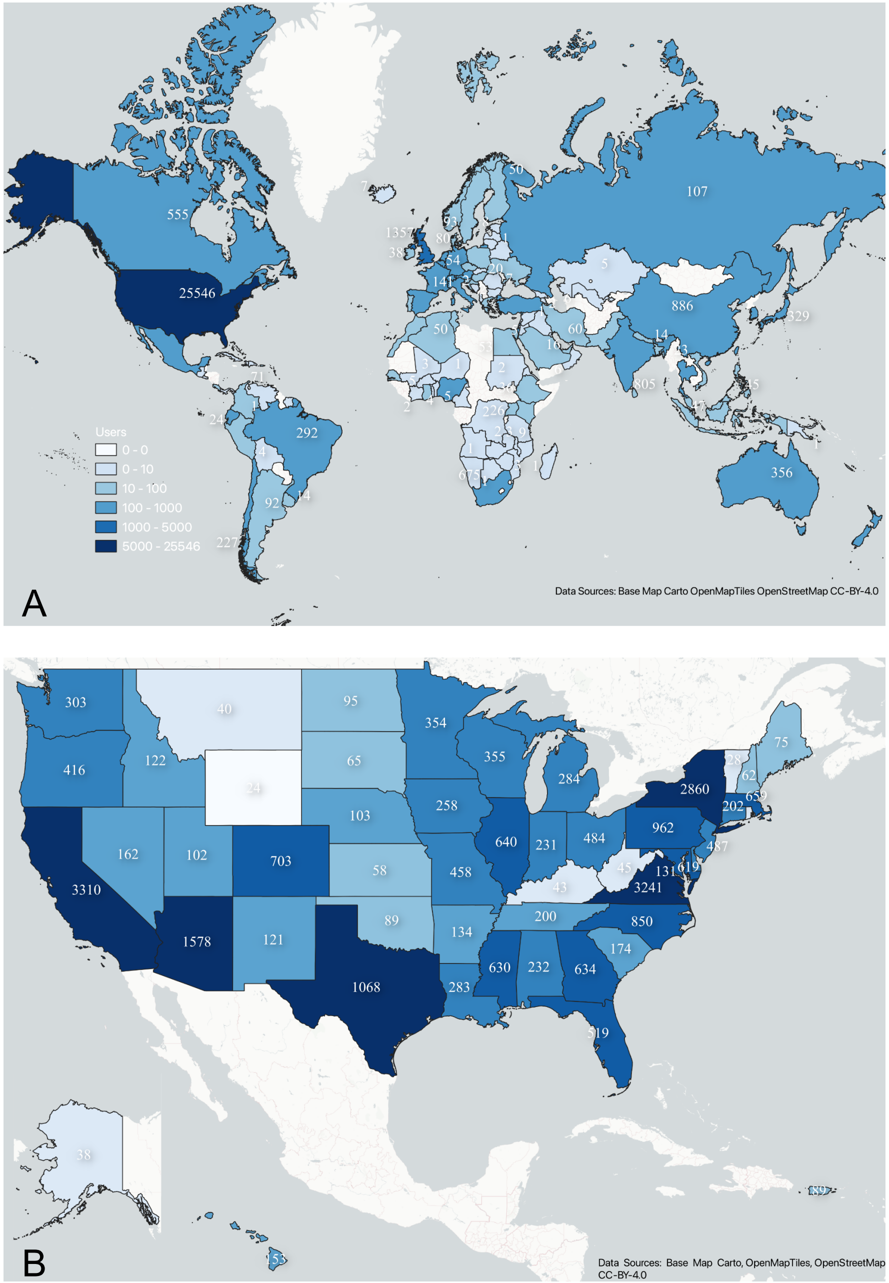


**Fig K. Global and USA distribution of CyVerse user accounts.** CyVerse registered accounts, by country (Panel A) and by US state (Panel B). Base-map from Carto and OpenStreetMap CC-BY 4.0 license (https://github.com/CartoDB/basemap-styles).

# References


1. Goff SA, Vaughn M, McKay S, Lyons E, Stapleton AE, Gessler D, et al. The iPlant Collaborative: Cyberinfrastructure for Plant Biology. Front Plant Sci. 2011;2:34.

2. Merchant N, Lyons E, Goff S, Vaughn M, Ware D, Micklos D, et al. The iPlant Collaborative: Cyberinfrastructure for Enabling Data to Discovery for the Life Sciences. PLoS Biol. 2016;14:e1002342.

3. Crowston K, Howison J. The social structure of free and open source software development. First Monday. 2005 [cited 15 Aug 2021]. doi:10.5210/fm.v10i2.1207

4. von Krogh G, von Hippel E. The Promise of Research on Open Source Software. Manage Sci. 2006;52:975–983.

5. Scacchi W, Feller J, Fitzgerald B, Hissam S, Lakhani K. Understanding free/open source software development processes. Softw Process Improv Pract. 2006;11:95–105.

6. Stewart TA. Intellectual Capital: The new wealth of organization. Crown; 2010.

7. Peng RD. Reproducible research in computational science. Science. 2011;334:1226–1227.

8. Wilkinson MD, Dumontier M, Aalbersberg IJJ, Appleton G, Axton M, Baak A, et al. The FAIR Guiding Principles for scientific data management and stewardship. Sci Data. 2016;3:160018.

9. Understanding Data Motion in the Modern HPC Data Center. [cited 9 May 2023]. Available: `https://ieeexplore.ieee.org/abstract/document/8955242`

10. Barone L, Williams J, Micklos D. Unmet needs for analyzing biological big data: A survey of 704 NSF principal investigators. PLoS Comput Biol. 2017;13:e1005755.

11. Gentemann CL, Holdgraf C, Abernathey R, Crichton D, Colliander J, Kearns EJ, et al. Science storms the cloud. AGU Advances. 2021;2. doi:10.1029/2020av000354

12. Abernathey RP, Augspurger T, Banihirwe A, Blackmon-Luca CC, Crone TJ, Gentemann CL, et al. Cloud-Native Repositories for Big Scientific Data. Computing in Science Engineering. 2021;23:26–35.

13. Buck S. Solving reproducibility. Science. 2015;348:1403.

14. Plesser HE. Reproducibility vs. Replicability: A Brief History of a Confused Terminology. Front Neuroinform. 2017;11:76.

15. Fairlie RW. Race and the Digital Divide. Contrib Econ Analysis Policy. 2004;3. doi:10.2202/1538-0645.1263

16. Norris P. The digital divide. Routledge; 2020.

17. Kitchin R. The Data Revolution: Big Data, Open Data, Data Infrastructures and Their Consequences. SAGE; 2014.

18. Michonneau F, Paul D. Scaling Up Data Literacy and Computing Skills Training in Biodiversity Science, Lessons Learned from The Carpentries. Biodiversity Information Science and Standards; Sofia. 2019. doi:10.3897/biss.3.35108

19. Hampton SE, Anderson SS, Bagby SC, Gries C, Han X, Hart EM, et al. The Tao of open science for ecology. Ecosphere. 2015;6:1–13.

20. Kratzke N, Quint P-C. Understanding cloud-native applications after 10 years of cloud computing - A systematic mapping study. J Syst Softw. 2017;126:1–16.

21. Ramachandran R, Bugbee K, Murphy K. From open data to open science. Earth Space Sci. 2021;8. doi:10.1029/2020ea001562

22. Understanding Data Motion in the Modern HPC Data Center. [cited 9 May 2023]. Available: https://ieeexplore.ieee.org/abstract/document/8955242

23. Boomija MD, Raja SVK. Securing medical data by role-based user policy with partially homomorphic encryption in AWS cloud. Soft Computing. 2022;27:559–568.

24. Understanding Data Motion in the Modern HPC Data Center. [cited 9 May 2023]. Available: https://ieeexplore.ieee.org/abstract/document/8955242

25. Mesirov JP. Computer science. Accessible reproducible research. Science. 2010;327:415–416.

26. Munafò MR, Nosek BA, Bishop DVM, Button KS, Chambers CD, Percie du Sert N, et al. A manifesto for reproducible science. Nature Human Behaviour. 2017;1:0021.

27. NIH Data Sharing Policy and implementation guidance. [cited 10 Sep 2021]. Available: https://grants.nih.gov/grants/policy/data_sharing/data_sharing_guidance.htm

28. Open Data at NSF. [cited 10 Sep 2021]. Available: https://www.nsf.gov/data/

29. Belhajjame K, Corcho O, Garijo D, Zhao J, Missier P, Newman DR, et al. Workflow-Centric Research Objects: A First Class Citizen in the Scholarly Discourse. SePublica@ ESWC. users.ox.ac.uk; 2012. pp. 1–12.

30. Hettne KM, Dharuri H, Zhao J, Wolstencroft K, Belhajjame K, Soiland-Reyes S, et al. Structuring research methods and data with the research object model: genomics workflows as a case study. J Biomed Semantics. 2014;5:41.

31. Edmunds SC, Li P, Hunter CI, Xiao SZ, Davidson RL, Nogoy N, et al. Experiences in integrated data and research object publishing using GigaDB. International Journal on Digital Libraries. 2017;18:99–111.

32. Palma R, Garcia-Silva A, Gomez-Perez JM, Krystek M. A Research Object-Based Toolkit to Support the Earth Science Research Lifecycle. 2018 IEEE 14th International Conference on e-Science (e-Science). ieeexplore.ieee.org; 2018. pp. 50–57.

33. Bucksch A, Das A, Schneider H, Merchant N, Weitz JS. Overcoming the Law of the Hidden in Cyberinfrastructures. Trends Plant Sci. 2017;22:117–123.

34. Sahneh F, Balk MA, Kisley M, Chan C-K, Fox M, Nord B, et al. Ten simple rules to cultivate transdisciplinary collaboration in data science. PLoS Comput Biol. 2021;17:e1008879.

35. Carroll SR, Garba I, Figueroa-Rodríguez OL, Holbrook J, Lovett R, Materechera S, et al. The CARE principles for indigenous data governance. Data Sci J. 2020;19. doi:10.5334/dsj-2020-043

36. Kelling S, Hochachka WM, Fink D, Riedewald M, Caruana R, Ballard G, et al. Data-intensive Science: A New Paradigm for Biodiversity Studies. Bioscience. 2009;59:613–620.

37. Faris J, Kolker E, Szalay A, Bradlow L, Deelman E, Feng W, et al. Communication and data-intensive science in the beginning of the 21st century. OMICS. 2011;15:213–215.

38. Wolf F, Hobby R, Lowry S, Bauman A, Franza BR, Lin B, et al. Education and data-intensive science in the beginning of the 21st century. OMICS. 2011;15:217–219.

39. Choudhary V. Software as a Service: Implications for Investment in Software Development. 2007 40th Annual Hawaii International Conference on System Sciences (HICSS'07). 2007. p. 209a–209a.

40. Morris K. Infrastructure as Code: Managing Servers in the Cloud. "O'Reilly Media, Inc."; 2016.

41. Ansible RH. Ansible is Simple IT Automation. [cited 11 Sep 2021]. Available: https://www.ansible.com/

42. Argo Workflows - The workflow engine for Kubernetes. [cited 11 Sep 2021]. Available: https://argoproj.github.io/argo-workflows/

43. Bernstein D. Containers and Cloud: From LXC to Docker to Kubernetes. IEEE Cloud Computing. 2014;1:81–84.

44. Terraform by HashiCorp. [cited 11 Sep 2021]. Available: https://www.terraform.io/

45. Beck M, Moore T. The Internet2 Distributed Storage Infrastructure project: an architecture for Internet content channels. Computer Networks and ISDN Systems. 1998;30:2141–2148.

46. Thain D, Tannenbaum T, Livny M. Distributed computing in practice: the Condor experience. Concurr Comput. 2005;17:323–356.

47. Team R, Others. RStudio: integrated development for R. RStudio, Inc, Boston, MA URL http://www.rstudio.com. 2015;42.

48. Perez F, Granger BE. Project Jupyter: Computational narratives as the engine of collaborative data science. Retrieved September. 2015;11:108.

49. Sole AD, Del Sole A. Introducing Visual Studio Code. Visual Studio Code Distilled. 2019. pp. 1–17. doi:10.1007/978-1-4842-4224-7_1

50. Conversational relationship platform. [cited 9 Sep 2021]. Available: https://www.intercom.com/

51. Wilson G. Software Carpentry: Getting Scientists to Write Better Code by Making Them More Productive. Computing in Science Engineering. 2006;8:66–69.

52. Wilson G. Software Carpentry: lessons learned. F1000Res. 2014;3:62.

53. Pugachev S. What are "the carpentries" and what are they doing in the library? Portal. 2019;19:209–214.

54. CyVerse UK. [cited 11 Sep 2021]. Available: `https://cyverseuk.org/`

55. Minotto A, Van Den Bergh E, Davey RP. CyVerse UK: Widening the Scope to the UK and Beyond. Plant and Animal Genome XXVI Conference (January 13-17, 2018). PAG; 2018. Available: `https://pag.confex.com/pag/xxvi/meetingapp.cgi/Paper/31449`

56. Lang K, Stryeck S, Bodruzic D, Stepponat M, Trajanoski S, Winkler U, et al. CyVerse Austria—A Local, Collaborative Cyberinfrastructure. Math Comput Appl. 2020;25:38.

57. Wieser F, Stryeck S, Lang K, Hahn C, Thallinger G, Feichtinger J, et al. A local platform for user-friendly FAIR data management and reproducible analytics. Journal of Biotechnology. 2021. doi:10.1016/j.jbiotec.2021.08.004

58. BioTechMed-Graz. [cited 11 Sep 2021]. Available: `https://biotechmedgraz.at/de/`

59. RDM - TU Graz Framework Policy for RDM. [cited 11 Sep 2021]. Available: `https://www.tugraz.at/sites/rdm/policies/tu-graz-framework-policy-for-rdm/`

60. Research Data Management. [cited 11 Sep 2021]. Available: `https://ub.uni-graz.at/en/services/publication-services/research-data-management/`

61. Austrian DataLAB and Services - Cluster Forschungsdaten. 11 May 2020 [cited 11 Sep 2021]. Available: `https://forschungsdaten.at/adls/`

62. The Event Horizon Telescope Collaboration. First M87 EHT results: Calibrated data. CyVerse Data Commons; 2019. doi:10.25739/G85N-F134

63. Morzinski KM, Close LM, Males JR, Kopon D, Hinz PM, Esposito S, et al. MagAO: Status and on-sky performance of the Magellan adaptive optics system. Adaptive Optics Systems IV. International Society for Optics and Photonics; 2014. p. 914804.

64. Ramírez-Andreotta MD, Walls R, Youens-Clark K, Blumberg K, Isaacs KE, Kaufmann D, et al. Alleviating Environmental Health Disparities Through Community Science and Data Integration. Front Sustain Food Syst. 2021;5. doi:10.3389/fsufs.2021.620470

65. Olschanowsky C, Maxwell RM, Condon LE, Strout M, Altintas I, Purawat S, et al. Hydroframe: A Software Framework to enable Continental Scale Hydrologic Simulation. 2019. p. A11A–01.

66. Furfaro R, Linares R, Gaylor D, Jah M, Walls R. Resident space object characterization and behavior understanding via machine learning and ontology-based Bayesian networks. Advanced Maui Optical and Space Surveillance Tech Conf(AMOS). amostech.com; 2016. Available: https://amostech.com/TechnicalPapers/2016/SSA-Algorithms/Furfaro.pdf

67. Walls RL, Gaylor D, Reddy V, Furfaro R, Jah M. Assessing the IADC Space Debris Mitigation Guidelines: A case for ontology-based data management. AMOS Paper. 2016. Available: https://amostech.com/TechnicalPapers/2016/SSA/Walls.pdf

68. Reddy V, Linder T, Linares R, Furfaro R, Tucker S, Campbell T. RAPTORS: Hyperspectral Survey of the GEO Belt. AMOS Technologies Conference, Maui Economic Development Board, Kihei, Maui, HI. amostech.com; 2018. Available: https://amostech.com/TechnicalPapers/2018/NROC/Reddy.pdf

69. Carlson O, Hohenstein S, Bui J, Tanquary H, Fritz C, Gross DC. Human Factors in the Unified Architecture Framework Applied to Space Situational Awareness. 2019 IEEE International Systems Conference (SysCon). ieeexplore.ieee.org; 2019. pp. 1–7.

70. Rathje EM, Dawson C, Padgett JE, Pinelli J-P, Stanzione D, Adair A, et al. DesignSafe: New cyberinfrastructure for natural hazards engineering. Nat Hazards Rev. 2017;18:06017001.

71. Tarboton DG, Idaszak R, Horsburgh JS, Heard J, Ames D, Goodall JL, et al. HydroShare: Advancing Collaboration through Hydrologic Data and Model Sharing. International Congress on Environmental Modelling and Software. 2014. Available: https://scholarsarchive.byu.edu/iemssconference/2014/Stream-A/7/

72. Purawat S, Olschanowsky C, Condon LE, Maxwell R, Altintas I. Scalable Workflow-Driven Hydrologic Analysis in HydroFrame. Computational Science – ICCS 2020. Springer International Publishing; 2020. pp. 276–289.

73. Swetnam TL, Pelletier JD, Rasmussen C, Callahan NR, Merchant N, Lyons E, et al. Scaling GIS Analysis Tasks from the Desktop to the Cloud Utilizing Contemporary Distributed Computing and Data Management Approaches: A Case Study of Project-based Learning and Cyberinfrastructure Concepts. Proceedings of the XSEDE16 Conference on Diversity, Big Data, and Science at Scale. New York, NY, USA: ACM; 2016. pp. 21:1–21:6.

74. Hancock DY, Stewart CA, Vaughn M, Fischer J, Lowe JM, Turner G, et al. Jetstream-Early operations performance, adoption, and impacts: Early Jetstream Performance and Results. Concurr Comput. 2018;57:e4683.

75. Mass open cloud – an open cloud exchange public cloud. [cited 11 Sep 2021]. Available: https://massopen.cloud/

76. Jackson LA, Zhao Y, Kolenic A 3rd, Fitzgerald HE, Harold R, Von Eye A. Race, gender, and information technology use: the new digital divide. Cyberpsychol Behav. 2008;11:437–442.

77. Sisneros L, Sponsler BA. Broadband access and implications for efforts to address equity gaps in postsecondary attainment. Education Commission of the States. 2016 [cited 19 Jun 2021]. Available: http://files.eric.ed.gov/fulltext/ED565437.pdf

78. Brown V. Technology Access Gap for Postsecondary Education: A Statewide Case Study. In: Promoting Global Competencies Through Media Literacy. IGI Global; 2018. pp. 20–40.

79. Nelson A. Office of science and technology policy (OSTP) memorandum on access to federal research. 2022 [cited 25 Mar 2023]. Available: `https://policycommons.net/artifacts/3159884/08-2022-ostp-public-access-memo/3957772/`

80. Stewart CA, Hancock DY, Wernert J, Link MR, Wilkins-Diehr N, Miller T, et al. Return on Investment for Three Cyberinfrastructure Facilities: A Local Campus Supercomputer, the NSF-Funded Jetstream Cloud System, and XSEDE (the eXtreme Science and Engineering Discovery Environment). 2018 IEEE/ACM 11th International Conference on Utility and Cloud Computing (UCC). IEEE; 2018. pp. 223–236.

81. Stewart CA, Apon A, Hancock DY, Furlani T, Sill A, Wernert J, et al. Assessment of non-financial returns on cyberinfrastructure: A survey of current methods. Proceedings of the Humans in the Loop: Enabling and Facilitating Research on Cloud Computing. New York, NY, USA: Association for Computing Machinery; 2019. pp. 1–10.

82. Stewart CA, Hancock DY, Wernert J, Furlani T, Lifka D, Sill A, et al. Assessment of financial returns on investments in cyberinfrastructure facilities: A survey of current methods. Proceedings of the Practice and Experience in Advanced Research Computing on Rise of the Machines (learning). New York, NY, USA: Association for Computing Machinery; 2019. pp. 1–8.

83. Chalker A, Hillegas CW, Sill A, Broude Geva S, Stewart CA. Cloud and on-premises data center usage, expenditures, and approaches to return on investment: A survey of academic research computing organizations. Practice and Experience in Advanced Research Computing. New York, NY, USA: Association for Computing Machinery; 2020. pp. 26–33.

84. Atkins DE. Revolutionizing Science and Engineering Through Cyberinfrastructure: Report of the National Science Foundation Blue-Ribbon Advisory Panel on Cyberinfrastructure. National Science Foundation; 2003.

85. Hacker TJ, Wheeler BC. Making research cyberinfrastructure a strategic choice. Educause Quarterly. 2007;30:21.

86. Shi W, Cao J, Zhang Q, Li Y, Xu L. Edge Computing: Vision and Challenges. IEEE Internet of Things Journal. 2016;3:637–646.

87. Satyanarayanan M. The Emergence of Edge Computing. Computer. 2017;50:30–39.

88. Willis C, Lambert M, McHenry K, Kirkpatrick C. Container-based Analysis Environments for Low-Barrier Access to Research Data. Proceedings of the Practice and Experience in Advanced Research Computing 2017 on Sustainability, Success and Impact. New York, NY, USA: ACM; 2017. pp. 58:1–58:4.

89. Orgogozo V, Morizot B, Martin A. The differential view of genotype-phenotype relationships. Front Genet. 2015;6:179.

90. Gonzalez EM, Zarei A, Hendler N, Simmons T, Zarei A, Demieville J, et al. PhytoOracle: Scalable, modular phenomics data processing pipelines. Front Plant Sci. 2023;14:1112973.

91. Kvilekval K, Fedorov D, Obara B, Singh A, Manjunath BS. Bisque: a platform for bioimage analysis and management. Bioinformatics. 2010;26:544–552.

92. Fedorov DV, Kvilekval KG, Doheny B, Sampson S, Miller RJ, Manjunath BS. Deep learning for all: managing and analyzing underwater and remote sensing imagery on the web using BisQue. 2017 [cited 27 Aug 2021]. Available: `https://escholarship.org/uc/item/9z73t7hv`

93. Polonsky AT, Lang CA, Kvilekval KG, Latypov MI, Echlin MP, Manjunath BS, et al. Three-dimensional Analysis and Reconstruction of Additively Manufactured Materials in the Cloud-Based BisQue Infrastructure. Integrating Materials and Manufacturing Innovation. 2019;8:37–51.

94. Latypov MI, Khan A, Lang CA, Kvilekval K, Polonsky AT, Echlin MP, et al. BisQue for 3D Materials Science in the Cloud: Microstructure–Property Linkages. Integrating Materials and Manufacturing Innovation. 2019;8:52–65.

95. Lyons EH. CoGe, a new kind of comparative genomics platform: Insights into the evolution of plant genomes. Freeing M, editor. University of California, Berkeley. 2008. Available: `http://ezproxy.library.arizona.edu/login?url=https://www.proquest.com/dissertations-theses/coge-new-kind-comparative-genomics-platform/docview/304696692/se-2`

96. Castillo AI, Nelson ADL, Haug-Baltzell AK, Lyons E. A tutorial of diverse genome analysis tools found in the CoGe web-platform using Plasmodium spp. as a model. Database. 2018;2018. doi:10.1093/database/bay030

97. Lyons E, Pedersen B, Kane J, Alam M, Ming R, Tang H, et al. Finding and comparing syntenic regions among Arabidopsis and the outgroups papaya, poplar, and grape: CoGe with rosids. Plant Physiol. 2008;148:1772–1781.

98. Haug-Baltzell A, Stephens SA, Davey S, Scheidegger CE, Lyons E. SynMap2 and SynMap3D: web-based whole-genome synteny browsers. Bioinformatics. 2017;33:2197–2198.

99. Hilgert U, McKay S, Khalfan M, Williams J, Ghiban C, Micklos D. DNA Subway: Making Genome Analysis Egalitarian. Proceedings of the 2014 Annual Conference on Extreme Science and Engineering Discovery Environment. New York, NY, USA: Association for Computing Machinery; 2014. pp. 1–3.

100. Wang L, Lu Z, Van Buren P, Ware D. SciApps: a cloud-based platform for reproducible bioinformatics workflows. Bioinformatics. 2018;34:3917–3920.

101. Wang L, Lu Z, delaBastide M, Van Buren P, Wang X, Ghiban C, et al. Management, Analyses, and Distribution of the MaizeCODE Data on the Cloud. Front Plant Sci. 2020;11:289.

102. Wang L, Lu Z, Regulski M, Jiao Y, Chen J, Ware D, et al. BSAseq: an interactive and integrated web-based workflow for identification of causal mutations in bulked F2 populations. doi:10.1101/2020.04.08.029801

103. Pascu C, Osimo D, Ulbrich M, Turlea G, Burgelman JC. The potential disruptive impact of Internet2 based technologies. First Monday. 2007;12. doi:10.5210/fm.v12i3.1630

104. Towns J, Cockerill T, Dahan M, Foster I, Gaither K, Grimshaw A, et al. XSEDE: Accelerating Scientific Discovery. Comput Sci Eng. 2014;16:62–74.

105. Bockelman B, Cartwright T, Frey J, Fajardo EM, Lin B, Selmeci M, et al. Commissioning the HTCondor-CE for the Open Science Grid. J Phys Conf Ser. 2015;664:062003.

106. Stewart CA, Cockerill TM, Foster I, Hancock D, Merchant N, Skidmore E, et al. Jetstream: a self-provisioned, scalable science and engineering cloud environment. Proceedings of the 2015 XSEDE Conference: Scientific Advancements Enabled by Enhanced Cyberinfrastructure. New York, NY, USA: Association for Computing Machinery; 2015. pp. 1–8.

107. Stanzione D, West J, Evans RT, Minyard T, Ghattas O, Panda DK. Frontera: The Evolution of Leadership Computing at the National Science Foundation. Practice and Experience in Advanced Research Computing. New York, NY, USA: Association for Computing Machinery; 2020. pp. 106–111.

108. Fischer J, Tuecke S, Foster I, Stewart CA. Jetstream: A Distributed Cloud Infrastructure for Under Resourced Higher Education Communities. Proceedings of the 1st Workshop on The Science of Cyberinfrastructure: Research, Experience, Applications and Models. New York, NY, USA: ACM; 2015. pp. 53–61.

109. Stewart CA, Cockerill TM, Foster I, Hancock D, Merchant N, Skidmore E, et al. Jetstream: A self-provisoned, scalable science and engineering cloud environment. 2015 [cited 5 Jun 2018]. Available: `https://scholarworks.iu.edu/dspace/handle/2022/20286`

110. Pepple K. Deploying OpenStack. "O'Reilly Media, Inc."; 2011.

111. Sefraoui O, Aissaoui M, Eleuldj M. OpenStack: toward an open-source solution for cloud computing. Int J Comput Appl Technol. 2012;55:38–42.

112. Kumar R, Gupta N, Charu S, Jain K, Jangir SK. Open source solution for cloud computing platform using OpenStack. International Journal of Computer Science and Mobile Computing. 2014;3:89–98.

113. Brewer EA. Kubernetes and the path to cloud native. Proceedings of the Sixth ACM Symposium on Cloud Computing. New York, NY, USA: Association for Computing Machinery; 2015. p. 167.

114. Burns B, Beda J, Hightower K. Kubernetes: Up and Running: Dive into the Future of Infrastructure. "O'Reilly Media, Inc."; 2019.

115. Stubbs J, Cardone R, Packard M, Jamthe A, Padhy S, Terry S, et al. Tapis: An API Platform for Reproducible, Distributed Computational Research. Advances in Information and Communication. Springer International Publishing; 2021. pp. 878–900.

116. Rajasekar A, Moore R, Hou C-Y, Lee CA, Marciano R, de Torcy A, et al. iRODS Primer: Integrated Rule-Oriented Data System. Synthesis Lectures on Information Concepts, Retrieval, and Services. 2010;2:1–143.

117. Corral - Texas advanced computing center. [cited 13 Sep 2021]. Available: https://www.tacc.utexas.edu/systems/corral

118. Momjian B. PostgreSQL: Introduction and Concepts. [cited 6 Sep 2021]. Available: https://momjian.us/main/writings/pgsql/other/bookfigs.pdf

119. Obe RO, Hsu LS. PostgreSQL: Up and Running: A Practical Guide to the Advanced Open Source Database. "O'Reilly Media, Inc."; 2017.

120. Free and open search: The creators of Elasticsearch, ELK  Kibana. [cited 13 Sep 2021]. Available: https://www.elastic.co/

121. Aubry P, Mathieu V, Marchal J. ESUP-Portail: open source single sign-on with CAS (central authentication service). Proc of EUNIS04–IT Innovation in a Changing World. 2004;172–178.

122. Christie MA, Bhandar A, Nakandala S, Marru S, Abeysinghe E, Pamidighantam S, et al. Using Keycloak for Gateway Authentication and Authorization. 2017. doi:10.6084/m9.figshare.5483557.v1

123. Hardt D, Others. The OAuth 2.0 authorization framework. RFC 6749, October; 2012. Available: http://www.hjp.at/doc/rfc/rfc6749.html

124. Haak LL, Fenner M, Paglione L, Pentz E, Ratner H. ORCID: a system to uniquely identify researchers. Learn Publ. 2012;25:259–264.

125. Goddard M. The EU General Data Protection Regulation (GDPR): European Regulation that has a Global Impact. International Journal of Market Research. 2017;59:703–705.

126. Basney J, Fleury T, Gaynor J. CILogon: A federated X.509 certification authority for cyberinfrastructure logon. Concurr Comput. 2014;26:2225–2239.

127. Foster I, Kesselman C. Globus: a Metacomputing Infrastructure Toolkit. The International Journal of Supercomputer Applications and High Performance Computing. 1997;11:115–128.

128. Swagger. In: Swagger [Internet]. [cited 16 May 2021]. Available: https://swagger.io/

129. Biehl M. Swagger and OpenAPI 2.0: Powertools for RESTful API Design. CreateSpace Independent Publishing Platform; 2018.

130. Skidmore E, Kim S-J, Kuchimanchi S, Singaram S, Merchant N, Stanzione D. iPlant atmosphere: a gateway to cloud infrastructure for the plant sciences. Proceedings of the 2011 ACM workshop on Gateway computing environments. New York, NY, USA: Association for Computing Machinery; 2011. pp. 59–64.

131. K3s - Lightweight Kubernetes. [cited 12 Sep 2021]. Available: https://rancher.com/docs/k3s/latest/en/

132. Whitehead EJ, Wiggins M. WebDAV: IEFT standard for collaborative authoring on the Web. IEEE Internet Comput. 1998;2:34–40.

133. Surhone LM, Timpledon MT, Marseken SF. WebDAV: WebDAV, Hypertext Transfer Protocol, World Wide Web, Internet Engineering Task Force, Server (computing), Working Group, Operating System. Betascript Publishing; 2010.

134. Feryn T. Getting Started with Varnish Cache: Accelerate Your Web Applications. "O'Reilly Media, Inc."; 2017.

135. Sherry ST, Ward MH, Kholodov M, Baker J, Phan L, Smigielski EM, et al. dbSNP: the NCBI database of genetic variation. Nucleic Acids Res. 2001;29:308–311.

136. Geer LY, Marchler-Bauer A, Geer RC, Han L, He J, He S, et al. The NCBI BioSystems database. Nucleic Acids Res. 2010;38:D492–6.

137. Federhen S. The NCBI Taxonomy database. Nucleic Acids Res. 2012;40:D136–43.

138. Leinonen R, Sugawara H, Shumway M, Collaboration INSD. The sequence read archive. Nucleic Acids Res. 2010;39:D19–D21.

139. Kodama Y, Shumway M, Leinonen R, International Nucleotide Sequence Database Collaboration. The Sequence Read Archive: explosive growth of sequencing data. Nucleic Acids Res. 2012;40:D54–6.

140. Brase J. DataCite - A Global Registration Agency for Research Data. 2009 Fourth International Conference on Cooperation and Promotion of Information Resources in Science and Technology. ieeexplore.ieee.org; 2009. pp. 257–261.

141. Starr J, Gastl A. isCitedBy: A metadata scheme for DataCite. D-lib magazine: a monthly magazine about innovation and research in digital libraries. 2011;17. Available: `https://www.research-collection.ethz.ch/handle/20.500.11850/85769`

142. Oliver SL, Lenards AJ, Barthelson RA, Merchant N, McKay SJ. Using the iPlant collaborative discovery environment. Curr Protoc Bioinformatics. 2013;Chapter 1:Unit1.22.

143. Devisetty UK, Kennedy K, Sarando P, Merchant N, Lyons E. Bringing your tools to CyVerse Discovery Environment using Docker. F1000Res. 2016;5:1442.

144. Mount DW. Using the Basic Local Alignment Search Tool (BLAST). CSH Protoc. 2007;2007:db.top17.

145. Bowtie. [cited 12 Sep 2021]. Available: `http://bowtie-bio.sourceforge.net/index.shtml`

146. Granger B, Grout J. JupyterLab: Building blocks for interactive computing. Slides of presentation made at SciPy. 2016. Available: `http://archive.ipython.org/media/SciPy2016JupyterLab.pdf`

147. Martin J, Mannehed S, Astrand P, Ross S. novnc: Html5 vnc client. 2015.

148. Rescheneder P. ngmlr: NGMLR is a long-read mapper designed to align PacBio or Oxford Nanopore (standard and ultra-long) to a reference genome with a focus on reads that span structural variations. Github; Available: `https://github.com/philres/ngmlr`

149. InterPro. [cited 12 Sep 2021]. Available: `https://www.ebi.ac.uk/interpro/search/sequence/`

150. SPAdes – Center for Algorithmic Biotechnology. [cited 12 Sep 2021]. Available: `https://cab.spbu.ru/software/spades/`

151. Welcome to iVirus's documentation! — iVirus documentation. [cited 12 Sep 2021]. Available: https://ivirus.readthedocs.io/en/latest/

152. Cook J. Docker Hub. Docker for Data Science. Berkeley, CA: Apress; 2017. pp. 103–118.

153. Your packages, at home with their code. Github; Available: https://github.com/features/packages

154. Quay. [cited 12 Sep 2021]. Available: https://quay.io/

155. BioContainers Community including registry, documentation, specification. [cited 12 Sep 2021]. Available: https://biocontainers.pro/

156. GPU-Accelerated Innovation with NGC. [cited 12 Sep 2021]. Available: https://www.nvidia.com/en-us/gpu-cloud/

157. Harbor. [cited 12 Sep 2021]. Available: https://goharbor.io

158. Granger B, Pérez F. Jupyter: Thinking and storytelling with code and data. Authorea Preprints. 2021. Available: https://www.authorea.com/doi/full/10.22541/au.161298309.98344404

159. Boettiger C, Eddelbuettel D. An Introduction to Rocker: Docker Containers for R. arXiv [cs.SE]. 2017. Available: http://arxiv.org/abs/1710.03675

160. Nust D, Eddelbuettel D, Bennett D, Cannoodt R, Clark D, Daroczi G, et al. The Rockerverse: Packages and Applications for Containerisation with R. R J. 2020;12:437–461.

161. Kurtzer GM, Sochat V, Bauer MW. Singularity: Scientific containers for mobility of compute. PLoS One. 2017;12:e0177459.

162. CernVM File System. [cited 12 Sep 2021]. Available: https://cernvm.cern.ch/fs/

163. Features • GitHub Actions. Github; Available: https://github.com/features/actions

164. Wickham H. Mastering Shiny. "O'Reilly Media, Inc."; 2021.

165. Grinberg M. Flask Web Development: Developing Web Applications with Python. "O'Reilly Media, Inc."; 2018.

166. Gosling J, Joy B, Steele G, Bracha G. The Java Language Specification. Addison-Wesley Professional; 2000.

167. Tilkov S, Vinoski S. Node.js: Using JavaScript to Build High-Performance Network Programs. IEEE Internet Comput. 2010;14:80–83.

168. Loizides F, Schmidt B. Positioning and Power in Academic Publishing: Players, Agents and Agendas: Proceedings of the 20th International Conference on Electronic Publishing. IOS Press; 2016.

169. Soni R. Nginx: From beginner to pro. 1st ed. New York, NY: APRESS; 2016. Available: https://www.nginx.com/

170. Gasperskaja E, Kučinskas V. The most common technologies and tools for functional genome analysis. Acta medica Lituanica. 2017. pp. 1–11. doi:10.6001/actamedica.v24i1.3457

171. Gropp W, Lusk E, Doss N, Skjellum A. A high-performance, portable implementation of the MPI message passing interface standard. Parallel Comput. 1996;22:789–828.

172. Chandra R, Dagum L, Kohr D, Menon R, Maydan D, McDonald J. Parallel Programming in OpenMP. Morgan Kaufmann; 2001.

173. Wang L, Van Buren P, Ware D. Architecting a Distributed Bioinformatics Platform with iRODS and iPlant Agave API. 2015 International Conference on Computational Science and Computational Intelligence (CSCI). ieeexplore.ieee.org; 2015. pp. 420–423.

174. Dooley R, Brandt SR, Fonner J. The Agave Platform: An Open, Science-as-a-Service Platform for Digital Science. Proceedings of the Practice and Experience on Advanced Research Computing. New York, NY, USA: ACM; 2018. pp. 28:1–28:8.

175. Gannon D, Barga R, Sundaresan N. Cloud-Native Applications. IEEE Cloud Computing. 2017;4:16–21.

176. Fathoni H, Yang C-T, Chang C-H, Huang C-Y. Performance Comparison of Lightweight Kubernetes in Edge Devices. Pervasive Systems, Algorithms and Networks. Springer International Publishing; 2019. pp. 304–309.

177. Spjuth O, Capuccini M, Carone M, Larsson A, Schaal W, Novella JA, et al. Approaches for containerized scientific workflows in cloud environments with applications in life science. F1000Res. 2021;10:513.

178. Orzechowski M, Balis B, Pawlik K, Pawlik M, Malawski M. Transparent Deployment of Scientific Workflows across Clouds - Kubernetes Approach. 2018 IEEE/ACM International Conference on Utility and Cloud Computing Companion (UCC Companion). ieeexplore.ieee.org; 2018. pp. 9–10.

179. Sabharwal N, Pandey S, Pandey P. Getting Started with HashiCorp Terraform. In: Sabharwal N, Pandey S, Pandey P, editors. Infrastructure-as-Code Automation Using Terraform, Packer, Vault, Nomad and Consul: Hands-on Deployment, Configuration, and Best Practices. Berkeley, CA: Apress; 2021. pp. 11–45.

180. Tierney B, Kissel E, Swany M, Pouyoul E. Efficient data transfer protocols for big data. 2012 IEEE 8th International Conference on E-Science. ieeexplore.ieee.org; 2012. pp. 1–9.

181. Dart E, Rotman L, Tierney B, Hester M, Zurawski J. The Science DMZ. Proceedings of the International Conference on High Performance Computing, Networking, Storage and Analysis. New York, NY, USA: ACM; 2013. doi:10.1145/2503210.2503245

182. Mona M. Introduction to Amazon Cloud EC2 Overview. 2017. Available: `https://pdfs.semanticscholar.org/a9f2/8561ddb26b3c94393b165842f57555ebc24b.pdf`

183. AWS Snow Family. In: AWS Snow Family [Internet]. [cited 16 May 2021]. Available: `https://aws.amazon.com/snow/`

184. Hey T, Tansley S, Tolle KM, Others. The fourth paradigm: data-intensive scientific discovery. Microsoft research Redmond, WA; 2009.

185. Ahrens J, Hendrickson B, Long G, Miller S, Ross R, Williams D. Data-Intensive Science in the US DOE: Case Studies and Future Challenges. Computing in Science Engineering. 2011;13:14–24.

186. Critchlow T, van Dam KK. Data-Intensive Science. CRC Press; 2016.

187. About AWS. In: About AWS [Internet]. Sep 2011 [cited 16 May 2020]. Available: https://web.archive.org/web/20121005123855/http://aws.amazon.com/about-aws/

188. iPhone. In: iPhone Wikipedia [Internet]. [cited 16 May 2021]. Available: https://en.wikipedia.org/wiki/IPhone

189. Microsoft News Center. Microsoft cloud strength fuels fourth quarter results. 27 Jul 2021 [cited 9 Sep 2021]. Available: https://news.microsoft.com/2021/07/27/microsoft-cloud-strength-fuels-fourth-quarter-results-2/

190. Dobberstein L. AWS growing so fast its revenue makes it bigger than Cisco or HP. In: The Register [Internet]. 30 Jul 2021 [cited 9 Sep 2021]. Available: https://www.theregister.com/2021/07/30/amazon_q2_2021/

191. McKenzie L. Cost concerns keep cloud services out of reach of many small colleges. In: Inside Higher Ed — Higher Education News, Events and Jobs [Internet]. 4 Jul 2018 [cited 18 May 2023]. Available: https://www.insidehighered.com/news/2018/07/05/cost-concerns-keep-cloud-services-out-reach-many-small-colleges
