# Supplementary material for: CyVerse: Cyberinfrastructure for open science
Source: PLoS Comput Biol. 2024 Feb 7;20(2):e1011270. doi: 10.1371/journal.pcbi.1011270 (PMC10878509; doi:10.1371/journal.pcbi.1011270)
Supplement: S1 Table — Frequently used abbreviations and acronyms with descriptions. (PDF) [file pcbi.1011270.s002.pdf]

Table 1. Glossary. Frequently used abbreviations and acronyms with descriptions.

Acronym	Terminology	Description
ACCESS	Advanced Cyberinfrastructure Coordination Ecosystem: Services & Support	NSF new cyberinfrastructure framework, to replace XSEDE
AI	Artificial Intelligence	
AMQP	Advanced Message Queueing Protocol	Protocol used by HTCondor for jobs
API	Application Programming Interface	
ARIA	Accessible Rich Internet Applications	Framework for increasing accessibility of web services for the disabled
AVU	Attribute, Value, Unit	iRODS metadata organization framework
BSD	Berkeley Source Distribution	Open source software license
CARE	Collective benefit, Authority to control, Responsibility, Ethics	Data principles to be applied when working with indigenous data
CAS	Central Authentication Service	
CAT	CyVerse Austria	
CC BY	Creative Commons	Software license, the BY refers to the attribution which follows
CI/CD	Continuous Integration / Continuous Development	Framework for developing software, typically used by agile teams
CLI	Command Line Interface	
CSI	Container Storage Interface	standard for exposing arbitrary block and file storage systems to containerized workloads
CUAHSI	Consortium of Universities for the Advancement of Hydrologic Science, Inc	International hydrological services group
CMS	Content Management System	Software for managing projects and people
CVMFS	Cern Virtual File Management System	
CyVerse	Cyber Universe	Research cyberinfrastructure for life sciences
DBI	Directorate for Biological Infrastructure	NSF Directorate for life sciences
DE	Discovery Environment	CyVerse data science workbench
DevOps	Development and Operations	
DNS	Domain Name Service	

Continued on next page

Table 1 – continued from previous page

Acronym	Terminology	Description
DOI	Digital Object Identifier	Unique number assigned to a digital record, code, dataset, publication
EDI	Ecological Data Initiative	
EOT	Education Outreach Training	
EU	European Union	
FAIR	Findable Accessible Interoperable Reusable	Data principles for reproducible research
FOSS1	Free Open Source Software	
FOSS2	Foundational Open Science Skills	CyVerse workshop series
FUSE	Filesystem in USErspace	
G2P	Genome to Phenotype (Fields)	
GDPR	General Data Protection Regulation	European Union act protecting data and individuals
GNU	GNU's Not Unix! (recursive)	Open source software
GPL	General Public License	Open source software license
GPU	Graphical Processing Unit	
GUI	Graphic User Interface	Visual interface for a software program
HPC	High Performance Computing	Classical supercomputer centers
HTCondor		A derivation of HTC and how it scavenges (like a condor) for unused computing cycles
HTC	High Throughput Computing	Parallel computing
IaC	Infrastructure as Code	
IaaS	Infrastructure as a Service	
IDE	Integrated Development Environment	Applications with graphic interfaces for development, e.g., VS Code, RStudio, Jupyter
IoT	Internet of Things	
iRODS	Integrated Rule Oriented Data System	
ITAR	International Traffic in Arms Regulations	
K8S	Kubernetes	Open Source container orchestration technology released by Google
LDAP	Lightweight Directory Access Protocol	
LTAR	Long Term Ecological Research	
LTER	Long Term Agricultural Research	
ML	Machine Learning	
MPI	Message Passing Interface	Software for running analyses across multiple cpu nodes
NCBI	National Center for Biotechnology Information	
NCSA	National Center for Supercomputing Applications	

Continued on next page

Table 1 – continued from previous page

Acronym	Terminology	Description
NEON	National Ecological Observatory Network	
NPN	National Phenology Network	
NSF	National Science Foundation	Refers to the United States NSF
OAUTH	Open AUTHorization	
OpenMP	Open Multiprocessing	
ORCID	Open Researcher and Contributor IDentification	
OSG	Open Science Grid	High throughput grid computing
OSI	Open Source Initiative	
RLM	Reprise License Manager	
SaaS	Software as a Service	
SDSC	San Diego Supercomputing Center	
SDK	Software Development Kit	
SRA	Sequence Read Archive	
SSD	Solid State Drive	
sUAS	small Uncrewed Aerial Systems	
TACC	Texas Advanced Computing Center	
Tapis	TACC APIs	
TB (also kB, MB, GB, PB)	Terabyte (kilobyte, megabyte, gigabyte, petabyte)	
URL	Uniform Resource Locator	
VM	Virtual Machine	A virtual computer 'instance', typically hosted on cloud
XSEDE	eXtreme Science Engineering Discovery Environment	NSF-funded High Performance Compute resources for science, engineering, and humanities scholars
