# Supplementary material for: CyVerse: Cyberinfrastructure for open science
Source: PLoS Comput Biol. 2024 Feb 7;20(2):e1011270. doi: 10.1371/journal.pcbi.1011270 (PMC10878509; doi:10.1371/journal.pcbi.1011270)
Supplement: S2 Table — Public and private version control organizations on GitHub and GitLab for CyVerse Software, Public Container Registry, and Education. (PDF) [file pcbi.1011270.s003.pdf]

Table 2. Version Control. Public and private version control organizations on GitHub and GitLab for CyVerse Software, Public Container Registry, and Education.

Name	Address (URL)	Platform (languages & code)
Core Software	https://github.com/cyverse	SDK, API, Authorization, iRODS
Cloud-Native Services	https://gitlab.com/cyverse	K8s, Argo, Terraform, OpenAPI
VICE Container Registry	https://github.com/cyverse-vice	Docker, Jupyter, RStudio, VS Code
Discovery Environment	https://github.com/cyverse-de	Workbench (K8s, HTCondor, Go)
Learning Center	https://github.com/cyverse-learning-materials	ReadTheDocs, Markdown
