# Supplementary material for: CyVerse: Cyberinfrastructure for open science
Source: PLoS Comput Biol. 2024 Feb 7;20(2):e1011270. doi: 10.1371/journal.pcbi.1011270 (PMC10878509; doi:10.1371/journal.pcbi.1011270)
Supplement: S3 Table — Websites under the *.cyverse.org DNS address. (PDF) [file pcbi.1011270.s004.pdf]

Table 3. Interfaces. Web DNS under the *.cyverse.org address

Interfaces	Address (URL)	Role
Public Homepage	https://cyverse.org	Landing Page, News, Links
User Portal	https://user.cyverse.org	Enrollment, Features, Requests
Core Software Docs	https://docs.cyverse.org	DevOps Documentation
Learning Center	https://learning.cyverse.org	Education, Training, Documentation
DNA Subway	https://dnasubway.cyverse.org/	Education, Training
DataCommons	https://datacommons.cyverse.org	Data Metadata (with DOI)
WebDav	https://data.cyverse.org	Data Transfers (https:// protocol)
Discovery Environment	https://de.cyverse.org	Data Science Workbench
Atmosphere	https://atmo.cyverse.org	Cloud Service (Deprecated)
CACAO	https://cacao.cyverse.org	Cloud Automation & Orchestration
BisQue	https://bisque.cyverse.org	Images, Views, Annotations
Harbor	https://harbor.cyverse.org	Container Registry
KeyCloak	https://kc.cyverse.org	Authentication, Authorization
API	https://de.cyverse.org/...	Swagger API, Metadata
Internal Wiki	https://wiki.cyverse.org	Internal Documentation (Legacy & Deprecated)
