# Supplementary material for: CyVerse: Cyberinfrastructure for open science
Source: PLoS Comput Biol. 2024 Feb 7;20(2):e1011270. doi: 10.1371/journal.pcbi.1011270 (PMC10878509; doi:10.1371/journal.pcbi.1011270)
Supplement: S4 Table — External projects supported by CyVerse within the last 5 years. Resources include (Web) Hosting, Compute, Data Storage, Discovery Environment (DE), & API access. (PDF) [file pcbi.1011270.s005.pdf]

Table 4. Powered by. External projects supported by CyVerse within the last 5 years. Resources include (Web) Hosting, Compute, Data Storage, Discovery Environment (DE), & API access.

**Project Name**	**Resources Used**	**Web Services**
Astrolabe	Compute	http://astrolabe.arizona.edu/
Animal Genome	Hosting	
BetyDB	Compute	http://welsch.cyverse.org/
BioExtract Server	API	https://www.bioextract.org/
BioViz Connect	DE	https://www.bioviz.org/connect.html
BlazeGraph	Hosting	https://blazegraph.com/
BIEN	DataStore, API	https://bien.nceas.ucsb.edu/bien/, https://biendata.org/, https://tnrs.biendata.org/
CIPRES	DE (deprecated)	https://www.phylo.org/
ClearedLeaves DB	DataStore	http://www.clearedleavesdb.org/
CHEESEHEAD	DataStore, DE	https://data.eol.ucar.edu/project/CHEESEHEAD
CoGe	API, DataStore, Compute	https://genomevolution.org/coge/
DesignSafe	API	https://www.designsafe-ci.org/
DIRT	API, DataStore	http://dirt.cyverse.org/
Legume Fed	DataStore, DE	https://legumeinfo.org/
Galaxy	Compute	https://galaxyproject.org/
GenoPhenoEnvo	DataStore, Compute	
Genomes to Fields	API, DataStore	https://www.genomes2fields.org/
Gramene	Compute	https://www.gramene.org/
iMicrobe	API, DataStore, Compute	https://www.imicrobe.us/
iVirus	API, DataStore	https://www.ivirus.us/
Integrated Breeding Platform	API	https://www.integratedbreeding.net/
BioViz	DataStore, API	https://www.bioviz.org/
iPToL Big Trees	Hosting, Data Store	
DNA Subway	API, DataStore, Compute	https://dnasubway.cyverse.org/
Mineral Nutrition	Compute	
Pathway Tools	Hosting	
PEcAN	DataStore, Compute	https://pecanproject.github.io/
PhyloTNRS	Hosting	https://www.evoio.org/wiki/Phylotastic/TNRS
PlantIT	DataStore, API, Compute	https://plantit.cyverse.org/
Plant Reactome	DataStore, Compute	https://plantreactome.gramene.org/
Planteome	Compute (mirror)	http://oxford.cyverse.org/, http://draco.cyverse.org/amigo
Plastome Database	Hosting, Data Store	
Promzea	Hosting	http://www.promzea.org/
SciApps	API, DataStore	https://www.sciapps.org/
SoyKB	Compute, DataStore	https://soykb.org
SD2E	API	https://sd2e.org
Symbiodinium	Hosting	
TERRA-REF	Compute	https://terraref.org/
TNRS	Compute	https://tnrs.biendata.org/
TARGeT	Hosting	http://target.iplantcollaborative.org/
