# Supplementary material for: CyVerse: Cyberinfrastructure for open science
Source: PLoS Comput Biol. 2024 Feb 7;20(2):e1011270. doi: 10.1371/journal.pcbi.1011270 (PMC10878509; doi:10.1371/journal.pcbi.1011270)
Supplement: S5 Table — On-premises resources maintained by CyVerse at the University of Arizona. DE = Discovery Environment, VICE = Virtual Interactive Compute Environment. (PDF) [file pcbi.1011270.s006.pdf]

Table 5. University of Arizona Hardware. On-premises resources maintained by CyVerse at the University of Arizona. DE = Discovery Environment, VICE = Virtual Interactive Compute Environment.

**Platform**	**Nodes**	**Cores**	**RAM**	**Storage (Types)**	**GPUs**
DE VICE Kubernetes Cluster	18	1052	7.68 TB	191 TB Local	21
DE Condor Cluster	24	612	5.2 TB	142 TB Local	-
OpenStack Cloud	69	1192	16.75 TB	203.6 TB Local, 428 TB Ceph	-
Virtualizations (Xen)	11	224	2.8 TB	57 TB NFS	-
Data Store (iRODS)	38	680	10 TB	8.18 PB	-
