# Supplementary material for: CyVerse: Cyberinfrastructure for open science
Source: PLoS Comput Biol. 2024 Feb 7;20(2):e1011270. doi: 10.1371/journal.pcbi.1011270 (PMC10878509; doi:10.1371/journal.pcbi.1011270)
Supplement: S6 Table — Duration of seconds within CyVerse, as well as from (download) and to (upload) other research HPC (TACC), cloud (XSEDE Jetstream2) and commercial cloud services (AWS, Google Cloud). *Transfer duration represents a rounded number of seconds as a geometric mean of n = 30 runs. (PDF) [file pcbi.1011270.s007.pdf]

Table 6. Benchmarking. Data Store transfer duration in seconds within CyVerse, as well as directly from (download) and uploads to other research HPC (TACC), cloud (ACCESS-CI Jetstream2) and commercial cloud services (AWS, Google Cloud) from CyVerse. Transfer duration represents the rounded number of seconds as a geometric μ mean of $n = 30$ runs.

**File Size**	**Platform**				
	**CyVerse**	**TACC**	**Jetstream-2 (IU)**	**AWS**	**Google Cloud**
**Download (seconds)**					
10 kiB	1	5	2	2	2
10 MiB	1	5	3	4	4
10 GiB	26	55	19	31	28
**Upload (seconds)**					
10 kiB	1	5	2	2	2
10 MiB	2	7	3	3	3
10 GiB	40	76	49	61	55
