# Supplementary material for: CyVerse: Cyberinfrastructure for open science
Source: PLoS Comput Biol. 2024 Feb 7;20(2):e1011270. doi: 10.1371/journal.pcbi.1011270 (PMC10878509; doi:10.1371/journal.pcbi.1011270)
Supplement: S7 Table — How they run, where they run, and popular applications. (PDF) [file pcbi.1011270.s008.pdf]

Table 7. DE Applications. How they run, where they run, and popular applications.

Type	Scheduler	Infrastructure	Workflows	Applications
Executable	HTCondor	CyVerse	Yes	BLAST [?], BowTie [?], Python, R
Interactive	Kubernetes	CyVerse	No	Jupyter [?], RStudio [?], noVNC [?]
High Throughput	HTCondor	OSG	Yes	NGMLR [?]
High Performance	TAPIS	TACC	Yes	InterProScan [?], SPAdes [?], iVirus tools [?]
