# Supplementary material for: CyVerse: Cyberinfrastructure for open science
Source: PLoS Comput Biol. 2024 Feb 7;20(2):e1011270. doi: 10.1371/journal.pcbi.1011270 (PMC10878509; doi:10.1371/journal.pcbi.1011270)
Supplement: S2 File — CyVerse’s services versus other public research and commercial cyberinfrastructure. Some services offered by commercial cloud have free tiers as well as paid subscriptions. (Also see link to table). (PDF) [file pcbi.1011270.s010.pdf]

DISCLAIMER: This table is presented as-is without any gauarantee of accuracy, readers are welcome to submit suggestions, changes, or additions to the list. **CCBY0 LICENSE**

Platform	Data				Users			Orchestration				Computing Resources				Containers		Access			
	Storage	Sharing	DOI	Logs	Logs	Auth	Support	Dev Ops	K8s	HT Condor	Terraform	Open Stack Cloud	Commercial Cloud	HPC	HTC	Docker	Hosted Registry	Free Tier	Allocation by Request	Subscription	Managed Cloud Billing
CyVerse	✓	✓	✓	✓	✓	✓	✓	✓	✓	✓	✓	✓	✓	✓	✓	✓	✓	✓	✓	✓	✓
Galaxy	✓	✓	✓			✓						✓		✓		✓		✓	✓		
GLOBUS		✓		✓	✓	✓	✓	✓										✓		✓	
DNA Nexus	✓	✓				✓							✓			✓				✓	
KBase	✓	✓				✓			✓					✓		✓	✓	✓			
Gen3	✓	✓				✓	✓	✓	✓			✓	✓							✓	✓
Terra.bio	✓	✓				✓	✓						✓			✓				✓	✓
WholeTale	✓	✓	✓	✓		✓										✓		✓			
National Research Platform	✓	✓				✓			✓	✓		✓		✓	✓	✓		✓	✓		
Pacific RP								✓	✓	✓				✓	✓	✓					
Data8 edX						✓	✓		✓				✓			✓		✓	✓		
CyberGIS	✓					✓			✓					✓		✓		✓	✓		
HydroShare	✓	✓				✓			✓							✓		✓			
DesignSafe	✓	✓	✓			✓												✓			
NDS Labs	✓	✓				✓			✓			✓		✓		✓		✓			
Chameleon																					
CloudLab																					
JetStream-2	✓	✓				✓	✓	✓	✓			✓				✓		✓	✓		
NIH Strides													✓								✓
CloudBank						✓	✓		✓				✓								✓
PATh	✓					✓				✓					✓				✓		
OSG						✓			✓	✓					✓		✓	✓	✓		
Posit (RStudio)	✓	✓		✓	✓	✓	✓						✓							✓	✓
GEE	✓	✓			✓	✓							✓					✓			✓
Microsoft PC	✓	✓		✓		✓							✓					✓			✓
PanGeo									✓			✓	✓			✓			✓		✓
OSF.io	✓					✓															✓
2i2c							✓	✓					✓			✓					✓
ICICLE									✓					✓		✓					✓
7 Bridges	✓	✓				✓	✓						✓	✓						✓	✓
GEMs																					
MyBinder.org		✓											✓			✓		✓			
DigitalOcean	✓	✓	✓			✓							✓							✓	✓
Google Colab	✓	✓				✓							✓							✓	✓
GitHub Codespaces	✓	✓				✓							✓			✓				✓	✓
GitPod	✓	✓				✓		✓	✓				✓			✓				✓	✓
Need to be updated:																					
D4Science																					
SMCE																					
Euro Data Cube																					
Sentinel-Hub																					
OpenEO Cloud																					
Open Data Cube																					
Climate Tool Box																					
Sci MAAP																					
Copernicus Climate Toolbox																					
InvenioDRM																					
AboVE																					
