# Supplementary material for: CyVerse: Cyberinfrastructure for open science
Source: PLoS Comput Biol. 2024 Feb 7;20(2):e1011270. doi: 10.1371/journal.pcbi.1011270 (PMC10878509; doi:10.1371/journal.pcbi.1011270)
Supplement: S1 File — Peer-reviewed research citing the use of resources from the iPlant Collaborative (2008–2017) and CyVerse (2017-Present). Also see https://cyverse.org/publications for the latest update. (PDF) [file pcbi.1011270.s009.pdf]

Key	Item Type	Publication Year	Author	Title	Publication Title	ISBN	ISSN	DOI	Url	Abstract Note	Date	Date Added	Date Modified	Access Date	Pages	Num Pages	Issue	Volume	Number Of Volur	Journal Abbrevia	Short Title	Series	Series Number	Series Text	Series Title	Publisher	Place	Language	Rights	Type
IKR2DU5S	bookSection	2022	Fitzgerald, Caleb	Using the Automa	Plant Gravitropis	978-1-07-161676-5 978-1-07-161677-2			https://link.springer.com/10.1007/978-1-0716-1677-2_6		2022	2022-02-24 21:4	2022-02-24 21:4	2022-02-24 21:4	81-94			2368								Springer US	New York, NY	en		
T79KGTJH	journalArticle	2022	Nguyen, Duong T	The genetics of v	Theoretical and Applied Genetics		0040-5752, 1432	10.1007/s00122-	https://link.springer.com/10.1007/s00122-021-03954-4		2022-01	2022-02-24 21:5	2022-02-25 1:01	2022-02-24 21:5	107-124		1	135		Theor Appl Gene	The genetics of vigour-related traits in chickpea (Cicer arietinum L.)							en		
KPNMQKC8	journalArticle	2022	Zhang, Xiao; Zhu	Genome-wide an	Theoretical and Applied Genetics		0040-5752, 1432	10.1007/s00122-	https://link.springer.com/10.1007/s00122-021-03965-1		2022-01	2022-02-24 21:5	2022-02-25 1:01	2022-02-24 21:5	273-290		1	135		Theor Appl Genet								en		
XUVZR3TN	bookSection	2022	Adams, Sally; Ca	Chromatin Immu	Plant Circadian N	978-1-07-161911-7 978-1-07-161912-4			https://link.springer.com/10.1007/978-1-0716-1912-4_12		2022-10-22	2022-02-24 22:0	2022-02-24 22:0	2022-02-24 22:0	135-150			2398								Springer US	New York, NY	en		
TDW8H2XH	journalArticle	2022	Amos, Beatrice; A	VEuPathDB: the	Nucleic Acids Research		0305-1048, 1362	10.1093/nar/gkab	https://academic.	Abstract The Eukaryotic Pathogen, Vector and Host Informatics Resource (VEuPathDB, https://veupathdb.o	2022-01-07	2022-02-25 1:21	2022-02-25 1:21	2022-02-25 1:21	D898-D911		D1	50			VEuPathDB							en		
BJCTJR9N	journalArticle	2022	Satapathy, Kaush	The Variability of	The Astrophysical Journal						2022	2022-02-25 1:31	2022-02-25 1:31		13		1	925												
2FE8M3MR	journalArticle	2022	Chiozzi, Giorgio;	Insular gazelles c	Mammalian Biology		1616-5047, 1618	10.1007/s42991-	https://link.springer.com/10.1007/s42991-021-00186-3		2022-02	2022-02-25 1:56	2022-02-25 1:56	2022-02-25 1:56	1-20		1	102		Mamm Biol	Insular gazelles of the circum-Arabian seas							en		
LRGZWW5C	journalArticle	2022	Morisse, Merlijn;	A European pers	Field Crops Research		3784290	10.1016/j.fcr.202	https://linkinghub.elsevier.com/retrieve/pii/S0378429021003178		2022-02	2022-02-25 2:18	2022-02-25 2:18	2022-02-25 2:18	108371			276		Field Crops Research								en		
86ER4UF3	journalArticle	2022	Zhao, Yating; Zha	Optimizing ear-pl	Field Crops Research		3784290	10.1016/j.fcr.202	https://linkinghub.elsevier.com/retrieve/pii/S0378429021003221		2022-02	2022-02-25 2:21	2022-02-25 2:21	2022-02-25 2:21	108376			276		Field Crops Research								en		
38YVJKIS	journalArticle	2022	Martinson, John V	De Novo Assemb	Environmental Toxicology and Che		0730-7268, 1552	10.1002/etc.5266	https://onlinelibrary.wiley.com/doi/10.1002/etc.5266		2022-02	2022-02-25 2:39	2022-02-25 2:39	2022-02-25 2:39	448-461		2	41		Enviro Toxic and Chemistry								en		
P4QDT3UK	journalArticle	2022	Gupta, Shreya; R	Innovations in Cr	Plant Abiotic Stress Physiology: Volume 1: Responses and Adaptations						2022	2022-02-25 2:59	2022-02-25 2:59		1															
ZUEF8EE8	journalArticle	2022	Sams, Eleanor I.	From karyotypes	Human Genetics and Genomics Ad		26662477	10.1016/j.xhgg.2	https://linkinghub.elsevier.com/retrieve/pii/S2666247721000622		2022-01	2022-02-25 3:04	2022-02-25 3:04	2022-02-25 3:04	100081		1	3		Human Genetics and Genomics Advances								en		
D6UAGM3J	journalArticle	2022	Tuggle, Christoph	The Agricultural G	Genome Biology		1474-760X	10.1186/s13059-	https://genomebiology.biomedcentral.com/articles/10.1186/s13059-021-02570-1		2022-12	2022-02-25 3:16	2022-02-25 3:16	2022-02-25 3:16	3, s13059-021-02570-1		1	23		Genome Biol	The Agricultural Genome to Phenome Initiative (AG2PI)							en		
L6XJDCSF	journalArticle	2022	Arras, Philipp; Fr	Variable structure	Nature Astronomy		2397-3366	10.1038/s41550-	https://www.natu	Abstract The immediate vicinity of an active supermassive black hole—with its event horizon, phot	2022-02	2022-02-25 3:18	2022-02-25 3:18	2022-02-25 3:18	259-269		2	6		Nat Astron								en		
DKB4PK52	journalArticle	2022	Shang, Jiayu; Su	CHERRY: a Com	arXiv:2201.01018 [cs, q-bio]				http://arxiv.org/ab	Prokaryotic viruses, which infect bacteria and archaea, are key players in microbial communities. Predicting the ho	2022-01-04	2022-02-25 3:19	2022-02-25 3:19	2022-02-25 3:19							CHERRY									
YGVAUZ2V	journalArticle	2022	Senn, Savanah;	Metagenomic Ins	BioTech		2673-6284	10.3390/biotech1	https://www.mdpi	The purpose of this paper is to elucidate the roles that microbes may be playing in the rootzone of the medicinal pla	2022-01-14	2022-02-25 3:21	2022-02-25 3:21	2022-02-25 3:21	1		1	11		BioTech								en		
QMRVCIGM	journalArticle	2022	Spalding, E.; Mor	High-contrast Ima	The Astronomical Journal		0004-6256, 1538	10.3847/1538-38	https://iopscience	Abstract The Large Binocular Telescope (LBT) has two 8.4 m primary mirrors that produce beams	2022-02-01	2022-02-25 3:22	2022-02-25 3:22	2022-02-25 3:22	62		2	163		AJ	High-contrast Imaging with Fizeau Interferometry									
GSW5DT5B	journalArticle	2022	Heilman, Kelly A.	Ecological foreca	Global Change Biology		1354-1013, 1365	10.1111/gcb.1603	https://onlinelibrary.wiley.com/doi/10.1111/gcb.16038		2022-01-13	2022-02-25 3:23	2022-02-25 3:23	2022-02-25 3:23	gcb.16038					Global Change B	Ecological forecasting of tree growth							en		
JDULYZD4	bookSection	2022	Wang, Liya; Lu, Z	SciApps: An Autc	Plant Bioinformat	978-1-07-162066-3 978-1-07-162067-0			https://link.springer.com/10.1007/978-1-0716-2067-0_10		2022	2022-02-25 3:39	2022-02-25 3:39	2022-02-25 3:39	197-209			2443			SciApps					Springer US	New York, NY	en		
X766VTH8	bookSection	2022	Williams, Jason	CyVerse for Repr	Plant Bioinformat	978-1-07-162066-3 978-1-07-162067-0			https://link.spring	Abstract Posing complex research questions poses complex reproducibility challenges. Datasets may need	2022	2022-02-25 3:40	2022-02-25 3:40	2022-02-25 3:40	57-79			2443			CyVerse for Reproducible Research					Springer US	New York, NY	en		
QW35VIU3	journalArticle	2022	Amundson, Kaela	Microbial coloniz	Microbiome		2049-2618	10.1186/s40168-	https://microbiom	Abstract Background Microbial colonization of subsurface shales following hydraulic fract	2022-12	2022-02-25 3:41	2022-02-25 3:41	2022-02-25 3:41	5		1	10		Microbiome								en		
FHKXL5NP	journalArticle	2022	Dykes, Jeff; Kapp	Using DNA Barco	The American Biology Teacher		0002-7685, 1938	10.1525/abt.2022	https://online.ucp	In 2019 the Biology 100 class of Wenatchee Valley College at Omak (WVCO) worked on a DNA barcoding project	2022-01-01	2022-02-25 3:44	2022-02-25 3:44	2022-02-25 3:44	40-44		1	84										en		
ZKPE8AM3	journalArticle	2022	Tran, Hoang; Zha	A hydrological sir	Scientific Data		2052-4463	10.1038/s41597-	https://www.natu	Abstract This article presents a hydrological reconstruction of the Upper Colorado River Basin with an hourl	2022-12	2022-02-25 3:44	2022-02-25 3:44	2022-02-25 3:44	16		1	9		Sci Data								en		
QDVHLU4Z	journalArticle	2022	Chávez Montes,	A comparative ge	Proceedings of the National Acade		0027-8424, 1091	10.1073/pnas.21	http://www.pnas.	Desiccation tolerance is an ancient and complex trait that spans all major lineages of life on earth. Although importa	2022-02-01	2022-02-25 3:48	2022-02-25 3:48	2022-02-25 3:48	e2118886119		5	119		Proc Natl Acad Sci USA								en		
6GQQPP39	journalArticle	2022	Boeckman, Justi	Sheep in wolves'	Virology		426822	10.1016/j.virol.20	https://linkinghub.elsevier.com/retrieve/pii/S0042682222000204		2022-03	2022-02-25 3:49	2022-02-25 3:49	2022-02-25 3:49	86-100			568		Virology	Sheep in wolves' clothing							en		
6PVDLWHZ	journalArticle	2022	Jhu, Min-Yao; Fa	Heinz-resistant tc	Plant Physiology		1532-2548	10.1093/plphys/k	https://academic.	Abstract Cuscuta species (dodders) are agriculturally destructive, parasitic angiosperms. These parasitic pl	2022-01-31	2022-02-25 3:52	2022-02-25 3:52	2022-02-25 3:52	kiac024													en		
5VP8HPWN	journalArticle	2022	Fernandez-Pozo,	An overview of bi	Journal of Experimental Botany		0022-0957, 1460	10.1093/jxb/erac	https://academic.	Abstract Bryophytes are useful models for the study of plant evolution, development, plant-fungal symbiosis	2022-02-11	2022-02-25 3:58	2022-02-25 3:58	2022-02-25 3:58	erac052													en		
M2KJ7TCA	report	2022	Xu, Gen; Lyu, Jin	A historically balanced locus under recent directional selection in responding to change					http://biorxiv.org/l	ABSTRACT Understanding the patterns of selection during plant evolution and recent crop improvement pro	2022-02-10	2022-02-25 3:59	2022-02-25 3:59	2022-02-25 3:59												Genomics		en		preprint
VSCSQ9KG	report	2022	Rhoades, Nichola	Functional, transcriptional, and microbial shifts associated with healthy pulmonary aging					http://biorxiv.org/l	ABSTRACT Older individuals are at increased risk of developing severe respiratory infections due to	2022-02-10	2022-02-25 3:59	2022-02-25 3:59	2022-02-25 3:59							Functional, transcriptional, and microbial shifts associated with healthy pulmonary aging					Immunology		en		preprint
9TLHGYQ7	thesis	2022	Palos, Kyle	Identifying Long Noncoding RNAs and Examining Their Functional Roles in Brassicace					https://ezproxy.lib	Tremendous progress has been made over the last century characterizing the structure, function, and evolutionary	2022	2022-02-25 4:01	2022-02-25 4:01			147												English		PhD Thesis
P2CC56PG	bookSection	2022	Wyman, Christina	The Model Legur	The Medicago tru	978-3-030-90756-3 978-3-030-90757-0			https://link.springer.com/10.1007/978-3-030-90757-0_1		2022	2022-02-25 4:03	2022-02-25 4:03	2022-02-25 4:03	1-9						The Model Legume, Medicago truncatula in the Genomic Era					Springer Internat	Cham	en		
KULYRYLR	report	2022	Martínez-Alvarez	With a pinch of salt: metagenomic insights into Namib Desert salt pan microbial mats ar					http://biorxiv.org/l	Abstract Playas, saline-rich springs surrounded by halite evaporates (also called salt pans) have pla	2022-02-20	2022-03-02 16:5	2022-03-02 16:5	2022-03-02 16:5							With a pinch of salt					Microbiology		en		preprint
6XJPEQ4T	journalArticle	2021	Du, Jingjie; Zaye	Spinal Cord Injur	mSystems		2379-5077	10.1128/mSyster	https://journals.a	To our knowledge, this is the first article to apply metagenomics to characterize changes in gut microbial population	2021-06-29	2021-09-07 13:5	2021-09-07 13:5	2021-09-07 13:5			3	6		mSystems								en		
49EWSH9B	journalArticle	2021	Gustafsson, Lena	Burn severity anc	Journal of Vegetation Science		1100-9233, 1654	10.1111/jvs.1296	https://onlinelibrary.wiley.com/doi/10.1111/jvs.12966		2021-01	2021-09-07 13:5	2021-12-07 20:1	2021-09-07 13:5			1	32		J Veg Sci								en		
LMUFG3ZU	journalArticle	2021	Hua, Xia; Berkow	Global Analysis c	Non-Coding RNA		2311-553X	10.3390/ncrna70	https://www.mdpi	RNA silencing pathways control eukaryotic gene expression transcriptionally or posttranscriptionally in a sequence-	2021-04-27	2021-09-07 13:5	2021-10-14 0:24	2021-09-07 13:5	28		2	7		ncRNA								en		
8Q5TV59T	journalArticle	2021	Kranawetter, Clay	A Medicago trunc	Metabolites		2218-1989	10.3390/metabo	https://www.mdpi	Plant roots are composed of many differentiated tissue types, with each tissue exhibiting differential quantitative an	2021-04-13	2021-09-07 13:5	2021-10-14 0:22	2021-09-07 13:5	238		4	11		Metabolites								en		
GF52F4BJ	journalArticle	2021	Swetnam, Tyson	On the Use of Sta	Remote Sensing		2072-4292	10.3390/rs13081	https://www.mdpi	In this work we explore three methods for quantifying ecosystem vegetation responses spatially and temporally usi	2021-04-08	2021-09-07 13:5	2022-02-25 0:41	2021-09-07 13:5	1448		8	13		Remote Sensing								en		
2IWTH2BX	journalArticle	2021	Werbin, Zoey R.;	The National Ecc	F1000Research		2046-1402	10.12688/f1000re	https://f1000rese	The National Ecological Observatory Network (NEON) annually performs shotgun metagenomic sequencing to san	2021-04-19	2021-09-07 13:5	2022-02-25 2:35	2021-09-07 13:5	299			10		F1000Res	The National Ecological Observatory Network's soil metagenomes							en		
HL8NTEAT	journalArticle	2021	Wu, Di; Tanaka, I	High-resolution g	G3 Genes|Genomes|Genetics		2160-1836	10.1093/g3journa	https://academic.	Abstract Despite its importance to plant function and human health, the genetics underpinning element leve	2021-04-15	2021-09-07 13:5	2021-10-14 0:24	2021-09-07 13:5	jkab059		4	11										en		
ACCI4R9E	journalArticle	2021	Thessen, Anne E	From Reductionis	PLOS Biology		1545-7885	10.1371/journal.p	https://dx.plos.or	Decades of reductionist approaches in biology have achieved spectacular progress, but the proliferation of subdisc	2021-03-26	2021-09-07 13:5	2021-12-03 18:4	2021-09-07 13:5	e3001129		3	19		PLoS Biol	From Reductionism to Reintegration							en		
AI9UEMYL	journalArticle	2021	Butina, Tatyana V	Extended Evalua	Microorganisms		2076-2607	10.3390/microorg	https://www.mdpi	Lake Baikal is a unique oligotrophic freshwater lake with unusually cold conditions and amazing biological diversity	2021-04-05	2021-09-07 13:5	2021-10-14 0:16	2021-09-07 13:5	760		4	9		Microorganisms								en		
77LI52UW	journalArticle	2021	Campbell, Malac	Translating insigh	Genetics		1943-2631	10.1093/genetics	https://academic.	Abstract Oat (Avena sativa L.) seed is a rich resource of beneficial lipids, soluble fiber, protein, and antioxid	2021-03-31	2021-09-07 13:5	2021-10-14 0:16	2021-09-07 13:5	iyaa043		3	217										en		
I89S8ARG	book	2021	Peterson, April Li	Evolution and Sexual Dimorphism in Meiotic Recombination Rates in Murids							2021	2021-09-07 14:0	2021-10-14 0:23													The University of Wisconsin-Madison				
BJH4Z6DN	journalArticle	2021	Natarajan, Priyar	QuasarNet: A nev	arXiv:2103.13932 [astro-ph]				http://arxiv.org/ab	We present Quasarnet, a novel research platform that enables deployment of data-driven modeling techniques for	2021-03-25	2021-09-07 14:0	2021-10-14 0:25	2021-09-07 14:0							QuasarNet									
U3C6EBKD	journalArticle	2021	Freewoman, Julia	Temporal gene re	Cell Cycle		1538-4101, 1551	10.1080/153841(	https://www.tandfonline.com/doi/full/10.1080/15384101.2021.1904554		2021-04-18	2021-09-07 14:0	2021-10-14 0:18	2021-09-07 14:0	792-807		8	20		Cell Cycle								en		
P2VL48EY	journalArticle	2021	Liu, Zhi-Fang; Ma	Can plastid geno	Botanical Journal of the Linnean S		0024-4074, 1095	10.1093/botlinne	https://academic.	Abstract Using DNA barcoding for species identification remains challenging for many plant groups. New se	2021-08-10	2021-09-07 14:0	2021-10-14 0:23	2021-09-07 14:0	1-14		1	197										en		
2HTWEZL2	journalArticle	2021	Barker, Richard;	Transcriptome: A	Higher Plants, Algae and Cyanobacteria in Space Environments						2021	2021-09-07 14:0	2021-09-07 14:0																	
VANNYVX8	journalArticle	2021	Salgado-Salazar,	Comparative gen	G3 Genes|Genomes|Genetics		2160-1836	10.1007/978-1-6	https://academic.	Abstract Neonectria faginata and Neonectria coccinea are the causal agents of the insect-fungus disease c	2021-04-15	2021-09-07 14:0	2022-03-03 23:3	2021-09-07 14:0	jkab071		4	11										en		
RK8TAS9X	journalArticle	2021	Holmfeldt, Karin;	The Fennoscand	Communications Biology		2399-3642	10.1038/s42003-	http://www.nature	Abstract The deep biosphere contains members from all three domains of life along with viruses. Here we i	2021-12	2021-09-07 14:0	2021-12-03 18:4	2021-09-07 14:0	307		1	4		Commun Biol								en		
BI4UPJRC	journalArticle	2021	Eshraghi, Mehdi;	Mutant Huntingtin	Nature Communications		2041-1723	10.1038/s41467-	http://www.nature	Abstract The polyglutamine expansion of huntingtin (mHTT) causes Huntington disease (HD) and neurodeg	2021-12	2021-09-07 14:0	2021-12-03 18:4	2021-09-07 14:0	1461		1	12		Nat Commun								en		
2QBLE4CW	journalArticle	2021	Amfar, Fadhliah;	Molecular identifi	Biodiversitas Journal of Biological Diversity						2021	2021-09-07 14:0	2021-10-14 0:16				3	22												
JPYJE4YX	report	2021	Cheng, Zizhang;	Analysis of Classic Tomato Mutants Reveals Influence of Leaf Vein Density on Fruit BRI					http://biorxiv.org/l	ABSTRACT Tomato bipinnate (bip) is a classic leaf mutant, with high	2021-03-01	2021-09-07 14:0	2021-10-14 0:17	2021-09-07 14:0												Plant Biology		en		preprint
R7D5XATD	report	2021	Martinson, John;	De novo assembly and annotation of a highly contiguous reference genome of the fathe					http://biorxiv.org/l	ABSTRACT The Fathead Minnow (FHM) is one of the most important and widely used model organi	2021-02-25	2021-09-07 14:0	2021-10-14 0:23	2021-09-07 14:0												Genomics		en		preprint
DYZDVUKQ	journalArticle	2021	Barbosa, Rodrigc	Key species sele	Ecological Engineering		9258574	10.1016/j.ecoleng	https://linkinghub.elsevier.com/retrieve/pii/S0925857421000446		2021-04	2021-09-07 14:0	2021-12-07 20:1	2021-09-07 14:0	106190			162		Ecological Engineering								en		
USSKNTV6	journalArticle	2021	Lee, Jun-Yeong;	Conserved dual-r	Nucleic Acids Research		0305-1048, 1362	10.1093/nar/gkab	https://academic.	Abstract Recent genomic data analyses have revealed important underlying logics in eukaryotic gene regul	2021-03-18	2021-09-07 14:0	2021-10-14 0:23	2021-09-07 14:0	2583-2597		5	49										en		
DKWWWDSM	journalArticle	2021	Hu, Ying; Colantc	Genome assemb	Nature Communications		2041-1723	10.1038/s41467-	http://www.nature	Abstract Sweet corn is one of the most important vegetables in the United States and Canada. He	2021-12	2021-09-07 14:0	2021-12-03 18:4	2021-09-07 14:0	1227		1	12		Nat Commun								en		
LD6CKPBE	journalArticle	2021	Harrison, Kyle; M	Lasting conseque	BMC Plant Biology		1471-2229	10.1186/s12870-	https://bmcplantb	Abstract Background The tomato psyllid, Bactericera cockerelli	2021-12	2021-09-07 14:0	2021-12-03 18:4	2021-09-07 14:0	114		1	21		BMC Plant Biol								en		
2LANJB88	report	2021	Baseggio, Mathe	Genome-wide association study suggests an independent genetic basis of zinc and cac					http://biorxiv.org/l	ABSTRACT Despite being one of the most consumed vegetables in the United States, the elementa	2021-02-20	2021-09-07 14:0	2021-10-14 0:20	2021-09-07 14:0												Genetics		en		preprint
4SJ23Q88	journalArticle	2021	Brandies, Parice	Ten simple rules	PLOS Computational Biology		1553-7358	10.1371/journal.p	https://dx.plos.org/10.1371/journal.pcbi.1008645		2021-02-18	2021-09-07 14:0	2021-12-07 20:1	2021-09-07 14:0	e1008645		2	17		PLoS Comput Biol								en		
IZ7SVCCZ	journalArticle	2021	Barakhshan, Par	Exchanging Best	arXiv:2102.09373 [cs]				http://arxiv.org/ab	We present best practices and tools for professionals who support computational and data intensive (CDI) research	2021-02-09	2021-09-07 14:0	2021-10-14 0:16	2021-09-07 14:0																
8HELASWN	journalArticle	2021	Moyer, Tessa B.;	Multiple Classes	Journal of Natural Products		0163-3864, 1520	10.1021/acs.jnat	https://pubs.acs.org/doi/10.1021/acs.jnatprod.0c01203		2021-02-26	2021-09-07 14:0	2021-10-14 0:25	2021-09-07 14:0	444-452		2	84		J. Nat. Prod.								en		
6ECYEEAJ	journalArticle	2021	Davami, Fatemeh	Fog-based archit	Computing		0010-485X, 1436	10.1007/s00607-	http://link.springer.com/10.1007/s00607-021-00905-1		2021-02-17	2021-09-07 14:0	2021-10-14 0:17	2021-09-07 14:0						Computing								en		
PUUEVMEW	journalArticle	2021	Taş, Neslihan; de	Metagenomic toc	Current Opinion in Biotechnology		9581669	10.1016/j.copbio.	https://linkinghub.elsevier.com/retrieve/pii/S0958166921000240		2021-02	2021-09-07 14:0	2021-12-07 20:1	2021-09-07 14:0	184-191			67		Current Opinion in Biotechnology								en		
ME752TXA	journalArticle	2021	Bearss, Jeremiah	EDC3 phosphory	EMBO reports		1469-221X, 1469	10.15252/embr.2	https://onlinelibrary.wiley.com/doi/10.15252/embr.202050835		2021-04-07	2021-09-07 14:0	2021-10-14 0:16	2021-09-07 14:0			4	22		EMBO Rep								en		
RLZ5HZFJ	journalArticle	2021	Valliyodan, Babu	Genetic variation	Scientific Data		2052-4463	10.1038/s41597-	http://www.nature	Abstract We report characteristics of soybean genetic diversity and structure from the resequencir	2021-12	2021-09-07 14:0	2021-12-03 18:4	2021-09-07 14:0	50		1	8		Sci Data								en		
KAQXJLLK	thesis	2021	Alamo, Marlen Ac	Impact of climate change on local tree species richness in Mexico							2021	2021-09-07 14:0	2021-12-03 18:5													Queens College				PhD Thesis
SS7BPELV	report	2021	Gillan, Jeffrey K.;	Innovations to expand drone data collection and analysis for rangeland monitoring					http://biorxiv.org/l	Abstract In adaptive management of rangelands, monitoring is the vital link that connects management actio	2021-02-08	2021-09-07 14:0	2022-02-25 0:41	2021-09-07 14:0												Ecology		en		preprint
PUCVWY6L	journalArticle	2021	Juneau, Stephan	Jupyter-Enabled	Computing in Science & Engineeri		1521-9615, 1558	10.1109/MCSE.2	https://ieeexplore.ieee.org/document/9347681/		2021-03-01	2021-09-07 14:0	2021-12-03 18:4	2021-09-07 14:0	15-25		2	23		Comput. Sci. Eng.										
6SE8P4QW	report	2021	Subramaniam, S	Ribosome Profiling Reveals a Dichotomy Between Ribosome Occupancy of Nuclear-En					http://biorxiv.org/l	Abstract Huntington disease (HD) is caused by an expanded polyglutamine mutation in huntingtin (mHTT), w	2021-02-01	2021-09-07 14:0	2021-10-14 0:25	2021-09-07 14:0												Neuroscience		en		preprint
KCIEE9LE	journalArticle	2021	Guo, Jiarong; Bo	VirSorter2: a mul	Microbiome		2049-2618	10.1186/s40168-	https://microbiom	Abstract Background Viruses are a significant player in many biosphere and human ecos	2021-12	2021-09-07 14:0	2021-12-03 18:4	2021-09-07 14:0	37		1	9		Microbiome	VirSorter2							en		
BDQW5LA9	journalArticle	2021	Mickelson-Young	A protocol for genome-wide analysis of DNA replication timing in intact root tips							2021	2021-09-07 14:0	2021-10-14 0:22																	
VWWVGWSJ	journalArticle	2021	Hamad, Zhala Ja	Recourses utiliza	International Journal of Science and Business						2021	2021-09-07 14:0	2021-10-14 0:21		42–53		2	5												
2U6WWAUZ	report	2021	Henningsen, Eva	Identification of candidate susceptibility genes to <i>Puccinia graminis</i> f. sp. <i>tritic					http://biorxiv.org/l	Abstract Wheat stem rust disease caused by Puccinia graminis f. sp. tritici	2021-01-25	2021-09-07 14:0	2021-10-14 0:24	2021-09-07 14:0												Plant Biology		en		preprint
SZ37U3CJ	journalArticle	2021	Komissarov, Serg	Numerical simula	New Astronomy Reviews		13876473	10.1016/j.newar.	https://linkinghub.elsevier.com/retrieve/pii/S1387647321000014		2021-06	2021-09-07 14:0	2021-12-03 18:4	2021-09-07 14:0	101610			92		New Astronomy Reviews								en		
X8S5RKWM	report	2021	Moore, Ryan M.;	PASV: Automatic protein partitioning and validation using conserved residues					http://biorxiv.org/l	Abstract Background Increasingly, researchers use protein-coding genes from targe	2021-01-21	2021-09-07 14:0	2021-10-14 0:25	2021-09-07 14:0							PASV					Bioinformatics		en		preprint
DC8RBUIF	journalArticle	2021	Martínez-Meyer,	Rangewide habit	Diversity and Distributions		1366-9516, 1472	10.1111/ddi.1322	https://onlinelibrary.wiley.com/doi/10.1111/ddi.13222		2021-04	2021-09-07 14:0	2021-12-07 20:1	2021-09-07 14:0	642-654		4	27		Divers Distrib								en		
MNTZA85Q	report	2021	Crispatzu, Giuliar	The chromatin, topological and regulatory properties of pluripotency-associated poised					http://biorxiv.org/l	Abstract Poised enhancers (PEs) represent a limited and genetically distinct set of distal regulatory e	2021-01-19	2021-09-07 14:0	2021-10-14 0:17	2021-09-07 14:0												Molecular Biology		en		preprint
SPDW76G8	journalArticle	2021	Bello, Abubakar;	Diversity and Dis	Phytotaxa						2021	2021-09-07 14:0	2021-10-14 0:16		103–124		2	480												
25LYSI7X	journalArticle	2021	Tserej, Olga; Fee	Variation in leaf t	Biotropica		0006-3606, 1744	10.1111/btp.1291	https://onlinelibrary.wiley.com/doi/10.1111/btp.12919		2021-05	2021-09-07 14:0	2021-12-03 18:4	2021-09-07 14:0	868-878		3	53		Biotropica								en		
VKGCERFF	journalArticle	2021	Firrao, Giuseppe	Orthology-Based	Pathogens		2076-0817	10.3390/pathoge	https://www.mdpi	Xylella fastidiosa is a xylem-limited bacterium phylogenetically related to the xanthomonads, with an unusually larg	2021-01-07	2021-09-07 14:0	2021-10-14 0:18	2021-09-07 14:0	46		1	10		Pathogens								en		
2B5FG77Q	journalArticle	2021	Bellas, Christoph	Polinton-like virus	Microbiome		2049-2618	10.1186/s40168-	https://microbiom	Abstract Background Polintons are large mobile genetic elements found in the	2021-12	2021-09-07 14:0	2021-12-03 18:4	2021-09-07 14:0	13		1	9		Microbiome								en		
DIXY4LIG	journalArticle	2021	Lozano, Roberto;	Comparative evo	Nature Plants		2055-0278	10.1038/s41477-	http://www.nature.com/articles/s41477-020-00834-5		2021-01	2021-09-07 14:0	2021-12-07 20:1	2021-09-07 14:0	17-24		1	7		Nat. Plants								en		
QPLLP73U	report	2021	Wang, Bo; Jiao, Y	Pan-genome Analysis in Sorghum Highlights the Extent of Genomic Variation and Suga					http://biorxiv.org/l	ABSTRACT Sorghum bicolor , one of the most important grass crops around the world, harb	2021-01-04	2021-09-07 14:1	2021-10-14 0:25	2021-09-07 14:1												Genomics		en		preprint
IG8IV847	conferencePaper	2021	Stubbs, Joe; Car	Sustainability in the Tapis Framework				10.24251/HICSS	http://hdl.handle.net/10125/71495		2021	2021-09-07 14:1	2021-10-14 0:25	2021-09-07 14:1																
GCVW4V3S	journalArticle	2021	Cosi, Michele; Fc	StarBLAST: a sca	Journal of Open Source Education		2577-3569	10.21105/jose.00	https://jose.theoj.org/papers/10.21105/jose.00102		2021-04-27	2021-09-07 14:1	2021-10-14 0:17	2021-09-07 14:1	102		38	4		JOSE	StarBLAST									
UXNJPV7U	journalArticle	2021	Deshmukh, Rupe	Soybean transpo	Physiologia Plantarum		0031-9317, 1399	10.1111/ppl.1328	https://onlinelibrary.wiley.com/doi/10.1111/ppl.13287		2021-04	2021-09-07 14:1	2021-12-07 20:1	2021-09-07 14:1	756-770		4	171		Physiologia Plan	Soybean transporter database							en		
TYVF37T3	journalArticle	2021	Shimizu, Kentaro	De Novo Genom	Plant and Cell Physiology		1471-9053	10.1093/pcp/pca	https://academic.	Abstract Bread wheat is a major crop that has long been the focus of basic and breeding research. Assemb	2021-03-25	2021-09-07 14:1	2021-10-14 0:23	2021-09-07 14:1	8-27		1	62										en		
3HPAIBMU	journalArticle	2021	Rogers, Anna R;	The importance c	G3 Genes|Genomes|Genetics		2160-1836	10.1093/g3journa	https://academic.	Abstract High-dimensional and high-throughput genomic, field performance, and environmental data are be	2021-04-12	2021-09-07 14:1	2021-10-14 0:26	2021-09-07 14:1	jkaa050		2	11										en		
2FVV4H8B	book	2021		Arabidopsis protocols		978-1-07-160880-7 978-1-07-160879-1					2021	2021-09-07 14:1	2021-09-07 14:1			465						Methods in Mole	2200			Humana Press	New York, NY	eng		
752N7TM3	journalArticle	2021	Tello-Ruiz, Marce	Gramene 2021: I	Nucleic Acids Research		0305-1048, 1362	10.1093/nar/gkaa	https://academic.	Abstract Gramene (http://www.gramene.org), a knowledgebase founded on comparative functional analyse	2021-01-08	2021-09-07 14:1	2021-10-14 0:24	2021-09-07 14:1	D1452-D1463		D1	49			Gramene 2021							en		
HYPYQY8M	book	2021		Encyclopedia of Virology. Volume		978-0-12-814516-6			https://search.ebscohost.com/login.aspx?direct=true&scope=site&db=nlebk&db=nlabk&AN=2431995		2021	2021-09-07 14:1	2021-09-07 14:1	2021-09-07 14:1												ELSEVIER ACAD	Place of publicati	English		
9GQNZCVB	journalArticle	2021	Satjarak, Anchitt	Shotgun metager	Journal of Phycology		0022-3646, 1529	10.1111/jpy.1308	https://onlinelibrary.wiley.com/doi/10.1111/jpy.13084		2021-02	2021-09-07 14:1	2021-12-07 20:1	2021-09-07 14:1	39-50		1	57		J. Phycol.								en		
I75C3QT5	journalArticle	2021	Ponsero, Alise J;	Planet Microbe: a	Nucleic Acids Research		0305-1048, 1362	10.1093/nar/gkaa	https://academic.	Abstract In recent years, large-scale oceanic sequencing efforts have provided a deeper understanding of r	2021-01-08	2021-09-07 14:1	2021-10-14 0:25	2021-09-07 14:1	D792-D802		D1	49			Planet Microbe							en		
2BEI72GQ	bookSection	2021		2. Stabilizing Dig	Fields of practice	978-0-429-45954-2			https://books.goo	"Many different cognitive research approaches have been generated to explore fields of practice where mutual tea	2021	2021-09-07 14:1	2021-09-07 14:2													CRC Press, Tayl	Boca Raton			
6VT87L3T	journalArticle	2021	Park, Isaac W.; R	Advancing frost c	Global Change Biology		1354-1013, 1365	10.1111/gcb.1538	https://onlinelibrary.wiley.com/doi/10.1111/gcb.15380		2021-01	2021-09-07 14:2	2021-12-07 20:1	2021-09-07 14:2	165-176		1	27		Glob. Change Biol.								en		
MMLWCS28	journalArticle	2021	Zhang, Jun; Con	A national topogr	Earth System Science Data						2021	2021-09-07 15:2	2021-09-07 15:2		3263–3279		7	13												
4667JJAH	journalArticle	2021	Fuchsman, Clara	Cyanophage hos	Environmental Microbiology		1462-2912, 1462	10.1111/1462-29	https://onlinelibrary.wiley.com/doi/10.1111/1462-2920.15219		2021-06	2021-09-07 15:2	2021-12-03 18:4	2021-09-07 15:2	2782-2800		6	23		Environ Microbiol								en		
4TT3NUWP	journalArticle	2021	Conti, Matteo; Ni	Match Algorithms	Plants		2223-7747	10.3390/plants10	http://dx.doi.org/10.3390/plants10050974		2021-05	2021-10-12 20:0	2021-10-12 20:0		974		5	10												
N6UYE2S4	journalArticle	2021	Ferdausi, Aleya;	Transcriptomic ar	Industrial Crops and Products		0926-6690	https://doi.org/10	https://www.scier	The Amaryllidaceae genus Narcissus contains diversified secondary metabolites, which are important sources of b	2021	2021-10-12 20:0	2021-10-12 20:0		113615			168												
X58PIK3U	journalArticle	2021	Sahneh, Faryad;	Ten simple rules	PLOS Computational Biology		1553-7358	10.1371/journal.p	https://dx.plos.org/10.1371/journal.pcbi.1008879		2021-05-13	2021-10-12 21:0	2021-12-03 18:4	2021-10-12 21:0	e1008879		5	17		PLoS Comput Biol								en		
VDY6V9AR	journalArticle	2021	Wang, Xin; Liu, J	Biodiversity datas	Ecological Research		0912-3814, 1440	10.1111/1440-17	https://onlinelibrary.wiley.com/doi/10.1111/1440-1703.12240		2021-07	2021-10-12 21:1	2021-12-03 18:4	2021-10-12 21:1	755-761		4	36		Ecological Research								en		
7DX4AHZ3	bookSection	2021	Stubbs, Joe; Car	Tapis: An API Pla	Advances in Info	978-3-030-73099-4 978-3-030-73100-7			https://link.springer.com/10.1007/978-3-030-73100-7_61		2021	2021-10-12 21:1	2021-10-12 21:1	2021-10-12 21:1	878-900			1363			Tapis					Springer Internat	Cham	en		
C2RRGTFQ	journalArticle	2021	Kocherlakota, Pra	Constraints on bl	Physical Review D		2470-0010, 2470	10.1103/PhysRev	https://link.aps.org/doi/10.1103/PhysRevD.103.104047		2021-05-20	2021-10-12 21:2	2021-12-03 18:4	2021-10-12 21:2	104047		10	103		Phys. Rev. D								en		
32SNQVHW	journalArticle	2021	Adak, Alper; Mur	Unoccupied aeria	The Plant Genome		1940-3372, 1940	10.1002/tpg2.201	https://onlinelibrary.wiley.com/doi/10.1002/tpg2.20102		2021-07	2021-10-12 21:2	2021-12-03 18:4	2021-10-12 21:2			2	14		Plant Genome								en		
2WCW7EPY	journalArticle	2021	Zeng, Shuai; Mac	G2PDeep: a web	Nucleic Acids Research		0305-1048, 1362	10.1093/nar/gkab	https://academic.	Abstract G2PDeep is an open-access web server, which provides a deep-learning framework for quantitativ	2021-07-02	2021-10-12 21:2	2021-10-12 21:2	2021-10-12 21:2	W228-W236		W1	49			G2PDeep							en		
GLEQ6SJ3	conferencePaper	2021	Gudukbay, Gulsu	GYAN: Accelerat	2021 IEEE Intern	978-1-66543-577-2		10.1109/IPDPSW	https://ieeexplore.ieee.org/document/9460606/		2021-06	2021-10-12 21:2	2021-12-03 18:4	2021-10-12 21:2	194-203						GYAN					IEEE	Portland, OR, USA			

94VC7BAP	journalArticle	2021	Kadow, Christoph	Introduction to Fr	Journal of Open Research Softwar		2049-9647	10.5334/jors.253	http://openresearchsoftware.metajnl.com/articles/10.5334/jors.253/		2021-06-02	2021-10-12 21:3	2021-10-12 21:3	2021-10-12 21:3	13			9							en	
Q4J2QXQV	journalArticle	2021	Tisza, Michael J.	A catalog of tens	Proceedings of the National Acade		0027-8424, 1091	10.1073/pnas.20	http://www.pnas.	Despite remarkable strides in microbiome research, the viral component of the microbiome has generally presente	2021-06-08	2021-10-12 21:3	2021-10-12 21:3	2021-10-12 21:3	e2023202118		23	118	Proc Natl Acad Sci USA						en	
8CYKQWNH	journalArticle	2021	Cornell, Carolyn	Temporal Change	mSphere		2379-5042	10.1128/mSphere	https://journals.a	Conversion of land alters the physiochemical and biological environments by not only changing the aboveground c	2021-06-30	2021-10-12 21:3	2021-10-12 21:3	2021-10-12 21:3			3	6	mSphere						en	
8QU7IKLR	journalArticle	2021	Satjarak, Anchittl	Microscopic and	American Fern Journal		0002-8444	10.1640/0002-84	https://bioone.org/journals/american-fern-journal/volume-111/issue-2/0002-8444-111.2.86/Microscopic-and-Metagenomic-Evidence-f		2021-06-07	2021-10-12 21:3	2021-12-03 18:4	2021-10-12 21:3			2	111	American Fern Journal							
IP2DK5KF	journalArticle	2021	Ramos-Barbero,	Ancient saltern m	Environmental Microbiology		1462-2912, 1462	10.1111/1462-29	https://onlinelibrary.wiley.com/doi/10.1111/1462-2920.15630		2021-07	2021-10-12 21:4	2021-12-03 18:4	2021-10-12 21:4	3477-3498		7	23	Environ Microbio	Ancient saltern metagenomics					en	
UURWARKK	thesis	2021	Cho, Kyoung Tak	Discovering relationships between genotype and phenotype: Machine learning approaches							2021	2021-10-12 21:4	2021-10-12 21:4										Iowa State University			
YM3ZSW3E	document	2021	Gustafsson, Lena	Disturbance interval modulates the starting point for vegetation succession					https://doi.org/10.1002/ecy.3439		2021	2021-10-12 21:5	2021-10-12 21:5										Wiley Online Library			
2NJRYM7F	journalArticle	2021	Zayed, Ahmed A;	efam: an <i>e</i	Bioinformatics		1367-4803, 1460	10.1093/bioinfor	https://academic.	Abstract Motivation Viruses infect, reprogram and kill microbes, leading to profound ecos	2021-06-16	2021-10-12 21:5	2021-10-12 21:5	2021-10-12 21:5	btab451					efam					en	
5K6L3Q5P	journalArticle	2021	Zhang, Tao; Mud	Selective inherita	Nature Communications		2041-1723	10.1038/s41467-	http://www.nature	Abstract Sexual reproduction constrains progeny to inherit allelic genes from both parents. Selecti	2021-12	2021-10-12 21:5	2021-12-03 18:4	2021-10-12 21:5	3854		1	12	Nat Commun						en	
XLR33I6F	journalArticle	2021	Wulff, J. A.; Kjeld	From the bellies	Insectes Sociaux		0020-1812, 1420	10.1007/s00040-	https://link.springer.com/10.1007/s00040-021-00822-8		2021-08	2021-10-12 22:0	2021-12-03 18:4	2021-10-12 22:0	245-253		2-3	68	Insect. Soc.	From the bellies of babes					en	
AJVRZ785	thesis	2021	Anwar, Zobia	Investigating the Role of Onecut1 in Establishment of Cell-type-Specific Enhancers during Mouse Motor Neuron Differentiation							2021	2021-10-12 22:0	2021-10-12 22:0										University of Toronto (Canada)			PhD Thesis
2CQ9LZWN	journalArticle	2021	Li, Yüze; Li, Tong	Plastic film mulch	Plant and Soil		0032-079X, 1573	10.1007/s11104-	https://link.springer.com/10.1007/s11104-021-05060-2		2021-07-09	2021-10-12 22:1	2021-10-12 22:1	2021-10-12 22:1					Plant Soil						en	
QBI6GTC7	journalArticle	2021	Sorgini, Crystal A	The genetic arch	G3 Genes|Genomes|Genetics		2160-1836	10.1093/g3journ	https://academic.	Abstract With increased demand on freshwater resources for agriculture, it is imperative that more water-us	2021-09-06	2021-10-12 22:1	2021-10-12 22:1	2021-10-12 22:1	jkab222		9	11							en	
C35RKEGK	conferencePaper	2021	Hancock, David Y	Jetstream2: Acce	Practice and Exp	978-1-4503-8292-2		10.1145/343735	https://dl.acm.org/doi/10.1145/3437359.3465565		2021-07-17	2021-10-12 22:1	2021-10-12 22:1	2021-10-12 22:1	1-8					Jetstream2			ACM	Boston MA USA	en	
8G8EMTE8	journalArticle	2021	Zhong, Zhi-Ping;	Glacier ice archiv	Microbiome		2049-2618	10.1186/s40168-	https://microbiom	Abstract Background Glacier ice archives information, including microbiology, that helps r	2021-12	2021-10-12 22:1	2021-12-03 18:4	2021-10-12 22:1	160		1	9	Microbiome						en	
IQ8XSQ5Y	journalArticle	2021	Balatskyi, Volody	Cardiac-specific	Mitochondrion		15677249	10.1016/j.mito.20	https://linkinghub.elsevier.com/retrieve/pii/S1567724921000982		2021-09	2021-10-12 22:1	2021-12-03 18:4	2021-10-12 22:1	59-69			60	Mitochondrion						en	
G5DM6VEZ	journalArticle	2021	Hearnshaw, Jose	Organizing know	F1000Research		2046-1402	10.12688/f1000re	https://f1000rese	Enormous volumes of COVID-19 research data have been published and this continues to increase daily. This crea	2021-07-30	2021-10-12 22:2	2021-12-03 18:4	2021-10-12 22:2	703			10	F1000Res						en	
9BVSX6LD	journalArticle	2021	Baseggio, Mathe	Genome-wide as	G3 Genes|Genomes|Genetics		2160-1836	10.1093/g3journ	https://academic.	Abstract Despite being one of the most consumed vegetables in the United States, the elemental profile of	2021-08-07	2021-10-12 22:2	2021-10-12 22:2	2021-10-12 22:2	jkab186		8	11							en	
67HNZUC6	journalArticle	2021	Moeinizade, Sab	An Applied Deep	arXiv:2108.00952 [cs, eess]				http://arxiv.org/ab	For a global breeding organization, identifying the next generation of superior crops is vital for its success. Recogni	2021-08-02	2021-10-12 22:2	2021-10-12 22:2	2021-10-12 22:2												
ACPIJHUN	report	2021	Rodríguez-Ramo	Microbial Genome-Resolved Metaproteomic Analyses Frame Intertwined Carbon and N					https://www.rese	Abstract Background: Rivers serve as a nexus for nutrient transfer between terrestrial and m	2021-08-02	2021-10-12 22:2	2021-10-12 22:2	2021-10-12 22:2									In Review			preprint
74M49XLP	journalArticle	2021	Zeng, Shuai; Škr	Application of SN	International Journal of Data Minin		1748-5673, 1748	10.1504/IJDMB.2	http://www.inderscience.com/link.php?id=116886		2021	2021-10-12 22:3	2021-10-12 22:3	2021-10-12 22:3	65		1/2	25	IJDMB						en	
ZE9B5KE4	document	2021	Kumar, Anirudh;	Omics Technologies for Sustainable Agriculture and Global Food Security (Vol II)					https://link.springer.com/content/pdf/10.1007/978-981-16-2956-3.pdf		2021	2021-10-12 22:3	2021-12-03 18:5										Springer			
7IH9CZLH	conferencePaper	2021	Deligiannakis, Ar	Extreme-Scale In	Proceedings of the 2nd Workshop on Search, Exploration, and Analys				http://pages.cs.aueb.gr/~kotidis/Publications/SEAData2021.pdf		2021	2021-10-12 22:4	2021-10-12 22:4		7–13											
YR3YC2HQ	journalArticle	2021	Wieser, Florian; S	A local platform fo	Journal of Biotechnology		1681656	10.1016/j.jbiotec.	https://linkinghub.elsevier.com/retrieve/pii/S0168165621002091		2021-11	2021-10-12 22:4	2021-12-03 18:4	2021-10-12 22:4	43-50			341	Journal of Biotechnology						en	
PXIMNT4A	journalArticle	2021	Pinto, Renan T.;	Genomic and tra	Plant Science		1689452	10.1016/j.plantsc	https://linkinghub.elsevier.com/retrieve/pii/S0168945221002144		2021-11	2021-10-12 22:4	2021-12-03 18:4	2021-10-12 22:4	111018			312	Plant Science	Genomic and transcriptomic inventory of membrane transporters in coffee					en	
UNGMIZ36	journalArticle	2021	Beck, Michael A.	Presenting an ex	arXiv:2108.05789 [cs]				http://arxiv.org/ab	We present two large datasets of labelled plant-images that are suited towards the training of machine learning and	2021-08-12	2021-10-12 22:4	2022-02-25 2:35	2021-10-12 22:4												
WWIUUHLP	journalArticle	2021	Ehsani, Mohamm	Nowcasting-Nets	arXiv:2108.06868 [cs]				http://arxiv.org/ab	Accurate and timely estimation of precipitation is critical for issuing hazard warnings (e.g., for flash floods or landsli	2021-08-15	2021-10-12 22:4	2021-10-12 22:4	2021-10-12 22:4						Nowcasting-Nets						
TSLR4SCM	journalArticle	2021	Yue, Longkai; Wa	Impacts of soil co	Soil and Tillage Research		1671987	10.1016/j.still.202	https://linkinghub.elsevier.com/retrieve/pii/S0167198721002397		2021-10	2021-10-12 22:5	2021-12-03 18:4	2021-10-12 22:5	105166			214	Soil and Tillage Research						en	
S8B4C4FW	journalArticle	2021	Katiyar, Neerja; F	Transcriptomic pr	Plant Gene		2352-4073	https://doi.org/10	https://www.scien	Rising global temperatures have caused a substantial loss in crop productivity throughout the world. Understanding	2021	2021-10-12 22:5	2021-10-12 22:5		100330			28								
BJHALE5Z	journalArticle	2021	Piombo, Edoardo	Sequencing of no	Fungal Genetics and Biology		10871845	10.1016/j.fgb.202	https://linkinghub.elsevier.com/retrieve/pii/S1087184521001067		2021-11	2021-10-12 23:0	2021-12-03 18:4	2021-10-12 23:0	103622			156	Fungal Genetics and Biology						en	
P52K8YL8	journalArticle	2021	Nakayama, Hoku	Leaf form diversit	Current Biology		9609822	10.1016/j.cub.20	https://linkinghub.elsevier.com/retrieve/pii/S0960982221011271		2021-09	2021-10-12 23:0	2021-12-03 18:4	2021-10-12 23:0	S0960982221011271				Current Biology						en	
XEBI584A	journalArticle	2021	Geyer, Philipp; S	Explainable AI fo	arXiv preprint arXiv:2108.13836						2021	2021-10-12 23:0	2021-10-12 23:0													
F6ZLKR2V	journalArticle	2021	Runck, Bryan C;	Digital agriculture	Agronomy Journal		0002-1962, 1435	10.1002/agj2.208	https://onlinelibrary.wiley.com/doi/10.1002/agj2.20873		2021-08-30	2021-10-12 23:0	2021-10-12 23:0	2021-10-12 23:0	agj2.20873				Agron.j.	Digital agriculture platforms					en	
SJEDRRSP	journalArticle	2021	Anderson, Nadja	Online Instruction	The American Biology Teacher		0002-7685, 1938	10.1525/abt.202	https://online.ucp	In the spring of 2020, remote learning was implemented in schools throughout the world due to the pandemic of SA	2021-09-01	2021-10-12 23:0	2021-10-12 23:0	2021-10-12 23:0	464-471		7	83							en	
B83YG486	journalArticle	2021	Kavas, Musa; Yıl	Genome-wide ide	Physiology and Molecular Biology		0971-5894, 0974	10.1007/s12298-	https://link.springer.com/10.1007/s12298-021-01052-9		2021-09	2021-10-12 23:0	2021-12-03 18:4	2021-10-12 23:0	1885-1902		9	27	Physiol Mol Biol Plants						en	
FP5LUVVY	journalArticle	2021	Neves, Danilo M.	The adaptive cha	Proceedings of the National Acade		0027-8424, 1091	10.1073/pnas.20	http://www.pnas.	The tropical conservatism hypothesis (TCH) posits that the latitudinal gradient in biological diversity arises because	2021-09-14	2021-10-12 23:0	2021-10-12 23:0	2021-10-12 23:0	e2021132118		37	118	Proc Natl Acad Sci USA						en	
H5Q45XJ7	journalArticle	2021	Stanschewski, Cl	Quinoa Phenotyp	Plants		2223-7747	10.3390/plants10	https://www.mdpi	Quinoa is a crop originating in the Andes but grown more widely and with the genetic potential for significant furthe	2021-08-24	2021-10-12 23:0	2021-10-12 23:0	2021-10-12 23:0	1759		9	10	Plants	Quinoa Phenotyping Methodologies					en	
JCQXJVGF	book	2021	Melendrez, M.C.;	Curriculum Applications In Microbi		978-2-88971-268-7			https://books.google.com/books?id=orFBEAAAQBAJ		2021	2021-10-12 23:1	2021-10-12 23:1								Frontiers Research Topics		Frontiers Media SA			
NCQJJ8J2	journalArticle	2021	Peterson, April L.	Higher Intercellul	Cytogenetic and Genome Researc		1424-8581, 1424	10.1159/000516	https://www.karg	Meiotic recombination affects fertility, shuffles genomes, and modulates the effectiveness of natural selection. Desp	2021-09-10	2021-10-12 23:1	2021-12-03 18:4	2021-10-12 23:1	1-7				Cytogenet Genome Res						en	
G9QA6TCM	journalArticle	2021	Chen, Yiqiang; W	Prokaryotic virus	Nature Communications		2041-1723	10.1038/s41467-	https://www.natu	Abstract As one of the largest biotechnological applications, activated sludge (AS) systems in wastewater tr	2021-12	2021-10-12 23:1	2021-12-03 18:4	2021-10-12 23:1	5398		1	12	Nat Commun						en	
NIYHJ7N5	journalArticle	2021	Rubio-Portillo, Es	Metagenomics U	mSystems		2379-5077	10.1128/mSyster	https://journals.a	Posidonia oceanica is a long-living and very slow-growing marine seagrass endemic to the Mediterranean	2021-09-14	2021-10-12 23:1	2021-10-12 23:1	2021-10-12 23:1					mSystems						en	
DH9V5QK3	journalArticle	2021	Lee, Yan-Jiun; Da	Pathways of thym	Nucleic Acids Research		0305-1048, 1362	10.1093/nar/gkab	https://academic.	Abstract The DNAs of bacterial viruses are known to contain diverse, chemically complex modifications to t	2021-09-15	2021-10-12 23:1	2021-10-12 23:1	2021-10-12 23:1	gkab781										en	
9RBYYW7T	journalArticle	2021	Woltz, Megan	Barcoding US An	The Confluence				https://digitalcommons.lindenwood.edu/cgi/viewcontent.cgi?article=1005&context=theconfluence		2021	2021-10-12 23:1	2021-10-12 23:1		3		1	1								
5TJ4WJ4P	bookSection	2021	Boopathi, N. Mar	Molecular Marke	The Moringa Ger	978-3-030-80955-3 978-3-030-80956-0			https://link.springer.com/10.1007/978-3-030-80956-0_9		2021	2021-10-12 23:1	2021-10-12 23:1	2021-10-12 23:1	85-100								Springer Internat	Cham	en	
BE78UZ8M	thesis	2021	Rengifo Faiffer, M	From Community to Transcriptome: Responses of 0RW1S34RfeSDcfkexd09rT2Syntrich					http://ezproxy.libr	The increasing temperatures and severity of droughts are modifying dryland ecosystems worldwide and reducing t	2021	2021-10-12 23:1	2021-10-12 23:1			144									English	PhD Thesis
EZE8E97T	journalArticle	2021	Bedre, Renesh; A	HTSQualC is a fl	Scientific Reports		2045-2322	10.1038/s41598-	https://www.natu	Abstract Use of high-throughput sequencing (HTS) has become indispensable in life science research. Rav	2021-12	2021-10-12 23:2	2021-12-03 18:4	2021-10-12 23:2	18725		1	11	Sci Rep						en	
65CBVB9R	report	2021	Bendaoud, Farah	GBS analysis of Orobanche crenata populations in Algeria supports local adaptation an					https://www.auth	Crenate broomrape (Orobanche crenata Forsk.) is a serious long-standing parasitic weed problem in Algeria, main	2021-09-24	2021-10-12 23:2	2021-12-03 18:4	2021-10-12 23:2									Preprints			preprint
SYFNMKMU	journalArticle	2021	Macías-Duarte, A	Geographic varia	Behavioral Ecology		1045-2249, 1465	10.1093/beheco/	https://academic.	Abstract Dispersal is one of the key elements of species' metapopulation dynamics and, hence, influences	2021-09-24	2021-10-12 23:2	2021-10-12 23:2	2021-10-12 23:2	arab100										en	
QJF26XX8	conferencePaper	2021	Maximova, Irina;	Study of XAI-cap	2021 Internationa	978-1-66543-900-8		10.1109/IJCNN5	https://ieeexplore.ieee.org/document/9534105/		2021-07-18	2021-10-12 23:2	2021-12-03 18:4	2021-10-12 23:2	1-8								IEEE	Shenzhen, China		
6PIPACZI	journalArticle	2021	Zhang, Jie; Cava	Timing RNA poly	Cell Reports Methods		26672375	10.1016/j.crmeth	https://linkinghub.elsevier.com/retrieve/pii/S2667237521001387		2021-09	2021-10-12 23:2	2021-12-03 18:4	2021-10-12 23:2	100083				Cell Reports Methods						en	
85AZDT2M	journalArticle	2021	Fesenko, Igor; Sl	A vast pool of line	Nucleic Acids Research		0305-1048, 1362	10.1093/nar/gkab	https://academic.	Abstract Pervasive transcription of eukaryotic genomes results in expression of long non-coding RNAs (Incl	2021-10-11	2021-10-12 23:2	2021-10-12 23:2	2021-10-12 23:2	10328-10346		18	49							en	
ES82IQR2	journalArticle	2021	Blais, Brian R; Sn	Phylogeography	Biological Journal of the Linnean S		0024-4066, 1095	10.1093/biolinnea	https://academic.	Abstract Phylogeographic studies can uncover robust details about the population structure, demographics	2021-09-30	2021-10-12 23:2	2021-10-12 23:2	2021-10-12 23:2	blab124					Phylogeography of the smooth greensnake, <i>Opheodrys vernalis</i> (Squamata					en	
WQBZUVMM	thesis	2021	Kusmec, Aaron M	Phenotypic Plasticity and Breeding for Future Climates: Case Studies in Zea mays					http://ezproxy.libr	Plant breeding has been enormously successful at increasing crop yields over the course of the 20th century, but fu	2021	2021-10-12 23:2	2021-10-12 23:2			206									English	PhD Thesis
GGPLQFUC	bookSection	2021	Alex Mason, G.;	Bioinformatic Too	Arabidopsis Prote	978-1-07-160879-1 978-1-07-160880-7			http://link.springer.com/10.1007/978-1-0716-0880-7_2		2021	2021-10-12 23:3	2021-10-12 23:3	2021-10-12 23:3	25-89			2200					Springer US	New York, NY	en	
UUMQ7PA5	artwork	2021	Lama, Tseten; Mi	Biodiversity of Plants in Highly Polluted Areas [Poster]					https://dnabarcoding101.org/files/uploads/0000/ubp/joralemon-2997-poster-NCJNT.pdf		2021-05-12	2021-10-12 23:5	2021-12-03 18:4													
DP2IV5X3	journalArticle	2021	Lo, Chien-Chi; Sh	EDGE COVID-19	arXiv:2006.08058 [q-bio]				http://arxiv.org/ab	Genomics has become an essential technology for surveilling emerging infectious disease outbreaks. A wide range	2021-06-24	2021-10-12 23:5	2021-10-12 23:5	2021-10-12 23:5						EDGE COVID-19						
78WWW3JQ	journalArticle	2021	Gazitúa, M. Cons	Potential virus-m	The ISME Journal		1751-7362, 1751	10.1038/s41396-	http://www.nature	Abstract Viruses play an important role in the ecology and biogeochemistry of marine ecosystems	2021-04	2021-10-13 23:5	2021-12-07 20:2	2021-10-13 23:5	981-998		4	15	ISME J						en	
DTQ9ILKH	bookSection	2021	Pappas, Nikolaos	Virus Bioinformat	Encyclopedia of V	978-0-12-814516-6			https://linkinghub.elsevier.com/retrieve/pii/B9780128145159000345		2021	2021-10-14 0:08	2021-10-14 0:08	2021-10-14 0:08	124-132								Elsevier		en	
GLCZ7MT7	journalArticle	2021	Ostezan, Alexand	Target region seq	Journal of Crop Science and Biote		1975-9479, 2005	10.1007/s12892-	https://link.springer.com/10.1007/s12892-020-00056-3		2021-01	2021-10-14 0:08	2021-12-07 20:2	2021-10-14 0:08	13-26		1	24	J. Crop Sci. Biotechnol.						en	
BGPN7THG	journalArticle	2021	Reiter, Taylor; Br	Streamlining data	GigaScience		2047-217X	10.1093/gigascie	https://academic.	Abstract As the scale of biological data generation has increased, the bottleneck of research has shifted fro	2021-01-13	2021-10-14 0:10	2021-10-14 0:10	2021-10-14 0:10	giaa140		1	10							en	
CCKWEQCL	bookSection	2021	Patel, Neha P.; Ja	Advancement in	Microbial and Na	978-0-12-820084-1			https://linkinghub.elsevier.com/retrieve/pii/B9780128200841000193		2021	2021-10-14 0:10	2021-10-14 0:10	2021-10-14 0:10	455-493								Elsevier		en	
9GH2JNMX	journalArticle	2021	Peterson, April L;	Sex-specific varia	Genetics		1943-2631	10.1093/genetics	https://academic.	Abstract In most species that reproduce sexually, successful gametogenesis requires recombination during	2021-03-03	2021-10-14 0:10	2021-10-14 0:10	2021-10-14 0:10	iyaa019		1	217							en	
WG42I8DM	journalArticle	2021	Lin, Joshua Yao-	VLBInet: Radio Ir	arXiv:2110.07185 [astro-ph]				http://arxiv.org/ab	The Event Horizon Telescope (EHT) recently released the first horizon-scale images of the black hole in M87. Com	2021-10-14	2022-02-24 21:5	2022-02-24 21:5	2022-02-24 21:5						VLBInet						
S8YRX5DK	thesis	2021	Jensen, Sarah E.	Predicting Protein Temperature Adaptation from Prokaryotes to Plants					https://ezproxy.lib	All domains of life are affected by temperature. Environmental temperature affects both macro- and molecular featu	2021	2022-02-24 21:5	2022-02-24 21:5			172									English	PhD Thesis
E36QLV8C	journalArticle	2021	Tan, Pengpeng; I	Ion transporters a	Plant Growth Regulation		0167-6903, 1573	10.1007/s10725-	https://link.springer.com/10.1007/s10725-021-00762-0		2021-10-19	2022-02-24 21:5	2022-02-24 22:0	2022-02-24 21:5	1-23		1	96	Plant Growth Regul						en	
RF8IMXA8	report	2021	Swartz, Landon C	Tracking dynamic changes of leaves in response to nutrient availability using an open-s					http://www.essoar.org/doi/10.1002/essoar.10508290.1		2021-10-15	2022-02-25 1:13	2022-02-25 1:13	2022-02-25 1:13									Agricultural, Veterinary and Food S		en	preprint
BC4NNQDT	conferencePaper	2021	Ketron, R.; Leona	A Case Study in	2021 IEEE 17th I	978-1-66540-361-0		10.1109/eScienc	https://ieeexplore.ieee.org/document/9582335/		2021-09	2022-02-25 1:19	2022-02-25 1:19	2022-02-25 1:19	249-250								IEEE	Innsbruck, Austria		
ZVVS2VVE	journalArticle	2021	Metzger, Stefan;	Novel approach t	Atmospheric Measurement Techni		1867-8548	10.5194/amt-14-	https://amt.coper	Abstract. The observing system design of multidisciplinary field measurements involves a variety of considerations	2021-11-01	2022-02-25 1:23	2022-02-25 1:23	2022-02-25 1:23	6929-6954		11	14	Atmos. Meas. Tech.						en	
XYZX6BMV	journalArticle	2021	Zhao, Lihua; Liu,	Characterization	Horticulture Research		2662-6810, 2052	10.1038/s41438-	https://www.natu	Abstract Understanding germline specification in plants could be advantageous for agricultural applications.	2021-12	2022-02-25 1:24	2022-02-25 1:24	2022-02-25 1:24	239		1	8	Hortic Res						en	
JTLK98QP	report	2021	Senn, Savanah;	Microbes in the Datura Rootzone Contribute to an Antioxidant Support System of Flavo					https://www.prep	The purpose of this paper is to elucidate the roles that microbes may be playing in the rootzone of the medicinal pla	2021-11-03	2022-02-25 1:26	2022-02-25 1:26	2022-02-25 1:26									BIOLOGY			preprint
J5I9K79E	manuscript	2021	Satapathy, Kaush	The Variability of the Black-Hole Image in M87 at the Dynamical Time Scale							2021-11-03	2022-02-25 1:40	2022-02-25 1:43													
4L9W9Z5Y	journalArticle	2021	Xin, Zhanguo; W	Sorghum genetic	Planta		0032-0935, 1432	10.1007/s00425-	https://link.spring	Abstract Main conclusion Sorghum research has entered an exciting and fruitful era due	2021-12	2022-02-25 1:46	2022-02-25 1:46	2022-02-25 1:46	114		6	254	Planta						en	
5VHEZK3X	journalArticle	2021	Varshney, Rajeev	A chickpea genet	Nature		0028-0836, 1476	10.1038/s41586-	https://www.natu	Abstract Zero hunger and good health could be realized by 2030 through effective conservation, c	2021-11-25	2022-02-25 1:51	2022-02-25 1:51	2022-02-25 1:51	622-627		7886	599	Nature						en	
3CDLARXR	journalArticle	2021	Gu, Chengxiang;	Saline lakes on th	iScience		25890042	10.1016/j.isci.202	https://linkinghub.elsevier.com/retrieve/pii/S2589004221014103		2021-12	2022-02-25 1:53	2022-02-25 1:53	2022-02-25 1:53	103439		12	24	iScience						en	
RGC6K9YQ	conferencePaper	2021	George, Simon; A	Phantom Echoes	Proc 22nd AMOS Conf						2021	2022-02-25 1:55	2022-02-25 1:55													
M5AAQL2M	journalArticle	2021	Schmitz, Alexa M	Generation of a C	Nature Communications		2041-1723	10.1038/s41467-	https://www.natu	Abstract Bioleaching of rare earth elements (REEs), using microorganisms such as Gluc	2021-12	2022-02-25 2:00	2022-02-25 2:00	2022-02-25 2:00	6693		1	12	Nat Commun						en	
AE3EBQDY	journalArticle	2021	Happel, Anna-Urs	Presence and Pe	Viruses		1999-4915	10.3390/v131223	https://www.mdpi	The interaction between gut bacterial and viral microbiota is thought to be important in human health. While fluctua	2021-11-23	2022-02-25 2:10	2022-02-25 2:10	2022-02-25 2:10	2341		12	13	Viruses						en	
MTHMB3NX	journalArticle	2021	McLean, Jared; C	Building a portal	Concurrency and Computation: Pra		1532-0626, 1532	10.1002/cpe.672	https://onlinelibrary.wiley.com/doi/10.1002/cpe.6727		2021-11-22	2022-02-25 2:14	2022-02-25 2:14	2022-02-25 2:14					Concurrency Computat Pract Exper						en	
K6XXMHSQ	journalArticle	2021	Morales, Nicolas;	Spatio-temporal modeling of high-throughput multi-spectral images improves agronomic trait genomic prediction in hybrid maize							2021	2022-02-25 2:15	2022-02-25 2:15													
8RKLYEDD	journalArticle	2021	Qin, Clara; Barte	From DNA seque	Ecosphere		2150-8925, 2150	10.1002/ecs2.38	https://onlinelibrary.wiley.com/doi/10.1002/ecs2.3842		2021-11	2022-02-25 2:20	2022-02-25 2:20	2022-02-25 2:20			11	12	Ecosphere	From DNA sequences to microbial ecology					en	
KSQCWGGE	journalArticle	2021	Brzozowski, Laur	Selection for see	G3 Genes|Genomes|Genetics		2160-1836	10.1093/g3journ	https://academic.	Abstract Plant breeding strategies to optimize metabolite profiles are necessary to develop health-promotin	2021-12-10	2022-02-25 2:33	2022-02-25 2:43	2022-02-25 2:33	jkab419										en	
3GL87SFS	journalArticle	2021	Beck, Michael A;	An extensive lab-and field-image dataset of crops and weeds for computer vision tasks in agriculture							2021	2022-02-25 2:34	2022-02-25 4:09													
TIAMAPTS	journalArticle	2021	Nagy, R Chelsea	Harnessing the N	Ecosphere						2021	2022-02-25 2:39	2022-02-25 2:39		e03833		12	12								
YZLIKHZD	journalArticle	2021	Cherednichenko,	Vascular plant oc	Biodiversity Data Journal		1314-2828, 1314	10.3897/BDJ.9.e	https://bdj.penso	Here we present the sampling event dataset that contributes to studying the flora of grasslands in Central Forest S	2021-12-10	2022-02-25 2:44	2022-02-25 2:44	2022-02-25 2:44	e76806			9	BDJ	Vascular plant occurrences in grasslands of Central Forest Nature Reserve (Russia)						
NZIXPJVE	journalArticle	2021	Kim, Yoonha	Imagery Acquisiti	The Korean Journal of Crop Science						2021-12-01	2022-02-25 2:54	2022-02-25 2:56		452-458											
VJSA8TCS	journalArticle	2021	Vuorinen, Katarii	Functional traits	Ecosphere		2150-8925, 2150	10.1002/ecs2.38	https://onlinelibrary.wiley.com/doi/10.1002/ecs2.3887		2021-12	2022-02-25 2:56	2022-02-25 2:56	2022-02-25 2:56			12	12	Ecosphere						en	
MJJNIBWC	conferencePaper	2021	Pandey, Ashish;	VECTrust: truste	Proceedings of th	978-1-4503-8564-0		10.1145/3468737	https://dl.acm.org/doi/10.1145/3468737.3494099		2021-12-06	2022-02-25 2:58	2022-02-25 2:58	2022-02-25 2:58	1-10					VECTrust			ACM	Leicester United	en	
8S4LMG6N	journalArticle	2021	Yan, Haidong; Ha	Exploring transpo	Plant Communications		25903462	10.1016/j.xplc.20	https://linkinghub.elsevier.com/retrieve/pii/S259034622100184X		2021-12	2022-02-25 3:03	2022-02-25 3:03	2022-02-25 3:03	100270				Plant Communications						en	
Z39C7V6J	journalArticle	2021	de Deus Vidal, Jc	Beyond the Tree-	Frontiers in Ecology and Evolution		2296-701X	10.3389/fevo.202	https://www.front	von Humboldt's tree-line concept has dominated mountain ecology for almost two hundred years, and is considere	2021-12-07	2022-02-25 3:07	2022-02-25 3:07	2022-02-25 3:07	760118			9	Front. Ecol. Evol.	Beyond the Tree-Line						
Z4MZSAZ7	thesis	2021	Lichwa, Evelyn M	Ecological and social drivers of Mexican gray wolf (Canis lupus baileyi) home range patterns across spatiotemporal scales							2021-12	2022-02-25 3:17	2022-02-25 3:18										Humboldt State University			Masters Thesis
ZLN9KMVM	thesis	2021	Joyce, Elizabeth	Evolution of the northern Australian flora: role of the Sunda-Sahul Floristic Exchange							2021	2022-02-25 3:50	2022-02-25 3:50										James Cook University			PhD Thesis
A4L8Z7SY	journalArticle	2021	Malhotra, S; Kau	Adhesion to Enar	Science Progress and Research		26350955, 2635	10.52152/spr/202	https://doi.org/htt	Bonding agents play a crucial role in the effective sealing and retention of resin-based composite restorations, whic	2021-10-05	2022-02-25 4:04	2022-02-25 4:04	2022-02-25 4:04	368-376		4	1	SPR	Adhesion to Enamel and Dentin						
PA379LYF	journalArticle	2021	Janssen, Michae	Event Horizon Te	Nature Astronomy		2397-3366	10.1038/s41550-	https://www.natu	Abstract Very-long-baseline interferometry (VLBI) observations of active galactic nuclei at millimet	2021-10	2021-10-12 22:1	2022-03-01 22:0	2022-02-25 4:07	1017-1028		10	5	Nat Astron						en	
AQHS88VL	book	2020	Samal, Ashok; Cl	Intelligent Image Analysis for Plant Phenotyping							2020	2021-09-07 13:5	2021-10-14 0:24										CRC Press			
3C65C5M3	thesis	2020	Stahlman, Gretch	Exploring the long tail of astronomy: A mixed-methods approach to searching for dark d					https://repository.arizona.edu/bitstream/handle/10150/642070/azu_etd_18157_sip1_m.pdf?sequence=1		2020	2021-09-07 14:1	2021-10-14 0:23										The University of Arizona			PhD Thesis
W666TX35	book	2020		Microbial and natural macromolecu		978-0-12-820084-1				"Illustrates fundamental discoveries and methodological advancements Discusses novel functional attributes of ma	2020	2021-09-07 14:2	2021-09-07 14:2							Microbial and natural macromolecules			Elsevier	Waltham		
57TFW3I3	report	2020	Liu, Jianing; Seet	Gapless assembly of maize chromosomes using long read technologies					http://biorxiv.org/	Abstract Creating gapless telomere-to-telomere assemblies of complex genomes is one of the ultimate challe	2020-01-15	2021-09-07 15:0	2021-09-07 15:0	2021-09-07 15:0									Bioinformatics		en	preprint
PVXDZJ9S	journalArticle	2020	Wegrzyn, Jill L.; I	Cyberinfrastructu	Evolutionary Applications		1752-4571, 1752	10.1111/eva.128	https://onlinelibrary.wiley.com/doi/10.1111/eva.12860		2020-01	2021-09-07 15:0	2021-12-07 20:1	2021-09-07 15:0	228-241		1	13	Evol Appl						en	
CB77RI56	journalArticle	2020	Staude, Ingmar R	Range size predi	Global Ecology and Biogeography		1466-822X, 1466	10.1111/geb.130	https://onlinelibrary.wiley.com/doi/10.1111/geb.13003		2020-01	2021-09-07 15:0	2021-12-07 20:1	2021-09-07 15:0	16-25		1	29	Global Ecol Biogeogr						en	
NF52KISZ	journalArticle	2020	Knight, Clarke A;	Community asse	The American Naturalist						2020	2021-09-07 15:0	2021-09-07 15:0		166–180		2	195								
ZJPXXVSA	journalArticle	2020	Gemeinholzer, Bi	Data storage and	Organisms Diversity & Evolution		1439-6092, 1618	10.1007/s13127-	http://link.springe	Abstract The ability to rapidly generate and share molecular, visual, and acoustic data, and to compare ther	2020-03	2021-09-07 15:0	2021-12-07 20:1	2021-09-07 15:0	1-8		1	20	Org Divers Evol						en	
U2JHAV3E	book	2020		Stem cell transcriptional networks:		978-1-07-160301-7 978-1-07-160300-0					2020	2021-09-07 15:1	2021-09-07 15:1			308				Stem cell transcr	Methods in mole	2117	Humana Press	New York, NY	eng	
VGFA2IVF	report	2020	Bezuidt, Oliver K	Phages actively challenge niche communities in the Antarctic soils					http://biorxiv.org/	Abstract By modulating the structure, diversity and trophic outputs of microbial communities, phages play cru	2020-01-20	2021-09-07 15:1	2021-09-07 15:1	2021-09-07 15:1									Microbiology		en	preprint
42IB4KWD	journalArticle	2020	Debauche, Olivie	Cloud architectur	Concurrency and Computation: Pr		1532-0626, 1532	10.1002/cpe.566	https://onlinelibrary.wiley.com/doi/10.1002/cpe.5661		2020-09-10	2021-09-07 15:1	2021-09-07 15:1	2021-09-07 15:1			17	32	Concurrency Computat Pract Exper						en	
3KJWH6LT	report	2020	Zhong, Zhi-Ping;	Glacier ice archives fifteen-thousand-year-old viruses					http://biorxiv.org/	Abstract While glacier ice cores provide climate information over tens to hundreds of thousands of ye	2020-01-07	2021-09-07 15:1	2021-09-07 15:1	2021-09-07 15:1									Ecology		en	preprint
YZU85H2B	journalArticle	2020	Coffman, Stepha	Haplotype structu	Theoretical and Applied Genetics		0040-5752, 1432	10.1007/s00122-	http://link.springer.com/10.1007/s00122-019-03486-y		2020-02	2021-09-07 15:1	2021-12-07 20:1	2021-09-07 15:1	547-561		2	133	Theor Appl Genet						en	

GJN9XW43	book	2020	Alhajj, Reda; Mo:	Data Management and Analysis: C		978-3-030-32587-9			https://doi.org/10.1007/978-3-030-32587-9		2020	2021-09-07 15:1	2021-09-07 15:1	2021-09-07 15:1							Data Management and Analysis							English		
ZZDSLDPU	book	2020		Data management and analysis: c		978-3-030-32586-2					2020	2021-09-07 15:1	2021-09-07 15:1			260					Data management and analysis					Springer Internat	Cham	eng		
ZB9RCZTK	journalArticle	2020	Falcon, Celeste M	Relative utility of	Crop Science		0011-183X, 1435	10.1002/csc2.20	https://onlinelibrary.wiley.com/doi/10.1002/csc2.20035		2020-01	2021-09-07 15:1	2021-12-07 20:1	2021-09-07 15:1	62-81		1	60		Crop Sci.								en		
D83XAX9M	journalArticle	2020	Howard-Varona,	Phage-specific m	The ISME Journal		1751-7362, 1751	10.1038/s41396-	http://www.nature	Abstract Ocean viruses are abundant and infect 20–40% of surface microbes. Infected cells, term	2020-04	2021-09-07 15:1	2021-12-07 20:1	2021-09-07 15:1	881-895		4	14		ISME J								en		
7LDUAG4T	report	2020	Tirado, Sara B.;	Utilizing top-down hyperspectral imaging for monitoring genotype and growth conditions					http://biorxiv.org/	Abstract There is significant enthusiasm about the potential for hyperspectral imaging to document variation	2020-01-23	2021-09-07 15:1	2021-09-07 15:1	2021-09-07 15:1												Plant Biology		en		preprint
2ESEXWF2	journalArticle	2020	Coate, Jeremy E	Robust Cytonucl	Genes		2073-4425	10.3390/genes11	https://www.mdpi	Polyploidy is hypothesized to cause dosage imbalances between the nucleus and the other genome-containing org	2020-01-28	2021-09-07 15:1	2021-09-07 15:1	2021-09-07 15:1	134		2	11		Genes								en		
CASYUTXP	journalArticle	2020	Joly-Lopez, Zoé;	An inferred fitnes	Nature Plants		2055-0278	10.1038/s41477-	http://www.nature.com/articles/s41477-019-0589-3		2020-02	2021-09-07 15:1	2021-12-07 20:1	2021-09-07 15:1	119-130		2	6		Nat. Plants								en		
F8XLHTQD	journalArticle	2020	García-Portela, M	Uptake of Inorga	Microorganisms		2076-2607	10.3390/microorg	https://www.mdpi	Dinoflagellate species of Dinophysis are obligate mixotrophs that require light, nutrients, and prey for sustained gro	2020-01-29	2021-09-07 15:1	2021-09-07 15:1	2021-09-07 15:1	187		2	8		Microorganisms								en		
9HGLJ7HP	journalArticle	2020	McFarland, Bridg	Maize genomes t	BMC Research Notes		1756-0500	10.1186/s13104-	https://bmcresno	Abstract Objectives Advanced tools and resources are needed to efficiently and sustainal	2020-12	2021-09-07 15:1	2021-12-07 20:1	2021-09-07 15:1	71		1	13		BMC Res Notes	Maize genomes to fields (G2F)							en		
6KAQFHNV	journalArticle	2020	Li, Juan; Demesy	Development of s	Molecular Biology Reports		0301-4851, 1573	10.1007/s11033-	http://link.springer.com/10.1007/s11033-020-05287-9		2020-03	2021-09-07 15:1	2021-12-07 20:1	2021-09-07 15:1	1905-1920		3	47		Mol Biol Rep								en		
TLT7PR3U	journalArticle	2020	Condon, Laura E	Evapotranspiratio	Nature Communications		2041-1723	10.1038/s41467-	http://www.nature	Abstract A warmer climate increases evaporative demand. However, response to warming depends on wate	2020-12	2021-09-07 15:1	2021-12-07 20:1	2021-09-07 15:1	873		1	11		Nat Commun								en		
JWZGBDFJ	report	2020	Miao, Chenyong;	Functional principal component based time-series genome-wide association in sorghum					http://biorxiv.org/	ABSTRACT The phenotypes of plants develop over time and change in response to the environment. New e	2020-02-17	2021-09-07 15:1	2021-09-07 15:1	2021-09-07 15:1												Plant Biology		en		preprint
6RJBW59G	report	2020	Chen, Lin-Xing; M	Large Freshwater Phages with the Potential to Augment Aerobic Methane Oxidation					http://biorxiv.org/	Abstract There is growing evidence that phages with unusually large genomes are common across v	2020-02-14	2021-09-07 15:1	2021-09-07 15:1	2021-09-07 15:1												Microbiology		en		preprint
G9VXQ2MF	journalArticle	2020	Gallagher, Racha	Open Science pr	Nature Ecology & Evolution		2397-334X	10.1038/s41559-	http://www.nature.com/articles/s41559-020-1109-6		2020-03-01	2021-09-07 15:1	2021-09-07 15:1	2021-09-07 15:1	294-303		3	4		Nat Ecol Evol								en		
5TITM8F9	document	2020	McArdle, Daniel;	Self-healing architecture for resilient computing services							2020-02-04	2021-09-07 15:1	2021-09-07 15:1													Google Patents				
6BCCCCJB	conferencePaper	2020	Nguyen, Minh; D	Security-aware R	Proceedings of th	978-1-4503-7751-5		10.1145/336974(	https://dl.acm.org/doi/10.1145/3369740.3369791		2020-01-04	2021-09-07 15:1	2021-09-07 15:1	2021-09-07 15:1	1-10											ACM	Kolkata India	en		
59R83ZK3	journalArticle	2020	Hannah, Lee; Ro	30% land conser	Ecography		0906-7590, 1600	10.1111/ecog.05	https://onlinelibrary.wiley.com/doi/10.1111/ecog.05166		2020-07	2021-09-07 15:1	2021-12-07 20:1	2021-09-07 15:1	943-953		7	43		Ecography								en		
IBLQHFQ6	journalArticle	2020	Taylor, Louis J; A	grabseqs: simple	Bioinformatics		1367-4803, 1460	10.1093/bioinfor	https://academic.	Abstract Summary High-throughput sequencing is a powerful technique for addressing bi	2020-06-01	2021-09-07 15:1	2021-09-07 15:1	2021-09-07 15:1	3607-3609		11	36			grabseqs							en		
IICNR2R5	journalArticle	2020	Francisco Ribeiro	Emerging Advanc	Plants		2223-7747	10.3390/plants90	https://www.mdpi	Agriculture remains critical to Africa's socioeconomic development, employing 65% of the work force and contributi	2020-03-19	2021-09-07 15:1	2021-09-07 15:1	2021-09-07 15:1	381		3	9		Plants								en		
U2GJZBNG	journalArticle	2020	Shenton, Matt; K	Evolution and div	Genome Biology and Evolution		1759-6653	10.1093/gbe/eva	https://academic.	Abstract The Oryza officinalis complex is the largest species group in Oryza, with more than nine species fr	2020-03-03	2021-09-07 15:1	2021-09-07 15:1	2021-09-07 15:1	evaa037													en		
QLU888KS	journalArticle	2020	Danczak, R. E.;	Ecological Assem	mSystems		2379-5077	10.1128/mSyster	https://journals.a	Interactions between viral communities and their microbial hosts have been the subject of many recent studies in a	2020-04-28	2021-09-07 15:1	2021-09-07 15:1	2021-09-07 15:1			2	5		mSystems								en		
GAUHCXJX	journalArticle	2020	Doak, Thomas; S	National Center for Genome Analysis Support (NCGAS): Genomics and other Science in the NSF-Funded Jetstream Cloud							2020	2021-09-07 15:1	2021-09-07 15:1																	
XARMU8N5	journalArticle	2020	Barker, Richard;	Test of Arabidops	Frontiers in Plant Science		1664-462X	10.3389/fpls.202	https://www.frontiersin.org/article/10.3389/fpls.2020.00147/full		2020-03-04	2021-09-07 15:1	2021-12-07 20:1	2021-09-07 15:1	147			11		Front. Plant Sci.	Test of Arabidopsis Space Transcriptome									
SK83KMQZ	journalArticle	2020	Jamnadass, Ram	Enhancing Africa	Nature Genetics		1061-4036, 1546	10.1038/s41588-	http://www.nature.com/articles/s41588-020-0601-x		2020-04	2021-09-07 15:1	2021-12-07 20:1	2021-09-07 15:1	356-360		4	52		Nat Genet								en		
CQ9G95WE	journalArticle	2020	Tsiola, Anastasia	Viral Metagenom	Viruses						2020	2021-09-07 15:1	2021-10-14 0:26		806		8	12												
IAKD6CJ4	journalArticle	2020	Lutsik, Pavlo; Ba	Globally altered e	Nature Communications		2041-1723	10.1038/s41467-	http://www.nature	Abstract The neoplastic stromal cells of giant cell tumor of bone (GCTB) carry a mutation in	2020-12	2021-09-07 15:1	2021-12-07 20:1	2021-09-07 15:1	5414		1	11		Nat Commun								en		
89PLKCRJ	journalArticle	2020	Tirado, Sara	Characterizing the impact of genetic and environmental variation on maize utilizing phe					https://conservan	To gain a better understanding of how genotypic elements interact with the environment, we need to develop more	2020	2021-09-07 15:1	2021-09-07 15:1																	
B4T6PX7G	thesis	2020	Dunkel, Friedrich	A Phase-Field Approach to Diffusion-Driven Fracture					https://digitalcom	In recent years applied mathematicians have used modern analysis to develop vari- ational phase-field models of f	2020	2021-09-07 15:1	2021-10-14 0:22																	
T3LB9MGK	journalArticle	2020	Navale, Vivek; Ji	Development of a	F1000Research		2046-1402	10.12688/f1000re	https://f1000rese	The Biomedical Research Informatics Computing System (BRICS) was developed to support multiple disease-focu	2020-07-13	2021-09-07 15:1	2021-12-03 18:5	2021-09-07 15:1	1430			8		F1000Res								en		
DGSI9S3J	thesis	2020	Choi, Illyoung	Remote Data Access in Scientific Computing					http://ezproxy.libr	Remote data access is becoming increasingly common in scientific computing. The trends toward collaborative res	2020	2021-09-07 15:1	2021-10-14 0:17													The University of Arizona				PhD Thesis
SPASM6HU	thesis	2020	Scarbrough, Dan	Identification and Characterization of Candidate Staphylococcus aureus Antigens for Inclusion in a Multivalent Bovine Mastitis Vaccine							2020	2021-09-07 15:1	2021-10-14 0:31																	
FVZW3QMR	conferencePaper	2020	Zeng, Shuai; Skr	SNPViz v2.0: A w	2020 IEEE Intern	978-1-72816-215-7		10.1109/BIBM49	https://ieeexplore.ieee.org/document/9313539/		2020-12-16	2021-09-07 15:1	2021-10-14 0:25	2021-09-07 15:1	1408-1415						SNPViz v2.0					IEEE	Seoul, Korea (South)			
YDCI363B	thesis	2020	Miao, Chenyong	Quantitative Genetics and Phenomics in Crops Using Statistical and Machine Learning Approaches							2020	2021-09-07 15:1	2021-10-14 0:25													The University of Nebraska-Lincoln				PhD Thesis
N6BAGD4J	journalArticle	2020	Horvath, David P	A New Diversity I	Agronomy		2073-4395	10.3390/agronon	https://www.mdpi	A diverse population (429 member) of canola (Brassica napus L.) consisting primarily of winter biotypes was assem	2020-12-20	2021-09-07 15:1	2021-10-14 0:22	2021-09-07 15:1	2006		12	10		Agronomy								en		
7YVILMZM	journalArticle	2020	goyal, Shreya; P	Enabling Secure and Effective Biomedical Data Sharing through Cybe				10.17605/OSF.IO	https://osf.io/6y8wg/		2020	2021-09-07 15:1	2021-10-14 0:18	2021-09-07 15:1															Creative Commons Attribution 4.0	
YAE5XLTH	thesis	2020	Alawadi, Ayad A	Effect of Western Diet and Caloric Substitution with Apple Pomace on Fatty Acid Composition and Gene Expression in the Hypothalamus of Growing Female Sprague-Dawley Rats							2020	2021-09-07 15:2	2021-10-14 0:16													West Virginia University				PhD Thesis
43GTGF3X	journalArticle	2020	Martinez, Ciera C	Spatial transcript	The Plant Cell		1532-298X	10.1093/plcell/ko	https://academic.	Abstract Leaf morphogenesis involves cell division, expansion, and differentiation in the developing leaf, wh	2020-12-02	2021-09-07 15:2	2021-10-14 0:25	2021-09-07 15:2	koaa012													en		
JQY2CJTT	journalArticle	2020	Reicher, Andreas	Pooled protein ta	Genome Research		1088-9051, 1549	10.1101/gr.2615(	http://genome.cshlp.org/lookup/doi/10.1101/gr.261503.120		2020-12	2021-09-07 15:2	2021-12-07 20:1	2021-09-07 15:2	1846-1855		12	30		Genome Res.								en		
F9FQTI84	journalArticle	2020	Cimen, Emre; Je	Building a tRNA t	Nucleic Acids Research		0305-1048, 1362	10.1093/nar/gkaa	https://academic.	Abstract Because ambient temperature affects biochemical reactions, organisms living in extreme temperat	2020-12-02	2021-09-07 15:2	2021-10-14 0:17	2021-09-07 15:2	12004-12015		21	48										en		
7VYUAYH2	journalArticle	2020	Yadav, Chanchal	Draft genome as	Data in Brief		23523409	10.1016/j.dib.202	https://linkinghub.elsevier.com/retrieve/pii/S235234092031413X		2020-12	2021-09-07 15:2	2021-12-07 20:1	2021-09-07 15:2	106531			33		Data in Brief	Draft genome assembly and annotation of the masked birch caterpillar, Drepana arcuata (Lepidoptera							en		
CWXZTJID	journalArticle	2020	Helmsauer, Kons	Enhancer hijacki	Nature Communications		2041-1723	10.1038/s41467-	http://www.nature	Abstract MYCN amplification drives one in six cases of neuroblastoma. The supernumera	2020-12	2021-09-07 15:2	2021-12-07 20:1	2021-09-07 15:2	5823		1	11		Nat Commun								en		
P2LIDQ2Q	thesis	2020	Yadav, Chanchal	Sociality in caterpillars: Investigations into the mechanisms associated with grouping behaviour, from vibroacoustics to sociogenomics							2020	2021-09-07 15:2	2021-10-14 0:25													Carleton University				PhD Thesis
UYAMNEK5	journalArticle	2020	Vekaria, Komal; (	Recommender-a	Concurrency and Computation: Pr		1532-0626, 1532	10.1002/cpe.608	https://onlinelibrary.wiley.com/doi/10.1002/cpe.6080		2020-11-11	2021-09-07 15:2	2021-10-14 0:25	2021-09-07 15:2						Concurrency Computat Pract Exper								en		
P4RZB7JU	journalArticle	2020	Hevroni, Gur; Flo	Seasonal and die	Proceedings of the National Acade		0027-8424, 1091	10.1073/pnas.20	http://www.pnas.	Virus–microbe interactions have been studied in great molecular details for many years in cultured model systems,	2020-11-24	2021-09-07 15:2	2021-10-14 0:25	2021-09-07 15:2	29738-29747		47	117		Proc Natl Acad Sci USA								en		
DDDMXY6H	book	2020	Risco-Martín, Jos	Simulation for cyber-physical syste		978-3-030-51909-4			https://doi.org/10	This comprehensive book examines a range of examples, prepared by a diverse group of academic and industry p	2020	2021-09-07 15:2	2021-09-07 15:2	2021-09-07 15:2							Simulation for cyber-physical systems engineering							English		
UU27XB6T	journalArticle	2020	Bessell, Travis; F	PHANTOM ECHOES: A Five-Eyes SDA Experiment to Examine GEO Rendezvous and Proximity Operations Simon George, Andrew Ash Defence Science & Technology Laboratory, UK							2020	2021-09-07 15:2	2021-10-14 0:16																	
LSGU2DRE	thesis	2020	Grover, Jeffrey W	Investigating the Role of RNA-Directed DNA Methylation in Plant Reproduction and Development of Computational Resources Enabling Epigenomics Research							2020	2021-09-07 15:2	2021-10-14 0:18													The University of Arizona				PhD Thesis
H7T47DZU	journalArticle	2020	Ponsero, Alise; B	Ten simple rules	PLOS Computational Biology		1553-7358	10.1371/journal.p	https://dx.plos.org/10.1371/journal.pcbi.1008226		2020-10-22	2021-09-07 15:2	2021-10-14 0:26	2021-09-07 15:2	e1008226		10	16		PLoS Comput Biol								en		
N7VCMIEQ	journalArticle	2020	Mayer, Manfred;	Discovery of ben	Nature Communications		2041-1723	10.1038/s41467-	http://www.nature	Abstract Genetic variation is of crucial importance for crop improvement. Landraces are valuable sources o	2020-12	2021-09-07 15:2	2021-12-07 20:1	2021-09-07 15:2	4954		1	11		Nat Commun								en		
6GXIMGKZ	journalArticle	2020	Jin, Xingyue; Ho	Genome-Wide Id	Tropical Plant Biology		1935-9756, 1935	10.1007/s12042-	http://link.springer.com/10.1007/s12042-020-09269-w		2020-12	2021-09-07 15:2	2021-12-07 20:1	2021-09-07 15:2	371-381		4	13		Tropical Plant Biol.								en		
MVIW2DII	journalArticle	2020	Arya, Sagar S.; F	Next-generation	Biotechnology Advances		7349750	10.1016/j.biotech	https://linkinghub.elsevier.com/retrieve/pii/S0734975020301373		2020-12	2021-09-07 15:2	2021-12-07 20:1	2021-09-07 15:2	107635			45		Biotechnology Advances								en		
Z8F5PU6X	journalArticle	2020	Wright, April M.;	The why, when, a	F1000Research		2046-1402	10.12688/f1000re	https://f1000rese	Many biologists are interested in teaching computing skills or using computing in the classroom, despite not being	2020-03-02	2021-09-07 15:2	2021-12-07 20:1	2021-09-07 15:2	1854			8		F1000Res								en		
BJJF2E9U	journalArticle	2020	Jennings, Emma	Nr4a1 and Nr4a3	Cell Reports		22111247	10.1016/j.celrep.	https://linkinghub.elsevier.com/retrieve/pii/S2211124720313176		2020-11	2021-09-07 15:2	2021-12-07 20:1	2021-09-07 15:2	108328		5	33		Cell Reports								en		
8N8D2ZX6	conferencePaper	2020	Giatrakos, Nikos;	INforE: Interactiv	Proceedings of th	978-1-4503-6859-9		10.1145/3340531	https://dl.acm.org/doi/10.1145/3340531.3417435		2020-10-19	2021-09-07 15:2	2021-10-14 0:18	2021-09-07 15:2	3389-3392						INforE					ACM	Virtual Event Irela	en		
Y3574TVB	journalArticle	2020	Psaltis, Dimitrios	Gravitational Tes	Physical Review Letters		0031-9007, 1079	10.1103/PhysRev	https://link.aps.org/doi/10.1103/PhysRevLett.125.141104		2020-10-01	2021-09-07 15:2	2021-12-07 20:1	2021-09-07 15:2	141104		14	125		Phys. Rev. Lett.								en		
BGBXUZIS	journalArticle	2020	Pistorius, Julian L	Exosphere -- Brir	arXiv:2008.10640 [cs]				http://arxiv.org/ab	Exosphere provides researcher-friendly software for managing computing workloads on OpenStack cloud infrastru	2020-10-13	2021-09-07 15:2	2021-10-14 0:23	2021-09-07 15:2																
GE3SHEEJ	journalArticle	2020	Heck, Michelle; N	Proteomics in No	Journal of Proteome Research		1535-3893, 1535	10.1021/acs.jpro	https://pubs.acs.org/doi/10.1021/acs.jproteome.0c00448		2020-09-04	2021-09-07 15:2	2021-10-14 0:21	2021-09-07 15:2	3595-3606		9	19		J. Proteome Res	Proteomics in Non-model Organisms							en		
YDHXMY5U	journalArticle	2020	Tucker, Jamila S.	The Role of Testo	Frontiers in Microbiology		1664-302X	10.3389/fmicb.20	https://www.frontiersin.org/article/10.3389/fmicb.2020.01921/full		2020-08-13	2021-09-07 15:2	2021-12-07 20:1	2021-09-07 15:2	1921			11		Front. Microbiol.										
Q4VJP72B	journalArticle	2020	Arifuzzaman, Mu	Transcriptome Ar	Journal of Plant Biology		1226-9239, 1867	10.1007/s12374-	https://link.springer.com/10.1007/s12374-020-09270-6		2020-08-04	2021-09-07 15:2	2021-10-14 0:16	2021-09-07 15:2						J. Plant Biol.								en		
6385YL5B	journalArticle	2020	Panwar, Pratibha	Influence of the p	Microbiome		2049-2618	10.1186/s40168-	https://microbiom	Abstract Background Cold environments dominate the Earth's biosphere and microbial ac	2020-12	2021-09-07 15:2	2021-12-07 20:1	2021-09-07 15:2	116		1	8		Microbiome								en		
DKDRXVR9	thesis	2020	Gough, Caren	Vertically Aligned Professional Learning in the New Biology							2020	2021-09-07 15:2	2021-10-14 0:18													State University of New York at Stony Brook				PhD Thesis
8BX44V2R	journalArticle	2020	Lambarey, Huma	Transcriptomic A	Plants		2223-7747	10.3390/plants90	https://www.mdpi	Maize (Zea mays L.) is a staple crop providing food security to millions of people in sub Saharan Africa. Fusarium v	2020-08-28	2021-09-07 15:2	2021-10-14 0:26	2021-09-07 15:2	1112		9	9		Plants								en		
QHA8WE3B	journalArticle	2020	Craven, Dylan; S	A cross-scale as	Global Ecology and Biogeography		1466-822X, 1466	10.1111/geb.131(	https://onlinelibrary.wiley.com/doi/10.1111/geb.13165		2020-11	2021-09-07 15:2	2021-12-07 20:1	2021-09-07 15:2	1940-1955		11	29		Global Ecol. Biogeogr.								en		
JTUKYCY9	journalArticle	2020	Rush, Tomás Alle	Lipo-chitooligosa	Nature Communications		2041-1723	10.1038/s41467-	https://www.natu	Abstract Lipo-chitooligosaccharides (LCOs) are signaling molecules produced by rhizobial bacteria that trig	2020-12	2021-09-07 15:2	2021-12-07 20:1	2021-09-07 15:2	3897		1	11		Nat Commun								en		
JDWWDJY4	journalArticle	2020	Zhang, Ruifei; Gu	Diverse alkaloids	Fitoterapia		0367326X	10.1016/j.fitote.2	https://linkinghub.elsevier.com/retrieve/pii/S0367326X20302793		2020-10	2021-09-07 15:2	2021-12-07 20:1	2021-09-07 15:2	104697			146		Fitoterapia	Diverse alkaloids and biological activities of Fumaria (Papaveraceae)							en		
UPWEGKQI	conferencePaper	2020	Esteva, Maria; X	Modeling Data C	Proceedings of th	978-1-4503-7585-6		10.1145/3383583	https://dl.acm.org/doi/10.1145/3383583.3398539		2020-08	2021-09-07 15:2	2021-12-07 20:1	2021-09-07 15:2	235-242						Modeling Data Curation to Scientific Inquiry					ACM	Virtual Event Chi	en		
3CT5QN67	conferencePaper	2020	Neuefeind, Claes	Sustainability Str	https://dh2020. adho. org/						2020	2021-09-07 15:2	2021-09-07 15:2													DH2020				
SCMGQXMP	journalArticle	2020	Ezebialu, Chinen	Screening and C	Advances in Microbiology		2165-3402, 2165	10.4236/aim.202	https://www.scirp.org/journal/doi.aspx?doi=10.4236/aim.2020.109031		2020	2021-09-07 15:2	2021-10-14 0:18	2021-09-07 15:2	422-433		9	10		AiM										
R8C3PEZY	journalArticle	2020	Woodburn, Matt;	Unity in Variety: [	Biodiversity Information Science ar		2535-0897	10.3897/biss.4.5	https://biss.pens	Digitisation and publication of museum specimen data is happening worldwide, but far from complete. Museums ca	2020-10-09	2021-09-07 15:2	2021-10-14 0:26	2021-09-07 15:2	e59233			4		BISS	Unity in Variety									
SM5M47EM	journalArticle	2020	Aktar, Rumana;	Robust mosaickir	Applications in Plant Sciences		2168-0450, 2168	10.1002/aps3.11	https://onlinelibrary.wiley.com/doi/10.1002/aps3.11387		2020-08	2021-09-07 15:2	2021-12-07 20:1	2021-09-07 15:2			8	8		Appl Plant Sci								en		
B59WU574	journalArticle	2020	Merritt, Emily F.;	Transcriptional P	mSphere		2379-5042	10.1128/mSpher	https://journals.a	Like other persistent intracellular pathogens, Toxoplasma gondii , a protozoan parasite, has evolv	2020-10-28	2021-09-07 15:2	2021-10-14 0:26	2021-09-07 15:2			5	5		mSphere								en		
59QVVT5F	journalArticle	2020	Studstill, Caleb J	Sphingosine kina	Journal of Clinical Investigation		0021-9738, 1558	10.1172/JCI1252	https://www.jci.org/articles/view/125297		2020-11-09	2021-09-07 15:2	2021-12-07 20:1	2021-09-07 15:2	6523-6538		12	130										en		
W2AYC68N	journalArticle	2020	Flynn, Logan; Pa	Creating a phenc	Journal of Emerging Investigators				https://www.rese	In this study, we aimed to determine whether the life cycle stages, or phenophases, of some plants in the urban en	2020-07-16	2021-09-07 15:2	2021-12-03 18:5					2												
6VNYM4SF	journalArticle	2020	Wang, Lilong; Zh	Is salinity the ma	BMC Plant Biology		1471-2229	10.1186/s12870-	https://bmcplantb	Abstract Background Soil salinity is a major abiotic constraint to plant growth and develop	2020-12	2021-09-07 16:2	2021-12-07 20:1	2021-09-07 16:2	461		1	20		BMC Plant Biol								en		
DQG73UDP	journalArticle	2020	Yu, Xiaolei; Jiang	Deciphering the (	International Journal of Biological M		1418130	10.1016/j.ijbioma	https://linkinghub.elsevier.com/retrieve/pii/S0141813020347073		2020-12	2021-09-07 16:2	2021-12-07 20:1	2021-09-07 16:2	1988-1999			165		International Journal of Biological Macromolecules								en		
XGF7JDX3	journalArticle	2020	Luan, Fei; Zeng,	Recent advances	Journal of Functional Foods		17564646	10.1016/j.jff.2020	https://linkinghub.elsevier.com/retrieve/pii/S1756464620304667		2020-12	2021-09-07 16:2	2021-12-07 20:1	2021-09-07 16:2	104242			75		Journal of Functi	Recent advances in Camellia oleifera Abel							en		
MASSNNBX	journalArticle	2020	Wang, Hongfeng	Mapping Asia Pla	Global Ecology and Conservation		23519894	10.1016/j.gecco.	https://linkinghub.elsevier.com/retrieve/pii/S2351989420308623		2020-12	2021-09-07 16:2	2021-12-07 20:1	2021-09-07 16:2	e01321			24		Global Ecology a	Mapping Asia Plants							en		
Q3JZN675	journalArticle	2020	Parry, Geraint; B	How to build an e	Journal of Experimental Botany		0022-0957, 1460	10.1093/jxb/eraa	https://academic.	Abstract Successful collaborative research is dependent on excellent ideas and innovative experimental ap	2020-12-31	2021-09-07 16:2	2021-10-14 0:24	2021-09-07 16:2	6881-6889		22	71			How to build an effective research network							en		
4TU8LNPV	journalArticle	2020	Tuttle, Matthew J	Lysogeny in the (	Environmental Microbiology		1462-2912, 1462	10.1111/1462-29	https://onlinelibrary.wiley.com/doi/10.1111/1462-2920.15233		2020-12	2021-09-07 16:2	2021-12-07 20:1	2021-09-07 16:2	4919-4933		12	22		Environ Microbio	Lysogeny in the oceans							en		
8V6P6MHD	journalArticle	2020	Bekkering, Cody	Image-Based, Or	Agronomy		2073-4395	10.3390/agronon	https://www.mdpi	Wheat was one of the first grain crops domesticated by humans and remains among the major contributors to the g	2020-08-30	2021-09-07 16:2	2021-10-14 0:16	2021-09-07 16:2	1287		9	10		Agronomy								en		
JZZ48CUF	journalArticle	2020	Cheng, Longjie;	Characterization	Mitochondrial DNA Part B		2380-2359	10.1080/2380235	https://www.tandfonline.com/doi/full/10.1080/23802359.2020.1815603		2020-07-02	2021-09-07 16:2	2021-09-07 16:2	2021-09-07 16:2	3318-3319		3	5		Mitochondrial DNA Part B								en		
8M4U263U	journalArticle	2020	Debauche, Olivie	A new Collaborat	Procedia Computer Science		18770509	10.1016/j.procs.2	https://linkinghub.elsevier.com/retrieve/pii/S1877050920323309		2020	2021-09-07 16:2	2021-10-14 0:17	2021-09-07 16:2	450-455			177		Procedia Computer Science								en		
2MADJ5EV	journalArticle	2020	Senapati, Manor	An Overview on S	International Journal of Current Mi		23197692, 23197	10.20546/ijcmas.	https://www.ijcmas.com/abstractview.php?ID=18196&vol=9-7-2020&SNo=223		2020-07-20	2021-09-07 16:2	2021-10-14 0:23	2021-09-07 16:2	1956-1964		7	9		Int.J.Curr.Microbiol.App.Sci										
JNPQW2CJ	bookSection	2020	Kee, Kerk F; Sch	Telephone intervi	Second international handbook of internet research						2020	2021-09-07 16:3	2021-10-14 0:29		351-365											Springer				
5R4W9DRP	journalArticle	2020	Malanson, Georg	Hemispheric-and	Annals of the American Association of Geographers						2020	2021-09-07 22:0	2021-09-07 22:0		1005–1021		4	110												
SN2RYPUK	journalArticle	2020	Munson-McGee,	An Uncultivated V	Journal of Virology		0022-538X, 1098	10.1128/JVI.012	https://journals.a	Here, we present evidence of the first virus found to infect Nanoarchaeota, a symbiotic archaean found in acidic ho	2020-01-17	2021-09-07 22:1	2021-09-07 22:1	2021-09-07 22:1			3	94		J Virol								en		
GMZTRTUS	journalArticle	2020	Bainard, Jillian D	Genome size and	Annals of Botany		0305-7364, 1095	10.1093/aob/mcz	https://academic.	Abstract Background and Aims Compared with other plant lineages, bryophytes have ver	2020-03-29	2021-09-07 22:1	2021-09-07 22:1	2021-09-07 22:1	543-555		4	125										en		
JYZ56K5B	journalArticle	2020	Aloisio, Jason M.	Introduced and n	Urban Ecosystems		1083-8155, 1573	10.1007/s11252-	https://link.springer.com/10.1007/s11252-020-00992-6		2020-12	2021-10-12 23:3	2021-12-07 20:2	2021-10-12 23:3	1227-1238		6	23		Urban Ecosyst								en		
DP6TXGKJ	journalArticle	2020	Anche, Mahlet T.	Temporal covaria	Theoretical and Applied Genetics		0040-5752, 1432	10.1007/s00122-	https://link.spring	Abstract Key message Heritable variation in phenotypes extracted from multi-spectral ima	2020-10	2021-10-12 23:3	2021-12-07 20:2	2021-10-12 23:3	2853-2868		10	133		Theor Appl Genet								en		
859R4C39	journalArticle	2020	Anderson, J.; Ke	High diversity of	Transactions of the Royal Society		0372-1426, 2204	10.1080/0372142	https://www.tandfonline.com/doi/full/10.1080/03721426.2020.1786779		2020-07-02	2021-10-12 23:3	2021-10-12 23:3	2021-10-12 23:3	180-199		2	144		Transactions of the Royal Society of South Australia								en		
XJQN3LMI	journalArticle	2020	Arnaud, Elizabet	The Ontologies C	Patterns		26663899	10.1016/j.patter.2	https://linkinghub.elsevier.com/retrieve/pii/S2666389920301392		2020-10	2021-10-12 23:3	2021-12-07 20:2	2021-10-12 23:3	100105		7	1		Patterns	The Ontologies Community of Practice							en		
PQ7GUA4I	journalArticle	2020	Baránková, Simo	Sex-chrom, a da	New Phytologist		0028-646X, 1469	10.1111/nph.1663	https://onlinelibrary.wiley.com/doi/10.1111/nph.16635		2020-09	2021-10-12 23:3	2021-12-07 20:2	2021-10-12 23:3	1594-1604		6	227		New Phytol								en		
PLB8QCF2	journalArticle	2020	Beckman, Noelle	Collaborative Research: Diverse selective pressure on fruit chemical t				10.26078/1EDM-	https://digitalcommons.usu.edu/funded_research_data/158		2020	2021-10-12 23:3	2021-10-12 23:3	2021-10-12 23:3							Collaborative Research									
JFGY3SXN	bookSection	2020	Bitterman, Thom	Offering Simulati	Simulation for Cy	978-3-030-51908-7 978-3-030-51909-4			http://link.springer.com/10.1007/978-3-030-51909-4_10		2020	2021-10-12 23:3	2021-10-12 23:3	2021-10-12 23:3	259-282											Springer Internat	Cham	en		
UCIUJBZW	book	2020	Kabbani, Omar K	Investigating the Regulatory Targets of the WRKY75 Transcription Factor during Age-As					https://www.proquest.com/docview/2408091828?pq-origsite=gscholar&fromopenview=true		2020	2021-10-12 23:5	2021-10-12 23:5													California State University, Long Beach				
3644Q4FE	thesis	2020	Lemert, Phebe H	Conservation of Dehydration Stress Memories Between Abiotic Stresses in Zea mays a					http://purl.flvc.org/fsu/fd/FSU_libsubv1_scholarship_submission_1588008687_cb4b5c41		2020	2021-10-12 23:5	2021-10-12 23:5																	
EC9TANN6	journalArticle	2020	Li, Po-E.; Gutiérr	A Public Website	arXiv:2006.04566 [q-bio]				http://arxiv.org/ab	Summary: Polymerase chain reaction-based assays are the current gold standard for detecting and diagnosing SA	2020-06-08	2021-10-12 23:5	2021-10-12 23:5	2021-10-12 23:5																
A42JQ75D	thesis	2020	Mutte, Sumanth I	Evolutionary analysis of a billion years of auxin biology					https://edepot.wur.nl/511001		2020	2021-10-13 0:01	2021-10-13 0:01													Wageningen University				PhD Thesis
2L93L4SM	thesis	2020	Patel, Esha	Characterization of Heavy Metal Resistance Genes from a Cryptic Plasmid of Pseudom					https://www.proquest.com/docview/2421495012?pq-origsite=gscholar&fromopenview=true		2020	2021-10-13 23:2	2021-10-13 23:2													Adelphi University				PhD Thesis
MDTN9GJF	thesis	2020	Richardson, Meg	Identifying Novel Transcriptional Effectors of the Juvenile Hormone Pathway in Aedes a					http://hdl.handle.net/10919/98538		2020	2021-10-13 23:3	2021-10-13 23:3													Virginia Tech				PhD Thesis
FQHDI2GB	bookSection	2020	Sawyer, Steve; S	Stabilizing Digital	Fields of Practice	978-0-429-45954-2					2020	2021-10-13 23:3	2021-10-13 23:3		21–36											CRC Press				
U6WUMJLR	thesis	2020	Sircar, Sanchari	Rice Stress Transcriptome Analysis–A Network Biology Approach							2020	2021-10-13 23:3	2021-10-13 23:3													International Institute of Information Technology Hyderabad				PhD Thesis
SDPZWAGS	thesis	2020	Tolosa, Sara B Ti	Characterizing the impact of genetic and environmental variation on maize utilizing phe					https://conservancy.umn.edu/bitstream/handle/11299/216817/Tirado_umn_0130E_21731.pdf?sequence=1		2020	2021-10-13 23:3	2021-10-13 23:3													University of Minnesota				PhD Thesis
STPZJS5U	thesis	2020	Williamson, Victo	Biodiversity of Microscopic Green Algae from Desert Soil Crusts					https://digitalcommons.assumption.edu/honorstheses/69		2020	2021-10-13 23:4	2021-10-13 23:4													Assumption College				Honors Thesis
SAAYVNYM	journalArticle	2020	Burnett, Karen G	Recommendatior	Integrative and Comparative Biolog		1540-7063, 1557	10.1093/icb/icaa(	https://academic.	Synopsis The 2020 SICB Society-wide Symposium "Building Bridges from Genome to Phenome: Molecules	2020-08-01	2021-10-13 23:4	2021-10-13 23:4	2021-10-13 23:4	397-401		2	60										en		
UCMEEPT2	journalArticle	2020	Campbell, Micha	Changes in gene	PLOS ONE		1932-6203	10.1371/journal.p	https://dx.plos.org/10.1371/journal.pone.0235444		2020-07-02	2021-10-13 23:4	2021-12-03 18:5	2021-10-13 23:4	e0235444		7	15		PLoS ONE								en		

ZCXJF7W6	journalArticle	2020	Castelletti, Sara;	Maize adaptation	PLOS Genetics		1553-7404	10.1371/journal.p	https://dx.plos.org/10.1371/journal.pgen.1008882		2020-07-16	2021-10-13 23:4	2021-12-03 18:5	2021-10-13 23:4	e1008882		7	16		PLoS Genet								en		
VU2UU825	journalArticle	2020	Busener, Natalie;	Image-based roo	PLANTS, PEOPLE, PLANET		2572-2611, 2572	10.1002/ppp3.10	https://onlinelibrary.wiley.com/doi/10.1002/ppp3.10130		2020-11	2021-10-13 23:4	2021-12-07 20:2	2021-10-13 23:4	678-687		6	2		Plants People Planet								en		
JJ8ZMZZN	journalArticle	2020	DeBarry, Jeremy	Practical Recomm	Data Science Journal		1683-1470	10.5334/dsj-2020	http://datascience.codata.org/articles/10.5334/dsj-2020-024/		2020-06-09	2021-10-13 23:5	2021-10-13 23:5	2021-10-13 23:5	24			19										en		
LGEV6EKP	journalArticle	2020	Dai, Xiuru; Xu, Zl	Non-homology-b	The Plant Genome		1940-3372, 1940	10.1002/tpg2.200	https://onlinelibrary.wiley.com/doi/10.1002/tpg2.20015		2020-07	2021-10-13 23:5	2021-12-07 20:2	2021-10-13 23:5			2	13		Plant Genome								en		
XKYYN69D	journalArticle	2020	Eissler, Yoanna;	Virus and Potenti	Microorganisms		2076-2607	10.3390/microorg	https://www.mdpi	Salar de Huasco is a wetland in the Andes mountains, located 3800 m above sea level at the Chilean Altiplano. He	2020-07-20	2021-10-13 23:5	2021-10-13 23:5	2021-10-13 23:5	1077		7	8		Microorganisms								en		
7NXDMDNG	book	2020		Benchmarking, Measuring, and Op		978-3-030-49555-8 978-3-030-49556-5			http://link.springer.com/10.1007/978-3-030-49556-5		2020	2021-10-13 23:5	2021-10-13 23:5	2021-10-13 23:5				12093			Benchmarking, M	Lecture Notes in Computer Science				Springer Internat	Cham	en		
MP74CQX3	journalArticle	2020	Garrison, Nicole	Shifting evolution	BMC Evolutionary Biology		1471-2148	10.1186/s12862-	https://bmcevolbi	Abstract Background Mygalomorph spiders represent a diverse, yet understud	2020-12	2021-10-13 23:5	2021-12-07 20:2	2021-10-13 23:5	68		1	20		BMC Evol Biol	Shifting evolutionary sands							en		
8T8YH8UK	journalArticle	2020	Gedir, Jay V.; Ca	Extreme drought	Ecosphere		2150-8925, 2150	10.1002/ecs2.31	https://onlinelibrary.wiley.com/doi/10.1002/ecs2.3175		2020-07	2021-10-13 23:5	2021-12-07 20:2	2021-10-13 23:5			7	11		Ecosphere								en		
LDJ5QH59	journalArticle	2020	Forsythe, Evan S	Biased Gene Ret	Genome Biology and Evolution		1759-6653	10.1093/gbe/eva	https://academic.	Abstract Phylogenomic analyses are recovering previously hidden histories of hybridization, revealing the g	2020-09-01	2021-10-13 23:5	2021-10-13 23:5	2021-10-13 23:5	1646-1663		9	12										en		
7TCBAGZM	journalArticle	2020	Giné-Bordonaba,	Investigation of th	Horticulture Research		2662-6810, 2052	10.1038/s41438-	http://www.nature	Abstract To elucidate the physiology underlying the development of superficial scald in pears, sus	2020-12	2021-10-14 0:00	2021-12-07 20:2	2021-10-14 0:00	49		1	7		Hortic Res								en		
LSV2WGLR	journalArticle	2020	Gulino, K.; Rahm	Initial Mapping of	mSystems		2379-5077	10.1128/mSyster	https://journals.a	Wastewater is a rich source of microbial life and contains bacteria, viruses, and other microbes found in human wa	2020-06-30	2021-10-14 0:00	2021-10-14 0:00	2021-10-14 0:00			3	5		mSystems								en		
VBHLL6EX	journalArticle	2020	Goller, Carlos C.;	Evolution of an 8	Biochemistry and Molecular Biolog		1470-8175, 1539	10.1002/bmb.21	https://onlinelibrary.wiley.com/doi/10.1002/bmb.21349		2020-07	2021-10-14 0:00	2021-12-07 20:2	2021-10-14 0:00	391-403		4	48		Biochem Mol Bio	Evolution of an 8-week upper-division metagenomics course							en		
ND7Q9NI6	journalArticle	2020	Graham, Linda E	Molecular marke	Phycologia		0031-8884, 2330	10.1080/0031888	https://www.tandfonline.com/doi/full/10.1080/00318884.2020.1761683		2020-07-03	2021-10-14 0:02	2021-10-14 0:02	2021-10-14 0:02	340-345		4	59		Phycologia								en		
25LCPYIQ	journalArticle	2020	Holzmeyer, Laura	Evaluation of pla	Proceedings of the National Acade		0027-8424, 1091	10.1073/pnas.19	http://www.pnas.	Antibiotic resistance and viral diseases are rising around the world and are becoming major threats to global health	2020-06-02	2021-10-14 0:03	2021-10-14 0:03	2021-10-14 0:03	12444-12451		22	117		Proc Natl Acad Sci USA								en		
DWYJG8IT	report	2020	Hejblum, Boris P.	Realistic and Robust Reproducible Research for Biostatistics					https://www.prep	The complexity of analysis pipelines in biomedical sciences poses a severe challenge for the transparency and rep	2020-06-02	2021-10-14 0:03	2021-10-14 0:03	2021-10-14 0:03												MEDICINE & PHARMACOLOGY				preprint
ZPJVDTMM	journalArticle	2020	Joyce, Elizabeth;	Checklist of the v	Biodiversity Data Journal		1314-2828, 1314	10.3897/BDJ.8.e	https://bdj.penso	Background The Sunda-Sahul Convergence Zone, defined here as the area comprising Australia, N	2020-05-18	2021-10-14 0:05	2021-10-14 0:05	2021-10-14 0:05	e51094			8		BDJ										
T99HDKH8	journalArticle	2020	Jones, Hamlyn G	What plant is tha	AoB PLANTS		2041-2851	10.1093/aobpla/	https://academic.	Abstract There has been a recent explosion in development of image recognition technology and its applica	2020-12-01	2021-10-14 0:05	2021-10-14 0:05	2021-10-14 0:05	plaa052		6	12			What plant is that?							en		
JY3FX4TE	journalArticle	2020	Hubbard, Allen; E	fRNAkenseq: a fu	PeerJ		2167-8359	10.7717/peerj.85	https://peerj.com	Background Decreasing costs make RNA sequencing technologies increasingly affordable for biologists. H	2020-05-14	2021-10-14 0:05	2021-10-14 0:05	2021-10-14 0:05	e8592			8			fRNAkenseq							en		
KY3EWMQF	journalArticle	2020	Klesse, Stefan; D	Continental-scale	Global Change Biology		1354-1013, 1365	10.1111/gcb.151	https://onlinelibrary.wiley.com/doi/10.1111/gcb.15170		2020-09	2021-10-14 0:05	2021-12-07 20:2	2021-10-14 0:05	5146-5163		9	26		Glob Change Bio	Continental-scale tree-ring-based projection of Douglas-fir growth							en		
THMDSXGJ	journalArticle	2020	Kramer, Mariann	N ⁶	Plant Direct		2475-4455, 2475	10.1002/pld3.23	https://onlinelibrary.wiley.com/doi/10.1002/pld3.239		2020-07	2021-10-14 0:05	2021-12-07 20:2	2021-10-14 0:05			7	4		Plant Direct								en		
3KXJY6CP	bookSection	2020	Jarvenpaa, Sirkk	Data Sourcing ar	Information Syste	978-3-030-45818-8 978-3-030-45819-5			http://link.springer.com/10.1007/978-3-030-45819-5_4		2020	2021-10-14 0:05	2021-10-14 0:05	2021-10-14 0:05	61-79						Data Sourcing and Data Partnerships					Springer Internat	Cham	en		
DW739KLW	journalArticle	2020	Liu, Yanhui; Chai	Genome-Wide Ar	International Journal of Genomics		2314-436X, 2314	10.1155/2020/31	https://www.hinda	This study identified 57 basic leucine zipper (bZIP) genes from the pineapple genome, and the analysis of these bZ	2020-05-11	2021-10-14 0:06	2021-10-14 0:06	2021-10-14 0:06	1-14			2020		International Journal of Genomics								en		
P4Q68JHY	journalArticle	2020	Liu, Hongyou; Za	Genetic diversity	Global Ecology and Conservation		23519894	10.1016/j.gecco.	https://linkinghub.elsevier.com/retrieve/pii/S2351989420307836		2020-12	2021-10-14 0:06	2021-12-07 20:2	2021-10-14 0:06	e01242			24		Global Ecology and Conservation								en		
RNUCFLUR	journalArticle	2020	Lang, Konrad; St	CyVerse Austria–	Mathematical and Computational A		2297-8747	10.3390/mca250	https://www.mdpi	Life sciences (LS) are advanced in research data management, since LS have established disciplinary tools for dat	2020-06-24	2021-10-14 0:07	2021-10-14 0:07	2021-10-14 0:07	38		2	25		MCA								en		
88A8FJ7V	journalArticle	2020	Moon, Kira; Kim,	Viral metagenom	Scientific Data		2052-4463	10.1038/s41597-	https://www.natu	Abstract A high number of viral metagenomes have revealed countless genomes of putative bacteriophages	2020-12	2021-10-14 0:08	2021-12-07 20:2	2021-10-14 0:08	349		1	7		Sci Data								en		
Q9VA5ZL2	journalArticle	2020	Metegnier, Gabri	Species specific	Scientific Reports		2045-2322	10.1038/s41598-	http://www.nature	Abstract Harmful algal blooms are caused by specific members of microbial communities. Unders	2020-12	2021-10-14 0:08	2021-12-07 20:2	2021-10-14 0:08	6182		1	10		Sci Rep								en		
M4SRPA8A	journalArticle	2020	Mukherjee, Shra	Quiescent stem c	Scientific Reports		2045-2322	10.1038/s41598-	http://www.nature	Abstract Grade 4 glioma or GBM has poor prognosis and is the most aggressive grade of glioma. Accurate	2020-12	2021-10-14 0:08	2021-12-07 20:2	2021-10-14 0:08	10937		1	10		Sci Rep								en		
QRZIEEP3	journalArticle	2020	Mutte, Sumanth;	High-resolution a	BIO-PROTOCOL		2331-8325	10.21769/BioPro	https://bio-protocol.org/e3566		2020	2021-10-14 0:08	2021-10-14 0:08	2021-10-14 0:08			6	10		BIO-PROTOCOL								en		
27BHLNC9	journalArticle	2020	Neuefeind, Claes	Sustainability Strategies for Digital Humanities Systems				10.17613/NCDT-	https://hcommon	Now that the Digital Humanities (DH) are becoming a well-established research field, producing seminal publicatior	2020	2021-10-14 0:08	2021-10-14 0:08	2021-10-14 0:08														en	Creative Commons Attribution Non	
MKMNZCYD	journalArticle	2020	Ng, Ashley P.; Co	An Erg-driven tra	Nature Communications		2041-1723	10.1038/s41467-	http://www.nature	Abstract B lymphoid development is initiated by the differentiation of hematopoietic stem cells into	2020-12	2021-10-14 0:08	2021-12-07 20:2	2021-10-14 0:08	3013		1	11		Nat Commun								en		
NDB42LUD	journalArticle	2020	Pomerantz, Mark	Prostate cancer r	Nature Genetics		1061-4036, 1546	10.1038/s41588-	http://www.nature.com/articles/s41588-020-0664-8		2020-08	2021-10-14 0:10	2021-12-07 20:2	2021-10-14 0:10	790-799		8	52		Nat Genet								en		
I85UHWVY	journalArticle	2020	Pinho Morais, Pe	Using public data	Plant Breeding		0179-9541, 1439	10.1111/pbr.1282	https://onlinelibrary.wiley.com/doi/10.1111/pbr.12827		2020-08	2021-10-14 0:10	2021-12-07 20:2	2021-10-14 0:10	697-707		4	139		Plant Breed								en		
86P2NT2I	journalArticle	2020	Ryder, Elizabeth	Incubators: Build	Biochemistry and Molecular Biolog		1470-8175, 1539	10.1002/bmb.21	https://onlinelibrary.wiley.com/doi/10.1002/bmb.21387		2020-07	2021-10-14 0:10	2021-12-07 20:2	2021-10-14 0:10	381-390		4	48		Biochem Mol Bio	Incubators							en		
IAV5VLPW	journalArticle	2020	Sharma, Manish;	Cyclic GMP-AMP	Proceedings of the National Acade		0027-8424, 1091	10.1073/pnas.20	http://www.pnas.	Huntington disease (HD) is caused by an expansion mutation of the N-terminal polyglutamine of huntingtin (mHTT)	2020-07-07	2021-10-14 0:10	2021-10-14 0:10	2021-10-14 0:10	15989-15999		27	117		Proc Natl Acad Sci USA								en		
I8JPK9LI	journalArticle	2020	Shaw, Felix; Etuk	COPO: a metada	F1000Research		2046-1402	10.12688/f1000re	https://f1000rese	Scientific innovation is increasingly reliant on data and computational resources. Much of today's life science resea	2020-06-02	2021-10-14 0:10	2021-12-07 20:2	2021-10-14 0:10	495			9		F1000Res	COPO							en		
NVJ24Q6F	journalArticle	2020	Salungyu, Jirawa	From lab to field:	Field Crops Research		3784290	10.1016/j.fcr.202	https://linkinghub.elsevier.com/retrieve/pii/S0378429020302884		2020-09	2021-10-14 0:10	2021-12-07 20:2	2021-10-14 0:10	107872			255		Field Crops Rese	From lab to field							en		
RW4X5RCT	journalArticle	2020	Voichek, Yoav; W	Identifying geneti	Nature Genetics		1061-4036, 1546	10.1038/s41588-	http://www.nature.com/articles/s41588-020-0612-7		2020-05	2021-10-14 0:12	2021-12-07 20:2	2021-10-14 0:12	534-540		5	52		Nat Genet								en		
EA5Z6ZFU	journalArticle	2020	Van Dolah, Franc	Transcriptomic ar	PLOS ONE		1932-6203	10.1371/journal.p	https://dx.plos.org/10.1371/journal.pone.0231400		2020-04-15	2021-10-14 0:12	2021-12-07 20:2	2021-10-14 0:12	e0231400		4	15		PLoS ONE								en		
HFPUMKMP	journalArticle	2020	Von Suchodoletz	Lessons learned	E-Science-Tage 2019			10.11588/HEIBO	https://books.ub.uni-heidelberg.de/index.php/heibooks/catalog/book/598/c8418		2020	2021-10-14 0:12	2021-10-14 0:12	2021-10-14 0:12														en		
YKFEFSLV	journalArticle	2020	Ude, George N.;	DNA barcoding fo	Conservation Genetics Resources		1877-7252, 1877	10.1007/s12686-	https://link.spring	Abstract Within Enugu and Anambra States, Nigeria, identification of fishes has been based on m	2020-12	2021-10-14 0:12	2021-12-07 20:2	2021-10-14 0:12	643-658		4	12		Conservation Genet Resour								en		
6RUKR8ZW	journalArticle	2020	Xie, Yulong; Wu,	Early Eocene sou	Global and Planetary Change		9218181	10.1016/j.gloplac	https://linkinghub.elsevier.com/retrieve/pii/S0921818120302113		2020-12	2021-10-14 0:13	2021-12-07 20:2	2021-10-14 0:13	103320			195		Global and Plane	Early Eocene southern China dominated by desert							en		
F6X9NUUX	journalArticle	2020	Wu, Shuang; Zho	Diverse and uniq	BMC Genomics		1471-2164	10.1186/s12864-	https://bmcgenor	Abstract Background Viruses are the most abundant biological entities on earth and play	2020-12	2021-10-14 0:13	2021-12-07 20:2	2021-10-14 0:13	441		1	21		BMC Genomics								en		
NGP4E6IK	journalArticle	2020	Yadav, Chanchal	Transcriptome ar	PLOS ONE		1932-6203	10.1371/journal.p	https://dx.plos.org/10.1371/journal.pone.0234903		2020-06-22	2021-10-14 0:13	2021-12-07 20:2	2021-10-14 0:13	e0234903		6	15		PLoS ONE	Transcriptome analysis of a social caterpillar, Drepana arcuata							en		
K8TGV6BE	report	2020	Zhang, Jun; Con	A national topographic dataset for hydrological modeling overcontiguous United States					https://essd.cope	Abstract. Topography is a fundamental input to hydrologic models critical for generating realistic streamflow networ	2020-11-21	2021-10-14 0:14	2021-10-14 0:14	2021-10-14 0:14												Hydrology and Soil Science – Hydrology				preprint
KPE4I6NI	journalArticle	2020	Zhong, Zhi-Ping;	Viral Ecogenomic	mSystems		2379-5077	10.1128/mSyster	https://journals.a	This study explores viral community structure and function in remote and extreme Arctic environments, including su	2020-06-30	2021-10-14 0:14	2021-10-14 0:14	2021-10-14 0:14			3	5		mSystems								en		
SYE5AK89	journalArticle	2020	Yin, Lilin; Zhang,	KAML: improving	Genome Biology		1474-760X	10.1186/s13059-	https://genomebi	Abstract Advances in high-throughput sequencing technologies have reduced the cost of genotypi	2020-12	2021-10-14 0:14	2021-12-07 20:2	2021-10-14 0:14	146		1	21		Genome Biol	KAML							en		
8HNNZD8N	report	2020	Zurawski, Jason;	University of Wisconsin-Madison Campus-Wide Deep Dive					https://www.osti.gov/servlets/purl/1631674/		2020-05-26	2021-10-14 0:15	2021-10-14 0:15	2021-10-14 0:15	LBNL--2001325, 1631674, ark:/13030/qt99c4v5xh															
C33ALPPD	journalArticle	2019	Chiovitti, Anthony	A citizen science	PLOS ONE		1932-6203	10.1371/journal.p	https://dx.plos.org/10.1371/journal.pone.0208604		2019-01-10	2021-09-07 16:3	2021-12-07 20:3	2021-09-07 16:3	e0208604		1	14		PLoS ONE								en		
YL2AZJK5	journalArticle	2019	Bischoff, Vera; Bi	Cobaviruses – a	The ISME Journal		1751-7362, 1751	10.1038/s41396-	http://www.nature.com/articles/s41396-019-0362-7		2019-06	2021-09-07 16:3	2021-12-07 20:3	2021-09-07 16:3	1404-1421		6	13		ISME J								en		
8YBH9W34	journalArticle	2019	Beedessee, Giris	Diversified secon	Scientific Reports		2045-2322	10.1038/s41598-	http://www.nature.com/articles/s41598-018-37792-0		2019-12	2021-09-07 16:3	2021-12-07 20:3	2021-09-07 16:3	1204		1	9		Sci Rep								en		
XDY89YXM	journalArticle	2019	Bolger, Anthony I	Computational as	The Plant Journal		9607412	10.1111/tpj.1417	https://onlinelibrary.wiley.com/doi/10.1111/tpj.14179		2019-01	2021-09-07 16:3	2021-12-07 20:3	2021-09-07 16:3	182-198		1	97		Plant J								en		
I2XN527D	journalArticle	2019	Brahmstedt, Evie	Assessment of m	Journal of Great Lakes Research		3801330	10.1016/j.jglr.201	https://linkinghub.elsevier.com/retrieve/pii/S0380133019300401		2019-08	2021-09-07 16:3	2021-12-07 20:3	2021-09-07 16:3	735-741		4	45		Journal of Great Lakes Research								en		
W8UBVJPW	journalArticle	2019	Calkins, Travis L.	GPCR annotation	General and Comparative Endocri		166480	10.1016/j.ygcen.	https://linkinghub.elsevier.com/retrieve/pii/S001664801830306X		2019-07	2021-09-07 16:3	2021-12-07 20:3	2021-09-07 16:3	89-103			278		General and Con	GPCR annotation, G proteins, and transcriptomics of fire ant (Solenopsis invicta) queen and worker brain							en		
LJZRA5QB	journalArticle	2019	Choi, Won-Gyu;	Variation in the tr	American Journal of Botany		29122	10.1002/ajb2.122	https://onlinelibrary.wiley.com/doi/10.1002/ajb2.1223		2019-01	2021-09-07 16:3	2021-12-07 20:3	2021-09-07 16:3	123-136		1	106		Am J Bot								en		
H9Q7CQXJ	journalArticle	2019	Bhat, Nadiem Na	Compendium of	Plant Gene		23524073	10.1016/j.plgene	https://linkinghub.elsevier.com/retrieve/pii/S2352407318300301		2019-03	2021-09-07 16:3	2021-12-07 20:3	2021-09-07 16:3	100166			17		Plant Gene								en		
ASVPWEAU	journalArticle	2019	Choi, Hong-Kyu	Translational gen	Genes & Genomics		1976-9571, 2092	10.1007/s13258-	http://link.springer.com/10.1007/s13258-018-0751-8		2019-02	2021-09-07 16:3	2021-12-07 20:3	2021-09-07 16:3	133-146		2	41		Genes Genom								en		
9K6IT8RI	journalArticle	2019	Colle, Marivi; Lei	Haplotype-phase	GigaScience		2047-217X	10.1093/gigascie	https://academic.oup.com/gigascience/article/doi/10.1093/gigascience/giz012/5304886		2019-03-01	2021-09-07 16:3	2022-03-03 19:5	2021-09-07 16:3			3	8										en		
64W4JNQF	journalArticle	2019	Busatto, Nicola;	Wide transcriptio	Horticulture Research		2052-7276	10.1038/s41438-	http://www.nature.com/articles/s41438-018-0115-1		2019-12	2021-09-07 16:3	2021-12-07 20:3	2021-09-07 16:3	32		1	6		Hortic Res								en		
9D962EWA	journalArticle	2019	Castillo, Andreina	Tail Wags the Do	Genome Biology and Evolution		1759-6653	10.1093/gbe/evz	https://academic.oup.com/gbe/article/11/2/497/5299488		2019-02-01	2021-09-07 16:3	2021-10-14 0:16	2021-09-07 16:3	497-507		2	11			Tail Wags the Dog?							en		
NVZ932FA	journalArticle	2019	Bokros, Norbert;	Multispecies gen	BMC Bioinformatics		1471-2105	10.1186/s12859-	https://bmcbioinformatics.biomedcentral.com/articles/10.1186/s12859-019-2624-9		2019-03	2021-09-07 16:3	2021-12-07 20:3	2021-09-07 16:3	99		S2	20		BMC Bioinformatics								en		
8I2ZCBS3	journalArticle	2019	Bertioli, David J.;	The genome seq	Nature Genetics		1061-4036, 1546	10.1038/s41588-	http://www.nature.com/articles/s41588-019-0405-z		2019-05	2021-09-07 16:3	2021-12-07 20:3	2021-09-07 16:3	877-884		5	51		Nat Genet								en		
G2G7JLC4	journalArticle	2019	Bin Jang, Ho; Bo	Taxonomic assig	Nature Biotechnology		1087-0156, 1546	10.1038/s41587-	http://www.nature.com/articles/s41587-019-0100-8		2019-06	2021-09-07 16:3	2021-12-07 20:3	2021-09-07 16:3	632-639		6	37		Nat Biotechnol								en		
IIB2HJ9G	journalArticle	2019	Calegari, Patrice;	Web Portals for H	ACM Transactions on the Web		1559-1131, 1559	10.1145/3197385	https://dl.acm.org	This article addresses web interfaces for High-performance Computing (HPC) simulation software. First, it presents	2019-02-20	2021-09-07 16:3	2021-10-14 0:16	2021-09-07 16:3	1-36		1	13		ACM Trans. Web	Web Portals for High-performance Computing							en		
C52EXVF6	journalArticle	2019	Coley, Phyllis D.;	Macroevolutionar	Journal of Ecology		0022-0477, 1365	10.1111/1365-27	https://onlinelibrary.wiley.com/doi/10.1111/1365-2745.13208		2019-07	2021-09-07 16:3	2021-12-07 20:3	2021-09-07 16:3	1620-1632		4	107		J Ecol	Macroevolutionary patterns in overexpression of tyrosine							en		
MAKE87TE	journalArticle	2019	Daly, Rebecca A.	Viruses control d	Nature Microbiology		2058-5276	10.1038/s41564-	http://www.nature.com/articles/s41564-018-0312-6		2019-02	2021-09-07 16:3	2021-12-07 20:3	2021-09-07 16:3	352-361		2	4		Nat Microbiol								en		
KUEH8SQ6	journalArticle	2019	Brenskelle, Laura	Integrating herba	Applications in Plant Sciences		2168-0450, 2168	10.1002/aps3.12	https://onlinelibrary.wiley.com/doi/10.1002/aps3.1231		2019-03	2021-09-07 16:3	2021-12-07 20:3	2021-09-07 16:3			3	7		Appl Plant Sci								en		
PTT9SA8J	journalArticle	2019	Cai, Hanyang; Zh	Genome-Wide C	Tropical Plant Biology		1935-9756, 1935	10.1007/s12042-	http://link.springer.com/10.1007/s12042-019-09224-4		2019-06	2021-09-07 16:3	2021-12-07 20:3	2021-09-07 16:3	117-131		2	12		Tropical Plant Biol.								en		
ZW4MNS4B	bookSection	2019	De Ron, AM; Álva	Common bean g	Genomic Designing of Climate-Smart Pulse Crops						2019	2021-09-07 16:3	2021-10-14 0:17		1–106											Springer				
3WR5UNEJ	journalArticle	2019	Sousa, Filipe; Fo	Nuclear protein p	New Phytologist		0028-646X, 1469	10.1111/nph.155	https://onlinelibrary.wiley.com/doi/10.1111/nph.15587		2019-04	2021-09-07 16:3	2021-12-07 20:3	2021-09-07 16:3	565-575		1	222		New Phytol								en		
CWXFQ9LB	journalArticle	2019	Edger, Patrick P.;	Origin and evolut	Nature Genetics		1061-4036, 1546	10.1038/s41588-	http://www.nature.com/articles/s41588-019-0356-4		2019-03	2021-09-07 16:3	2021-12-07 20:3	2021-09-07 16:3	541-547		3	51		Nat Genet								en		
ULSEWZRF	journalArticle	2019	Debeljak, Pavla;	Microbial iron me	Environmental Microbiology		1462-2912, 1462	10.1111/1462-29	https://onlinelibrary.wiley.com/doi/10.1111/1462-2920.14621		2019-07	2021-09-07 16:3	2021-12-07 20:3	2021-09-07 16:3	2360-2374		7	21		Environ Microbiol								en		
SNCMYG39	journalArticle	2019	Deelman, Ewa; V	The Evolution of	Computing in Science & Engineeri		1521-9615, 1558	10.1109/MCSE.2	https://ieeexplore.ieee.org/document/8725518/		2019-07-01	2021-09-07 16:3	2021-12-07 20:3	2021-09-07 16:3	22-36		4	21		Comput. Sci. Eng.										
Z9RCABHZ	journalArticle	2019	Esteva, Maria; W	Identifier Service	Data and Information Management						2019	2021-09-07 16:3	2021-10-14 0:18		26–39		1	3												
4IZ8PD3G	journalArticle	2019	Fernandez-Perez	Hand2 Selectivel	Cell Reports		22111247	10.1016/j.celrep.	https://linkinghub.elsevier.com/retrieve/pii/S2211124719305571		2019-05	2021-09-07 16:3	2021-12-07 20:3	2021-09-07 16:3	2354-2369.e7		8	27		Cell Reports								en		
L8Y732NA	book	2019		Computational science - ICCS 201		978-3-030-22733-3					2019	2021-09-07 16:3	2021-09-07 16:3			659					Computational sc	Lecture notes in	11536			Springer	Cham	eng		
NHYL8YSJ	journalArticle	2019	Furler, Robert L.;	Nef-induced diffe	Virus Genes		0920-8569, 1572	10.1007/s11262-	http://link.springer.com/10.1007/s11262-019-01670-2		2019-08	2021-09-07 16:3	2021-12-07 20:3	2021-09-07 16:3	541-544		4	55		Virus Genes								en		
289YE2QF	bookSection	2019	Gardiner, Laura-J	Next-Generation	Applications of Genetic and Genomic Research in Cereals						2019	2021-09-07 16:3	2021-09-07 16:3		49–63											Elsevier				
TUGW6TJU	thesis	2019	Gillan, Jeffrey K	Rangeland Inventory and Monitoring with Unmanned Aerial System Imagery							2019	2021-09-07 16:3	2021-10-14 0:18													The University of Arizona				PhD Thesis
PGYZK5FK	journalArticle	2019	Gillan, Jeffrey K.;	Estimating Forag	Rangeland Ecology & Managemer		15507424	10.1016/j.rama.2	https://linkinghub.elsevier.com/retrieve/pii/S1550742418302914		2019-07	2021-09-07 16:3	2021-12-07 20:3	2021-09-07 16:3	575-585		4	72		Rangeland Ecology & Management								fr		
HKCIMVZN	journalArticle	2019	Hendre, Prasad S	African Orphan C	Planta		0032-0935, 1432	10.1007/s00425-	http://link.springer.com/10.1007/s00425-019-03156-9		2019-09	2021-09-07 16:3	2021-12-07 20:3	2021-09-07 16:3	989-1003		3	250		Planta	African Orphan Crops Consortium (AOCC)							en		
C2P6SHQ8	journalArticle	2019	Gruenstaeudl, Mi	EMBL2checklists	PLOS ONE		1932-6203	10.1371/journal.p	https://dx.plos.org/10.1371/journal.pone.0210347		2019-01-10	2021-09-07 16:3	2021-12-07 20:3	2021-09-07 16:3	e0210347		1	14		PLoS ONE	EMBL2checklists							en		
TD6R6XYP	journalArticle	2019	Leung, Brian; Hu	A new baseline fo	Ecological Applications		1051-0761, 1939	10.1002/eap.186	https://onlinelibrary.wiley.com/doi/10.1002/eap.1866		2019-04	2021-09-07 16:3	2021-12-07 20:3	2021-09-07 16:3			3	29		Ecol Appl	A new baseline for countrywide α-diversity and species distributions							en		
54WYA3P9	journalArticle	2019	Kong, Hyun Gi; K	The Galleria mell	Cell Reports		22111247	10.1016/j.celrep.	https://linkinghub.elsevier.com/retrieve/pii/S2211124719301809		2019-02	2021-09-07 16:3	2021-12-07 20:3	2021-09-07 16:3	2451-2464.e5		9	26		Cell Reports								en		
E7TUZCUC	journalArticle	2019	Naithani, Sushma	Involving commu	Database		1758-0463	10.1093/databas	https://academic.oup.com/database/article/doi/10.1093/database/bay146/5289625		2019-01-01	2021-09-07 16:3	2021-10-14 0:24	2021-09-07 16:3				2019										en		
XEGM5SIE	journalArticle	2019	Mejía-Guerra, Ma	A k-mer gramma	BMC Plant Biology		1471-2229	10.1186/s12870-	https://bmcplantbiol.biomedcentral.com/articles/10.1186/s12870-019-1693-2		2019-12	2021-09-07 16:3	2021-12-07 20:3	2021-09-07 16:3	103		1	19		BMC Plant Biol								en		
LKSM38RF	journalArticle	2019	Liu, Yating; Sarge	G-OnRamp: a Ga	Bioinformatics		1367-4803, 1460	10.1093/bioinfor	https://academic.	Abstract Summary G-OnRamp provides a user-friendly, web-based platform for collabora	2019-11-01	2021-09-07 16:3	2021-10-14 0:24	2021-09-07 16:3	4422-4423		21	35			G-OnRamp							en		
PAQ7YGPT	journalArticle	2019	LeFebvre, Miche	ZooArchNet: Cor	PLOS ONE		1932-6203	10.1371/journal.p	https://dx.plos.org/10.1371/journal.pone.0215369		2019-04-12	2021-09-07 16:3	2021-12-07 20:3	2021-09-07 16:3	e0215369		4	14		PLoS ONE	ZooArchNet							en		
P2VGERTR	journalArticle	2019	Nkukwana, T.T.	Global poultry pro	South African Journal of Animal Sc		2221-4062, 0375	10.4314/sajas.v4	https://www.ajol.info/index.php/sajas/article/view/181613		2019-01-09	2021-09-07 16:3	2021-10-14 0:24	2021-09-07 16:3	869		5	48		SA J. An. Sci.	Global poultry production									
DW6CQ8UU	journalArticle	2019	Necer, Abdeldjab	Vegetation heter	Botanical Sciences		2007-4476, 2007	10.17129/botsci.	http://www.botan	<p>Background: Despite its ecological importance, the study of Algerian alder forests has been	2019-03-13	2021-09-07 16:3	2021-10-14 0:26	2021-09-07 16:3	13		1	97		Bot. Sci.										
6QHBBE6R	journalArticle	2019	Ljungberg, M. Ce	Spatial distributio	Data in Brief		23523409	10.1016/j.dib.201	https://linkinghub.elsevier.com/retrieve/pii/S2352340918313672		2019-02	2021-09-07 16:3	2021-12-07 20:3	2021-09-07 16:3	365-372			22		Data in Brief								en		
359I9MZQ	journalArticle	2019	Lien, Max R.; Ba	A low-cost and o	Plant Methods		1746-4811	10.1186/s13007-	https://plantmethods.biomedcentral.com/articles/10.1186/s13007-019-0392-1		2019-12	2021-09-07 16:3	2021-12-07 20:3	2021-09-07 16:3	6		1	15		Plant Methods								en		
UWQG46UH	journalArticle	2019	Lunde, China; Ki	Tasselseed5 ove	Communications Biology		2399-3642	10.1038/s42003-	http://www.nature.com/articles/s42003-019-0354-1		2019-12	2021-09-07 16:3	2021-12-07 20:3	2021-09-07 16:3	114		1	2		Commun Biol								en		
FT7GU2HT	journalArticle	2019	Kengkanna, Jitra	Phenotypic varia	Applications in Plant Sciences		2168-0450, 2168	10.1002/aps3.12	https://onlinelibrary.wiley.com/doi/10.1002/aps3.1238		2019-04	2021-09-07 16:3	2021-12-07 20:3	2021-09-07 16:3			4	7		Appl Plant Sci								en		
PRFLR8TK	journalArticle	2019	Macías-Duarte, A	Genetic Variation	Journal of Raptor Research		0892-1016	10.3356/JRR-18-	https://bioone.org/journals/journal-of-raptor-research/volume-53/issue-2/JRR-18-00002/Genetic-Variation-among-Island-and-Contine		2019-05-09	2021-09-07 16:3	2021-12-07 20:3	2021-09-07 16:3	127		2	53		Journal of Raptor Research										
2IH65FC2	journalArticle	2019	McFadden, Ian R	Temperature sha	Ecology Letters		1461-023X, 1461	10.1111/ele.1326	https://onlinelibrary.wiley.com/doi/10.1111/ele.13269		2019-07	2021-09-07 16:3	2021-12-07 20:3	2021-09-07 16:3	1126-1135		7	22		Ecol Lett								en		
CZ4LAV7N	journalArticle	2019	Ng, Wei Lun; Wu	Comparative tran	AoB PLANTS		2041-2851	10.1093/aobpla/	https://academic.oup.com/aobpla/article/doi/10.1093/aobpla/plz019/5421212		2019-03-01	2021-09-07 16:3	2021-10-14 0:23	2021-09-07 16:3			2	11										en		
R3IH7HIX	journalArticle	2019	Van Norman, Kar	A survey of the vascular flora of Black Hawk County, Iowa							2019	2021-09-07 16:3	2021-09-07 16:3																	
DU2R3V8A	journalArticle	2019	Park, Isaac W.; M	Climate affects th	Global Ecology and Biogeography		1466-822X, 1466	10.1111/geb.129	https://onlinelibrary.wiley.com/doi/10.1111/geb.12916		2019-04-04	2021-09-07 16:3	2021-10-14 0:23	2021-09-07 16:3	geb.12916					Global Ecol Biog	Climate affects the rate at which species successively flower							en		
SGD7753N	journalArticle	2019	Palagi, Jason M.	Deer Florivory Is	International Journal of Plant Scier		1058-5893, 1537	10.1086/702861	https://www.journals.uchicago.edu/doi/10.1086/702861		2019-06	2021-09-07 16:3	2021-12-07 20:3	2021-09-07 16:3	357-365		5	180		International Journal of Plant Sciences								en		
3UKNNZVL	journalArticle	2019	Pearson, Katelin	Spring- and fall-fl	International Journal of Biometeor		0020-7128, 1432	10.1007/s00484-	http://link.springer.com/10.1007/s00484-019-01679-0		2019-04	2021-09-07 16:3	2021-12-07 20:3	2021-09-07 16:3	481-492		4	63		Int J Biometeorol								en		
NW9UUPV8	bookSection	2019	Penfold, Christop	Inferring Gene Re	Gene Regulatory Networks						2019	2021-09-07 16:3	2021-10-14 0:30		251–282											Springer				
SMQ2XCXX	journalArticle	2019	Phillips, Juan; Ra	Differences in ca	Biotropica		0006-3606, 1744	10.1111/btp.1267	https://onlinelibrary.wiley.com/doi/10.1111/btp.12675		2019-07	2021-09-07 16:3	2021-12-07 20:3	2021-09-07 16:3	490-499		4	51		Biotropica								en		
Z6I5P3U5	journalArticle	2019	Porth, Oliver; Ch	The event horizo	The Astrophysical Journal Supplement Series						2019	2021-09-07 21:3	2021-10-14 0:26		26		2	243												
KY5YJI5T	bookSection	2019	Raborn, R Taylor	Using RAMPAGE	Insect Genomics						2019	2021-09-07 21:3	2021-10-14 0:26		99–116											Springer				
UUYWCWK2	journalArticle	2019	Ramos-Barbero,	Prokaryotic and v	Environmental Microbiology		1462-2912, 1462	10.1111/1462-29	https://onlinelibrary.wiley.com/doi/10.1111/1462-2920.14549		2019-06	2021-09-07 21:4	2021-12-07 20:3	2021-09-07 21:4	2029-2042		6	21		Environ Microbiol								en		
JCFISM6Y	journalArticle	2019	Rapacciuolo, Gio	Understanding ec	Ecography		0906-7590, 1600	10.1111/ecog.04	https://onlinelibrary.wiley.com/doi/10.1111/ecog.04616		2019-05-13	2021-09-07 21:4	2021-10-14 0:26	2021-09-07 21:4	ecog.04616					Ecography	Understanding ecological change across large spatial, temporal and taxonomic scales							en		

L4PAXBAP	journalArticle	2019	Shi, Yan; Xu, Hui	Comparative Ana	Tropical Plant Biology		1935-9756, 1935	10.1007/s12042-	http://link.springer.com/10.1007/s12042-019-09230-6		2019-09	2021-09-07 21:4	2021-12-07 20:3	2021-09-07 21:4	174-185		3	12	Tropical Plant Biol.						en	
KCHPPQU9	journalArticle	2019	Stucky, Brian; Ba	Developing a voc	Biodiversity Data Journal		1314-2828, 1314	10.3897/BDJ.7.e	https://bdj.pensof	Insects are possibly the most taxonomically and ecologically diverse class of multicellular organisms on Earth. Con	2019-03-13	2021-09-07 21:4	2021-10-14 0:23	2021-09-07 21:4	e33303			7	BDJ	Developing a vocabulary and ontology for modeling insect natural history data						
YUKWYF8A	journalArticle	2019	Wiens, John J.; C	Climate change,	Molecular Ecology		0962-1083, 1365	10.1111/mec.150	https://onlinelibrary.wiley.com/doi/10.1111/mec.15073		2019-05	2021-09-07 21:4	2021-12-07 20:3	2021-09-07 21:4	2610-2624		10	28	Mol Ecol						en	
ZGALBQQD	journalArticle	2019	Schumacker, Sco	RNA sequencing	Data in Brief		23523409	10.1016/j.dib.201	https://linkinghub.elsevier.com/retrieve/pii/S2352340919303592		2019-08	2021-09-07 21:4	2021-12-07 20:3	2021-09-07 21:4	104006			25	Data in Brief						en	
YXSV9CFJ	journalArticle	2019	Ude, George N.;	Genetic Diversity	American Journal of Plant Science		2158-2742, 2158	10.4236/ajps.201	http://www.scirp.org/journal/doi.aspx?DOI=10.4236/ajps.2019.101015		2019	2021-09-07 21:4	2021-10-14 0:24	2021-09-07 21:4	179-207		1	10	AJPS							
C2DX34R7	journalArticle	2019	Wang, Runfeng;	Co-localization o	Theoretical and Applied Genetics		0040-5752, 1432	10.1007/s00122-	http://link.springer.com/10.1007/s00122-019-03277-5		2019-04	2021-09-07 21:4	2021-12-07 20:3	2021-09-07 21:4	1263-1281		4	132	Theor Appl Genet						en	
MUNMHURV	journalArticle	2019	Xiao, Huogen; Li	Metagenomic An	Plant Disease		0191-2917, 1943	10.1094/PDIS-09	https://apsjournal	The virome of a major white wine grape of cultivar Riesling showing decline and leafroll disease symptoms was ana	2019-06	2021-09-07 21:4	2021-12-07 20:3	2021-09-07 21:4	1275-1285		6	103	Plant Disease						en	
LGFDN8JY	journalArticle	2019	Wang, Chong; Jia	TAF Family Prote	Journal of Virology		0022-538X, 1098	10.1128/JVI.005	https://journals.as	SEs play critical roles in cancer development. Since SEs assemble much bigger protein complexes on enhancers t	2019-08-15	2021-09-07 21:4	2021-10-14 0:25	2021-09-07 21:4			16	93	J Virol						en	
NA4TFLRI	journalArticle	2019	Trubl, Gareth; Ro	Towards optimized viral metagenomes for double-stranded and single				10.7287/peerj.pre	https://peerj.com/	Soils impact global carbon cycling and their resident microbes are critical to their biogeochemical processing and e	2019-04-06	2021-09-07 21:4	2021-10-14 0:32	2021-09-07 21:4											en	
JSVIZM6H	journalArticle	2019	Saptari, R. T.; Su	Data mining stud	In Vitro Cellular & Developmental B		1054-5476, 1475	10.1007/s11627-	http://link.springer.com/10.1007/s11627-018-9947-5		2019-04	2021-09-07 21:4	2021-12-07 20:3	2021-09-07 21:4	139-152		2	55	In Vitro Cell.Dev.Biol.-Plant						en	
KQYB9CT7	journalArticle	2019	Syrett, Camille M	Altered X-chromo	JCI Insight		2379-3708	10.1172/jci.insigh	https://insight.jci.org/articles/view/126751		2019-04-04	2021-09-07 21:4	2021-12-07 20:3	2021-09-07 21:4	e126751		7	4							en	
3WA73XCS	journalArticle	2019	Wai, Ching Man;	Time of day and	PLOS Genetics		1553-7404	10.1371/journal.p	https://dx.plos.org/10.1371/journal.pgen.1008209		2019-06-14	2021-09-07 21:4	2021-12-07 20:3	2021-09-07 21:4	e1008209		6	15	PLoS Genet						en	
RWSYBQGX	journalArticle	2019	Walls, Ramona L	The Plant Ontolo	Frontiers in Plant Science		1664-462X	10.3389/fpls.201	https://www.frontiersin.org/article/10.3389/fpls.2019.00631/full		2019-06-04	2021-09-07 21:4	2021-12-07 20:3	2021-09-07 21:4	631			10	Front. Plant Sci.							
BTXHJHDU	journalArticle	2019	Xu, Weijia; Gupta	Improving publica	Data and Information Management						2019	2021-09-07 21:4	2021-10-14 0:24		3–17		1	3								
4BA3S6CK	journalArticle	2019	Youens-Clark, Ke	iMicrobe: Tools a	GigaScience						2019	2021-09-07 21:4	2021-09-07 21:4		giz083		7	8								
HIDLW4PL	journalArticle	2019	Verdier, James M	Joanne S. Tornow	BioScience		0006-3568, 1525	10.1093/biosci/bi	https://academic.	Abstract Joanne S. Tornow was selected as assistant director for the National Science Foundation's (NSF)	2019-08-01	2021-09-07 21:5	2021-09-07 21:5	2021-09-07 21:5	594-601		8	69		Joanne S. Tornow					en	
WAK6HZYC	journalArticle	2019	Stucky, Brian; De	From Field Obse	Biodiversity Information Science ar		2535-0897	10.3897/biss.3.3	https://biss.pensof	Ideally, an information system that automates the integration of disparate datasets should be able to minimize the l	2019-07-02	2021-09-07 21:5	2021-09-07 21:5	2021-09-07 21:5	e37614			3	BISS	From Field Observations and Plant Specimens to a Trans-continental Knowledge Base						
WHR7WD5P	journalArticle	2019	Yan, Yujing; Tang	Protecting endem	Journal of Plant Ecology		1752-9921, 1752	10.1093/jpe/rtz0	https://academic.	Abstract Aims Climate change in the near future may become a major threat to high-altitu	2019-11-28	2021-09-07 21:5	2021-09-07 21:5	2021-09-07 21:5	962-971		6	12		Protecting endemic seed plants on the Tibetan Plateau under future climate change					en	
NDDRXTT5	book	2019		Universal access in human-compu		978-3-030-23563-5 978-3-030-23562-8					2019	2021-09-07 21:5	2021-09-07 21:5			608				Universal access	Lecture notes in	11573	Springer	Cham	eng	
A7S78ML5	book	2019		Phytochromes: methods and proto		978-1-4939-9612-4 978-1-4939-9611-7					2019	2021-09-07 21:5	2021-09-07 21:5			278				Phytochromes	Methods in mole	2026	Humana Press	New York, NY	eng	
SGZB5JI5	journalArticle	2019	Gesing, Sandra;	The Science Gat	Future Generation Computer Syste		0167739X	10.1016/j.future.2	https://linkinghub.elsevier.com/retrieve/pii/S0167739X18315176		2019-12	2021-09-07 21:5	2021-12-07 20:3	2021-09-07 21:5	951-958			101	Future Generatio	The Science Gateways Community Institute					en	
YHYIUID8	journalArticle	2019	Anderson, Steven	Prediction of Mai	The Plant Phenome Journal		2578-2703, 2578	10.2135/tppj2019	https://onlinelibrary.wiley.com/doi/abs/10.2135/tppj2019.02.0004		2019-01	2021-09-07 21:5	2021-12-07 20:3	2021-09-07 21:5	1-15		1	2	Plant phenome j.						en	
J4ZB7GEV	journalArticle	2019	Prenger, Elizabet	Identification and	Theoretical and Applied Genetics		0040-5752, 1432	10.1007/s00122-	http://link.springer.com/10.1007/s00122-019-03399-w		2019-11	2021-09-07 21:5	2021-12-07 20:3	2021-09-07 21:5	2965-2983		11	132	Theor Appl Genet						en	
DTF4QIHV	artwork	2019	Das, Abhiram; Bu	Infrastructure for managing and analyzing biological networks derived from collections c					http://www.computational-plant-science.org/joomla30/downloads/dasBigData.pdf		2019	2021-09-07 21:5	2021-10-14 19:3													
CMQ5XL9I	journalArticle	2019	Via, Allegra; Attw	A new pan-Europ	Briefings in Bioinformatics		1477-4054	10.1093/bib/bbx1	https://academic.oup.com/bib/article/20/2/405/4237465		2019-03-25	2021-09-07 21:5	2021-10-12 22:4	2021-09-07 21:5	405-415		2	20		A new pan-European Train-the-Trainer programme for bioinformatics					en	
KKSIVFNN	journalArticle	2019	Arndt, David; Ma	PHAST, PHASTE	Briefings in Bioinformatics		1467-5463, 1477	10.1093/bib/bbx1	https://academic.	Abstract PHAST (PHAge Search Tool) and its successor PHASTER (PHAge Search Tool – Enhanced Rele	2019-07-19	2021-09-07 21:5	2021-10-12 22:4	2021-09-07 21:5	1560-1567		4	20		PHAST, PHASTER and PHASTEST					en	
NH9U7T3B	journalArticle	2019	Xie, Xiao-Ting; K	Prevalence of fec	MicrobiologyOpen		20458827	10.1002/mbo3.62	https://onlinelibrary.wiley.com/doi/10.1002/mbo3.622		2019-01	2021-09-07 21:5	2021-12-07 20:3	2021-09-07 21:5	e00622		1	8	MicrobiologyOpen						en	
T2LC6ZFG	journalArticle	2019	Dovrolis, Nikolas	Computational pr	Briefings in Bioinformatics		1477-4054	10.1093/bib/bbx1	https://academic.oup.com/bib/article/20/3/825/4665690		2019-05-21	2021-09-07 21:5	2021-10-12 22:4	2021-09-07 21:5	825-841		3	20		Computational profiling of the gut–brain axis					en	
9ST5CQXQ	journalArticle	2019	Schneider, Maria	Establishing a dis	Briefings in Bioinformatics		1477-4054	10.1093/bib/bbx0	https://academic.oup.com/bib/article/20/2/384/3904481		2019-03-25	2021-09-07 21:5	2021-10-12 22:4	2021-09-07 21:5	384-389		2	20							en	
IF9WISIS	book	2019		Insect genomics: methods and pro		978-1-4939-8775-7 978-1-4939-9389-5					2019	2021-09-07 21:5	2021-09-07 21:5			237				Insect genomics	Methods in mole	1858	Humana Press	New York, NY	eng	
SSQVHANG	journalArticle	2019	Sankoff, David; Z	Models for Simila	IEEE/ACM Transactions on Compu		1545-5963, 1557	10.1109/TCBB.20	https://ieeexplore.ieee.org/document/8423659/		2019-05-01	2021-09-07 21:5	2021-12-07 20:3	2021-09-07 21:5	727-737		3	16	IEEE/ACM Trans. Comput. Biol. and Bioinf.							
XX2THD24	journalArticle	2019	D'Agostino, Dani	A science gatewa	Future Generation Computer Syste		0167739X	10.1016/j.future.2	https://linkinghub.elsevier.com/retrieve/pii/S0167739X17310051		2019-05	2021-09-07 21:5	2021-12-07 20:3	2021-09-07 21:5	868-878			94	Future Generation Computer Systems						en	
WAHTIZRJ	journalArticle	2019	Kalyanam, Rajes	MyGeoHub—A s	Future Generation Computer Syste		0167739X	10.1016/j.future.2	https://linkinghub.elsevier.com/retrieve/pii/S0167739X17310087		2019-05	2021-09-07 21:5	2021-12-07 20:3	2021-09-07 21:5	820-832			94	Future Generation Computer Systems						en	
RIWLKECG	journalArticle	2019	McCord, Bruce R	Forensic DNA An	Analytical Chemistry		0003-2700, 1520	10.1021/acs.ana	https://pubs.acs.org/doi/10.1021/acs.analchem.8b05318		2019-01-02	2021-09-07 21:5	2021-10-14 0:24	2021-09-07 21:5	673-688		1	91	Anal. Chem.						en	
E4K84P9D	journalArticle	2019	Enke, Raymond A	Genomics RNA-seq Analysis part 1-CyVerse DE, FastQC, Trimmomatic (updated 11/17)							2019	2021-09-07 22:0	2021-09-07 22:0													
35I6FKS8	journalArticle	2019	Čerekovič, Nataš	Differences in Physiological, Biochemical, and Molecular Mechanisms of Water Stress T					https://www.researchgate.net/profile/Palmiro_Poltronieri2/publication/322356803_Variability_in_Physiological_Biochemical_and_Mol		2019	2021-09-07 22:0	2021-10-14 19:2													
2ELBKTAP	journalArticle	2019	O'Mullane, Willia	The demise of th	arXiv:1907.13060 [astro-ph]				http://arxiv.org/ab	Many astronomy data centres still work on filesystems. Industry has moved on; current practice in computing infras	2019-07-31	2021-09-07 22:0	2021-09-07 22:0	2021-09-07 22:0												
UFU53RJB	conferencePaper	2019	Susla, Mykhailo;	Principles of Buil	2019 9th Internat	978-1-72810-449-2 978-1-72810-4		10.1109/ACITT.2	https://ieeexplore.ieee.org/document/8780093/		2019-06	2021-09-07 22:0	2021-12-07 20:3	2021-09-07 22:0	145-148								IEEE	Ceske Budejovice, Czech Republic		
ZQU6HWP9	conferencePaper	2019	Rosenberg, Jake	Leveraging Elast	Proceedings of th	978-1-4503-7227-5		10.1145/333218	https://dl.acm.org/doi/10.1145/3332186.3332230		2019-07-28	2021-09-07 22:0	2021-09-07 22:0	2021-09-07 22:0	1-5								ACM	Chicago IL USA	en	
3NYREL92	journalArticle	2019	Cohen, James I.	The conservation	Conservation Genetics		1566-0621, 1572	10.1007/s10592-	http://link.springer.com/10.1007/s10592-019-01212-1		2019-12	2021-09-07 22:0	2021-12-07 20:3	2021-09-07 22:0	1315-1328		6	20	Conserv Genet						en	
HY3NC6XE	journalArticle	2019	Gill, Ajay; Blackb	Prospects for Wi	Publications of the Astronomical So		0004-6280, 1538	10.1088/1538-38	https://iopscience.iop.org/article/10.1088/1538-3873/ab32a8		2019-10-01	2021-09-07 22:0	2021-09-07 22:0	2021-09-07 22:0	124501		1006	131	PASP							
9BN6VRTR	conferencePaper	2019	Baller, Joshua; K	CHURP: A Lightw	Proceedings of th	978-1-4503-7227-5		10.1145/333218	https://dl.acm.org/doi/10.1145/3332186.3333156		2019-07-28	2021-09-07 22:0	2021-09-07 22:0	2021-09-07 22:0	1-5					CHURP			ACM	Chicago IL USA	en	
3IMCDXW5	journalArticle	2019	Peterson, April L.	Conservation of t	Heredity		0018-067X, 1365	10.1038/s41437-	http://www.nature.com/articles/s41437-019-0252-9		2019-10	2021-09-07 22:0	2021-12-07 20:3	2021-09-07 22:0	442-457		4	123	Heredity						en	
ZAAFATD8	journalArticle	2019	Oktavia, Dina; Jin	Species-habitat a	Perspectives in Plant Ecology, Evo		14338319	10.1016/j.ppees.	https://linkinghub.elsevier.com/retrieve/pii/S1433831918301793		2019-10	2021-09-07 22:0	2021-12-07 20:3	2021-09-07 22:0	125482			40	Perspectives in Plant Ecology, Evolution and Systematics						en	
8CFH6IWU	journalArticle	2019	Navale, Vivek; Ji	Development of a	F1000Research		2046-1402	10.12688/f1000re	https://f1000rese	Biomedical translational research can benefit from informatics system that support the confidentiality, integrity and a	2019-08-14	2021-09-07 22:0	2021-12-07 20:3	2021-09-07 22:0	1430			8	F1000Res						en	
XURG7IWM	conferencePaper	2019	Subramanian, Ra	Parallel Framewo	Proceedings of th	978-1-4503-7227-5		10.1145/333218	https://dl.acm.org/doi/10.1145/3332186.3338097		2019-07-28	2021-09-07 22:0	2021-09-07 22:0	2021-09-07 22:0	1-8								ACM	Chicago IL USA	en	
Q3I46EXG	conferencePaper	2019	Cleveland, Sean	Enabling Real-Ti	Proceedings of th	978-1-4503-7227-5		10.1145/333218	https://dl.acm.org/doi/10.1145/3332186.3332252		2019-07-28	2021-09-07 22:0	2021-09-07 22:0	2021-09-07 22:0	1-6					Enabling Real-Time User Interaction For Decision Support			ACM	Chicago IL USA	en	
2V96MCWB	journalArticle	2019	Deaton, Jonathan	Mini-Metagenom	Advanced Biosystems		2366-7478, 2366	10.1002/adbi.201	https://onlinelibrary.wiley.com/doi/10.1002/adbi.201900108		2019-11	2021-09-07 22:0	2021-12-07 20:3	2021-09-07 22:0	1900108		11	3	Adv. Biosys.						en	
2PXJTF4V	journalArticle	2019	Jantzen, Johanna	Effects of taxon s	Ecology and Evolution		2045-7758, 2045	10.1002/ece3.54	https://onlinelibrary.wiley.com/doi/10.1002/ece3.5425		2019-09	2021-09-07 22:0	2021-12-07 20:3	2021-09-07 22:0	9479-9499		17	9	Ecol Evol						en	
S4JX72JD	journalArticle	2019	Huang, Yu; Zhou	A comparative st	Ecology and Evolution		2045-7758, 2045	10.1002/ece3.55	https://onlinelibrary.wiley.com/doi/10.1002/ece3.5589		2019-09	2021-09-07 22:0	2021-12-07 20:3	2021-09-07 22:0	10707-10722		18	9	Ecol Evol						en	
IX6CD6SX	journalArticle	2019	Feng, Zijin; Hou,	Dissecting the M	Frontiers in Pharmacology		1663-9812	10.3389/fphar.20	https://www.frontiersin.org/article/10.3389/fphar.2019.00845/full		2019-07-30	2021-09-07 22:0	2021-12-07 20:3	2021-09-07 22:0	845			10	Front. Pharmacol.							
NCLIPPYT	journalArticle	2019	Liu, Zac Yung-Ch	Deep learning ac	Animal Biotelemetry		2050-3385	10.1186/s40317-	https://animalbiotelemetry.biomedcentral.com/articles/10.1186/s40317-019-0175-5		2019-12	2021-09-07 22:0	2021-12-07 20:3	2021-09-07 22:0	14		1	7	Anim Biotelemetry						en	
5PQJ5CW6	book	2019		Genetics and genomics of Linum		978-3-030-23964-0 978-3-030-23966-4					2019	2021-09-07 22:0	2021-09-07 22:0			270					Plant genetics ar	volume 23	Springer	Cham, Switzerlar	eng	
JAUR4MN9	journalArticle	2019	Duong, Duy An;	Draft Genome As	Microbiology Resource Announcer		2576-098X	10.1128/MRA.00	https://journals.as	Photorhabdus luminescens subsp. sonorensis strain Caborca is an entomopathogenic b	2019-09-05	2021-09-07 22:0	2021-09-07 22:0	2021-09-07 22:0			36	8	Microbiol Resour Announc						en	
AN2LQ96T	journalArticle	2019	Crimmins, M. A.;	Does an Early Sp	Journal of Geophysical Research:		2169-8953, 2169	10.1029/2019JG	https://onlinelibrary.wiley.com/doi/abs/10.1029/2019JG005297		2019-08	2021-09-07 22:0	2021-12-07 20:3	2021-09-07 22:0	2628-2641		8	124	J. Geophys. Res	Does an Early Spring Indicate an Early Summer?					en	
B8AY27GG	journalArticle	2019	Castillo, Yaiza M.	Assessing the vir	Molecular Ecology		0962-1083, 1365	10.1111/mec.152	https://onlinelibrary.wiley.com/doi/10.1111/mec.15210		2019-09	2021-09-07 22:0	2021-12-07 20:3	2021-09-07 22:0	4272-4289		18	28	Mol Ecol						en	
VU6J35A7	journalArticle	2019	Suzuki, Yoshihiko	Long-read metag	Microbiome		2049-2618	10.1186/s40168-	https://microbiomejournal.biomedcentral.com/articles/10.1186/s40168-019-0737-z		2019-12	2021-09-07 22:0	2021-12-07 20:3	2021-09-07 22:0	119		1	7	Microbiome						en	
U7MYSNZL	journalArticle	2019	Danczak, Robert	Capability for ars	PLOS ONE		1932-6203	10.1371/journal.p	https://dx.plos.org/10.1371/journal.pone.0221694		2019-09-06	2021-09-07 22:0	2021-12-07 20:3	2021-09-07 22:0	e0221694		9	14	PLoS ONE						en	
MPAFD4NL	journalArticle	2019	Onyia, Christie; C	A new approach	Journal of Biodiversity & Endangered Species						2019	2021-09-07 22:0	2021-09-07 22:0		1–4		2	7								
NB5F8ASQ	journalArticle	2019	Buoso, Sara; Pag	'Candidatus Phyt	BMC Genomics		1471-2164	10.1186/s12864-	https://bmcgenor	Abstract Background ' Candidatus Phytoplasma solani' is (	2019-12	2021-09-07 22:0	2021-12-07 20:3	2021-09-07 22:0	703		1	20	BMC Genomics						en	
U8ITNRTA	book	2019	Martinez, Delsy	Identifying chromosomal regions associated with glucocorticoid-regulated gene transcription							2019	2021-09-07 22:0	2021-09-07 22:0										University of California, San Francisco			
4AHIBRP4	conferencePaper	2019	Stewart, Craig A.	Assessment of no	Proceedings of th	978-1-4503-7279-4		10.1145/3355738	https://dl.acm.org/doi/10.1145/3355738.3355749		2019-07-29	2021-09-07 22:0	2021-09-07 22:0	2021-09-07 22:0	1-10					Assessment of non-financial returns on cyberinfrastructure			ACM	Chicago IL USA	en	
654ZTAQN	journalArticle	2019	Monson, Melissa	Genetic lines res	Scientific Reports		2045-2322	10.1038/s41598-	http://www.nature	Abstract Exposure to high temperatures is known to impair immune functions and disease resistance of pou	2019-12	2021-09-07 22:0	2021-12-07 20:3	2021-09-07 22:0	13649		1	9	Sci Rep						en	
NR9HTG25	journalArticle	2019	Hudson, Kathleen	Maternal cadmiu	Scientific Reports		2045-2322	10.1038/s41598-	http://www.nature	Abstract Cadmium (Cd) is a toxic heavy metal ubiquitous in the environment. Maternal exposure to Cd is as	2019-12	2021-09-07 22:0	2021-12-07 20:3	2021-09-07 22:0	13553		1	9	Sci Rep						en	
6S2B5QRD	book	2019	Arias, Joshua Da	Phylogenomic Framework of the Plant Family Acanthaceae							2019	2021-09-07 22:0	2021-09-07 22:0										California State University, Long Beach			
Y4TENHSX	thesis	2019	Roy, Samapriya	Delta Dynamics: Understanding Process, Pattern, and People Using Remote Sensing and Systems Analysis in Coastal Louisiana and Amazon River Delta							2019	2021-09-07 22:0	2021-09-07 22:0										Indiana University			PhD Thesis
AXUMR6WZ	journalArticle	2019	Santana de Carv	An Integrated Genomics And Phenomics Approach To Study The Evolution Of C4 Photosynthesis							2019	2021-09-07 22:0	2021-09-07 22:0													
KL6IBVCH	journalArticle	2019	Metzger, Stefan;	From NEON Fiel	Bulletin of the American Meteorolo		0003-0007, 1520	10.1175/BAMS-D	https://journals.ar	Abstract The National Ecological Observatory Network (NEON) is a multidecadal and continental-	2019-11	2021-09-07 22:0	2021-12-07 20:3	2021-09-07 22:0	2305-2325		11	100		From NEON Field Sites to Data Portal						
B8LFVC4T	journalArticle	2019	Kalidindi, Surya R	Data Infrastructu	Integrating Materials and Manufact		2193-9764, 2193	10.1007/s40192-	http://link.springer.com/10.1007/s40192-019-00156-1		2019-12	2021-09-07 22:0	2021-12-07 20:3	2021-09-07 22:0	441-454		4	8	Integr Mater Man	Data Infrastructure Elements in Support of Accelerated Materials Innovation					en	
CGDDJYTH	book	2019	Qiao, Pengfei	Pattern Recognition in the Developing Maize Leaf Epidermis: Gene Network Analyses and Machine Learning Approaches							2019	2021-09-07 22:1	2021-09-07 22:1										Cornell University			
4WL8NZ56	journalArticle	2019	Groom, Quentin;	Improving Darwin Core for research and management of alien species							2019	2021-09-07 22:1	2021-09-07 22:1													
YD6HDVG3	book	2019		Industrial and food crops		978-3-030-23265-8 978-3-030-23264-1					2019	2021-09-07 22:1	2021-09-07 22:1			744					Advances in plan	Volume 6	Springer	Cham	eng	
7Y9RMRWW	journalArticle	2019	Smith, Ashley R	Dissection of Floral Organ Development and Sterility in Sorghum Bicolor							2019	2021-09-07 22:1	2021-09-07 22:1													
KINRPDPF	journalArticle	2019	Carpenter, Eric J	Access to RNA-s	GigaScience		2047-217X	10.1093/gigascie	https://academic.	Abstract Background The 1000 Plant transcriptomes initiative (1KP) explored genetic dive	2019-10-01	2021-09-07 22:1	2021-09-07 22:1	2021-09-07 22:1	giz126		10	8		Access to RNA-sequencing data from 1,173 plant species					en	
6D95587F	journalArticle	2019	Morera, Andrés A	TDP-43 regulates	Biochimica et Biophysica Acta (BB		18749399	10.1016/j.bbagrm	https://linkinghub.elsevier.com/retrieve/pii/S1874939919300987		2019-10	2021-09-07 22:1	2021-12-07 20:3	2021-09-07 22:1	194434		10	1862	Biochimica et Biophysica Acta (BBA) - Gene Regulatory Mechanisms						en	
W6528JY2	journalArticle	2019	Bao, Yin; Zareco	Assessing plant p	Plant Methods		1746-4811	10.1186/s13007-	https://plantmeth	Abstract Background Assessing the impact of the environment on plant performance requ	2019-12	2021-09-07 22:1	2021-12-07 20:3	2021-09-07 22:1	117		1	15	Plant Methods						en	
TXLEHGFW	journalArticle	2019	Leebens-Mack, J	One thousand plant transcriptomes and the phylogenomics of green plants							2019	2021-09-07 22:1	2021-09-07 22:1													
HDILAF37	journalArticle	2019	McLean, Jared H	The 'Ike Wai Haw	Proc. Gateways, San Diego, CA, USA						2019	2021-09-07 22:1	2021-09-07 22:1													
BICT5F6P	journalArticle	2019	Horvath, David P	Varying weed der	The Plant Genome						2019	2021-09-07 22:1	2021-09-07 22:1				3	12								
R6HYIQQL	journalArticle	2019	Naithani, Sushma	Plant Reactome:	Nucleic Acids Research		0305-1048, 1362	10.1093/nar/gkz9	https://academic.	Abstract Plant Reactome (https://plantreactome.gramene.org) is an open-source, comparative plant pathwa	2019-11-04	2021-09-07 22:1	2021-09-07 22:1	2021-09-07 22:1	gkz996					Plant Reactome					en	
2EQC5G8Y	conferencePaper	2019	Nguyen, Minh; D	Multi-Cloud Perfo	2019 IEEE 27th I	978-1-72812-700-2		10.1109/ICNP.20	https://ieeexplore.ieee.org/document/8888071/		2019-10	2021-09-07 22:1	2021-12-07 20:3	2021-09-07 22:1	1-2								IEEE	Chicago, IL, USA		
5HCZCHEU	conferencePaper	2019	Pandey, Ashish; V	Data-intensive W	2019 IEEE 27th International Conference on Network Protocols (ICNP)						2019	2021-09-07 22:1	2021-09-07 22:1		1–2								IEEE			
HJGW53SY	journalArticle	2019	Crimmins, Theres	Biologically-Rele	Geophysical Research Letters		0094-8276, 1944	10.1029/2019GL	https://onlinelibrary.wiley.com/doi/10.1029/2019GL085251		2019-11-16	2021-09-07 22:1	2021-09-07 22:1	2021-09-07 22:1	12377-12387		21	46	Geophys. Res. Lett.						en	
7CDBCRJ2	book	2019	Priyadarshan, P.	Plant breeding: classical to modern		9789811370953 9789811370946					2019	2021-09-07 22:1	2021-09-07 22:1			570				Plant breeding			Springer	Singapore	eng	
BEVVIEWT	journalArticle	2019	Tran, Mai Hoang	Tar-filled shells a	BIOS		0005-3155	10.1893/0005-31	https://bioone.org/journals/bios/volume-90/issue-2/0005-3155-90.2.122/Tar-filled-shells-and-tar-balls-on-the-Alabama-Gulf/10.1893/0		2019-11-08	2021-09-07 22:1	2021-12-07 20:3	2021-09-07 22:1	122		2	90	BIOS							
H9RIA3RL	journalArticle	2019	Koutouleas, Athin	On the hunt for th	Ecology and Evolution		2045-7758, 2045	10.1002/ece3.57	https://onlinelibrary.wiley.com/doi/10.1002/ece3.5755		2019-12	2021-09-07 22:1	2021-12-07 20:3	2021-09-07 22:1	13619-13631		23	9	Ecol Evol						en	
QPCN2DN2	journalArticle	2019	Nelson, Andrew D	Transcriptomic an	BMC Plant Biology		1471-2229	10.1186/s12870-	https://bmcplantb	Abstract Background Guayule (Parthenium argentatum Gray	2019-12	2021-09-07 22:1	2021-12-07 20:3	2021-09-07 22:1	494		1	19	BMC Plant Biol						en	
XHLJ7J8W	thesis	2019	Luis, Jane Marian	Sexual Reproduction and Enhancing the Efficacy of Biocontrol of Aflatoxin Contamination by Aspergillus flavus							2019	2021-09-07 22:1	2021-09-07 22:1										North Carolina State University			PhD Thesis
3LQLSSDY	book	2019	Parveen, Alia	Chasing the Genetics of Ascites in Broilers Using Whole Genome Resequencing							2019	2021-09-07 22:1	2021-09-07 22:1										University of Arkansas			
8X3DTZR9	journalArticle	2019	Hollebecq, Jean-	Identification of objects with Uniform Resource Identifier (URI): recommendations for application in plant phenotyping							2019	2021-09-07 22:1	2021-09-07 22:1													
5A75NRP5	journalArticle	2019	Mahalingam, Ram	Analysis of the B	International Journal of Molecular S		1422-0067	10.3390/ijms210	https://www.mdpi	Barley seeds are one of the main ingredients of the malting industry for brewing beer. The barley rootlets that are s	2019-12-26	2021-09-07 22:1	2021-09-07 22:1	2021-09-07 22:1	179		1	21	IJMS						en	
G3MIN8EM	journalArticle	2019	Van Goethem, M	Characteristics o	mBio		2161-2129, 2150	10.1128/mBio.02	https://journals.as	This work forms part of an overarching research theme studying the effects of a changing climate on biological soil	2019-12-24	2021-09-07 22:1	2021-09-07 22:1	2021-09-07 22:1			6	10	mBio						en	
K8X54YKA	journalArticle	2019	Zeng, Shuai; Lyu	Knowledge Base	BMC Genomics		1471-2164	10.1186/s12864-	https://bmcgenor	Abstract Background Knowledge Base Commons (KBCommons) v1.1 is a universal and	2019-12	2021-09-07 22:1	2021-12-07 20:3	2021-09-07 22:1	947		S11	20	BMC Genomics	Knowledge Base Commons (KBCommons) v1.1					en	
M7ZCTMCJ	conferencePaper	2019	Mondal, Amit Kur	An Exploratory S	2019 19th Interna	978-1-72814-937-0		10.1109/SCAM.2	https://ieeexplore.ieee.org/document/8930885/		2019-09	2021-09-07 22:1	2021-12-07 20:3	2021-09-07 22:1	62-73								IEEE	Cleveland, OH, USA		
39Y8GXS3	journalArticle	2019	Patterson, Eric L.	Omics Potential i	Plants		2223-7747	10.3390/plants81	https://www.mdpi	The rapid development of omics technologies has drastically altered the way biologists conduct research. Basic pla	2019-12-14	2021-09-07 22:1	2021-09-07 22:1	2021-09-07 22:1	607		12	8	Plants						en	
IYBRI4UR	journalArticle	2019	Nixon, Sophie L.;	Genome-Resolve	mSphere		2379-5042	10.1128/mSphere	https://journals.as	The Verrucomicrobia phylum of bacteria is widespread in many different ecosystems; however, its	2019-12-18	2021-09-07 22:1	2021-09-07 22:1	2021-09-07 22:1			6	4	mSphere						en	
4UQIY4VU	thesis	2019	Dobrin, Barbara	Aspects of Phylogenetic Inference: Missing Data and Rate Variation							2019	2021-09-07 22:1	2021-09-07 22:1										The University of Arizona			PhD Thesis
7V82CCHI	journalArticle	2019	Wright, April M.; S	The why, when, a	F1000Research		2046-1402	10.12688/f1000re	https://f1000rese	Many biologists are interested in teaching computing skills or using computing in the classroom, despite not being t	2019-11-05	2021-09-07 22:2	2021-12-07 20:3	2021-09-07 22:2	1854			8	F1000Res						en	
MBBNSNTD	journalArticle	2019	Šímová, Irena; S	The relationship	Journal of Ecology		0022-0477, 1365	10.1111/1365-274	https://onlinelibrary.wiley.com/doi/10.1111/1365-2745.13163		2019-09	2021-09-07 22:2	2021-12-07 20:2	2021-09-07 22:2	2278-2290		5	107	J Ecol						en	
6QMM5LZH	journalArticle	2019	Paniagua-Ugarte	Using DNA Barco	Banisteria, Virginia Natural History Society				http://www.virginianaturalhistorysociety.com/banisteria/pdf-files/ban53/Banisteria53_Paniagua_Ugarte_etal_Digitizing.pdf		2019	2021-09-07 22:2	2021-10-14 19:3				22-26	53								
D2LWECSW	journalArticle	2019	Stoltzfus, Rutger	Community and Code: Nine Lessons from Nine NES			2046-1402	10.12688/f1000re	https://f1000rese	"In recent years, there has been an explosion in the popularity of hackathons — creative, participant-driven meetin	2019-11-23	2021-10-12 2:55	2021-12-07 20:3				786									
ATHFZK3Z	bookSection	2019	Raborn, Volker P	Using RAMPAGE to Identify and A		978-1-4939-8774-0 978-1-4939-8775-7			http://link.springer.com/10.1007/978-1-4939-8775-7_9		2019	2021-10-12 2:57	2021-10-12 2:57		99-116			1858								
AI3PWRQ3	journalArticle	2019	Choi, Bonnie L, Il	Libra: scalable <i>k-</i> mer–based tool for massive			2047-217X	10.1093/gigascie	https://academic.oup.com/gigascience/article/doi/10.1093/gigascience/giy165/5266304		2019-02-01	2021-10-12 2:57	2021-12-07 20:3				2	8							en	
9QHLHNQ8	journalArticle	2019	Čerekovič, Palmi	Differences in Physiological, Biochemical, and Molecular Mechanisms of Water Stress Tolerance in Chickpea Varieties							2019	2021-10-12 2:57	2021-12-03 18:5													
7BPQES69	thesis	2019	Youens-Clark, by	iMicrobe: Tools and Data-Driven Discovery Platform for the Microbiome Sciences					https://search.pro	Background: Scientists have amassed a wealth of microbiome datasets making it possible to study microbes in bio	2019	2021-10-12 23:3	2021-10-12 23:3										University of Arizona			PhD Thesis
JGXRE32I	journalArticle	2019	Ley-López, Juan	Plants and gall h	Revista de Biología Tropical		2215-2075	10.15517/rbt.v67	https://revistas.uc	There has been an increasing number of inventories of gall-inducing arthropods in the Neotropics. Nonetheless, ve	2019-05	2021-10-12 23:3	2022-03-04 17:1		S212–S227		2 SUPL	67								

8K3K5RFX	journalArticle	2019	Gardiner, Laura-J	Next-Generation	Applications of Genetic and Genomic Research in C			10.1016/B978-0-	https://www.scier	Wheat is a major world food crop and its production needs to increase to meet future demand. However, having a l	2019-01	2021-10-12 23:3	2021-10-12 23:3		49–63										
WR4CRJUT	report	2019	Krueger, Raymor	A Survey of the Vascular Flora of Adams County, North Dakota					https://search.proquest.com/docview/2229833052?pq-origsite=gscholar		2019	2021-10-12 23:3	2021-10-12 23:3												
RBYGVL5A	journalArticle	2019	Khangura, Rajde	A Very Oil Yellow	G3 (Bethesda, Md.)		2160-1836	10.1534/g3.118.2	http://www.ncbi.n	Forward genetics determines the function of genes underlying trait variation by identifying the change in DNA respo	2019-02	2021-10-12 23:3	2021-10-12 23:3		375–390		2	9							
L5YE72Y5	journalArticle	2019	Jang, Ho Bin; Bo	Gene sharing net	bioRxiv			10.1101/533240	https://www.biorx	Viruses of bacteria and archaea are likely to be critical to all natural, engineered and human ecosystems, and yet tl	2019-01	2021-10-12 23:3	2021-10-12 23:3		533240										
WXQRS7YW	journalArticle	2019	Oliver, Jeffrey C.;	Data Science Su	Journal of Library Administration		0193-0826	10.1080/0193082	https://www.tandf	AbstractData science is a rapidly growing field with applications across all scientific domains. The demand for supp	2019-04	2021-10-12 23:3	2021-10-12 23:3		241–257		3	59							
XPTQNRJK	journalArticle	2019	Zhang, Qi	Data Analysis of	Computational Epigenetics and Diseases			10.1016/B978-0-	https://www.scier	In this chapter, we reviewed many aspects of the common workflow for ChIP-Seq data analysis. First, we remindec	2019-01	2021-10-12 23:3	2021-10-12 23:3		67–77										
PM5EAPGU	bookSection	2019	Ferreira da Silva,	Accurately Simulating Energy Consumption of I/O-Intensive Scientific Workflows					http://link.springer.com/10.1007/978-3-030-22734-0{_}11		2019-06	2021-10-12 23:3	2021-10-12 23:3		138–152							Springer, Cham			
ZBKVQS7Z	thesis	2019	Youens-Clark, Ch	iMicrobe: Tools and Data-Driven Discovery Platform for the Microbiome Sciences					http://ezproxy.libr	Background: Scientists have amassed a wealth of microbiome datasets making it possible to study microbes in bio	2019	2021-10-12 23:3	2021-10-12 23:3			38						University of Arizona			PhD Thesis
SDWD36UY	journalArticle	2019	Feehan, Spencer	A Prototype Citizen-Science Microplastics Sampling Protocol to Quantify the Magnitude					https://edsd.csd.columbia.edu/sites/default/files/content/Curriculum%20and%20Manuals/FEEHAN.SPENCER.11758.pdf		2019	2021-10-12 23:4	2021-12-03 18:5												
DY7LPWB5	journalArticle	2019	Powell, J; Stubbs	Streamed data vi	Proceedings of Science Gateways				https://www.researchgate.net/profile/Sean-Cleveland/publication/340875036_Streamed_Data_via_Cloud-Hosted_Real-Time_Data_S		2019	2021-10-13 23:3	2021-10-13 23:3		2019			2019							
MPYF78PW	report	2019	Parvathaneni, Ra	The regulatory landscape of early maize inflorescence development					http://biorxiv.org/l	Abstract Background The functional genome of agronomically important plant species remair	2019-12-10	2021-10-14 0:10	2021-10-14 0:10	2021-10-14 0:10								Plant Biology		en	preprint
E9TL82AM	bookSection	2019	Zhang, Qi	Chapter 5 - Data	Computational Epigenetics and Diseases				https://www.scier	In this chapter, we reviewed many aspects of the common workflow for ChIP-Seq data analysis. First, we remindec	2019	2021-10-14 19:3	2021-10-14 19:3		67-77			9			Translational Epigenetics	Academic Press			
MCAXJYWZ	journalArticle	2019	Sternberger, Ann	DNA Detective: C	CourseSource		23326530	10.24918/cs.201	https://qubeshub.org/community/groups/coursesource/publications?id=2652&v=1		2019	2022-02-24 21:5	2022-02-24 21:5	2022-02-24 21:5				6	CS	DNA Detective					
7LP2M2VC	book	2018	IEEE Internationa	Proceedings, 2018 IEEE International Conference on Bioinformatics and Biomedicine: 3					https://ieeexplore.ieee.org/servlet/opac?punumber=8609864		2018	2021-09-07 16:3	2021-09-07 16:3	2021-09-07 16:3						Proceedings, 2018 IEEE International Conference on Bioinformatics and Biomedicine				English	
YQGVQ4JG	thesis	2018	Saha, Pankaj	Exploring resource fairness and container orchestration in a cloud environment for scientific computing workloads							2018	2021-09-07 21:4	2021-10-14 0:23									State University of New York at Binghamton			PhD Thesis
V9VUNE89	journalArticle	2018	Shi, Jianting; Xue	Differentiation of	Current Protocols in Stem Cell Bio		1941-7322, 1938	10.1002/cpsc.74	https://onlinelibrary.wiley.com/doi/10.1002/cpsc.74		2018-12-10	2021-09-07 21:4	2021-10-14 0:23	2021-09-07 21:4	e74				Current Protocols in Stem Cell Biology					en	
ZDT7Q4YY	conferencePaper	2018	Xu, Weijia; Huan	Enabling User Dr	2018 IEEE International Conference on Big Data (Big Data)						2018	2021-09-07 21:4	2021-10-14 0:32		4276–4284							IEEE			
SUMIJ5GC	conferencePaper	2018	Zeng, Shuai; Lyu	Knowledge Base	2018 IEEE International Conference on Bioinformatics and Biomedicine (BIBM)						2018	2021-09-07 21:4	2021-10-14 0:25		589–594							IEEE			
Y8U267MU	document	2018	Bruno-Barcena, J	Systems and methods of carbon fixation using solventogenic clostridium beijerinckii							2018	2021-09-07 21:5	2021-09-07 21:5									Google Patents			
8ZP3UBHL	book	2018		Gene regulatory networks: method		978-1-4939-8881-5					2018	2021-09-07 21:5	2021-09-07 21:5							Gene regulatory networks		Springer Science	New York, NY		
JEH2FBF7	journalArticle	2018	Antequera, Tsege	ADON: Application-Driven Overlay Network-as-a-Se			2168-7161, 2372	10.1109/TCC.201	https://ieeexplore.ieee.org/document/7364221/		2018-07-01	2021-10-12 2:51	2021-12-07 20:0		640-655		3	6							
CXRK95N5	journalArticle	2018	Weeks, James M	High-performance epistasis detection in quantitative			1094-3420, 1741	10.1177/1094342	http://journals.sagepub.com/doi/10.1177/1094342016658110		2018-05	2021-10-12 2:51	2021-10-12 2:51		321-336		3	32						en	
62D2XCSE	journalArticle	2018	Pelletier, Greg E.	Which way do you lean? Using slope aspect variatio			1979337	10.1002/esp.430	http://doi.wiley.com/10.1002/esp.4306		2018-04	2021-10-12 2:55	2021-10-12 2:55		1133-1154		5	43						en	
YHPERS2N	bookSection	2018	Bertolini, Erica, E	Transcriptional and Posttranscripti		978-1-4939-7276-0 978-1-4939-7278-4			http://link.springer.com/10.1007/978-1-4939-7278-4_2		2018	2021-10-12 2:55	2021-10-12 2:55		21-29			1667							
UBQ8LENH	bookSection	2018	Irigoyen, Kranthi	Genomic Approaches to Analyze A		978-1-4939-7276-0 978-1-4939-7278-4			http://link.springer.com/10.1007/978-1-4939-7278-4_7		2018	2021-10-12 2:55	2021-10-12 2:55		73-85			1667						en	
XSUGZJEQ	bookSection	2018	Li, Nathan M., Qi	Detection of DNA Methylation by W		978-1-4939-7314-9 978-1-4939-7315-6			http://link.springer.com/10.1007/978-1-4939-7315-6_11		2018	2021-10-12 2:55	2021-10-12 2:55		185-196			1676							
W27S3LVI	bookSection	2018	Oliveira Alvareng	A Practical Guide for Comparative		978-1-4939-7461-0 978-1-4939-7463-4			http://link.springer.com/10.1007/978-1-4939-7463-4_7		2018	2021-10-12 2:55	2021-10-12 2:55		213-242			1704							
CTA8GWK8	bookSection	2018	Michener, William	Creating and Managing Metadata		978-3-319-59926-7 978-3-319-59928-1			http://link.springer.com/10.1007/978-3-319-59928-1_5		2018	2021-10-12 2:55	2021-10-12 2:55		71-88									en	
F4K6IPCZ	journalArticle	2018	Gesing, Alexande	Gathering requirements for advancing simulations in			0167739X	10.1016/j.future.2	https://linkinghub.elsevier.com/retrieve/pii/S0167739X17303011		2018-05	2021-10-12 2:55	2021-10-12 2:55		544-554			82						en	
SVZ2HSJY	journalArticle	2018	Polański, David L	Bringing numerous methods for expression and pror			1367-4803, 1460	10.1093/bioinforr	https://academic.oup.com/bioinformatics/article/34/5/884/4596941		2018-03-01	2021-10-12 2:55	2021-12-07 20:2		884-886		5	34						en	
6GYSW2WL	journalArticle	2018	Liang, James C,	Conventional and hyperspectral time-series imaging			2047-217X	10.1093/gigascie	https://academic.oup.com/gigascience/article/doi/10.1093/gigascience/gix117/4656251		2018-02-01	2021-10-12 2:55	2021-12-07 20:2				2	7						en	
JQEHFWBL	journalArticle	2018	Tello-Ruiz, Doree	Gramene 2018: unifying comparative genomics and			0305-1048, 1362	10.1093/nar/gkx1	http://academic.oup.com/nar/article/46/D1/D1181/4641898		2018-01-04	2021-10-12 2:55	2021-12-07 20:2		D1181-D1189		D1	46						en	
FJ8A3RTI	journalArticle	2018	Cooper, Pankaj, I	The Planteome database: an integrated resource for			0305-1048, 1362	10.1093/nar/gkx1	http://academic.oup.com/nar/article/46/D1/D1168/4653531		2018-01-04	2021-10-12 2:55	2021-12-07 20:2		D1168-D1180		D1	46						en	
UH5V2UJS	journalArticle	2018	Zhang, Sai Shrey	Social Plane for Recommenders in Network Perform			1932-4537	10.1109/TNSM.2	http://ieeexplore.ieee.org/document/8105883/		2018-03	2021-10-12 2:55	2021-10-12 2:55		97-111		1	15							
TAII73J6	journalArticle	2018	Hoffman, Melinda	Gene expression differs in codominant prairie grasse			1755098X	10.1111/1755-09	http://doi.wiley.com/10.1111/1755-0998.12733		2018-03	2021-10-12 2:55	2021-10-12 2:55		334-346		2	18						en	
QHUQYAXU	journalArticle	2018	Maitner, Brian J.,	The <span style="font-variant:small-caps;">bien r</s			2041210X	10.1111/2041-21	http://doi.wiley.com/10.1111/2041-210X.12861		2018-02	2021-10-12 2:55	2021-10-12 2:55		373-379		2	9						en	
EKW6T462	journalArticle	2018	Kissling, Alex R.,	Building essential biodiversity variables (EBVs) of sp			14647931	10.1111/brv.1235	http://doi.wiley.com/10.1111/brv.12359		2018-02	2021-10-12 2:55	2021-10-12 2:55		600-625		1	93						en	
YDYEXH7T	journalArticle	2018	Lang, Stefan A., I	The <i>Physcomitrella patens</i> chromosome-scal			9607412	10.1111/tpj.1380	http://doi.wiley.com/10.1111/tpj.13801		2018-02	2021-10-12 2:55	2021-10-12 2:55		515-533		3	93						en	
BFK6H5MD	book	2018	Dick, Gregory	Genomic Approaches in Earth and		978-1-118-70823-1 978-1-118-70824-8			http://doi.wiley.com/10.1002/9781118708231		2018-05-14	2021-10-12 2:57	2021-12-07 18:4											en	
Y8S68D6S	journalArticle	2018	Pease, Stephen A	Quartet Sampling distinguishes lack of support from			29122	10.1002/ajb2.101	http://doi.wiley.com/10.1002/ajb2.1016		2018-03	2021-10-12 2:57	2021-10-12 2:57		385-403		3	105						en	
JTHLRWM3	journalArticle	2018	Yost, Susan J., J	Digitization protocol for scoring reproductive phenolc			21680450	10.1002/aps3.10	http://doi.wiley.com/10.1002/aps3.1022		2018-02	2021-10-12 2:57	2021-10-12 2:57		e1022		2	6						en	
DACVUV2X	journalArticle	2018	McKain, Ya, Mich	Practical considerations for plant phylogenomics			21680450	10.1002/aps3.10	http://doi.wiley.com/10.1002/aps3.1038		2018-03	2021-10-12 2:57	2021-10-12 2:57		e1038		3	6						en	
EV3926QC	journalArticle	2018	Ayella, Moriah R.	A course-based undergraduate research experience			14708175	10.1002/bmb.211	http://doi.wiley.com/10.1002/bmb.21115		2018-05	2021-10-12 2:57	2021-10-12 2:57		285-296		3	46						en	
IR45JIIP	journalArticle	2018	Chougule, Doree	Improved RNA-seq Workflows Using CyVerse Cyber			19343396	10.1002/cpbi.53	http://doi.wiley.com/10.1002/cpbi.53		2018-09	2021-10-12 2:57	2021-10-14 0:17		e53		1	63						en	
BG725WR2	journalArticle	2018	Calkins, Patricia	Brain gene expression analyses in virgin and mated			20457758	10.1002/ece3.39	http://doi.wiley.com/10.1002/ece3.3976		2018-04	2021-10-12 2:57	2021-10-12 2:57		4312-4327		8	8						en	
K2C889GZ	journalArticle	2018	Horvath, Sharon,	Weed presence altered biotic stress and light signali			24754455	10.1002/pld3.57	http://doi.wiley.com/10.1002/pld3.57		2018-04	2021-10-12 2:57	2021-10-12 2:57		e00057		4	2						en	
XRBXQI7Y	bookSection	2018	York, Larry M.	Phenotyping Crop Root Crowns: G		978-1-4939-7746-8 978-1-4939-7747-5			http://link.springer.com/10.1007/978-1-4939-7747-5_2		2018	2021-10-12 2:57	2021-10-12 2:57		23-32			1761							
N9NIGR45	bookSection	2018	Putonti, Siobhan	Freshwater Viromes: From Sampli		978-1-4939-8726-9 978-1-4939-8728-3			http://link.springer.com/10.1007/978-1-4939-8728-3_2		2018	2021-10-12 2:57	2021-10-12 2:57		17-27			1849							
MDSENAB5	bookSection	2018	Tang, Eric, Haiba	From Alpha-Duplication to Triplicat		978-3-319-43692-0 978-3-319-43694-4			http://link.springer.com/10.1007/978-3-319-43694-4_5		2018	2021-10-12 2:57	2021-10-12 2:57		99-109										
FU4HJJ9M	bookSection	2018	Gesing, Sandra	Science Gateways in HPC: Usabili		978-3-319-73766-9 978-3-319-73767-6			http://link.springer.com/10.1007/978-3-319-73767-6_5		2018	2021-10-12 2:57	2021-10-12 2:57		73-86			36							
Q6SA4EHL	bookSection	2018	Stahl, Patricia, R	What Does Reality Look Like?		978-3-319-76988-2 978-3-319-76989-9			http://link.springer.com/10.1007/978-3-319-76989-9_2		2018	2021-10-12 2:57	2021-10-12 2:57		14-Nov									en	
KXQ9K7RB	bookSection	2018	Oriol, Ernest, Xa	An Ontology-Based Framework for		978-3-319-91562-3 978-3-319-91563-0			http://link.springer.com/10.1007/978-3-319-91563-0_14		2018	2021-10-12 2:57	2021-10-12 2:57		220-235			10816							
PCS6AUDB	bookSection	2018	McCallum, Martir	Molecular Mapping of Genes and (		978-3-319-95824-8 978-3-319-95825-5			http://link.springer.com/10.1007/978-3-319-95825-5_13		2018	2021-10-12 2:57	2021-10-12 2:57		181-196										
6NFDISYQ	bookSection	2018	Pal, Hemant J., F	Bioinformatics Tools for Shotgun M		978-981-10-7454-7 978-981-10-7455-4			http://link.springer.com/10.1007/978-981-10-7455-4_6		2018	2021-10-12 2:57	2021-10-12 2:57		91-110									en	
9KHZE5HY	journalArticle	2018	Liu, Siobhan, Uda	The conservation value of germplasm stored at the I			0960-3115, 1572	10.1007/s10531-	http://link.springer.com/10.1007/s10531-018-1497-y		2018-05	2021-10-12 2:57	2021-10-12 2:57		1347-1386		6	27						en	
6TUEFR4D	journalArticle	2018	Perdrial, Jon, Jul	A net ecosystem carbon budget for snow dominated			0168-2563, 1573	10.1007/s10533-	http://link.springer.com/10.1007/s10533-018-0440-3		2018-05	2021-10-12 2:57	2021-10-12 2:57		225-243		3	138						en	
Y7ERXJ94	journalArticle	2018	Nguyen, Tomer, T	Guidelines for RNA-seq projects: applications and o			0018-8158, 1573	10.1007/s10750-	http://link.springer.com/10.1007/s10750-018-3682-0		2018-12	2021-10-12 2:57	2021-10-12 2:57		27-May		1	825						en	
NUW2DYBB	journalArticle	2018	Huot, Cecilia, Orc	Global gene regulation in tomato plant (Solanum lyc			0167-4412, 1573	10.1007/s11103-(	http://link.springer.com/10.1007/s11103-018-0724-y		2018-05	2021-10-12 2:57	2021-10-12 2:57		57-72		2-Jan	97						en	
GG9U9F2Z	journalArticle	2018	Dickinson, Julie,	Integrating transcriptomic techniques and k-means c			1573-3882, 1573	10.1007/s11306-	http://link.springer.com/10.1007/s11306-018-1424-y		2018-10	2021-10-12 2:57	2021-10-14 0:17		126		10	14						en	
MQNWFWF2	journalArticle	2018	Do, Kristen S., H	Residential Building Energy Consumption: a Review			2196-3010	10.1007/s40518-	http://link.springer.com/10.1007/s40518-018-0099-3		2018-03	2021-10-12 2:57	2021-10-12 2:57		76-85		1	5						en	
UPE7HQB5	bookSection	2018	Méndez-García,	Metagenomic Protocols and Strate		978-0-08-102268-9			https://linkinghub.elsevier.com/retrieve/pii/B9780081022689000021		2018	2021-10-12 2:57	2021-10-12 2:57		15-54									en	
5JANULFE	journalArticle	2018	Das, Donald L., S	Transcriptomic analysis of differentially expressed ge			1744117X	10.1016/j.cbd.20	https://linkinghub.elsevier.com/retrieve/pii/S1744117X18300339		2018-12	2021-10-12 2:57	2021-10-12 2:57		37-53			28						en	
4X36ATP8	journalArticle	2018	Wang, Xiang H.-I	The Osteogenic Niche Is a Calcium Reservoir of Bor			15356108	10.1016/j.ccell.2(	https://linkinghub.elsevier.com/retrieve/pii/S1535610818304689		2018-11	2021-10-12 2:57	2021-10-14 0:26		823-839.e7		5	34						en	
N79RGVX7	journalArticle	2018	Triant, Akito Y, De	Lepidoptera genomes: current knowledge, gaps and			22145745	10.1016/j.cois.20	https://linkinghub.elsevier.com/retrieve/pii/S221457451730175X		2018-02	2021-10-12 2:57	2021-10-12 2:57		99-105			25						en	
NXWYHMPE	journalArticle	2018	Woehle, Tal, Chri	A Novel Eukaryotic Denitrification Pathway in Foram			9609822	10.1016/j.cub.20	https://linkinghub.elsevier.com/retrieve/pii/S0960982218308182		2018-08	2021-10-12 2:57	2021-10-14 0:22		2536-2543.e5		16	28						en	
8VAZ9SHD	journalArticle	2018	Domingues, Lane	The assembled transcriptome of the adult horn fly, H			23523409	10.1016/j.dib.201	https://linkinghub.elsevier.com/retrieve/pii/S2352340918307467		2018-08	2021-10-12 2:57	2021-10-12 2:57		1933-1940			19						en	
3KVFEYS4	journalArticle	2018	Essawy, Tanu, Ba	Integrating scientific cyberinfrastructures to improve			13648152	10.1016/j.envsofi	https://linkinghub.elsevier.com/retrieve/pii/S1364815217307880		2018-07	2021-10-12 2:57	2021-10-12 2:57		217-229			105						en	
WZCGDGSE	journalArticle	2018	Wijayasekara, Je	Molecular characterization of two badnavirus genom			1681702	10.1016/j.virusre	https://linkinghub.elsevier.com/retrieve/pii/S0168170217306044		2018-01	2021-10-12 2:57	2021-10-12 2:57		19-24			243						en	
LTP6XMZV	journalArticle	2018	Horvath, Shosha	Gene Space and Transcriptome Assemblies of Leafy			1550-2759	10.1017/wsc.201	https://www.caml	Abstract Leafy spurge (Euphorbia esula L.) is an invasive perennial weed infesting range and recreational lands of	2018-05	2021-10-12 2:57	2021-10-12 2:57		355-367		3	66						en	
RTKIKL65	journalArticle	2018	Rosario, Mya, Ka	Diversity of DNA and RNA Viruses in Indoor Air As A			0013-936X, 152(	10.1021/acs.est.7	https://pubs.acs.org/doi/10.1021/acs.est.7b04203		2018-02-06	2021-10-12 2:57	2021-12-07 20:2		1014-1027		3	52						en	
VC7BRBV8	journalArticle	2018	Wang, Tong, Yuli	High-Resolution Temporal and Spatial Patterns of Vi			0013-936X, 152(	10.1021/acs.est.(	https://pubs.acs.org/doi/10.1021/acs.est.8b03446		2018-09-18	2021-10-12 2:57	2021-12-07 20:2		10337-10346		18	52						en	
4I58E3VL	bookSection	2018	Baker, Stokes S.	Using DNA Barcoding To Identify D		978-0-8412-3286-0 978-0-8412-3285-3			https://pubs.acs.org/doi/abs/10.1021/bk-2018-1276.ch005		2018-01	2021-10-12 2:57	2021-10-12 2:57		67-79			1276						en	
VW6YBYWE	journalArticle	2018	Kremling, Edwar	Dysregulation of expression correlates with rare-alle			0028-0836, 1476	10.1038/nature2	http://www.nature.com/articles/nature25966		2018-03	2021-10-12 2:57	2021-10-12 2:57		520-523		7697	555						en	
3FVBT92C	journalArticle	2018	Howard-Varona,	Multiple mechanisms drive phage infection efficiency			1751-7362, 1751	10.1038/s41396-	http://www.nature.com/articles/s41396-018-0099-8		2018-06	2021-10-12 2:57	2021-10-12 2:57		1605-1618		6	12						en	
A8DDVPN4	journalArticle	2018	Wang, Ting F., G	Genetic variation may confound analysis of CRISPR			2056-5968	10.1038/s41421-	http://www.nature.com/articles/s41421-018-0025-2		2018-12	2021-10-12 2:57	2021-10-12 2:57		18		1	4						en	
ZID4S6AB	journalArticle	2018	Xu, Jisen, Huimir	PGD: Pineapple Genomics Database			2052-7276	10.1038/s41438-	http://www.nature.com/articles/s41438-018-0078-2		2018-12	2021-10-12 2:57	2021-10-14 0:25		66		1	5						en	
MT9KHWM7	journalArticle	2018	Wang, Vincent L.	Improving wood properties for wood utilization throu			2041-1723	10.1038/s41467-	http://www.nature.com/articles/s41467-018-03863-z		2018-12	2021-10-12 2:57	2021-10-12 2:57		1579		1	9						en	
7GHRTX7B	journalArticle	2018	McMullan, Matthe	The ash dieback invasion of Europe was founded by			2397-334X	10.1038/s41559-	http://www.nature.com/articles/s41559-018-0548-9		2018-06	2021-10-12 2:57	2021-10-12 2:57		1000-1008		6	2						en	
J7WQRZ3K	journalArticle	2018	Kissling, Robert F	Towards global data products of Essential Biodiversi			2397-334X	10.1038/s41559-	http://www.nature.com/articles/s41559-018-0667-3		2018-10	2021-10-12 2:57	2021-10-14 0:26		1531-1540		10	2						en	
PBXP4LIR	journalArticle	2018	Emerson, Matthe	Host-linked soil viral ecology along a permafrost thav			2058-5276	10.1038/s41564-	http://www.nature.com/articles/s41564-018-0190-y		2018-08	2021-10-12 2:57	2021-10-12 2:57		870-880		8	3						en	
KHAQ8S5N	journalArticle	2018	Stein, Rod A., Jo	Genomes of 13 domesticated and wild rice relatives			1061-4036, 1546	10.1038/s41588-	http://www.nature.com/articles/s41588-018-0040-0		2018-02	2021-10-12 2:57	2021-10-12 2:57		285-296		2	50						en	
N6SG8CR9	journalArticle	2018	Zhang, Susan J.,	Transcriptome Analysis in Spleen Reveals Differenti			2045-2322	10.1038/s41598-	http://www.nature.com/articles/s41598-018-19754-8		2018-12	2021-10-12 2:57	2021-10-12 2:57		1278		1	8						en	
RRJVNNF4	journalArticle	2018	Nakayama, Seisu	Comparative transcriptomics with self-organizing ma			2045-2322	10.1038/s41598-	http://www.nature.com/articles/s41598-018-21646-w		2018-12	2021-10-12 2:57	2021-10-12 2:57		3302		1	8						en	
ZFYHQYQ7	journalArticle	2018	Shyamal, D. S., S	Transcriptomic analysis of crustacean molting gland			2045-2322	10.1038/s41598-	http://www.nature.com/articles/s41598-018-25368-x		2018-12	2021-10-12 2:57	2021-10-12 2:57		7307		1	8						en	
NS8EKBQ3	journalArticle	2018	Ahmed, Hugo, M	A rust fungal effector binds plant DNA and modulate:			2045-2322	10.1038/s41598-	http://www.nature.com/articles/s41598-018-32825-0		2018-12	2021-10-12 2:57	2021-10-14 0:15		14718		1	8						en	
SX3AQZFI	journalArticle	2018	Kalbfleisch, Jame	Improved reference genome for the domestic horse			2399-3642	10.1038/s42003-	http://www.nature.com/articles/s42003-018-0199-z		2018-12	2021-10-12 2:57	2021-10-14 0:24		197		1	1						en	
63SXBKCM	journalArticle	2018	Fusi, Eric, Federi	Building Global Genomics Initiatives and Enabling D			1557-8100	10.1089/omi.201	http://www.liebertpub.com/doi/10.1089/omi.2017.0214		2018-04	2021-10-12 2:57	2021-10-12 2:57		237-247		4	22						en	
IZ28ACF5	journalArticle	2018	Nelson, Eric, And	EPIC-CoGe: managing and analyzing genomic data			1367-4803, 1460	10.1093/bioinforr	https://academic.oup.com/bioinformatics/article/34/15/2651/4883494		2018-08-01	2021-10-12 2:57	2021-12-07 20:2		2651-2653		15	34						en	
P52RVYSA	journalArticle	2018	Wang, Doreen, L	SciApps: a cloud-based platform for reproducible bic			1367-4803, 1460	10.1093/bioinforr	https://academic.oup.com/bioinformatics/advance-article/doi/10.1093/bioinformatics/bty439/5036173		2018-06-12	2021-10-12 2:57	2021-12-07 20:2											en	
Z387JRVW	journalArticle	2018	Farley, John W, S	Situating Ecology as a Big-Data Science: Current Ac			0006-3568, 1525	10.1093/biosci/bi	https://academic.oup.com/bioscience/article/68/8/563/5049569		2018-08-01	2021-10-12 2:57	2021-12-07 20:2		563-576		8	68						en	
47F7MPUS	journalArticle	2018	Beardsley, James	Addressing Biological Informatics Workforce Needs:			0006-3568, 1525	10.1093/biosci/bi	https://academic.oup.com/bioscience/advance-article/doi/10.1093/biosci/biy116/5110140		2018-10-10	2021-10-12 2:57	2021-12-07 20:2											en	
RX9MS3IL	journalArticle	2018	Prewitt, Roisin C.	Cotton CENTRORADIALIS/TERMINAL FLOWER 1/			0022-0957, 1460	10.1093/jxb/ery3	https://academic.oup.com/jxb/advance-article/doi/10.1093/jxb/ery324/5093783		2018-09-10	2021-10-12 2:57	2021-12-07 20:2											en	
FWW29QFR	journalArticle	2018	Shaw, Marlene, A	Metrics matter: the effect of parasite richness, intens			0962-8452, 1471	10.1098/rspb.201	https://royalsocietypublishing.org/doi/10.1098/rspb.2018.2147		2018-11-21	2021-10-12 2:57	2021-12-07 20:3		20182147		1891	285						en	
78SXCBTR	journalArticle	2018	Concia, Linda, Lo	Genome-Wide Analysis of the Arabidopsis Replicatic			0032-0889, 1532	10.1104/pp.17.01	http://www.plantphysiol.org/lookup/doi/10.1104/pp.17.01537		2018-03	2021-10-12 2:57	2021-10-12 2:57		2166-2185		3	176						en	
LLVNQQTQ	journalArticle	2018	Zhang, Zuxin, Da	<i>GRF-interacting factor1</i> Regulates Shoot Arcl			1040-4651, 1532	10.1105/tpc.17.0(	http://www.plantcell.org/lookup/doi/10.1105/tpc.17.00791		2018-02	2021-10-12 2:57	2021-10-12 2:57		360-374		2	30						en	
9CTFNETV	conferencePaper	2018	Dewan, Nirav, Pr	Human-Centric Programming in th		978-1-5386-4235-1		10.1109/VLHCC.	https://ieeexplore.ieee.org/document/8506564/		2018-10	2021-10-12 2:57	2021-10-14 0:17		295-297										
LSFNX99Y	journalArticle	2018	Beckman, Rober	High dispersal ability is related to fast life-history stra			220477	10.1111/1365-27	http://doi.wiley.com/10.1111/1365-2745.12989		2018-07	2021-10-12 2:57	2021-10-12 2:57		1349-1362		4	106						en	
6SVXL2FW	journalArticle	2018	Park, Susan J., Is	Overlooked climate parameters best predict flowerin			13541013	10.1111/gcb.144	http://doi.wiley.com/10.1111/gcb.14447		2018-12	2021-10-12 2:57	2021-10-14 0:25		5972-5984		12	24						en	
ZUV6ZKJV	journalArticle	2018	Corrie, Felix, Bria	iReceptor: A platform for querying and analyzing ant			1052896	10.1111/imr.1266	http://doi.wiley.com/10.1111/imr.12666		2018-07	2021-10-12 2:57	2021-10-12 2:57		24-41		1	284						en	
RMB9NMNJ	journalArticle	2018	Šímová, Brian J.,	Spatial patterns and climate relationships of major p			3050270	10.1111/jbi.1317	http://doi.wiley.com/10.1111/jbi.13171		2018-04	2021-10-12 2:57	2021-10-12 2:57		895-916		4	45						en	
IW8QHTFZ	journalArticle	2018	Ohlrogge, Bertra	PlantFAdb: a resource for exploring hundreds of plar			9607412	10.1111/tpj.1410	http://doi.wiley.com/10.1111/tpj.14102		2018-12	2021-10-12 2:57	2021-10-14 0:25		1299-1308		6	96						en	
CWWEUTLK	journalArticle	2018	Ramírez-Gonzále	The transcriptional landscape of polyploid wheat			0036-8075, 1095	10.1126/science.	http://www.scienc	The coordinated expression of highly related homoeologous genes in polyploid species underlies the phenotypes c	2018-08-17	2021-10-12 2:57	2021-12-07 18:4		eaar6089		6403	361						en	
25MXG2L6	journalArticle	2018	Budnick, Clayton	Characterization of Three Small Proteins in <i>Bruce			0021-9193, 1098	10.1128/JB.0012	http://jb.asm.org/	ABSTRACT Elucidating the function of proteins <50 amino acids in length is no small task. Nevertheless, small pro	2018-07-02	2021-10-12 2:57	2021-12-07 20:2		e00127-18, /jb/200/18/e00127-18.a		18	200						en	
F52ETJSC	journalArticle	2018	Howard-Varona,	Whole-Genome Sequences of Phages p000v and p(			2576-098X	10.1128/MRA.01	http://genomea.a	ABSTRACT We report here the genome sequences and morphological characterizations of phages p000v and p00	2018-11-21	2021-10-12 2:57	2021-12-07 20:3		e01400-18, /mra/7/20/e01400-18.a		20	7						en	
RXWJ2FX3	conferencePaper	2018	Lowe, David Y., J	High Availability on Jetstream: Pra		978-1-4503-5863-7		10.1145/3217880	http://dl.acm.org/citation.cfm?doid=3217880.3217884		2018	2021-10-12 2:57	2021-10-12 2:57		7-Jan									en	
6CB9THX6	conferencePaper	2018	Wang, Doreen, L	SciApps: a Bioinformatics Workflov		978-1-4503-6446-1		10.1145/3219104	http://dl.acm.org/citation.cfm?doid=3219104.3219109		2018	2021-10-12 2:57	2021-10-12 2:57		5-Jan									en	

I7ZBGCAP	conferencePaper	2018	Dooley, John, Ri	The Agave Platform: An Open, Sci		978-1-4503-6446-1		10.1145/3219104	http://dl.acm.org/citation.cfm?doid=3219104.3219129		2018	2021-10-12 2:57	2021-10-12 2:57		8-Jan													en		
79UZE4RM	conferencePaper	2018	Breen, Ben, Joe;	Building the SLATE Platform		978-1-4503-6446-1		10.1145/3219104	http://dl.acm.org/citation.cfm?doid=3219104.3219144		2018	2021-10-12 2:57	2021-10-12 2:57		7-Jan													en		
BRHX36Y8	conferencePaper	2018	Cleveland, Gwen	Building Science Gateway Infrastru		978-1-4503-6446-1		10.1145/3219104	http://dl.acm.org/citation.cfm?doid=3219104.3219151		2018	2021-10-12 2:57	2021-10-12 2:57		8-Jan													en		
MPVFBQC6	conferencePaper	2018	Wang, Weijia, Yi	Authentication with User Driven W		978-1-4503-6446-1		10.1145/3219104	http://dl.acm.org/citation.cfm?doid=3219104.3229290		2018	2021-10-12 2:57	2021-10-12 2:57		7-Jan													en		
8RX5I6CG	journalArticle	2018	Ge, Runan, Stev	iDEP: an integrated web application for differential e			1471-2105	10.1186/s12859-	https://bmcbioinformatics.biomedcentral.com/articles/10.1186/s12859-018-2486-6		2018-12	2021-10-12 2:57	2021-10-14 0:18		534		1	19										en		
RSTZITKK	journalArticle	2018	Monson, Susan J	Immunomodulatory effects of heat stress and lipopol			1471-2164	10.1186/s12864-	https://bmcgenomics.biomedcentral.com/articles/10.1186/s12864-018-5033-y		2018-12	2021-10-12 2:57	2021-10-14 0:24		643		1	19										en		
XW5B8UC4	journalArticle	2018	Wang, Yingxiong	Evolutionary insights from comparative transcriptom			1471-2229	10.1186/s12870-	https://bmcplantbiol.biomedcentral.com/articles/10.1186/s12870-018-1429-8		2018-12	2021-10-12 2:57	2021-10-14 0:23		208		1	18										en		
TMCMT8AW	journalArticle	2018	Gregory, Brian C	Smoking is associated with quantifiable differences i			1465-993X	10.1186/s12931-	https://respiratory-research.biomedcentral.com/articles/10.1186/s12931-018-0878-9		2018-12	2021-10-12 2:57	2021-10-14 0:18		174		1	19										en		
KKD4T9NK	journalArticle	2018	Ogeron, Damien,	Palikur traditional roundwood construction in easterr			1746-4269	10.1186/s13002-	https://ethnobiomed.biomedcentral.com/articles/10.1186/s13002-018-0226-7		2018-12	2021-10-12 2:57	2021-10-12 2:57		28		1	14										en		
MQDHMUYB	journalArticle	2018	Miller, Edgar P., M	A machine vision platform for measuring imbibition o			1746-4811	10.1186/s13007-	https://plantmethods.biomedcentral.com/articles/10.1186/s13007-018-0383-7		2018-12	2021-10-12 2:57	2021-10-14 0:22		115		1	14										en		
H3KK2R3B	journalArticle	2018	Levy, J. Creighto	Transcriptome of Russet Norkotah and its clonal sel			1756-0500	10.1186/s13104-	https://bmcresnotes.biomedcentral.com/articles/10.1186/s13104-018-3254-4		2018-12	2021-10-12 2:57	2021-10-12 2:57		160		1	11										en		
KQFWS4UM	journalArticle	2018	AlKhalifah, Carol	Maize Genomes to Fields: 2014 and 2015 field seas			1756-0500	10.1186/s13104-	https://bmcresnotes.biomedcentral.com/articles/10.1186/s13104-018-3508-1		2018-12	2021-10-12 2:57	2021-10-12 2:57		452		1	11										en		
5C88P6K6	journalArticle	2018	Dalcin Martins, M	Viral and metabolic controls on high rates of microbi			2049-2618	10.1186/s40168-	https://microbiomejournal.biomedcentral.com/articles/10.1186/s40168-018-0522-4		2018-12	2021-10-12 2:57	2021-10-14 0:17		138		1	6										en		
3K9PCYDJ	journalArticle	2018	Katz, Jay, Daniel	The principles of tomorrow's university			2046-1402	10.12688/f1000re	https://f1000rese	In the 21st Century, research is increasingly data- and computation-driven. Researchers, funders, and the larger co	2018-12-11	2021-10-12 2:57	2021-12-07 20:3		1926			7										en		
8CNHJ98P	journalArticle	2018	Lotterhos, Ann E.	Analysis validation has been neglected in the Age of			1545-7885	10.1371/journal.p	http://dx.plos.org/10.1371/journal.pbio.3000070		2018-12-10	2021-10-12 2:57	2021-12-07 20:3		e3000070		12	16										en		
F9LYGZ38	journalArticle	2018	Brown, Steven B	Ten quick tips for sharing open genomic data			1553-7358	10.1371/journal.p	http://dx.plos.org/10.1371/journal.pcbi.1006472		2018-12-27	2021-10-12 2:57	2021-12-07 20:3		e1006472		12	14										en		
JZQCNT4G	journalArticle	2018	Park, Judith K., S	Low-phosphate-selected Auxenochlorella protothecc			1932-6203	10.1371/journal.p	https://dx.plos.org/10.1371/journal.pone.0198953		2018-06-19	2021-10-12 2:57	2021-12-07 20:2		e0198953		6	13										en		
FCAK4NMY	journalArticle	2018	Marizzi, David Ar	DNA barcoding Brooklyn (New York): A first assessm			1932-6203	10.1371/journal.p	https://dx.plos.org/10.1371/journal.pone.0199015		2018-07-18	2021-10-12 2:57	2021-12-07 20:2		e0199015		7	13										en		
T368F4YV	journalArticle	2018	Sari, Sabine, Ehs	Defense responses of lentil (Lens culinaris) genotyp			1932-6203	10.1371/journal.p	http://dx.plos.org/10.1371/journal.pone.0204124		2018-09-20	2021-10-12 2:57	2021-12-07 20:2		e0204124		9	13										en		
HA4LKYIF	journalArticle	2018	Hacisalihoglu, A.	Modulation of early maize seedling performance via			1932-6203	10.1371/journal.p	http://dx.plos.org/10.1371/journal.pone.0206861		2018-11-05	2021-10-12 2:57	2021-12-07 20:2		e0206861		11	13										en		
9WVG83LL	journalArticle	2018	Zhang, Susan J.,	Distinct genes and pathways associated with transcr			1932-6203	10.1371/journal.p	http://dx.plos.org/10.1371/journal.pone.0207715		2018-12-05	2021-10-12 2:57	2021-12-07 20:3		e0207715		12	13										en		
H6QUGJJV	journalArticle	2018	Nagaraj, Amulya	Emerging trend of big data analytics in bioinformatic			1744-5485, 1744	10.1504/IJBRA.2	http://www.inderscience.com/link.php?id=89175		2018	2021-10-12 2:57	2021-10-12 2:57		144		2-Jan	14										en		
PAW55GK5	journalArticle	2018	Molina, Nicole, J	DNA barcoding of online herbal supplements: crowd			2391-5412	10.1515/biol-201	http://www.degru	Abstract Herbal medicinal products (HMPs) have grown increasingly popular in the United States, many of them wi	2018-04-06	2021-10-12 2:57	2021-12-07 20:2		48-55		1	13												
WKFMCYZR	journalArticle	2018	Simakov, Thoma	A Workload Analysis of NSF's Innovative HPC Resources Using XDMoD					http://arxiv.org/ab	Workload characterization is an integral part of performance analysis of high performance computing (HPC) systen	2018-01-12	2021-10-12 2:57	2021-12-07 20:2																	
WLUG5XBS	journalArticle	2018	Atallah, Jo Ann, N	Sex Determination in <i>Ceratopteris richardii</i> Is			2160-1836	10.1534/g3.118.2	http://g3journal.org/lookup/doi/10.1534/g3.118.200292		2018-07	2021-10-12 2:57	2021-10-12 2:57		2205-2214		7	8										en		
LS3GX4XR	journalArticle	2018	Selvakumar, Chir	Genomic Advances in Root Vegetable Crops			23197692, 23197	10.20546/ijcmas.	https://www.ijcmas.com/abstractview.php?ID=10420&vol=7-10-2018&SNo=308		2018-10-20	2021-10-12 2:57	2021-12-07 20:2		2649-2660		10	7												
TWKZTH5A	journalArticle	2018	Shaibu, R., A. A.;	Screening African Rice ( ) for Tolerance to Abiotic St			0011-183X	10.2135/cropsci2	https://dl.sciencesocieties.org/publications/cs/abstracts/58/1/133		2018	2021-10-12 2:57	2021-10-12 2:57		133		1	58										en		
4HNVRT8A	journalArticle	2018	Rosenfield, Alyss	AAS WorldWide Telescope: Seamless, Cross-Platfo			1538-4365	10.3847/1538-43	http://arxiv.org/ab	The American Astronomical Society's WorldWide Telescope (WWT) project enables terabytes of astronomical imag	2018-05-11	2021-10-12 2:57	2021-12-07 20:2		22		1	236												
CFGKI64S	journalArticle	2018	Swetnam, Jason	Considerations for Achieving Cross-Platform Point C			1664-462X	10.3389/fpls.201	http://journal.frontiersin.org/article/10.3389/fpls.2017.02144/full		2018-01-10	2021-10-12 2:57	2021-12-07 20:2		2144			8												
8WV7PX38	journalArticle	2018	Heidorn, Julie, P.	Astrolabe: Curating, Linking and Computing Astrono			1538-4365	10.3847/1538-43	http://arxiv.org/ab	Where appropriate repositories are not available to support all relevant astronomical data products, data can fall in	2018-05-11	2021-10-12 2:57	2021-12-07 20:2		3		1	236												
8R5U7DZT	journalArticle	2018	Afgan, Nuwan, E	CloudLaunch: Discover and Deploy Cloud Applications					http://arxiv.org/ab	Cloud computing is a common platform for delivering software to end users. However, the process of making comp	2018-05-10	2021-10-12 2:57	2021-12-07 20:2																	
Q22QQ6NJ	journalArticle	2018	Stahlman, Julie,	The Astrolabe Project: Identifying and Curating Astro			2100-014X	10.1051/epjconf/	http://arxiv.org/ab	As research datasets and analyses grow in complexity, data that could be valuable to other researchers and to sup	2018	2021-10-12 2:57	2021-10-12 2:57		3003			186												
FUN7PT9Y	journalArticle	2018	Spalding, Jordan	Towards controlled Fizeau observations with the Large Binocular Telescope					http://arxiv.org/ab	The Large Binocular Telescope Interferometer (LBTI) can perform Fizeau interferometry in the focal plane, which a	2018-06-15	2021-10-12 2:57	2021-12-07 20:2																	
UHFHBTS4	journalArticle	2018	Proctor, Joseph,	Virtualizing the Stampede2 Supercomputer with Applications to HPC i				10.1145/3219104	http://arxiv.org/ab	Methods developed at the Texas Advanced Computing Center (TACC) are described and demonstrated for automa	2018	2021-10-12 2:57	2021-10-12 2:57		6-Jan															
PZCV3VYL	journalArticle	2018	Gadelha Jr., Fabi	A Survey of e-Biodiversity: Concepts, Practices, and Challenges					http://arxiv.org/ab	The unprecedented size of the human population, along with its associated economic activities, have an ever incre	2018-09-29	2021-10-12 2:57	2021-12-07 20:2																	
34MIU9BE	journalArticle	2018	Stucky, Ramona,	The Plant Phenology Ontology: A New Informatics R			1664-462X	10.3389/fpls.201	http://journal.frontiersin.org/article/10.3389/fpls.2018.00517/full		2018-05-01	2021-10-12 2:57	2021-12-07 20:2		517			9												
8Q8QAS26	journalArticle	2018	Lent, Eric, Heath	Science Citation Knowledge Extractor			2504-0537	10.3389/frma.201	https://www.frontiersin.org/article/10.3389/frma.2018.00035/full		2018-12-18	2021-10-12 2:57	2021-12-07 20:3		35			3												
QHTZ5355	journalArticle	2018	Xu, Jennifer, Zhe	Existing and Potential Statistical and Computational			2073-4395	10.3390/agronom	http://www.mdpi.com/2073-4395/8/5/71		2018-05-14	2021-10-12 2:57	2021-12-07 20:2		71		5	8										en		
LDZZMB5S	journalArticle	2018	Howard-Varona,	Fighting Fire with Fire: Phage Potential for the Treat			2079-6382	10.3390/antibioti	http://www.mdpi.c	Hemolytic–uremic syndrome is a life-threating disease most often associated with Shiga toxin-producing microorga	2018-11-16	2021-10-12 2:57	2021-12-07 20:2		101		4	7										en		
52R3EUZM	journalArticle	2018	Orozco-Arias, Ro	Inpactor, Integrated and Parallel Analyzer and Class			2079-7737	10.3390/biology7	http://www.mdpi.com/2079-7737/7/2/32		2018-05-25	2021-10-12 2:57	2021-12-07 20:2		32		2	7										en		
3LIRXNF5	journalArticle	2018	Maggio, Dae-Jin,	It's Hard to Avoid Avoidance: Uncoupling the Evoluti			1422-0067	10.3390/ijms191	http://www.mdpi.c	In the last 100 years, agricultural developments have favoured selection for highly productive crops, a fact that has	2018-11-20	2021-10-12 2:57	2021-12-07 20:3		3671		11	19										en		
N38FCQVY	journalArticle	2018	Inostroza, Patrici	Understanding the Complexity of Cold Tolerance in V			1940-3372	10.3835/plantger	https://dl.sciencesocieties.org/publications/tpg/abstracts/11/3/170096		2018	2021-10-12 2:57	2021-10-14 0:26		0		3	11										en		
D636L9M7	journalArticle	2018	Endara, Hong, Lo	Modifier Ontologies for frequency, certainty, degree,			1314-2828, 1314	10.3897/BDJ.6.e	https://bdj.pensof	Background: When phenotypic characters are described in the literature, they may be constrained or clarified with a	2018-11-28	2021-10-12 2:57	2021-12-07 20:3		e29232			6												
GEBD85CR	journalArticle	2018	Cox, Ramona, Si	Specimen Identifiers in Related Disciplines: What ca			2535-0897	10.3897/biss.2.2	https://biss.pensc	Life sciences research, and even more specifically biodiversity sciences research, has yet to coalesece on a single	2018-05-21	2021-10-12 2:57	2021-12-07 20:2		e26615			2												
4YSG53EC	journalArticle	2018	Guralnick, David,	The Death of VertNet Has Been Greatly Exaggerate			2535-0897	10.3897/biss.2.2	https://biss.pensc	VertNet (vertnet.org) is a collaborative project that makes biodiversity data free and available on the web. VertNet is	2018-05-22	2021-10-12 2:57	2021-12-07 20:2		e26836			2												
XAHPCR9H	report	2018	Bagley, Steven H	Genotyping-by-sequencing and ecological niche modeling illuminate phylogeography, a					https://peerj.com	Populus tremuloides is the widest-ranging tree species in North America, and an ecologically important component	2018-08-29	2021-10-12 2:57	2021-12-07 20:2															en		
CIYTILTF	journalArticle	2018	Rivera, Jo Anne,	Genome analysis of the ubiquitous boxwood pathog			2167-8359	10.7717/peerj.54	https://peerj.com	Boxwood (Buxus spp.) are broad-leaved, evergreen landscape plants valued for their longevity and ornamental qu	2018-08-24	2021-10-12 2:57	2021-12-07 18:4		e5401			6										en		
Q2UMQA8B	journalArticle	2018	Borton, Julie M;	Coupled laboratory and field investigations resolve microbial interactions that underpin persistence in hydraulically fractured shales							2018	2021-10-12 2:57	2021-10-12 2:57		E6585–E6594		28	115												
UM8B8QAE	thesis	2018	Linero, ARPAT, W	Seed Dispersal by Chelonians: From Individuals to Communities							2018	2021-10-12 2:57	2021-10-12 2:57																	
C3CDIYWH	thesis	2018	Lorant, Anne	Plasticity and genetic adaptation as contributors to the evolutionary history of cultivated maize and its wild relatives							2018	2021-10-12 2:57	2021-10-12 2:57																	
65NQ5KLZ	thesis	2018	Lampe, Robert H	Distinct Strategies by Bloom-forming Diatoms to Frequently-encountered Shifts in Their Environment							2018	2021-10-12 2:57	2021-10-12 2:57																	
H8HUVX49	thesis	2018	Zhou, Shaoqun	Genetic and Physiological Regulation of Specialized Metabolism and Biochemical Defense in Zea mays							2018	2021-10-12 2:57	2021-10-14 0:33																	
8FEZKBCX	conferencePaper	2018	Merchant, Tyson	CyVerse: a Ten-year Perspective on Cyberinfrastructure Development, Collaboration, a					http://adsabs.harvard.edu/abs/2018AGUFMIN23B0767S		2018	2021-10-12 2:57	2021-10-12 2:57					IN23B-0767												
RTLHQ397	conferencePaper	2018	Davies, Rich, , N	Blockchain-enabled Data Trusts for Futuromics Research					http://adsabs.harvard.edu/abs/2018AGUFMIN53A..08D		2018	2021-10-12 2:57	2021-10-12 2:57					IN53A-08												
QCW9JYLE	conferencePaper	2018	Hazekamp, Doug	MAKER as a Service: Moving HPC applications to Jetstream Cloud					http://ccl.cse.nd.edu/research/papers/maker-service-ic2e2018.pdf		2018	2021-10-12 2:57	2021-10-12 2:57																	
CIQEMI59	conferencePaper	2018	Egyedi, Mark A.,	Using Semantic Technologies to Enhance Metadata Submissions to Public Repositories					http://ceur-ws.org/Vol-2275/paper1.pdf		2018	2021-10-12 2:57	2021-10-12 2:57		1			2275												
552MI3HS	conferencePaper	2018	Trigkakis, B., , Di	Planteome & BisQue: Automating Image Annotation with Ontologies using Deep-Learni					http://ceur-ws.org/Vol-2285/ICBO_2018_paper_11.pdf		2018	2021-10-12 2:57	2021-10-12 2:57					2285, No. 11												
Z6PXQCC2	conferencePaper	2018	Gupta, Jennifer, ,	Domain Informational Vocabulary Extraction Experiences with Publication Pipeline Integ					http://ceur-ws.org/Vol-2285/ICBO_2018_paper_43.pdf		2018	2021-10-12 2:57	2021-10-12 2:57					2285, No. 43												
C73T6NPY	conferencePaper	2018	Stubbs, Julia, , J	Rapid Development of Scalable, Distributed Computation with Abaco					http://ceur-ws.org/Vol-2357/paper3.pdf		2018	2021-10-12 2:57	2021-10-12 2:57																	
E9DMVIDZ	conferencePaper	2018	Dooley, Sean, Ri	Accelerating Gateway Development with Agave ToGo Webapps and Microsites					http://ceur-ws.org/Vol-2357/paper4.pdf		2018	2021-10-12 2:57	2021-10-12 2:57																	
65GNQ2AT	thesis	2018	Hubbard, Allen H	Data and -omics-driven approaches to understand the heat stress response: the develo					http://udspace.udel.edu/bitstream/handle/19716/23816/Hubbard_udel_0060D_13357.pdf?sequence=1&isAllowed=y		2018	2021-10-12 2:57	2021-10-12 2:57																	
ZB4AIQT3	conferencePaper	2018	Spalding, Edgar	Helping the Community Transform High-Throughput Imaging Data into Phenotype Infor					http://www.intlpag.org/2018/images/pdf/PAGXXVI-abstracts-workshops.pdf		2018	2021-10-12 2:57	2021-10-12 2:57					W064												
EI34CKM8	report	2018	Hamm, Elena Lo	Biogeography of Gracilaria species in Waquoit Bay					http://www.mbl.edu/ses/files/2018/02/Hamm_Lauren.pdf		2018	2021-10-12 2:57	2021-10-12 2:57																	
WN3U3YKX	conferencePaper	2018	Reddy, Tanner, ,	RAPTORS: Hyperspectral Survey of the GEO Belt					https://amostech.com/TechnicalPapers/2018/NROC/Reddy.pdf		2018	2021-10-12 2:57	2021-10-12 2:57																	
55RI9DYZ	bookSection	2018	Ndulo, Steve, , M	Evaluating the NEPAD Resource Mobilization Strategy					https://books.google.com/books?hl=en&lr=lang_en&id=7PhVDwAAQBAJ&oi=fnd&pg=PA74&dq=%22iPlant+Collaborative%22&ots=		2018	2021-10-12 2:57	2021-10-12 2:57		88															
DYCHD3RS	conferencePaper	2018	Joyce, Blake	Image Analysis using CyVerse					https://community.plantae.org/files/posts/5044038905985238342/32510ad6914bb14e0dd8290b4f55866c_Phenome%202018%20Sp		2018	2021-10-12 2:57	2021-10-12 2:57																	
VRYMZ9SN	conferencePaper	2018	Hirsh, Corey	Machine vision phenotyping platform for seedling growth and morphology					https://community.plantae.org/files/posts/5044038905985238342/32510ad6914bb14e0dd8290b4f55866c_Phenome%202018%20Sp		2018	2021-10-12 2:57	2021-10-12 2:57																	
6WIY5K8S	conferencePaper	2018	Miller, Nathan	A Machine-Vision Seedling Emergence Assay					https://community.plantae.org/files/posts/5044038905985238342/32510ad6914bb14e0dd8290b4f55866c_Phenome%202018%20Sp		2018	2021-10-12 2:57	2021-10-12 2:57																	
I6NJTPNH	conferencePaper	2018	Swetnam, Tyson	Saguaro Cactus - 5					https://community.plantae.org/files/posts/5044038905985238342/32510ad6914bb14e0dd8290b4f55866c_Phenome%202018%20Sp		2018	2021-10-12 2:57	2021-10-12 2:57																	
M2T3PCV6	conferencePaper	2018	Enders, Edgar, ,	Machine vision phenotyping platform for seedling growth and morphology					https://community.plantae.org/files/posts/5044038905985238342/32510ad6914bb14e0dd8290b4f55866c_Phenome%202018%20Sp		2018	2021-10-12 2:57	2021-10-12 2:57																	
B9I5KSK8	journalArticle	2018	Fischer, Sanjana	Introduction to jetstream					https://dl.acm.org/citation.cfm?id=3280519		2018	2021-10-12 2:57	2021-10-12 2:57		189-191			34, No. 1												
ZSX9C2BG	journalArticle	2018	Prabhakar, Kumk	Reaffirming the Power of Nature's Pharmacy through Pharmacognosy					https://macub.org/wp-content/uploads/2018/09/Fall-2018-In-Vivo.pdf		2018	2021-10-12 2:57	2021-10-12 2:57		19-23			40, No. 1												
JQ9DTKIJ	journalArticle	2018	Firrao, Giuseppe	Genomic Structu	Frontiers in Microbiology		1664-302X	10.3389/fmicb.20	http://journal.frontiersin.org/article/10.3389/fmicb.2018.00656/full		2018-04-05	2021-10-12 22:5	2021-12-07 20:2	2021-10-12 22:5	656			9		Front. Microbiol.										
AXCWDZNB	journalArticle	2018	Manching, Heath	Maize Phyllosphe	F1000Research		2046-1402	10.12688/f1000re	https://f1000rese	Background : The phyllosphere hosts a variety of microorganisms, including bacteria, which can play a po	2018-01-18	2021-10-12 23:1	2021-12-07 20:1	2021-10-12 23:1	1698			6		F1000Res								en		
PTP2KUCQ	bookSection	2018	Tang, Haibao; Zh	From Alpha-Duplication to Triplication and Sextuplication					http://link.springer.com/10.1007/978-3-319-43694-4{_}5		2018	2021-10-12 23:3	2021-10-12 23:3		99–109											Springer, Cham				
BW9GPV7V	thesis	2018	Patterson, Eric L	The Genetics and Genomics of Herbicide Resistant Kochia scoparia					https://search.pro	Weed genomics resources lag behind other plant biology disciplines despite larger annual crop losses occurring du	2018	2021-10-12 23:3	2021-10-12 23:3													Colorado State University				PhD Thesis
TX59TFQQ	conferencePaper	2018	Reddy, Vishnu; F	Synergistic Neo-I	Proceedings of the 1st NEO and Debris Detection Conference					The University of Arizona (UoA) is a world leader in the detection and characterization of near-Earth objects (NEOs	2018	2021-10-12 23:3	2021-10-12 23:3		6												Darmstadt, Germany			
EJLPEAYQ	journalArticle	2018	Fischer, Jeremy;	Introduction to Je	J. Comput. Sci. Coll.		1937-4771		http://dl.acm.org/citation.cfm?id=3280489.3280519		2018-10	2021-10-12 23:3	2021-10-14 0:18		189–191		1	34												
XT28IM59	thesis	2018	Boggess, Erin E.	Methods for analysis of derivative strains from metabolic evolution experiments						One of the largest challenges in genomics studies is determining the relationship between genotype and phenotyp	2018	2021-10-12 23:3	2021-10-12 23:3			178										Iowa State University				PhD Thesis
S5X86WHI	thesis	2018	West, Donnelly A	The Evolution of Responses to Abiotic Factors in Leaves and Their Specialized Cell Typ					http://ezproxy.libr	The plant kingdom encompasses over 400,000 species which span the globe (Kew Royal Botanical Gardens, 2017	2018	2021-10-12 23:3	2021-10-12 23:3			181										University of Arizona				PhD Thesis
TZ2MX835	thesis	2018	Gladstein, Ariella	Inference of Recent Demographic History of Population Isolates Using Genome-Wide H					https://search.pro	In this dissertation I addressed the problem of SNP array bias when finding runs of ho- mozygosity. I demonstrated	2018	2021-10-12 23:3	2021-10-12 23:3													University of Arizona				PhD Thesis
MCYP8XBL	thesis	2018	Chapa, Mario Xa	A Cyberinfrastructure Plat Form for the Modeling and Optimization of Biomass Logistics					https://search.pro	This thesis presents the development of a web-based decision support system for the purpose of aiding researche	2018	2021-10-12 23:3	2021-10-12 23:3													The University of Texas at San Antonio				PhD Thesis
YLRMEWZV	bookSection	2018	Putonti, Catherin	Freshwater Viromes: From Sampling to Evaluation					http://link.springer.com/10.1007/978-1-4939-8728-3{_}2		2018	2021-10-12 23:3	2021-10-12 23:3		17–27											Humana Press, New York, NY				
4CMTDYE6	thesis	2018	He, Mingze	Analysis of G-quadruplexes as environmental sensors: Novel statistical models and con					https://search.pro	The occurrence of G-quadruplex (G4) structures in both genic and non-genic sequences has been well documente	2018	2021-10-12 23:3	2021-10-14 19:2													Iowa State University				PhD Thesis
NS3QYHE3	thesis	2018	Falćon, Wilfredo	Seed dispersal by chelonians: From individuals to communities					https://www.resea	Plants are sessile for the most of their life cycle, but their gametes (pollen) and their propagules (seeds) can move	2018	2021-10-12 23:3	2021-10-12 23:3													University of Zurich				PhD Thesis
SQTPSQMG	bookSection	2018	McCallum, John;	Molecular Mapping of Genes and QTL: Progress to Date and Development of New Pop					http://link.springer.com/10.1007/978-3-319-95825-5{_}13		2018	2021-10-12 23:3	2021-10-12 23:3		181–196											Springer, Cham				
HYJ5SZER	journalArticle	2018	Davies, N.; Deck	Blockchain-enabl	American Geophysical Union, Fall Meeting 2018, abstract #IN53A-08				http://adsabs.harvard.edu/abs/2018AGUFMIN53A..08D		2018	2021-10-12 23:3	2021-10-12 23:3																	
BUN92HMV	journalArticle	2018	Bagley, Justin C;	Genotyping-by-sequencing and ecological niche mo			2167-9843	10.7287/peerj.pre	https://peerj.com/preprints/27162/		2018-08	2021-10-12 23:3	2021-10-12 23:3																	
NUWT6HQQ	journalArticle	2018	Roberts, Steven	Opportunities in I	Journal of Shellfish Research		0730-8000	10.2983/035.037	http://www.bioone	As computational and sequencing technologies continue to flourish, the barrier for those interested in complementi	2018-10	2021-10-12 23:3	2021-10-12 23:3		747–754		4	37												
K39XR4AP	conferencePaper	2018	de Vila, Milton H	MULTI-X, a State	2018 IEEE Intern	978-1-5386-5488-0		10.1109/BIBM.20	https://ieeexplore.ieee.org/document/8621317/		2018-12	2021-10-12 23:3	2021-10-12 23:3		1726–1733											IEEE				
YIJPT3JW	journalArticle	2018	Gadelha, Luiz M.	A Survey of e-Biodiversity: Concepts, Practices, and Challenges					http://arxiv.org/ab	The unprecedented size of the human population, along with its associated economic activities, have an ever incre	2018-09	2021-10-12 23:3	2021-10-12 23:3																	
DRB5DTBE	journalArticle	2018	Swetnam, T. L.; V	CyVerse: a Ten-y	American Geophysical Union, Fall Meeting 2018, abstract #IN23B-076				http://adsabs.harvard.edu/abs/2018AGUFMIN23B0767S		2018	2021-10-12 23:3	2021-10-12 23:3																	
NZUBCJP5	conferencePaper	2018	Trigkakis, Dimitri	Planteome & Bis	Proceedings of the 9th International Conference on Biological Ontolog				http://ceur-ws.org	The field of computer vision has recently experienced tremendous progress due to advances in deep learning [1]. T	2018	2021-10-12 23:3	2021-10-12 23:3		2285, No. 11												Corvallis, Oregon, USA			
63Y9U6IB	conferencePaper	2018	Gupta, Amit; Xu,	Domain Informati	Proceedings of the 9th International Conference on Biological Ontology (ICBO 2018)					We will present updates on an ongoing project DIVE (Domain Informational Vocabulary Extraction), a system desig	2018	2021-10-12 23:3	2021-10-12 23:3		2285, No. 43												Corvallis, Oregon, USA			
58GE7XF9	thesis	2018	Hubbard, Jr., Alle	Data and -omics-driven approaches to understand the heat stress response: the develo					https://udspace.u	This dissertation concerns the broad computational challenges that face labs in the -omics era, in the service of ad	2018	2021-10-12 23:3	2021-10-14 19:2													University of Delaware				PhD Thesis
I3MMRTI2	conferencePaper	2018	Reddy, Vishnu; L	RAPTORS: Hype	In The Advanced Maui Optical and Space Surveillance Technologies (				https://amostech.	Robotic Automated Pointing Telescopes for Optical Reflectance Spectroscopy (RAPTORS) is an automated 0.6- m	2018	2021-10-12 23:3	2021-10-12 23:3																	
7GHV95K6	journalArticle	2017	Moller, Chun, Abi	MetaCRAST: reference-guided extraction of CRISPR spacers from unassembled metagenomes							2017	2021-10-12 2:33	2021-10-12 2:33		e3788			5												
UZSBDC2Q	journalArticle	2017	Santana-Perez, (	Reproducibility of execution environments in comput			0167739X	10.1016/j.future.2	https://linkinghub.elsevier.com/retrieve/pii/S0167739X16000029		2017-02	2021-10-12 2:51	2021-10-12 2:51		354-367			67										en		
KIVAWFKP	journalArticle	2017	Jamil, Hasan M.	A Visual Interface for Querying Heterogeneous Phyl			1545-5963	10.1109/TCBB.20	http://ieeexplore.ieee.org/document/7390046/		2017-01-01	2021-10-12 2:51	2021-12-07 20:0		131-144		1	14												
KP4LWZNR	journalArticle	2017	Weber, Jonas	Idiomatic and Reproducible Software Builds using Containers for Reliable Computing					http://arxiv.org/ab	Containers as the unit of application delivery are the 'next big thing' in the software development world. They enabl	2017-02-09	2021-10-12 2:51	2021-12-07 20:0																	
TRGS4362	journalArticle	2017	Makarevitch, Bet	Killing two birds with one stone: Model plant systems			18749399	10.1016/j.bbagrm	https://linkinghub.elsevier.com/retrieve/pii/S1874939916300803		2017-01	2021-10-12 2:51	2021-10-12 2:51		166-173		1	1860										en		
MFJI64DS	journalArticle	2017	Howard-Varona,	Regulation of infection efficiency in a globally abund			1751-7362, 1751	10.1038/ismej.20	http://www.nature.com/articles/ismej201681		2017-01	2021-10-12 2:51	2021-10-12 2:51		284-295		1	11										en		
69E2MSZ5	journalArticle	2017	Weirauch, Pamel	Areas of endemism in the Nearctic: a case study of			7483007	10.1111/cla.1216	http://doi.wiley.com/10.1111/cla.12169		2017-06	2021-10-12 2:51	2021-10-12 2:51		279-294		3	33										en		
E2TVXNVB	journalArticle	2017	Bolduc, Matthew	iVirus: facilitating new insights in viral ecology with s			1751-7362, 1751	10.1038/ismej.20	http://www.nature.com/articles/ismej201689		2017-01	2021-10-12 2:51	2021-10-12 2:51		14-Jul		1	11										en		
DEBIVPW9	journalArticle	2017	Muhammad, Terr	More than meets the eye: Emergent properties of tra			18749399	10.1016/j.bbagrm	https://linkinghub.elsevier.com/retrieve/pii/S1874939916301675		2017-01	2021-10-12 2:51	2021-10-12 2:51		64-74		1	1860										en		
79W4H3XW	journalArticle	2017	Dinkins, Christop	Transcriptome response of <i>Lolium arundinaceum			0028646X	10.1111/nph.141	http://doi.wiley.com/10.1111/nph.14103		2017-01	2021-10-12 2:51	2021-10-12 2:51		324-337		1	213										en		

QVTHFSY5	bookSection	2017	Hacker, Shirley, T	Cyberinfrastructure to Empower Sc	978-3-319-41215-3 978-3-319-41217-7			http://link.springer.com/10.1007/978-3-319-41217-7_26		2017	2021-10-12 2:51	2021-10-12 2:51	483-495			en
7EEXKE2S	journalArticle	2017	Williams, Tracy K	A vision for collaborative training infrastructure for bi		778923	10.1111/nyas.132	http://doi.wiley.com/10.1111/nyas.13207		2017-01	2021-10-12 2:51	2021-10-12 2:51	54-60	1	1387	en
9UW9LJDW	report	2017	D'Agostino, Andr	A microservice-based portal for X-ray transient and variable sources				https://peerj.com	Modern soft X-ray observatories can yield unique insights into time domain astrophysics, and a huge amount of inf	2017-06-30	2021-10-12 2:51	2021-12-07 20:03				en
NQLFZ99W	journalArticle	2017	Gnimpieba, Caro	Bio-TDS: bioscience query tool discovery system		0305-1048, 1362	10.1093/nar/gkw	https://academic.oup.com/nar/article-lookup/doi/10.1093/nar/gkw940		2017-01-04	2021-10-12 2:51	2021-12-07 20:02	D1117-D1122	D1	45	en
5SELVZTW	journalArticle	2017	Yang, Yun, Chao	Utilizing Cloud Computing to address big geospatial		1989715	10.1016/j.compe	https://linkinghub.elsevier.com/retrieve/pii/S0198971516303106		2017-01	2021-10-12 2:51	2021-10-12 2:51	120-128		61	en
9BJQHD9I	journalArticle	2017	Belete, Gerard F.	An overview of the model integration process: From		13648152	10.1016/j.envsoft	https://linkinghub.elsevier.com/retrieve/pii/S1364815216308805		2017-01	2021-10-12 2:51	2021-10-12 2:51	49-63		87	en
LNPBHTTC	report	2017	Shahand, Sílvia,	Rosemary: a flexible programming framework to build Science Gateways				https://peerj.com	The lessons learned during six years of experience in design, development, and operation of four Science Gatewa	2017-02-18	2021-10-12 2:51	2021-12-07 20:03				en
UFYN29FH	journalArticle	2017	Janssen, John M	Towards a new generation of agricultural system dat		0308521X	10.1016/j.agsy.20	https://linkinghub.elsevier.com/retrieve/pii/S0308521X16305637		2017-07	2021-10-12 2:51	2021-10-12 2:51	200-212		155	en
YG7U6N6H	journalArticle	2017	König, Holger, Cl	Dissecting global turnover in vascular plants: Global		1466822X	10.1111/geb.125	http://doi.wiley.com/10.1111/geb.12536		2017-02	2021-10-12 2:51	2021-10-12 2:51	228-242	2	26	en
JE7NXB8Q	journalArticle	2017	Schneweis, Dorit	Thrips developmental stage-specific transcriptome r		426822	10.1016/j.virol.20	https://linkinghub.elsevier.com/retrieve/pii/S0042682216303038		2017-01	2021-10-12 2:51	2021-10-12 2:51	226-237		500	en
ZQXZ7EQN	journalArticle	2017	Miller, Edgar P., M	A robust, high-throughput method for computing mai		9607412	10.1111/tpj.1332	http://doi.wiley.com/10.1111/tpj.13320		2017-01	2021-10-12 2:51	2021-10-12 2:51	169-178	1	89	en
Z3QZVCVH	journalArticle	2017	Brozynska, Robe	Sequencing of Australian wild rice genomes reveals		14677644	10.1111/pbi.1267	http://doi.wiley.com/10.1111/pbi.12674		2017-06	2021-10-12 2:51	2021-10-12 2:51	765-774	6	15	en
8BBHEDBM	journalArticle	2017	Ahlgren, Fengzhu	Alignment-free d_2^* oligonucleotide frequency di		0305-1048, 1362	10.1093/nar/gkw	https://academic.	Abstract Viruses and their host genomes often share similar oligonucleotide frequency (ONF) patterns, which can b	2017-01-09	2021-10-12 2:51	2021-12-07 20:02	39-53	1	45	en
LFFEBLWC	bookSection	2017	Joshi, Dong, Truj	The Evolution of Soybean Knowled	978-1-4939-6656-1 978-1-4939-6658-5			http://link.springer.com/10.1007/978-1-4939-6658-5_7		2017	2021-10-12 2:51	2021-10-12 2:51	149-159		1533	en
IFHP3G44	conferencePaper	2017	Esteva, Ramona	Identifier services: tracking objects and metadata across time and distributed storage systems						2017	2021-10-12 2:51	2021-10-12 2:51	85–86			
MR3WMAQ5	journalArticle	2017	Craig, Paul A.	A survey on faculty perspectives on the transition to		14708175	10.1002/bmb.210	http://doi.wiley.com/10.1002/bmb.21060		2017-09	2021-10-12 2:55	2021-10-12 2:55	426-436	5	45	en
E3BK6I7Y	journalArticle	2017	Agnew, Christoph	Whole‐Plant Manual and Image‐Based Phenotyping		2379-8068, 2379	10.1002/cppb.20	https://onlinelibrary.wiley.com/doi/abs/10.1002/cppb.20044		2017-03	2021-10-12 2:55	2021-10-12 2:55	21-Jan	1	2	en
Y7ERKXBY	journalArticle	2017	Swetnam, Erika L	Topographically driven differences in energy and wa		21508925	10.1002/ecs2.17	http://doi.wiley.com/10.1002/ecs2.1797		2017-04	2021-10-12 2:55	2021-10-12 2:55	e01797	4	8	en
JJJS6C45	journalArticle	2017	Grover, Eric, Jeff	CoGe LoadExp+: A web-based suite that integrates		24754455	10.1002/pld3.8	http://doi.wiley.com/10.1002/pld3.8		2017-07	2021-10-12 2:55	2021-10-12 2:55		2	1	en
VZYYUE2M	bookSection	2017	Sparks, Philip N.	Tissue-Specific Transcriptome Pro	978-1-4939-7001-8 978-1-4939-7003-2			http://link.springer.com/10.1007/978-1-4939-7003-2_8		2017	2021-10-12 2:55	2021-10-12 2:55	107-122		1610	en
NE32E7VP	bookSection	2017	Singh, Amit Kum	Discovery and Role of Molecular M	978-3-319-67155-0 978-3-319-67156-7			http://link.springer.com/10.1007/978-3-319-67156-7_12		2017	2021-10-12 2:55	2021-10-12 2:55	303-328			en
D6K7NY8W	journalArticle	2017	Reyer, Klaus, He	Exploring the genetics of feed efficiency and feeding		1617-4615, 1617	10.1007/s00438-	http://link.springer.com/10.1007/s00438-017-1325-1		2017-10	2021-10-12 2:55	2021-10-12 2:55	1001-1011	5	292	en
ILQCCMDA	journalArticle	2017	Chao, Michael E.	Comparison of phytohormone levels and transcript p		0167-4412, 1573	10.1007/s11103-	http://link.springer.com/10.1007/s11103-017-0607-7		2017-06	2021-10-12 2:55	2021-10-12 2:55	281-302	3	94	en
YYBTWPQU	journalArticle	2017	Nualart, Jordi, Ne	Assessing the Relevance of Herbarium Collections a		0006-8101, 1874	10.1007/s12229-	http://link.springer.com/10.1007/s12229-017-9188-z		2017-09	2021-10-12 2:55	2021-10-12 2:55	303-325	3	83	en
KT7IGP8W	journalArticle	2017	Lamsal, Pabodha	Efficient crop model parameter estimation and site c		0308521X	10.1016/j.agsy.20	https://linkinghub.elsevier.com/retrieve/pii/S0308521X17301464		2017-10	2021-10-12 2:55	2021-10-12 2:55	170-184		157	en
K85R83FW	journalArticle	2017	Brasil, Michele R	Microalgae and cyanobacteria as enzyme biofactorie		22119264	10.1016/j.algal.20	https://linkinghub.elsevier.com/retrieve/pii/S2211926417304460		2017-07	2021-10-12 2:55	2021-10-12 2:55	76-89		25	en
CXVIW6HT	journalArticle	2017	Coppens, Stijn, F	Unlocking the potential of plant phenotyping data thr		24523100	10.1016/j.coisb.2	https://linkinghub.elsevier.com/retrieve/pii/S2452310017300069		2017-08	2021-10-12 2:55	2021-10-12 2:55	58-63		4	en
RHPTF5P5	journalArticle	2017	Geniza, Pankaj, I	Tools for building de novo transcriptome assembly		22146628	10.1016/j.cpb.20	https://linkinghub.elsevier.com/retrieve/pii/S2214662817301032		2017-09	2021-10-12 2:55	2021-10-12 2:55	41-45		12-Nov	en
WCKHP3JX	journalArticle	2017	Yang, Joshua W.	Scalability and Validation of Big Data Bioinformatics		20010370	10.1016/j.csbj.20	https://linkinghub.elsevier.com/retrieve/pii/S200103701730048X		2017	2021-10-12 2:55	2021-10-12 2:55	379-386		15	en
RJHQTSR9	journalArticle	2017	Tardieu, Malcolm	Plant Phenomics, From Sensors to Knowledge		9609822	10.1016/j.cub.20	https://linkinghub.elsevier.com/retrieve/pii/S0960982217306218		2017-08	2021-10-12 2:55	2021-10-12 2:55	R770-R783	15	27	en
4Z3ZADK3	journalArticle	2017	Jin, Dawn C., Xic	A prototype cloud-based reproducible data analysis		13648152	10.1016/j.envsoft	https://linkinghub.elsevier.com/retrieve/pii/S1364815216310428		2017-10	2021-10-12 2:55	2021-10-12 2:55	172-180		96	en
5ZSKKKG8	journalArticle	2017	Bolger, Björn, Ma	From plant genomes to phenotypes		1681656	10.1016/j.jbiotec.	https://linkinghub.elsevier.com/retrieve/pii/S0168165617302845		2017-11	2021-10-12 2:55	2021-10-12 2:55	46-52		261	en
P3A9D3LI	journalArticle	2017	Morey, Frances M	De novo transcriptome assembly and RNA-Seq expr		18747787	10.1016/j.marger	https://linkinghub.elsevier.com/retrieve/pii/S1874778717301666		2017-10	2021-10-12 2:55	2021-10-12 2:55	77-92		35	en
XJ4RICHG	journalArticle	2017	Bucksch, Joshua	Overcoming the Law of the Hidden in Cyberinfrastru		13601385	10.1016/j.tplants.	https://linkinghub.elsevier.com/retrieve/pii/S1360138516301996		2017-02	2021-10-12 2:55	2021-10-12 2:55	117-123	2	22	en
LTZUSKNU	journalArticle	2017	Lobet, Guillaume	Image Analysis in Plant Sciences: Publish Then Peri		13601385	10.1016/j.tplants.	https://linkinghub.elsevier.com/retrieve/pii/S1360138517300912		2017-07	2021-10-12 2:55	2021-10-12 2:55	559-566	7	22	en
VPD2RDML	journalArticle	2017	Cutcher-Gershen	Five ways consortia can catalyse open science		0028-0836, 1476	10.1038/543615a	http://www.nature.com/articles/543615a		2017-03	2021-10-12 2:55	2021-10-12 2:55	615-617	7647	543	en
RZG45CPW	journalArticle	2017	Luo, Jan, Ming-C	Genome sequence of the progenitor of the wheat D		0028-0836, 1476	10.1038/nature24	http://www.nature.com/articles/nature24486		2017-11	2021-10-12 2:55	2021-10-12 2:55	498-502	7681	551	en
ULQ3MLD3	journalArticle	2017	Wang, Xiang H.-I	Bone-in-culture array as a platform to model early-st		2041-1723	10.1038/ncomms	http://www.nature.com/articles/ncomms15045		2017-04	2021-10-12 2:55	2021-10-12 2:55	15045	1	8	en
VPY9MHSF	journalArticle	2017	Paez-Espino, Nik	Nontargeted virus sequence discovery pipeline and		1754-2189, 1750	10.1038/nprot.20	http://www.nature.com/articles/nprot.2017.063		2017-08	2021-10-12 2:55	2021-10-12 2:55	1673-1682	8	12	en
6LT9SBFG	journalArticle	2017	Roux, Matthew B	Ecogenomics of virophages and their giant virus hos		2041-1723	10.1038/s41467-	http://www.nature.com/articles/s41467-017-01086-2		2017-12	2021-10-12 2:55	2021-10-12 2:55	858	1	8	en
UDDESPYJ	journalArticle	2017	Lopes dos Santo	Chloropicophyceae, a new class of picophytoplankto		2045-2322	10.1038/s41598-	http://www.nature.com/articles/s41598-017-12412-5		2017-12	2021-10-12 2:55	2021-10-12 2:55	14019	1	7	en
5LULU68M	journalArticle	2017	Xu, Ian T., Shuqi	Wild tobacco genomes reveal the evolution of nicoti		0027-8424, 1091	10.1073/pnas.17	http://www.pnas.	Nicotine, the signature alkaloid of Nicotiana species responsible for the addictive properties of human tobacco smo	2017-06-06	2021-10-12 2:55	2021-12-07 20:07	6133-6138	23	114	en
HSTPZSM4	journalArticle	2017	Monier, Thomas A	Host-derived viral transporter protein for nitrogen up		0027-8424, 1091	10.1073/pnas.17	http://www.pnas.org/lookup/doi/10.1073/pnas.1708097114		2017-09-05	2021-10-12 2:55	2021-12-07 20:09	E7489-E7498	36	114	en
3HYGAKEH	journalArticle	2017	Farman, Jorge, M	The Lolium Pathotype of <i>Magnaporthe oryzae</i		0191-2917	10.1094/PDIS-05	http://apsjournals.apsnet.org/doi/10.1094/PDIS-05-16-0700-RE		2017-05	2021-10-12 2:55	2021-10-12 2:55	684-692	5	101	en
F2EA2495	journalArticle	2017	DeChant, Hod, C	Automated Identification of Northern Leaf Blight-Infe		0031-949X	10.1094/PHYTO-	https://apsjournals.apsnet.org/doi/10.1094/PHYTO-11-16-0417-R		2017-11	2021-10-12 2:55	2021-10-12 2:55	1426-1432	11	107	en
JCKNYXZA	journalArticle	2017	Zajitschek, Mollie	High-protein paternal diet confers an advantage to s		1744-9561, 1744	10.1098/rsbl.201	https://royalsocietypublishing.org/doi/10.1098/rsbl.2016.0914		2017-02-28	2021-10-12 2:55	2021-12-07 20:05	20160914	2	13	en
H2FKJWDH	report	2017	Robbins, José R.	Growth is required for perception of water availability to pattern plant root branches				http://biorxiv.org/	Water availability is a potent regulator of development in plants and acts as a positional cue to induce root branchir	2017-01-02	2021-10-12 2:55	2021-12-07 20:04				en
TGY9NS6C	report	2017	Nelson, Mark A.,	Evolinc: a comparative transcriptomics and genomics pipeline for quickly identifying sec				http://biorxiv.org/	Abstract Long intergenic non-coding RNAs (lincRNAs) are an abundant and functionally diverse class of eukaryotic	2017-02-20	2021-10-12 2:55	2021-12-07 20:05				en
QNNWA7DI	report	2017	Riemer, Ethan, K	No general relationship between mass and temperature in endothermic species				http://biorxiv.org/	Bergmann's rule is a widely-accepted biogeographic rule stating that individuals within a species are smaller in war	2017-03-08	2021-10-12 2:55	2021-12-07 20:05				en
KX8IFG6F	journalArticle	2017	Friesner, Siobhan	The Next Generation of Training for Arabidopsis Res		0032-0889, 1532	10.1104/pp.17.01	http://www.plantphysiol.org/lookup/doi/10.1104/pp.17.01490		2017-12	2021-10-12 2:55	2021-10-12 2:55	1499-1509	4	175	en
FWXBSBTE	journalArticle	2017	Blažun Vošner, P	Openness and information technology: a bibliometric		0368-492X	10.1108/K-10-20	https://www.emerald.com/insight/content/doi/10.1108/K-10-2016-0292/full/html		2017-05-02	2021-10-12 2:55	2021-12-07 20:00	750-766	5	46	en
CM3ZQEE2	conferencePaper	2017	Knepper, Sudhak	Using the Jetstream Research Clo	978-1-5090-6611-7		10.1109/CCGRID	http://ieeexplore.ieee.org/document/7973774/		2017-05	2021-10-12 2:55	2021-10-12 2:55	753-757			
847V242Y	conferencePaper	2017	Krishnamurthy, S	xDCI, a data science cyberinfrastru	978-1-5386-3472-1		10.1109/HPEC.2	http://ieeexplore.ieee.org/document/8091022/		2017-09	2021-10-12 2:55	2021-10-12 2:55	7-Jan			
JEZ5QHND	conferencePaper	2017	Roy, Kawser, Bar	Towards a Reference Architecture	978-1-5090-5729-0		10.1109/ICSA.20	http://ieeexplore.ieee.org/document/7930197/		2017-04	2021-10-12 2:55	2021-10-12 2:55	41-50			
MSQBNMUM	journalArticle	2017	Carvajal-Endara,	Habitat filtering not dispersal limitation shapes ocear		1461023X	10.1111/ele.1275	http://doi.wiley.com/10.1111/ele.12753		2017-04	2021-10-12 2:55	2021-10-12 2:55	495-504	4	20	en
XSVVKPM8	journalArticle	2017	Satjarak, Linda E	Comparative DNA sequence analyses of <i>Pyramir		223646	10.1111/jpy.1251	http://doi.wiley.com/10.1111/jpy.12515		2017-04	2021-10-12 2:55	2021-10-12 2:55	415-424	2	53	en
BIRRY7KE	journalArticle	2017	Satjarak, Linda E	Complete mitochondrial genomes of prasinophyte al		223646	10.1111/jpy.1252	http://doi.wiley.com/10.1111/jpy.12521		2017-06	2021-10-12 2:55	2021-10-12 2:55	601-615	3	53	en
XQHWM6JV	journalArticle	2017	Becker Scarpitta,	Long-term community change: bryophytes are more		11009233	10.1111/jvs.1257	http://doi.wiley.com/10.1111/jvs.12579		2017-11	2021-10-12 2:55	2021-10-12 2:55	1220-1229	6	28	en
46M2IAPD	journalArticle	2017	Minervini, Sotiris	Phenotiki: an open software and hardware platform f		9607412	10.1111/tpj.1347	http://doi.wiley.com/10.1111/tpj.13472		2017-04	2021-10-12 2:55	2021-10-12 2:55	204-216	1	90	en
JCDNUJ3U	journalArticle	2017	Rai, Mami, Amit;	Integrated omics analysis of specialized metabolism		9607412	10.1111/tpj.1348	http://doi.wiley.com/10.1111/tpj.13485		2017-05	2021-10-12 2:55	2021-10-12 2:55	764-787	4	90	en
NGW3Z45K	journalArticle	2017	Deist, Susan J., I	Novel Mechanisms Revealed in the Trachea Transcr		1556-6811, 1556	10.1128/CVI.000	http://cvi.asm.org	ABSTRACT Newcastle disease virus (NDV) has a devastating impact on poultry production in developing countries	2017-05	2021-10-12 2:55	2021-10-12 2:55	e00027-17, /cdli/24/5/e00027-17.a	5	24	en
I4UY5Q9I	journalArticle	2017	Nigro, Grieg F., C	Viruses in the Oceanic Basement		2150-7511	10.1128/mBio.02	http://mbio.asm.o	ABSTRACT Microbial life has been detected well into the igneous crust of the seafloor (i.e., the oceanic basement)	2017-05-03	2021-10-12 2:55	2021-12-07 20:00	e02129-16, /mbio/8/2/e02129-16.a	2	8	en
XVBDGZ7S	conferencePaper	2017	Coronel, Stepher	DesignSafe: Using Elasticsearch t	978-1-4503-5272-7		10.1145/3093338	http://dl.acm.org/citation.cfm?doid=3093338.3093386		2017	2021-10-12 2:55	2021-10-12 2:55	3-Jan			en
7KUSLXDR	conferencePaper	2017	Willis, Christine,	Container-based Analysis Environ	978-1-4503-5272-7		10.1145/3093338	http://dl.acm.org/citation.cfm?doid=3093338.3104164		2017	2021-10-12 2:55	2021-10-12 2:55	4-Jan			en
NBHABY8I	conferencePaper	2017	Fischer, Craig A.,	Jetstream: A Cloud System Enabli	978-1-4503-4919-2		10.1145/3123458	http://dl.acm.org/citation.cfm?doid=3123458.3123466		2017	2021-10-12 2:55	2021-10-12 2:55	67-72			en
NRBGBUB7	conferencePaper	2017	Hancock, David	Jetstream: Early Operations Perfo	978-1-4503-5149-2		10.1145/3147213	http://dl.acm.org/citation.cfm?doid=3147213.3155104		2017	2021-10-12 2:55	2021-10-12 2:55	7-Jul			en
NXKZSB2H	journalArticle	2017	Mozzherin, Davic	"gnparser": a powerful parser for scientific names ba		1471-2105	10.1186/s12859-	http://bmcbioinformatics.biomedcentral.com/articles/10.1186/s12859-017-1663-3		2017-12	2021-10-12 2:55	2021-10-12 2:55	279	1	18	en
BSNLNJG9	journalArticle	2017	Molin, Christophe	The unique genomic landscape surrounding the EPS		1471-2164	10.1186/s12864-	http://bmcgenomics.biomedcentral.com/articles/10.1186/s12864-016-3336-4		2017-12	2021-10-12 2:55	2021-10-12 2:55	91	1	18	en
4BWNQJKW	journalArticle	2017	Chen, Jisen, Yihc	Evolution and expression of the fructokinase gene fa		1471-2164	10.1186/s12864-	http://bmcgenomics.biomedcentral.com/articles/10.1186/s12864-017-3535-7		2017-12	2021-10-12 2:55	2021-10-12 2:55	197	1	18	en
W9Y5T2LW	journalArticle	2017	Zhang, Susan J,	Transcriptome analysis reveals potential mechanism		1471-2164	10.1186/s12864-	http://bmcgenomics.biomedcentral.com/articles/10.1186/s12864-017-3675-9		2017-12	2021-10-12 2:55	2021-10-12 2:55	295	1	18	en
KJW3SGRL	journalArticle	2017	Levy, Elizabeth A	Global gene expression in two potato cultivars in res		1471-2164	10.1186/s12864-	https://bmcgenomics.biomedcentral.com/articles/10.1186/s12864-017-4313-2		2017-12	2021-10-12 2:55	2021-10-12 2:55	960	1	18	en
UFHAY26T	journalArticle	2017	Fonseca, Lucia C	Differences in global gene expression in muscle tiss		1471-2164	10.1186/s12864-	https://bmcgenomics.biomedcentral.com/articles/10.1186/s12864-017-4323-0		2017-12	2021-10-12 2:55	2021-10-12 2:55	945	1	18	en
YJVDT258	journalArticle	2017	Deist, Susan J., I	Resistant and susceptible chicken lines show distinc		1471-2164	10.1186/s12864-	https://bmcgenomics.biomedcentral.com/articles/10.1186/s12864-017-4380-4		2017-12	2021-10-12 2:55	2021-10-12 2:55	989	1	18	en
7HKWG7YP	journalArticle	2017	Kang, Rosa, Dae	Microbiota Transfer Therapy alters gut ecosystem ar		2049-2618	10.1186/s40168-	http://microbiomejournal.biomedcentral.com/articles/10.1186/s40168-016-0225-7		2017-12	2021-10-12 2:55	2021-10-12 2:55	10	1	5	en
TRJW8BBE	journalArticle	2017	Hyman, Sibiry, G	Improving agricultural knowledge management: The		2046-1402	10.12688/f1000re	https://f1000rese	Background: Opportunities to use data and information to address challenges in international agricultural research	2017-03-24	2021-10-12 2:55	2021-12-07 20:05	317		6	en
7IX4DLBA	journalArticle	2017	Eckes, Wiktor, Ar	Introducing the Brassica Information Portal: Towards		2046-1402	10.12688/f1000re	https://f1000rese	The Brassica Information Portal (BIP) is a centralised repository for Brassica phenotypic data. Trait data associatec	2017-04-12	2021-10-12 2:55	2022-03-03 23:2	465		6	en
IV6PGRAT	journalArticle	2017	Mondal, Nagendr	First de novo draft genome sequence of Oryza coar		2046-1402	10.12688/f1000re	https://f1000rese	Oryza coarctata plants, collected from Sundarban delta of West Bengal, India, have been used in the present study	2017-09-25	2021-10-12 2:55	2021-12-07 20:09	1750		6	en
EFWF7C2Q	journalArticle	2017	Manching, Ann E	Maize Phyllosphere Microbial Community Niche Dev		2046-1402	10.12688/f1000re	https://f1000rese	Background : The phyllosphere hosts a variety of microorganisms, including bacteria, which can play a positive role	2017-12-13	2021-10-12 2:55	2021-12-07 20:10	1698		6	en
WXFIIB3G	conferencePaper	2017	Jiang, Rui, Yu; Li	<i>GPhenoVision: A Ground Mobile System with Multi-modal Ima			10.13031/aim.20	http://elibrary.asabe.org/abstract.asp?JID=5&AID=47955&CID=spo2017&T=1		2017	2021-10-12 2:55	2021-10-12 2:55				en
F5FGPP7Z	journalArticle	2017	Gorgolewski, Rus	BIDS apps: Improving ease of use, accessibility, anc		1553-7358	10.1371/journal.p	https://dx.plos.org/10.1371/journal.pcbi.1005209		2017-03-09	2021-10-12 2:55	2021-12-07 20:05	e1005209	3	13	en
ACD6KY3Z	journalArticle	2017	Barone, David, L	Unmet needs for analyzing biological big data: A sur		1553-7358	10.1371/journal.p	http://dx.plos.org/10.1371/journal.pcbi.1005755		2017-10-19	2021-10-12 2:55	2021-12-07 20:10	e1005755	10	13	en
5VQ8232J	journalArticle	2017	Trail, Jeffrey P., F	The ancestral levels of transcription and the evolutio		1553-7404	10.1371/journal.p	https://dx.plos.org/10.1371/journal.pgen.1006867		2017-07-13	2021-10-12 2:55	2021-12-07 20:08	e1006867	7	13	en
QTMCTU9U	journalArticle	2017	Yang, Jeffrey, Jin	Incomplete dominance of deleterious alleles contribu		1553-7404	10.1371/journal.p	https://dx.plos.org/10.1371/journal.pgen.1007019		2017-09-27	2021-10-12 2:55	2021-12-07 20:09	e1007019	9	13	en
T97EZM2T	journalArticle	2017	Zou, Zhe-Xue, Bi	MIPE: A metagenome-based community structure e:		1932-6203	10.1371/journal.p	https://dx.plos.org/10.1371/journal.pone.0174609		2017-03-28	2021-10-12 2:55	2021-12-07 20:05	e0174609	3	12	en
6J4MXAQF	journalArticle	2017	Shi, Zhongguo, S	Variations of five eIF4E genes across cassava acces		1932-6203	10.1371/journal.p	https://dx.plos.org/10.1371/journal.pone.0181998		2017-08-03	2021-10-12 2:55	2021-12-07 20:08	e0181998	8	12	en
SZFCN22J	journalArticle	2017	Iamartino, John L	Design and validation of a 90K SNP genotyping ass		1932-6203	10.1371/journal.p	https://dx.plos.org/10.1371/journal.pone.0185220		2017-10-05	2021-10-12 2:55	2021-12-07 20:10	e0185220	10	12	en
UUI7KCC9	journalArticle	2017	List, Markus	Using Docker Compose for the Simple Deployment		1613-4516	10.1515/jib-2017	http://www.degru	Abstract Docker virtualization allows for software tools to be executed in an isolated and controlled environment ref	2017-06-27	2021-10-12 2:55	2021-12-07 20:08		2	14	
SZM45CHX	journalArticle	2017	Nelson, Mark A.,	Evolinc: A Tool for the Identification and Evolutionary		1664-8021	10.3389/fgene.20	http://journal.frontiersin.org/article/10.3389/fgene.2017.00052/full		2017-05-09	2021-10-12 2:55	2021-12-07 20:07	52		8	
9E4LGYZY	journalArticle	2017	Liu, Guangwu, H	Quantitative Trait Locus Analysis for Deep-Sowing G		1664-462X	10.3389/fpls.201	http://journal.frontiersin.org/article/10.3389/fpls.2017.00813/full		2017-05-22	2021-10-12 2:55	2021-12-07 20:07	813		8	
WZJVH53H	journalArticle	2017	Zhang, Dan, Hen	Transcriptome Dataset of Soybean (Glycine max) G		2306-5729	10.3390/data202	http://www.mdpi.com/2306-5729/2/2/17		2017-05-16	2021-10-12 2:55	2021-12-07 20:07	17	2	2	en
MP5K9S8Y	journalArticle	2017	Grealy, Michael,	Time to Spread Your Wings: A Review of the Avian A		2073-4425	10.3390/genes8	http://www.mdpi.com/2073-4425/8/7/184		2017-07-18	2021-10-12 2:55	2021-12-07 20:08	184	7	8	en
LBPN8SCH	journalArticle	2017	Gillan, Michael, J	Fine-Resolution Repeat Topographic Surveying of D		2072-4292	10.3390/rs90504	http://www.mdpi.com/2072-4292/9/5/437		2017-05-03	2021-10-12 2:55	2021-12-07 20:00	437	5	9	en
FXNA9EZ3	journalArticle	2017	Dolan, Andrew L.	Floristic response to urbanization: Filtering of the bic		0002-9122, 1537	10.3732/ajb.170	http://doi.wiley.com/10.3732/ajb.1700136		2017-08	2021-10-12 2:55	2021-10-12 2:55	1179-1187	8	104	en
7U4FA5IQ	journalArticle	2017	Joyce, Eric, Blak	Leveraging CyVerse Resources for De Novo Compa		1940-087X	10.3791/55009	https://www.jove.com/video/55009/leveraging-cyverse-resources-for-de-novo-comparative-transcriptomics		2017-05-09	2021-10-12 2:55	2021-12-07 20:00	55009	123		en
W2GDKKF4	journalArticle	2017	Deck, Robert, Jo	A High-throughput Data Ingest Pipeline for Semantic		2535-0897	10.3897/tdwgpro	https://tdwgproceedings.pensoft.net/articles.php?id=20208		2017-08-11	2021-10-12 2:55	2021-12-07 20:08	e20208		1	
CZRW26SW	journalArticle	2017	Walls, Pier Luigi,	Semantically Defining Populations for 'Omics Resea		2535-0897	10.3897/tdwgpro	https://tdwgproceedings.pensoft.net/articles.php?id=20435		2017-08-20	2021-10-12 2:55	2021-12-07 20:09	e20435		1	
7ZK676NT	journalArticle	2017	Stucky, Robert, E	An Update on the Plant Phenology Ontology and Pla		2535-0897	10.3897/tdwgpro	https://tdwgproceedings.pensoft.net/articles.php?id=20487		2017-08-21	2021-10-12 2:55	2021-12-07 20:09	e20487		1	
KL8GNNBR	journalArticle	2017	Adhikari, Ali M., L	Insights into Seasonal Dormancy of Perennial Herba		2158-2742, 2158	10.4236/ajps.201	http://www.scirp.org/journal/doi.aspx?DOI=10.4236/ajps.2017.811179		2017	2021-10-12 2:55	2021-10-12 2:55	2650-2680	11	8	
IHZQMQNW	report	2017	Chard, Jason, Ky	The Modern Research Data Portal: A design pattern for networked, data-intensive scien				https://peerj.com	We describe best practices for providing convenient, high-speed, secure access to large data via research data po	2017-09-12	2021-10-12 2:55	2021-12-07 20:09				en
5RJFMZIR	journalArticle	2017	Bolduc, Matthew	vConTACT: an iVirus tool to classify double-strandec		2167-8359	10.7717/peerj.32	https://peerj.com	Taxonomic classification of archaeal and bacterial viruses is challenging, yet also fundamental for developing a pre	2017-05-03	2021-10-12 2:55	2021-12-07 20:00	e3243		5	en
DLZESG5T	journalArticle	2017	Holdgraf, Dani, C	Portable learning environments for hands-on computational instruction: Using container				http://arxiv.org/ab	There is an increasing interest in learning outside of the traditional classroom setting. This is especially true for topi	2017-06-19	2021-10-12 2:55	2021-12-07 20:07				
AU9JNW9H	journalArticle	2017	Perrin, Bruno J.,	OMICtools: a community-driven search engine for biological data analysis				http://arxiv.org/ab	With high-throughput biotechnologies generating unprecedented quantities of data, researchers are faced with the	2017-07-12	2021-10-12 2:55	2021-12-07 20:08				
9GCKXG5D	book	2017	Gilman, Ian Sper	Systematics of Chloropyron (Orobanchaceae): Implications of missing data on quartet-based species tree methods						2017	2021-10-12 2:55	2021-10-12 2:55				
CFXFWMXS	conferencePaper	2017	Fonner, Rion, Jol	Containerizing Conda						2017	2021-10-12 2:55	2021-10-12 2:55				
3RR3BKVG	conferencePaper	2017	Wang, Hao, Chui	Formalizing Data Management Systems: A Case Study of Syndicate Protocol						2017	2021-10-12 2:55	2021-10-12 2:55	1031–1034			
UE6HBBGG	book	2017	Belete, Getachev	Integrating models on the web: applications for socio-environmental studies						2017	2021-10-12 2:55	2021-10-12 2:55				

DUCSITDQ	book	2017	Hwang, Min, Kai;	Big-data analytics for cloud, IoT and cognitive computing						2017	2021-10-12 2:55	2021-10-12 2:55						
9YPJ97B6	journalArticle	2017	Van Es, Joshua,	Innovation in agriculture and food systems in the digital age						2017	2021-10-12 2:55	2021-10-12 2:55		97–104				
RA4L78HY	journalArticle	2017	Cicero, John R, (	Biodiversity Informatics and Data Quality on a Global Scale1						2017	2021-10-12 2:55	2021-10-12 2:55						
SKUQKCM9	book	2017	Hwang, K	Cloud Computing for Machine Learning and Cognitive Applications: A Machine Learning Approach						2017	2021-10-12 2:55	2021-10-12 2:55						
67SZF9HU	book	2017	Taber, Lynne, Ke	Teaching Gifted Learners in STEM Subjects: Developing talent in science, technology, engineering and mathematics						2017	2021-10-12 2:55	2021-10-12 2:55						
3ZWLATGS	conferencePaper	2017	Zhao, I Luk; othe	GABBs-Reusable Geospatial Data Analysis Building Blocks for Science Gateways.						2017	2021-10-12 2:55	2021-10-12 2:55						
4XDB33PC	journalArticle	2017	Roux, Matthew B	Benchmarking viromics: an in silico evaluation of metagenome-enabled estimates of viral community composition and diversity						2017	2021-10-12 2:55	2021-10-12 2:55		e3817			5	
S5S84KEN	thesis	2017	Nasko, Daniel Jo	Uncovering the Biological Features of Unknown Viruses Using Shotgun Metagenomic Sequence Data						2017	2021-10-12 2:55	2021-10-12 2:55						
ZMCS864W	journalArticle	2017	Vaidya, Gaurav (	Taxonomic Checklists as Biodiversity Data: How Series of Checklists can Provide Information on Synonymy, Circumscription Change and Taxonomic Discovery						2017	2021-10-12 2:55	2021-10-12 2:55						
PYAJGVJ2	thesis	2017	Knepper, Richard	Shifting Modalities of Use in the XSEDE Project						2017	2021-10-12 2:55	2021-10-12 2:55						
V2VLCICD	journalArticle	2017	Enke, Melika, Ra	Genomics RNA-seq Analysis part 1-CyVerse DE, FastQC, Trimmomatic (updated 11/17)						2017-11	2021-10-12 2:55	2021-12-03 18:5						
JTM5XAFL	book	2017	Buonaccorsi, A, \	An Introduction to Eukaryotic Genome Analysis in Non-model Species for Undergraduates: A tutorial from the Genome Consortium for Active Teaching						2017	2021-10-12 2:55	2021-10-12 2:55						
PTEEYQP4	thesis	2017	Aloisio, Jason M	Rooftop plants: Community and nutrient dynamics of New York City green roofs						2017	2021-10-12 2:55	2021-10-12 2:55						
QA3BLQNU	conferencePaper	2017	Eckes, Wiktor, , A	The Brassica Information Portal: Towards Integrating Phenotype and Genotype Data				https://www.intlpag.org/2022/images/pdf/2017/PAGXXV-abstracts-computer_demos.pdf		2017	2021-10-12 2:55	2022-03-03 23:2					C11	
FVPQ4U8Y	conferencePaper	2017	Stanzione, Matth	The New Advanced Computing Landscape: Opportunities for Molecular Biology				http://www.smbe2017.org/wp-content/uploads/2016/02/SMBE-Abstract-Book-22-June.pdf		2017	2021-10-12 2:55	2021-10-12 2:55		55				
D3CFGM9C	report	2017	Es, Joshua, , Hai	Innovation in Agriculture and Food Systems in the Digital Age				http://www.wipo.int/edocs/pubdocs/en/wipo_pub_gii_2017-chapter4.pdf		2017	2021-10-12 2:55	2021-10-12 2:55		97-104				
9FNIMNFL	conferencePaper	2017	Espiritu, Kang Mi	Assessment of SO2 Pollution in Central Park by DNA Barcoding of lichen specimens				https://aaas.confex.com/aaas/2017/webprogram/Paper19818.html		2017	2021-10-12 2:55	2021-10-12 2:55						
EEICCDQP	book	2017	Foster, Dennis B.	Cloud Computing for Science and Engineering				https://books.google.com/books?id=FYc3DwAAQBAJ&printsec=frontcover&source=gbs_ge_summary_r&cad=0#v=onepage&q&f=fa		2017	2021-10-12 2:55	2021-10-12 2:55						
RUPN27QV	report	2017	Fedorov, Kristian	Scalable Image Informatics				https://escholarship.org/uc/item/7nf7492x		2017	2021-10-12 2:55	2021-10-12 2:55						
XNH8TLJK	journalArticle	2016	Xu, Weijia; Huan	Supporting Efficie	SciDataCon			https://www.researchgate.net/profile/Ruizhu-Huang/publication/307856303_Supporting_Efficient_Content_Based_Collection_Compa		2016-09-06	2021-09-07 21:5	2022-03-04 15:5						
NMJ4SCS4	journalArticle	2016	Boyle, Adrian A.,	Geographic comparison of plant genera used in frug		2752565	10.1002/ajp.2242	http://doi.wiley.com/10.1002/ajp.22422		2016-05	2021-10-12 2:39	2021-10-12 2:39		493-506		5	78	en
9QKHZ9ES	journalArticle	2016	Berton, Fernando	Gene expression profile of intramuscular muscle in I		1471-2164	10.1186/s12864-	http://bmcgenomics.biomedcentral.com/articles/10.1186/s12864-016-3232-y		2016-12	2021-10-12 2:39	2021-10-12 2:39		972		1	17	en
R5YVL5HQ	journalArticle	2016	Brum, Matthew B	Seasonal time bombs: dominant temperate viruses a		1751-7362, 1751	10.1038/ismej.20	http://www.nature.com/articles/ismej2015125		2016-02	2021-10-12 2:39	2021-10-12 2:39		437-449		2	10	en
5PPA5RZM	conferencePaper	2016	Minervini, Sotirio	An interactive tool for semi-automated leaf annotation						2016	2021-10-12 2:39	2021-10-12 2:39						
W5MLD4XB	journalArticle	2016	Chard, Ian, Kyle;	Globus Nexus: A Platform-as-a-Service provider of r		0167739X	10.1016/j.future.2	https://linkinghub.elsevier.com/retrieve/pii/S0167739X1500285X		2016-03	2021-10-12 2:39	2021-10-12 2:39		571-583			56	en
63CYEZXJ	journalArticle	2016	Barker, Donald A	On the relative abundance of autopolyploids and allo		0028646X	10.1111/nph.1369	http://doi.wiley.com/10.1111/nph.13698		2016-04	2021-10-12 2:39	2021-10-12 2:39		391-398		2	210	en
YAX657GT	journalArticle	2016	Wang, Andrew H	Comparative genomic de-convolution of the cotton g		0028-646X, 1469	10.1111/nph.1368	https://onlinelibrary.wiley.com/doi/abs/10.1111/nph.13689		2016-02	2021-10-12 2:39	2021-10-12 2:39		1252-1263		3	209	en
GRJL9SXT	journalArticle	2016	Kelly, Toni, Derek	Extracting complex lesion phenotypes in Zea mays		0932-8092, 1432	10.1007/s00138-	http://link.springer.com/10.1007/s00138-015-0718-6		2016-01	2021-10-12 2:39	2021-10-12 2:39		145-156		1	27	en
JAJUE9ZB	journalArticle	2016	Dash, Steven B.,	Legume information system (LegumeInfo.org): a key		0305-1048, 1362	10.1093/nar/gkv1	https://academic.oup.com/nar/article-lookup/doi/10.1093/nar/gkv1159		2016-01-04	2021-10-12 2:39	2021-12-07 19:5		D1181-D1188		D1	44	en
LLT8HHMG	journalArticle	2016	Kelly, Toni, Derek	An opinion on imaging challenges in phenotyping fie		0932-8092, 1432	10.1007/s00138-	http://link.springer.com/10.1007/s00138-015-0728-4		2016-07	2021-10-12 2:39	2021-10-12 2:39		681-694		5	27	en
WM3CVY2Y	journalArticle	2016	Doughty, Mauro,	Megafauna extinction, tree species range reduction,		9067590	10.1111/ecog.018	http://doi.wiley.com/10.1111/ecog.01587		2016-02	2021-10-12 2:39	2021-10-12 2:39		194-203		2	39	en
ZWIURB89	bookSection	2016	Cooper, Pankaj, I	The Plant Ontology: A Tool for Plar	978-1-4939-3166-8 978-1-4939-3167-5			http://link.springer.com/10.1007/978-1-4939-3167-5_5		2016	2021-10-12 2:39	2021-10-12 2:39		89-114			1374	
VQN5ADTG	bookSection	2016	Jaiswal, Björn, Pa	Plant Pathway Databases	978-1-4939-3166-8 978-1-4939-3167-5			http://link.springer.com/10.1007/978-1-4939-3167-5_4		2016	2021-10-12 2:39	2021-10-12 2:39		71-87			1374	
8CEBFXXW	journalArticle	2016	Tello-Ruiz, Doree	Gramene 2016: comparative plant genomics and pa		0305-1048, 1362	10.1093/nar/gkv1	https://academic.oup.com/nar/article-lookup/doi/10.1093/nar/gkv1179		2016-01-04	2021-10-12 2:39	2021-12-07 19:5		D1133-D1140		D1	44	en
DT9JZS9M	journalArticle	2016	Minervini, Sotirio	Finely-grained annotated datasets for image-based		1678655	10.1016/j.patrec.	https://linkinghub.elsevier.com/retrieve/pii/S0167865515003645		2016-10	2021-10-12 2:39	2021-10-12 2:39		80-89			81	en
4KUZE8VT	bookSection	2016	Yin, Paul C., Xiny	Crop Systems Biology: Where Are	978-3-319-20561-8 978-3-319-20562-5			http://link.springer.com/10.1007/978-3-319-20562-5_10		2016	2021-10-12 2:39	2021-10-12 2:39		219-227				en
J2LEUX96	journalArticle	2016	Morueta-Holme, .	A network approach for inferring species association		9067590	10.1111/ecog.018	http://doi.wiley.com/10.1111/ecog.01892		2016-12	2021-10-12 2:39	2021-10-12 2:39		1139-1150		12	39	en
BR98I7SD	journalArticle	2016	Cahill, Jeffrey S.,	Genetic differentiation and reduced genetic diversity		9621083	10.1111/mec.134	http://doi.wiley.com/10.1111/mec.13497		2016-01	2021-10-12 2:39	2021-10-12 2:39		515-526		2	25	en
CRXQRQYY	journalArticle	2016	Das, Donald L., S	Transcriptome analysis of the molting gland (Y-organ		1744117X	10.1016/j.cbd.20	https://linkinghub.elsevier.com/retrieve/pii/S1744117X15000672		2016-03	2021-10-12 2:39	2021-10-12 2:39		26-40			17	en
I539WENY	journalArticle	2016	Engemann, Jens	Patterns and drivers of plant functional group domin		244074	10.1111/boj.1236	https://academic.oup.com/botlinnean/article-lookup/doi/10.1111/boj.12362		2016-02	2021-10-12 2:39	2021-10-12 2:39		141-160		2	180	en
GBB7RM2L	journalArticle	2016	Baldeck, G. P., C	Environmental drivers of tree community turnover in		9067590	10.1111/ecog.018	http://doi.wiley.com/10.1111/ecog.01575		2016-11	2021-10-12 2:39	2021-10-12 2:39		1089-1099		11	39	en
M3DKVLKE	journalArticle	2016	Scharr, Sotirios A	Leaf segmentation in plant phenotyping: a collation s		0932-8092, 1432	10.1007/s00138-	http://link.springer.com/10.1007/s00138-015-0737-3		2016-05	2021-10-12 2:39	2021-10-12 2:39		585-606		4	27	en
R5ANPGW3	journalArticle	2016	Ostria-Gallardo, I	Transcriptomic analysis suggests a key role for <i>S		0028646X	10.1111/nph.1377	http://doi.wiley.com/10.1111/nph.13776		2016-04	2021-10-12 2:39	2021-10-12 2:39		694-708		2	210	en
RIE2N8TV	journalArticle	2016	Wang, Dexiang, '	Bioinformatic landscapes for plant transcription facto		0032-0935, 1432	10.1007/s00425-	http://link.springer.com/10.1007/s00425-015-2453-7		2016-02	2021-10-12 2:39	2021-10-12 2:39		297-304		2	243	en
9QK78V4W	journalArticle	2016	Billah, Reagan W	Using a data grid to automate data preparation pipel		13648152	10.1016/j.envsof	https://linkinghub.elsevier.com/retrieve/pii/S1364815215301249		2016-04	2021-10-12 2:51	2021-10-12 2:51		31-39			78	en
Y87VWTG9	journalArticle	2016	Song, Ting, Miao	Spatiotemporal data representation and its effect on		983004	10.1016/j.cageo.	https://linkinghub.elsevier.com/retrieve/pii/S0098300415300820		2016-02	2021-10-12 2:51	2021-10-12 2:51		21-Nov			87	en
RIYQWXR3	journalArticle	2016	Papanicolaou, Al	The life cycle of a genome project: perspectives and		2046-1402	10.12688/f1000re	https://f1000rese	Many research programs on non-model species biology have been empowered by genomics. In turn, genomics is	2016-01-05	2021-10-12 2:51	2021-12-07 19:5		18			5	en
RS8DKBA6	journalArticle	2016	Tyler, Ana L., Luc	Population Structure in the Model Grass Is Highly Co		1940-3372	10.3835/plantger	https://dl.sciencesocieties.org/publications/tpg/abstracts/9/2/plantgenome2015.08.0074		2016	2021-10-12 2:51	2021-10-12 2:51		0		2	9	en
4LSCHBTZ	journalArticle	2016	Cruz-Garcia, Dav	Wild harvest: distribution and diversity of wild food p		15735214	10.1016/j.njas.20	https://linkinghub.elsevier.com/retrieve/pii/S1573521415300105		2016-09	2021-10-12 2:51	2021-10-12 2:51		11-Jan			78	en
NUPB49T4	journalArticle	2016	Merchant, Parker	The iPlant Collaborative: Cyberinfrastructure for Ena		1545-7885	10.1371/journal.p	http://dx.plos.org/10.1371/journal.pbio.1002342		2016-01-11	2021-10-12 2:51	2021-12-07 19:5		e1002342		1	14	en
E69SIPZF	journalArticle	2016	Brooks, Steven S	Working beyond the confines of academic discipline		13960466	10.5210/fm.v21i2	http://journals.uic.edu/ojs/index.php/fm/article/view/6103		2016-01-26	2021-10-12 2:51	2021-12-07 19:5				2	21	
SEBSLPRZ	journalArticle	2016	Koureas, Vincent	Unifying European Biodiversity Informatics (BioUnify		2367-7163	10.3897/rio.2.e77	http://rio.pensoft.net/articles.php?id=7787		2016-01-19	2021-10-12 2:51	2021-12-07 19:5		e7787			2	
M6AY9IKS	journalArticle	2016	Zhang, Jisen, Qir	Structure, phylogeny, allelic haplotypes and express		1471-2164	10.1186/s12864-	http://www.biomedcentral.com/1471-2164/17/88		2016-12	2021-10-12 2:51	2021-10-12 2:51		88		1	17	en
W3VFE5KW	journalArticle	2016	Schneider, Gernc	Inbreeding drives maize centromere evolution		0027-8424, 1091	10.1073/pnas.15	http://www.pnas.org/lookup/doi/10.1073/pnas.1522008113		2016-02-23	2021-10-12 2:51	2021-12-07 19:5		E987-E996		8	113	en
TKCC7KZ7	journalArticle	2016	Goldsmith, Brian	<i>Plant-O-Matic</i> : a dynamic and mobile guide t		2041210X	10.1111/2041-21	http://doi.wiley.com/10.1111/2041-210X.12548		2016-08	2021-10-12 2:51	2021-10-12 2:51		960-965		8	7	en
VKF5PNKC	journalArticle	2016	Ranjan, Neelima	eQTL Regulating Transcript Levels Associated with I		0032-0889, 1532	10.1104/pp.16.00	http://www.plantphysiol.org/lookup/doi/10.1104/pp.16.00289		2016-09	2021-10-12 2:51	2021-10-12 2:51		328-340		1	172	en
B7GTQNHS	journalArticle	2016	Bassi, Shahryar I	Fast-forward genetics by radiation hybrids to saturat		14677644	10.1111/pbi.1253	http://doi.wiley.com/10.1111/pbi.12532		2016-08	2021-10-12 2:51	2021-10-12 2:51		1716-1726		8	14	en
A7VRUWU7	report	2016	Martinez, Neelim	A Sister of PIN1 gene in tomato (Solanum lycopersicum) defines organ initiation pattern				http://biorxiv.org/l	The spatiotemporal localization of the plant hormone auxin acts as a positional cue during early leaf and flower org	2016-03-02	2021-10-12 2:51	2021-12-07 19:5						en
XGZREB7M	journalArticle	2016	McNutt, J. L., M.;	Liberating field science samples and data		0036-8075, 1095	10.1126/science.	http://www.sciencemag.org/cgi/doi/10.1126/science.aad7048		2016-03-04	2021-10-12 2:51	2021-12-07 19:5		1024-1026		6277	351	en
6Q28GM4U	journalArticle	2016	Freese, Ann E., I	Integrated genome browser: visual analytics platform		1367-4803, 1460	10.1093/bioinfor	https://academic.oup.com/bioinformatics/article-lookup/doi/10.1093/bioinformatics/btw069		2016-07-15	2021-10-12 2:51	2021-12-07 20:0		2089-2095		14	32	en
4J32VFVR	journalArticle	2016	Kim, Konstantino	Visual Omics Explorer (VOE): a cross-platform porta		1367-4803, 1460	10.1093/bioinfor	https://academic.oup.com/bioinformatics/article-lookup/doi/10.1093/bioinformatics/btw119		2016-07-01	2021-10-12 2:51	2021-12-07 19:5		2050-2052		13	32	en
B3GNBIGB	journalArticle	2016	Chakrabarti, Arth	De novo Transcriptome Assembly and Dynamic Spa		1940-3372	10.3835/plantger	https://dl.sciencesocieties.org/publications/tpg/abstracts/9/2/plantgenome2015.06.0048		2016	2021-10-12 2:51	2021-10-12 2:51		0		2	9	en
SLD3T39I	journalArticle	2016	Gill, David, Gunb	Effect of Drought on Herbivore-Induced Plant Gene		2223-7747	10.3390/plants50	http://www.mdpi.com/2223-7747/5/1/13		2016-03-11	2021-10-12 2:51	2021-12-07 19:5		13		1	5	en
5MXRWQPT	journalArticle	2016	McKain, Jim H., I	A phylogenomic assessment of ancient polyploidy ar		1759-6653	10.1093/gbe/evw	https://academic.oup.com/gbe/article-lookup/doi/10.1093/gbe/evw060		2016-03-17	2021-10-12 2:51	2021-12-07 19:5		evw060				en
SEB66PH4	journalArticle	2016	Nuelle Jr, William	Biogeography of a disjunct population of Hemileuca peigleri (Lepidoptera: Saturniidae) in the Coastal Bend of Texas						2016	2021-10-12 2:51	2021-10-12 2:51		543–549			4	
7MXKAXW7	journalArticle	2016	Leipzig, Jeremy	A review of bioinformatic pipeline frameworks		1467-5463, 1477	10.1093/bib/bbw	https://academic.oup.com/bib/article-lookup/doi/10.1093/bib/bbw020		2016-03-24	2021-10-12 2:51	2021-12-07 19:5		bbw020				en
SWFDPSE5	journalArticle	2016	Liu, Yongrui, Hon	Gene duplication confers enhanced expression of 27		0027-8424, 1091	10.1073/pnas.16	http://www.pnas.org/lookup/doi/10.1073/pnas.1601352113		2016-05-03	2021-10-12 2:51	2021-12-07 19:5		4964-4969		18	113	en
4Q2ZDR8D	journalArticle	2016	Weigelt, Holger, I	Late Quaternary climate change shapes island biodi		0028-0836, 1476	10.1038/nature17	http://www.nature.com/articles/nature17443		2016-04	2021-10-12 2:51	2021-10-12 2:51		99-102		7597	532	en
P4YSASMC	journalArticle	2016	López de Heredia	RNA-seq analysis in forest tree species: bioinformat		1614-2942, 1614	10.1007/s11295-	http://link.springer.com/10.1007/s11295-016-0995-x		2016-04	2021-10-12 2:51	2021-10-12 2:51		30		2	12	en
J3XI9DFM	journalArticle	2016	Grossman, Walt,	A Case for Data Commons: Towards Data Science as a Service				http://arxiv.org/ab	As the amount of scientific data continues to grow at ever faster rates, the research community is increasingly in ne	2016-04-09	2021-10-12 2:51	2021-12-07 19:5						
ZHCG2NUB	journalArticle	2016	Blank, Ramona L	MicrO: an ontology of phenotypic and metabolic cha		2041-1480	10.1186/s13326-	http://jbiomedsem.biomedcentral.com/articles/10.1186/s13326-016-0060-6		2016-12	2021-10-12 2:51	2021-10-12 2:51		18		1	7	en
FSQ9M7GJ	journalArticle	2016	Afgan, Jeremy, E	The Galaxy platform for accessible, reproducible and		0305-1048, 1362	10.1093/nar/gkw	https://academic.oup.com/nar/article-lookup/doi/10.1093/nar/gkw343		2016-07-08	2021-10-12 2:51	2021-12-07 20:0		W3-W10		W1	44	en
46VQYKFG	journalArticle	2016	Engel, Benjamin,	The Bee Microbiome: Impact on Bee Health and Mo		2150-7511	10.1128/mBio.02	http://mbio.asm.c	ABSTRACT As pollinators, bees are cornerstones for terrestrial ecosystem stability and key components in agricult	2016-05-04	2021-10-12 2:51	2021-12-07 19:5		e02164-15, /mbio/7/2/e02164-15.a		2	7	en
XGQTHC7G	bookSection	2016	Popescu, Sorina	Big Data in Plant Science: Resour	978-1-4939-3570-3 978-1-4939-3572-7			http://link.springer.com/10.1007/978-1-4939-3572-7_27		2016	2021-10-12 2:51	2021-10-12 2:51		533-547			1415	en
I6HBIQLJ	journalArticle	2016	Gardiner, Anthon	Mapping-by-sequencing in complex polyploid genom		9607412	10.1111/tpj.1320	http://doi.wiley.com/10.1111/tpj.13204		2016-08	2021-10-12 2:51	2021-10-12 2:51		403-419		4	87	en
SQ86MII7	journalArticle	2016	Knecht, Harkama	Image Harvest: an open-source platform for high-thr		0022-0957, 1460	10.1093/jxb/erw1	https://academic.oup.com/jxb/article-lookup/doi/10.1093/jxb/erw176		2016-05	2021-10-12 2:51	2021-10-12 2:51		3587-3599		11	67	en
BNURQBT8	journalArticle	2016	Moody, Qin, Greg	Facilitating the Transformational: An Exploration of C		1047-7047, 1526	10.1287/isre.201	http://pubsonline.informs.org/doi/10.1287/isre.2016.0619		2016-06	2021-10-12 2:51	2021-10-12 2:51		324-346		2	27	en
AJEC464L	journalArticle	2016	Wang, Chuanzhu	DNA methylation changes facilitated evolution of ger		1474-760X	10.1186/s13059-	http://genomebiology.biomedcentral.com/articles/10.1186/s13059-016-0954-8		2016-12	2021-10-12 2:51	2021-10-12 2:51		92		1	17	en
WIDQ96F3	journalArticle	2016	Augustine, Richa	Defining the SUMO System in Maize: SUMOylation		0032-0889, 1532	10.1104/pp.16.00	http://www.plantphysiol.org/lookup/doi/10.1104/pp.16.00353		2016-07	2021-10-12 2:51	2021-10-12 2:51		2191-2210		3	171	en
3GD77PPN	journalArticle	2016	Kittelmann, L., M	Serial block face scanning electron microscopy and		222720	10.1111/jmi.1242	http://doi.wiley.com/10.1111/jmi.12424		2016-08	2021-10-12 2:51	2021-10-12 2:51		200-211		2	263	en
7DTKQ76Y	journalArticle	2016	Kyrpides, Natalia	Microbiome Data Science: Understanding Our Micro		0966842X	10.1016/j.tim.201	https://linkinghub.elsevier.com/retrieve/pii/S0966842X16000482		2016-06	2021-10-12 2:51	2021-10-12 2:51		425-427		6	24	en
LZZ78CG4	journalArticle	2016	An, Cynthia, Nan	Plant high-throughput phenotyping using photogram		1681699	10.1016/j.compa	https://linkinghub.elsevier.com/retrieve/pii/S0168169916301168		2016-09	2021-10-12 2:51	2021-10-12 2:51		376-394			127	en
SCEFMR6M	journalArticle	2016	Bombarely, Cris,	Insight into the evolution of the Solanaceae from the		2055-0278	10.1038/nplants.	http://www.nature.com/articles/nplants201674		2016-06	2021-10-12 2:51	2021-10-12 2:51		16074		6	2	en
RBZT9F6S	journalArticle	2016	Macrander, Mary	Tissue-Specific Venom Composition and Differential		1759-6653	10.1093/gbe/evw	https://academic.oup.com/gbe/article-lookup/doi/10.1093/gbe/evw155		2016-08	2021-10-12 2:51	2021-10-12 2:51		2358-2375		8	8	en
V4RJF5LT	conferencePaper	2016	Foster, Steven, Ia	The Discovery Cloud: Accelerating	978-1-5090-1961-8		10.1109/IC2E.20	http://ieeexplore.ieee.org/document/7484165/		2016-04	2021-10-12 2:51	2021-10-12 2:51		68-77				
TCSLYHC7	bookSection	2016	Heyduk, Travis C	Targeted DNA Region Re-sequenc	978-3-319-31348-1 978-3-319-31350-4			http://link.springer.com/10.1007/978-3-319-31350-4_3		2016	2021-10-12 2:51	2021-10-12 2:51		43-68				en
XMQWZAXV	journalArticle	2016	Larkin, Andrea T.	Keeping All the PIECES: Phylogenetically Informed I		1932-6203	10.1371/journal.p	https://dx.plos.org/10.1371/journal.pone.0156973		2016-06-03	2021-10-12 2:51	2021-12-07 19:5		e0156973		6	11	en
6GC8EYL8	journalArticle	2016	Cutcher-Gershen	Build It, But Will They Come? A Geoscience Cyberin		1683-1470	10.5334/dsj-2016	http://datascience.codata.org/article/10.5334/dsj-2016-008/		2016-07-11	2021-10-12 2:51	2021-12-07 20:0		8		0	15	
6NZYWDTW	journalArticle	2016	King abdulaziz U	Empirical Analysis of HPC Using Different Programm		20750161, 2075(	10.5815/ijmecs.2	http://www.mecs-press.org/ijmecs/ijmecs-v8-n6/v8n6-4.html		2016-06-08	2021-10-12 2:51	2021-12-07 19:5		27-34		6	8	
5L7TJPCJ	journalArticle	2016	Pamidighantam,	Community Science Exemplars in SEAGrid Science		18770509	10.1016/j.procs.2	https://linkinghub.elsevier.com/retrieve/pii/S1877050916310262		2016	2021-10-12 2:51	2021-10-12 2:51		1927-1939			80	en
55GBS7PN	journalArticle	2016	Henzen, L, Chris	Representing research collaborations and linking scientific project results in spatial data infrastructures by provenance information						2016	2021-10-12 2:51	2021-10-12 2:51						
B53JGPQW	journalArticle	2016	Maya-Lastra, Ca	ColectoR, a Digital Field Notebook for Voucher Spec		2168-0450	10.3732/apps.16	http://doi.wiley.com/10.3732/apps.1600035		2016-07	2021-10-12 2:51	2021-10-12 2:51		1600035		7	4	en
FLG8BFFX	journalArticle	2016	Wiser, Susan K.	Achievements and challenges in the integration, reu		11009233	10.1111/jvs.1241	http://doi.wiley.com/10.1111/jvs.12419		2016-09	2021-10-12 2:51	2021-10-12 2:51		868-879		5	27	en
J9DEMQZF	journalArticle	2016	Satjarak, Eunsoo	Complete Chloroplast Genome Sequence of Phagor		2169-8287	10.1128/genome	http://genomea.a	We report here the complete chloroplast genome sequence of Cymbomonas tetramitiformis strain PLY262, which i	2016-06-30	2021-10-12 2:51	2021-12-07 19:5		e00551-16, /ga/4/3/e00551-16.ato		3	4	en
SNLELHI2	journalArticle	2016	Satagopam, Reir	Integration and Visualization of Translational Medicir		2167-6461, 2167	10.1089/big.2015	http://www.liebertpub.com/doi/10.1089/big.2015.0057		2016-06	2021-10-12 2:51	2021-10-12 2:51		97-108		2	4	en
T5L7T562	journalArticle	2016	Devisetty, Eric, U	Bringing your tools to CyVerse Discovery Environme		2046-1402	10.12688/f1000re	https://f1000rese	Docker has become a very popular container-based virtualization platform for software distribution that has revoluti	2016-06-21	2021-10-12 2:51	2021-12-07 19:5		1442			5	en
XN9V869Y	journalArticle	2016	Cuevas, Andrew	The Evolution of Photoperiod-Insensitive Flowering i		0737-4038, 1537	10.1093/molbev/	https://academic.oup.com/mbe/article-lookup/doi/10.1093/molbev/msw120		2016-09	2021-10-12 2:51	2021-10-12 2:51		2417-2428		9	33	en
39X3IWUJ	bookSection	2016	Foster, Ian	Reasoning About Discovery Cloud	978-3-319-39085-7 978-3-319-39086-4			http://link.springer.com/10.1007/978-3-319-39086-4_1		2016	2021-10-12 2:51	2021-10-12 2:51		10-Mar			9698	
HKCVN66M	journalArticle	2016	Trujillo, Rebecca	The Argonaute-binding platform of NRPE1 evolves t		0028646X	10.1111/nph.140	http://doi.wiley.com/10.1111/nph.14089		2016-12	2021-10-12 2:51	2021-10-12 2:51		1094-1105		4	212	en
8W66RUVY	journalArticle	2016	Davey, Peter, Pe	The emergence of molecular profiling and omics tec		1438-793X, 1438	10.1007/s10142-	http://link.springer.com/10.1007/s10142-016-0501-4		2016-09	2021-10-12 2:51	2021-10-12 2:51		465-480		5	16	en
SA2IPVFH	journalArticle	2016	Gunadi, John J.,	Characterization of 40 soybean (Glycine max) prom		0167-6857, 1573	10.1007/s11240-	http://link.springer.com/10.1007/s11240-016-1038-x		2016-10	2021-10-12 2:51	2021-10-12 2:51		145-160		1	127	en
SSLQL8TV	journalArticle	2016	Center for Molec	Genome-wide transcriptome profiling of Gossypium		18360661, 1836:	10.21475/poj.16.	http://www.pomics.com/tilahun_9_3_2016_198_204.pdf		2016-06-20	2021-10-12 2:51	2021-12-07 19:5		198-204		3	9	
IM8F4GXL	journalArticle	2016	Narra, Marc J., H	A <i>Coccidioides posadasii CPS1</i> Deletion Mut		0019-9567, 1098	10.1128/IAI.0063	http://iai.asm.org	The CPS1 gene was identified as a virulence factor in the maize pathogen Cochliobolus heterostrophus . Hypothes	2016-10	2021-10-12 2:51	2021-10-12 2:51		3007-3016		10	84	en
QALFHAIS	journalArticle	2016	Torre, Federico, S	De Novo Assembly and Comparative Transcriptome		1932-6203	10.1371/journal.p	https://dx.plos.org/10.1371/journal.pone.0160370		2016-08-02	2021-10-12 2:51	2021-12-07 20:0		e0160370		8	11	en
XLRSUZMB	conferencePaper	2016	Haug-Baltzell, La	High-contrast imaging in the cloud with klipReduce and Findr			10.1117/12.2234	http://proceedings.spiedigitallibrary.org/proceeding.aspx?doi=10.1117/12.2234095		2016-08-08	2021-10-12 2:51	2021-12-07 20:0		99130F				
CLBD7MI5	conferencePaper	2016	Chard, Ian, Kyle;	Globus: Recent Enhancements an	978-1-4503-4755-6		10.1145/2949550	http://dl.acm.org/citation.cfm?doid=2949550.2949554		2016	2021-10-12 2:51	2021-10-12 2:51		8-Jan				en

P4TWKSM3	journalArticle	2016	Mushegian, Sant	Changes in the composition of the RNA virome mark			1741-7007	10.1186/s12915-	http://bmcbiol.biomedcentral.com/articles/10.1186/s12915-016-0288-8		2016-12	2021-10-12 2:51	2021-10-12 2:51		68	1	14		en
AH99N5ND	journalArticle	2016	Walsh, Jesse R	Computational methods for integrated analysis of omics and pathway data							2016	2021-10-12 2:51	2021-10-12 2:51						
LMLHGKMC	journalArticle	2016	Phillips, Adriana,	Live aboveground carbon stocks in natural forests of			3781127	10.1016/j.foreco.	https://linkinghub.elsevier.com/retrieve/pii/S0378112716302365		2016-08	2021-10-12 2:51	2021-10-12 2:51		119-128		374		en
4QJI8LPQ	journalArticle	2016	Ariani, Luca, And	Comparative epigenomic and transcriptomic analysis			988472	10.1016/j.envexp	https://linkinghub.elsevier.com/retrieve/pii/S0098847216301630		2016-12	2021-10-12 2:51	2021-10-12 2:51		16-27		132		en
SFZI9EG9	conferencePaper	2016	Hintsch, Klaus, J	A Review of the Literature on Configuration Management Tools.							2016	2021-10-12 2:51	2021-10-12 2:51		71				
8X943R9V	journalArticle	2016	Martinez, Neelim	A sister of PIN1 gene in tomato (Solanum lycopersic			121606	10.1016/j.ydbio.2	https://linkinghub.elsevier.com/retrieve/pii/S0012160616303062		2016-11	2021-10-12 2:51	2021-10-12 2:51		85-98	1	419		en
YMYZIJFQ	journalArticle	2016	Michaletz, Brian .	The energetic and carbon economic origins of leaf th			2055-0278	10.1038/nplants.	http://www.nature.com/articles/nplants2016129		2016-09	2021-10-12 2:51	2021-10-12 2:51		16129	9	2		en
5V6XSWL9	journalArticle	2016	Teytelman, Bonn	Protocols.io: Virtual Communities for Protocol Devel			1545-7885	10.1371/journal.p	https://dx.plos.org/10.1371/journal.pbio.1002538		2016-08-22	2021-10-12 2:51	2021-12-07 20:0		e1002538	8	14		en
BUFXR5RQ	bookSection	2016	Malik, Adeel	Genomics Resources for Plants		978-3-319-31701-4 978-3-319-31703-8			http://link.springer.com/10.1007/978-3-319-31703-8_2		2016	2021-10-12 2:51	2021-10-12 2:51		29-57				en
GDBMQHWJ	report	2016	Herndon, Jill L, N	CartograTree: Enabling landscape genomics for forest trees					https://peerj.com/	Forest trees cover just over 30% of the earth's surface and are studied by researchers around the world for both th	2016-08-27	2021-10-12 2:51	2021-12-07 20:0						en
T4D2R26A	journalArticle	2016	van Eeuwijk, Mar	What Should Students in Plant Breeding Know Abou			0011-183X	10.2135/cropsci2	https://dl.sciencesocieties.org/publications/cs/abstracts/56/5/2119		2016	2021-10-12 2:51	2021-10-12 2:51		2119	5	56		en
HFXX8865	journalArticle	2016	Burris, Jason Nei	Nanocomposite Adhesive of English ivy (Hedera helix): Bioproduction, Nanoparticle Isolation, and Molecular Analysis							2016	2021-10-12 2:51	2021-10-12 2:51						
DGTEGM4I	journalArticle	2016	Grossman, Walt,	A Case for Data Commons: Toward Data Science as			1521-9615	10.1109/MCSE.2	http://ieeexplore.ieee.org/document/7548983/		2016-09	2021-10-12 2:51	2021-10-12 2:51		20-Oct	5	18		
I8494IVV	journalArticle	2016	Perea, Jorge, Cla	Bioinformatic analysis of genotype by sequencing (G			1471-2164	10.1186/s12864-	http://bmcgenomics.biomedcentral.com/articles/10.1186/s12864-016-2827-7		2016-08	2021-10-12 2:51	2021-10-12 2:51		498	S5	17		en
QYHL4WSV	journalArticle	2016	Song, Matthew W	Bisulfite Sequence Analyses Using CyVerse Discove			2379-8068, 2379	10.1002/cppb.20	https://onlinelibrary.wiley.com/doi/abs/10.1002/cppb.20034		2016-09	2021-10-12 2:51	2021-10-12 2:51		510-529	3	1		en
BHUFFWB3	journalArticle	2016	Xu, Maria, Weijia	Supporting Efficient Content Based Collection Comparison for Curation							2016	2021-10-12 2:51	2021-12-03 18:5						
9VTLVN2Z	conferencePaper	2016	Swetnam, V., T. L	Scaling GIS analysis tasks from th		978-1-4503-4755-6		10.1145/294955(	http://dl.acm.org/citation.cfm?doid=2949550.2949573		2016	2021-10-12 2:51	2022-02-25 0:41		6-Jan				en
QIH8F7B7	conferencePaper	2016	Nakandala, Marl(	Anatomy of the SEAGrid Science (		978-1-4503-4755-6		10.1145/294955(	http://dl.acm.org/citation.cfm?doid=2949550.2949591		2016	2021-10-12 2:51	2021-10-12 2:51		8-Jan				en
2IWTQP3X	conferencePaper	2016	Scarborough, Ma	Case Study: Microservice Evolutio		978-1-4503-4755-6		10.1145/294955(	http://dl.acm.org/citation.cfm?doid=2949550.2949655		2016	2021-10-12 2:51	2021-10-12 2:51		5-Jan				en
J4XERGNM	conferencePaper	2016	Stewart, John Mi	Jetstream: performance, early exp		978-1-4503-4755-6		10.1145/294955(	http://dl.acm.org/citation.cfm?doid=2949550.2949639		2016	2021-10-12 2:51	2021-10-12 2:51		8-Jan				en
SE7I5UJL	journalArticle	2016	Morey, Frances M	RNA-Seq analysis of seasonal and individual variati			1471-2164	10.1186/s12864-	http://bmcgenomics.biomedcentral.com/articles/10.1186/s12864-016-3020-8		2016-12	2021-10-12 2:51	2021-10-12 2:51		720	1	17		en
PT29BHWG	journalArticle	2016	Zhang, Qifa, Jian	Building two indica rice reference genomes with Pac			2052-4463	10.1038/sdata.20	http://www.nature.com/articles/sdata201676		2016-12	2021-10-12 2:51	2021-10-12 2:51		160076	1	3		en
ZX8Z9AZZ	journalArticle	2016	Moore, Helen, Re	Trustworthy Policies for Distributed Repositories			1947-945X, 1947	10.2200/S00732I	http://www.morganclaypool.com/doi/10.2200/S00732ED1V01Y201609ICR051		2016-09-11	2021-10-12 2:51	2021-12-07 20:0		i-133	3	8		en
TCPZML7G	journalArticle	2016	Schnase, Laura,	Big Data Challenges in Climate Science: Improving t			2168-6831, 2473	10.1109/MGRS.2	http://ieeexplore.ieee.org/document/7570342/		2016-09	2021-10-12 2:51	2021-10-12 2:51		22-Oct	3	4		
6Z5BT7G4	journalArticle	2016	Mykles, Jonathor	Resources and Recommendations for Using Transci			1540-7063, 1557	10.1093/icb/icw0	https://academic.oup.com/icb/article-lookup/doi/10.1093/icb/icw083		2016-12	2021-10-12 2:51	2021-10-12 2:51		1183-1191	6	56		en
5SPG77VA	thesis	2016	Macrander, Jasoi	Venomics of Sea Anemones: A Bioinformatic Approach to Tissue Specific Venom Composition and Toxin Gene Family Evolution.							2016	2021-10-12 2:51	2021-10-12 2:51						
8KZM2PL7	journalArticle	2016	Yu, Jianming, Xia	Genomic prediction contributing to a promising globa			2055-0278	10.1038/nplants.	http://www.nature.com/articles/nplants2016150		2016-10	2021-10-12 2:51	2021-10-12 2:51		16150	10	2		en
S6MPIMUC	journalArticle	2016	Liu, Trupti, Yang;	PGen: large-scale genomic variations analysis workf			1471-2105	10.1186/s12859-	http://bmcbioinformatics.biomedcentral.com/articles/10.1186/s12859-016-1227-y		2016-10	2021-10-12 2:51	2021-10-12 2:51		337	S13	17		en
WMP4U62R	journalArticle	2016	Martin, H. Arthur,	Editorial for <i>Integrative and Comparative Biology-			1540-7063, 1557	10.1093/icb/icw1	https://academic.oup.com/icb/article-lookup/doi/10.1093/icb/icw114		2016-11	2021-10-12 2:51	2021-10-12 2:51		755-757	5	56		en
7CQ6WASY	journalArticle	2016	Wren, Shraddha,	Proceedings of the 2016 MidSouth Computational B			1471-2105	10.1186/s12859-	http://bmcbioinformatics.biomedcentral.com/articles/10.1186/s12859-016-1213-4		2016-10	2021-10-12 2:51	2021-10-12 2:51		356, s12859-016-1213-4	S13	17		en
7HT5CC7P	journalArticle	2016	Lambirth, Kennet	Transcript Polymorphism Rates in Soybean Seed Tis			1687-5370, 1687	10.1155/2016/15	https://www.hinda	Transgenic crops have been utilized for decades to enhance agriculture and more recently have been applied as b	2016	2021-10-12 2:51	2021-10-12 2:51		12-Jan		2016		en
XB8CQGJM	journalArticle	2016	Tara Oceans Coc	Ecogenomics and potential biogeochemical impacts			0028-0836, 1476	10.1038/nature1!	http://www.nature.com/articles/nature19366		2016-09	2021-10-12 2:51	2021-10-12 2:51		689-693	7622	537		en
YXAWQA9I	journalArticle	2016	Buttigieg, Christo	The environment ontology in 2016: bridging domains			2041-1480	10.1186/s13326-	http://jbiomedsem.biomedcentral.com/articles/10.1186/s13326-016-0097-6		2016-12	2021-10-12 2:51	2021-10-12 2:51		57	1	7		en
8TFFFSUY	book	2016	Leonelli, Sabina	Data-centric biology: A philosophical study							2016	2021-10-12 2:51	2021-10-12 2:51						
9P8SUM8Q	bookSection	2016	Gupta, Thomas,	Improving Re-annotation of Annota		978-3-319-41278-8 978-3-319-41279-5			http://link.springer.com/10.1007/978-3-319-41279-5_5		2016	2021-10-12 2:51	2021-10-12 2:51		171-195				en
2JNMGYNQ	journalArticle	2016	Halse-Gramkow,	Using evolutionary tools to search for novel psychoa			1479-2621, 1479	10.1017/S14792(	https://www.caml	Abstract Bioprospecting is the search for valuable products from natural sources. Given that most species are poor	2016-12	2021-10-12 2:51	2021-10-12 2:51		246-255	4	14		en
GJRT5YAB	journalArticle	2016	Martinez, Manue	Computational tools for genomic studies in plants							2016	2021-10-12 2:51	2021-10-12 2:51		509–514	6	17		
AUWSPM36	journalArticle	2016	Studer, Thomas I	The draft genome of the C3 panicoid grass species I			1474-760X	10.1186/s13059-	http://genomebiology.biomedcentral.com/articles/10.1186/s13059-016-1080-3		2016-12	2021-10-12 2:51	2021-10-12 2:51		223	1	17		en
2VDHRMWI	journalArticle	2016	Joyce, Eric, Blak(	FractBias: a graphical tool for assessing fractionatio			1367-4803, 1460	10.1093/bioinforr	https://academic.oup.com/bioinformatics/article-lookup/doi/10.1093/bioinformatics/btw666		2016-10-29	2021-10-12 2:51	2021-12-07 20:0		btw666				en
6VLH5XBL	journalArticle	2016	Vogelstein, Micha	To the Cloud! A Grassroots Proposal to Accelerate B			8966273	10.1016/j.neuron	https://linkinghub.elsevier.com/retrieve/pii/S0896627316307838		2016-11	2021-10-12 2:51	2021-10-12 2:51		622-627	3	92		en
UPZSEBGQ	conferencePaper	2016	Coulter, Craig, Er	Cyberinfrastructure as a Platform t		978-1-4503-4095-3		10.1145/2974927	http://dl.acm.org/citation.cfm?doid=2974927.2974962		2016	2021-10-12 2:51	2021-10-12 2:51		37-42				en
GXFTTHEG	journalArticle	2016	Wasike, T. A., C.	1683 Genome-wide association analysis of residual			0021-8812, 1525	10.2527/jam2016	https://academic.oup.com/jas/article/94/suppl_5/820/4767567		2016-10-01	2021-10-12 2:51	2021-12-07 20:0		820-820	suppl_5	94		en
WVDYB3QU	conferencePaper	2016	Tuecke, Ian, Stev	Globus Auth: A research identity and access management platform							2016	2021-10-12 2:51	2021-10-12 2:51		203–212				
BWFAPVYN	journalArticle	2016	Brozynska, Marta	Genomic characterisation of Australian wild rice species							2016	2021-10-12 2:51	2021-10-12 2:51						
I2XH9P6A	journalArticle	2016	Hamilton, Robert	Structure of the germline genome of Tetrahymena th			2050-084X	10.7554/eLife.19	https://elifescienc	The germline genome of the binucleated ciliate Tetrahymena thermophila undergoes programmed chromosome br	2016-11-28	2021-10-12 2:51	2021-12-07 20:0		e19090		5		en
PFUVS5DS	conferencePaper	2016	Lenhardt, Alyson	Cross-institutional research cyberi		978-1-5090-3525-0		10.1109/HPEC.2	http://ieeexplore.ieee.org/document/7761597/		2016-09	2021-10-12 2:51	2021-10-12 2:51		6-Jan				
WJJ3RY5J	journalArticle	2016	Roux, Matthew B	Towards quantitative viromics for both double-strand			2167-8359	10.7717/peerj.27	https://peerj.com/	Background Viruses strongly influence microbial population dynamics and ecosystem functions. However, our abilit	2016-12-08	2021-10-12 2:51	2021-12-07 20:0		e2777		4		en
F4R5GNAG	journalArticle	2016	Church, Andrzej I	Data and data servers exposed as services integrate			2043-9989, 2043	10.1504/IJCC.20	http://www.inderscience.com/link.php?id=80901		2016	2021-10-12 2:51	2021-10-12 2:51		264	4	5		en
278KVPDW	journalArticle	2016	Guralnick, Laura,	The importance of digitized biocollections as a sourc			1758-0463	10.1093/databas	https://academic.oup.com/database/article-lookup/doi/10.1093/database/baw158		2016	2021-10-12 2:51	2021-10-12 2:51		baw158		2016		en
SF9HA9BQ	journalArticle	2016	Hoopen, Pelin, P	Plant specimen contextual data consensus			2047-217X	10.1093/gigascie	https://academic.oup.com/gigascience/article/doi/10.1093/gigascience/giw002/2756883		2016-12-01	2021-10-12 2:51	2021-12-07 20:0			1	5		en
9TLWQ7VQ	thesis	2016	Kang, Yoonja	An investigation of pelagophyte ecology							2016	2021-10-12 2:51	2021-10-12 2:51						
YKF7KZJM	conferencePaper	2016	Furfaro, Ramona	Resident Space Object Characterization and Behavior Understanding via Machine Learning and Ontology-based Bayesian Networks							2016	2021-10-12 2:51	2021-10-12 2:51						
QXITU6AA	journalArticle	2016	Walls, Moriba, R;	Assessing the IADC Space Debris Mitigation Guidelines: A case for ontology-based data management							2016	2021-10-12 2:51	2021-10-12 2:51						
7K37GLDU	conferencePaper	2016	Xu, Patti, Weijia;	A Web Application for Extracting Key Domain Information for Scientific Publications Using Ontology.							2016	2021-10-12 2:51	2021-10-12 2:51						
EVKS2G2Q	conferencePaper	2016	Xu, Patti, Weijia;	Enhancing Information Accessibility of Scientific Publications with Text Mining and Ontology							2016	2021-10-12 2:51	2021-10-12 2:51		2–3		1747		
LUNQN8KU	conferencePaper	2016	Buttigieg, Christo	Environmental Semantics for Sustainable Development in an Interconnected Biosphere.							2016	2021-10-12 2:51	2021-10-12 2:51						
HXIZYEPV	conferencePaper	2016	Cooper, Pankaj; (	The Planteome Project.							2016	2021-10-12 2:51	2021-10-12 2:51						
KYLYKP9I	journalArticle	2016	Preece, Sinisa; o	Plant Image Segmentation and Annotation with Ontologies in BisQue.							2016	2021-10-12 2:51	2021-10-12 2:51						
EBCZDHED	conferencePaper	2016	Walls, Ramona L	Defining and Sustaining Populations and Communities.							2016	2021-10-12 2:51	2021-10-12 2:51						
G84K8FZD	conferencePaper	2016	Stubbs, Ann, Joe	Endofday: A Container Workflow Engine for Scalable, Reproducible Computation.							2016	2021-10-12 2:51	2021-10-12 2:51						
J7FJZR35	conferencePaper	2016	Bomhoff, Matt	Next-Gen Sequence Analysis in CoGe: Read Alignment, Expression Analysis, SNP Ider					https://pag.confex.com/pag/xxiv/webprogram/Paper18549.html		2016	2021-10-12 2:51	2021-10-12 2:51				W411		
7DZNIDK3	conferencePaper	2016	DeBarry, Jeremy	Integration of CoGe's Data Services in iPlant					https://pag.confex.com/pag/xxiv/webprogram/Paper18554.html		2016	2021-10-12 2:51	2021-10-12 2:51				W415		
PARNZWKB	conferencePaper	2016	Dill, Carolyn	Genomes to Fields (G2F)					https://pag.confex.com/pag/xxiv/webprogram/Paper21710.html		2016	2021-10-12 2:51	2021-10-12 2:51				W585		
3QIU7KW6	conferencePaper	2016	Dvorak, Jan, et a	Reference Sequence of the Genome of Aegilops tauschii, the Progenitor of the Wheat D					https://pag.confex.com/pag/xxiv/webprogram/Paper20195.html		2016	2021-10-12 2:51	2021-10-12 2:51				W972		
KAMKVF4T	conferencePaper	2016	Goller, Carlos C.	Sifting Through Metagenomes using the iPlant Discovery Environment					https://pag.confex.com/pag/xxiv/webprogram/Paper21872.html		2016	2021-10-12 2:51	2021-10-12 2:51				W558		
S4SPCYAY	conferencePaper	2016	Harris, Stephen	Using DNA Barcoding and Educational Bioinformatics to Create Authentic Research Ex					https://pag.confex.com/pag/xxiv/webprogram/Paper21871.html		2016	2021-10-12 2:51	2021-10-12 2:51				W556		
KYGV4LRX	conferencePaper	2016	Krishnakumar, Vi	Teaching Bioinformatics Data Analysis Using Cloud Computing Technology					https://pag.confex.com/pag/xxiv/webprogram/Paper22324.html		2016	2021-10-12 2:51	2021-10-12 2:51				W940		
V894WNW9	conferencePaper	2016	Lee, Joslynn	Creating a Positive Space to Train Native Americans / American Indians / Alaskan Nativ					https://pag.confex.com/pag/xxiv/webprogram/Paper22088.html		2016	2021-10-12 2:51	2021-10-12 2:51				W560		
LEN4C2EA	conferencePaper	2016	Lushbough, Carc	Bioinformatics Tutorials leveraging the BioExtract Server					https://pag.confex.com/pag/xxiv/webprogram/Paper19688.html		2016	2021-10-12 2:51	2021-10-12 2:51				W936		
8KQERQNG	conferencePaper	2016	Teal, Tracy	Data Carpentry: Data Skills Training to Enable More Effective Research					https://pag.confex.com/pag/xxiv/webprogram/Paper21881.html		2016	2021-10-12 2:51	2021-10-12 2:51				W559		
D4PC9TIN	conferencePaper	2016	Thurber, Carrie	RNA-Seq in the Classroom: Pathways to Undergraduate Research					https://pag.confex.com/pag/xxiv/webprogram/Paper21870.html		2016	2021-10-12 2:51	2021-10-12 2:51				W557		
5FSKPSQS	conferencePaper	2016	Antin, Parker	Introducing CyVerse					https://pag.confex.com/pag/xxiv/webprogram/Paper21734.html		2016	2021-10-12 2:51	2021-10-12 2:51				W242		
IBT9BG3N	conferencePaper	2016	Castelaanos, JA	Integrated, Accurate and Multi-Environment Structural Variation Discovery from Whole (					https://pag.confex.com/pag/xxiv/webprogram/Paper19823.html		2016	2021-10-12 2:51	2022-03-04 15:5				P19823		
I69V4SRK	conferencePaper	2016	Cottingham, Rob	The DOE Systems Biology Knowledgebase (KBase): Introduction to a System for Collal					https://pag.confex.com/pag/xxiv/webprogram/Paper19547.html		2016	2021-10-12 2:51	2021-10-12 2:51				P1278		
XTF43ZEG	conferencePaper	2016	DeBarry, Jeremy	How to do Big-Data Science - Data Management for Genomics					https://pag.confex.com/pag/xxiv/webprogram/Paper21738.html		2016	2021-10-12 2:51	2021-10-12 2:51				W246		
DCSLCS5G	conferencePaper	2016	Fritz-Waters, Eric	The iPlant Agave Application Program Interface, High Performance Computing, and You					https://pag.confex.com/pag/xxiv/webprogram/Paper21735.html		2016	2021-10-12 2:51	2021-10-12 2:51				W245		
UJTCPH94	conferencePaper	2016	Gardiner, Jack, e	Data Management and Analysis Solutions for Maize Predictive Phenomics: A Partnersh					https://pag.confex.com/pag/xxiv/webprogram/Paper21167.html		2016	2021-10-12 2:51	2021-10-12 2:51				P0319		
8N35UTH5	conferencePaper	2016	Hendre, Prasad,	African Orphan Crops Consortium: a Global Partnership to Address Food and Nutritiona					https://pag.confex.com/pag/xxiv/webprogram/Paper22498.html		2016	2021-10-12 2:51	2021-10-12 2:51				P1309		
8DPHWEJS	conferencePaper	2016	Hubbard, Allen, e	fRNAkenseq: a Powered-by-iPlant RNA Sequencing Analysis Platform					https://pag.confex.com/pag/xxiv/webprogram/Paper19675.html		2016	2021-10-12 2:51	2021-10-12 2:51				P0358		
WZPPYBHX	conferencePaper	2016	Johnson, Ryan	iPlantUK: Creating User Friendly RNA-Seq and Mapping by Sequencing Workflows					https://pag.confex.com/pag/xxiv/webprogram/Paper22479.html		2016	2021-10-12 2:51	2021-10-12 2:51				P1292		
HJS6IASD	conferencePaper	2016	Luo, Ming-Cheng	Integrated Approach Towards Sequencing a Large and Complex Genome - iPlant Porta					https://pag.confex.com/pag/xxiv/webprogram/Paper21777.html		2016	2021-10-12 2:51	2021-10-12 2:51				W243		
DVLIDXMA	conferencePaper	2016	Marla, Sandeep I	Functional Genomics of Seedling Cold Adaptation in Sorghum bicolor					https://pag.confex.com/pag/xxiv/webprogram/Paper22509.html		2016	2021-10-12 2:51	2021-10-12 2:51				P1305		
SKVVIM3P	conferencePaper	2016	Nelson, Andrew I	Evolnc: A Pipeline for Comparative Genomic and Transcriptomic Analyses of Long Non-					https://pag.confex.com/pag/xxiv/webprogram/Paper22209.html		2016	2021-10-12 2:51	2021-10-12 2:51				W244		
PP92733C	conferencePaper	2016	Phan, L	NCBI Variation Portal: Databases and Tools for Genetic Variation Discovery, Analysis, a					https://pag.confex.com/pag/xxiv/webprogram/Paper21034.html		2016	2021-10-12 2:51	2021-10-12 2:51				P21034		
EYRKGLNV	conferencePaper	2016	Farmer, Andrew I	The Legume Information System and The Legume Federation: Working Together for the					https://pag.confex.com/pag/xxiv/webprogram/Paper21325.html		2016	2021-10-12 2:51	2021-10-12 2:51				C18		
F7W7KY5F	conferencePaper	2016	Stein, Joshua, et	Exploring and Comparing Plant Genomes Using the Gramene/Ensembl Plants Browser					https://pag.confex.com/pag/xxiv/webprogram/Paper21917.html		2016	2021-10-12 2:51	2021-10-12 2:51				P0720		
JIMHJF9V	journalArticle	2016	Brum, Jennifer R	Illuminating struc	Proceedings of the National Acade		0027-8424, 1091	10.1073/pnas.15	http://www.pnas.(	Viruses are ecologically important, yet environmental virology is limited by dominance of unannotated genomic seq	2016-03-01	2021-10-12 2:51	2021-10-12 2:51	2021-10-12 2:51	2436-2441	9	113	Proc Natl Acad Sci USA	en
NJDW55FB	conferencePaper	2016	Xu, Ramona, We	Content-based comparison for collections identification							2016	2021-10-12 2:55	2021-10-12 2:55		3283–3289				
5UB8X226	journalArticle	2015	Zulfiqar, Shumail	Role of genetic s	Nature Sci						2015	2021-09-07 21:5	2021-09-07 21:5				13		
88DMHTLK	journalArticle	2015	Antoni, Angelika;	A Bioinformatics Approach to Systemic Lupus Erythematosus and Autoimmunity					https://www.aai.org/AAISite/media/Education/HST/Archive/2015_Seifert_Final.pdf		2015	2021-09-07 21:5	2021-10-14 19:2						
J9B4NKTM	journalArticle	2015	Dooley, Matthew	Recipes 2.0: building for today and tomorrow: RECIF			15320626	10.1002/cpe.328	http://doi.wiley.com/10.1002/cpe.3285		2015-02	2021-10-12 2:33	2021-10-12 2:33		258-270	2	27		en
AX4SKLXA	journalArticle	2015	Kociumaka, Tomi	Fast algorithm for partial covers in words							2015	2021-10-12 2:33	2021-10-12 2:33		217–233	1	73		
QSY6HB27	journalArticle	2015	Law, Mark, MeiYe	Automated Update, Revision, and Quality Control of			0032-0889, 1532	10.1104/pp.114.2	http://www.plantphysiol.org/lookup/doi/10.1104/pp.114.245027		2015-01	2021-10-12 2:35	2021-10-12 2:35		25-39	1	167		en
P7FGKL4M	journalArticle	2015	Wang, Lincoln, Li	A genome-wide association study platform built on iF			15320626	10.1002/cpe.323	http://doi.wiley.com/10.1002/cpe.3236		2015-02	2021-10-12 2:35	2021-10-12 2:35		420-432	2	27		en
SNN6XHET	journalArticle	2015	Lushbough, Rion	Life science data analysis workflow development usi			15320626	10.1002/cpe.323	http://doi.wiley.com/10.1002/cpe.3237		2015-02	2021-10-12 2:35	2021-10-12 2:35		408-419	2	27		en
8CC3GUXL	journalArticle	2015	Crivelli, Stephen;	Creating a gateway that enables large-scale science			15320626	10.1002/cpe.327	http://doi.wiley.com/10.1002/cpe.3270		2015-02	2021-10-12 2:35	2021-10-12 2:35		446-457	2	27		en
62MEQE7X	bookSection	2015	Ma, Arthur G., Liu	A 3′ RACE Protocol to Confirm Pol		978-1-4939-2174-4 978-1-4939-2175-1			http://link.springer.com/10.1007/978-1-4939-2175-1_12		2015	2021-10-12 2:35	2021-10-12 2:35		135-144		1255		
S69NS5T9	journalArticle	2015	Poldrack, Jean-B	The publication and reproducibility challenges of sha			13646613	10.1016/j.tics.20	https://linkinghub.elsevier.com/retrieve/pii/S1364661314002526		2015-02	2021-10-12 2:35	2021-10-12 2:35		59-61	2	19		en
H5X366A5	journalArticle	2015	Hurwitz, Matthew	Depth-stratified functional and taxonomic niche spec			1751-7362, 1751	10.1038/ismej.20	http://www.nature.com/articles/ismej2014143		2015-02	2021-10-12 2:35	2021-10-12 2:35		472-484	2	9		en
3Q6YUGC8	journalArticle	2015	Mirarab, Tandy, S	PASTA: Ultra-Large Multiple Sequence Alignment fo			1066-5277, 1557	10.1089/cmb.201	http://www.liebertpub.com/doi/10.1089/cmb.2014.0156		2015-05	2021-10-12 2:35	2021-10-12 2:35		377-386	5	22		en
LCTZ45BD	journalArticle	2015	Martin, R., L.; Co	Data mining with iPlant: A meeting report from the 20			0022-0957, 1460	10.1093/jxb/eru4	https://academic.oup.com/jxb/article-lookup/doi/10.1093/jxb/eru402		2015-01-01	2021-10-12 2:35	2021-12-07 19:4		6-Jan	1	66		en
RHJW38NQ	journalArticle	2015	Cannon, Jim, Ste	Multiple Polyploidy Events in the Early Radiation of I			1537-1719, 0737	10.1093/molbev/	https://academic.oup.com/mbe/article-lookup/doi/10.1093/molbev/msu296		2015-01	2021-10-12 2:35	2021-10-12 2:35		193-210	1	32		en
BIRBKN85	journalArticle	2015	Krishnakumar, Cl	Araport: the Arabidopsis Information Portal			1362-4962, 0305	10.1093/nar/gku1	http://academic.oup.com/nar/article/43/D1/D1003/2439074/Araport-the-Arabidopsis-Information-Portal		2015-01-28	2021-10-12 2:35	2021-12-07 19:4		D1003-D1009	D1	43		en
FPWIHEIS	journalArticle	2015	Poelchau, Kevin,	The i5k Workspace@NAL—enabling genomic data a			1362-4962, 0305	10.1093/nar/gku9	http://academic.oup.com/nar/article/43/D1/D714/2439695/The-i5k-WorkspaceNALenabling-genomic-data-access		2015-01-28	2021-10-12 2:35	2021-12-07 19:5		D714-D719	D1	43		en
5KZPGUID	journalArticle	2015	Williams, Brent J	Cold truths: how winter drives responses of terrestria			14647931	10.1111/brv.1210	http://doi.wiley.com/10.1111/brv.12105		2015-02	2021-10-12 2:35	2021-10-12 2:35		214-235	1	90		en
HRF9FTLL	journalArticle	2015	Šímová, Brian J.,	Shifts in trait means and variances in North America			9067590	10.1111/ecog.00	http://doi.wiley.com/10.1111/ecog.00867		2015-07	2021-10-12 2:35	2021-10-12 2:35		649-658	7	38		en

ISEEFNP4	journalArticle	2015	Sullivan, Matthew	Viromes, Not Gene Markers, for Studying Double-St		0022-538X, 1098	10.1128/JVI.0328	http://jvi.asm.org/	Microbes have recently been recognized as dominant forces in nature, with studies benefiting from gene markers t	2015-03-01	2021-10-12 2:35	2021-12-07 19:5	2459-2461	5	89	en
CSACYHAM	journalArticle	2015	Ananthakrishnan	Globus platform-as-a-service for collaborative science applications						2015	2021-10-12 2:36	2021-10-12 2:36	290–305	2	27	
JB3PFXXE	conferencePaper	2015	Liu, Yang et al.	Large-Scale Next-Generation Resequencing of Soybean Germplasm for Trait Discovery						2015-01-12	2021-10-12 2:36	2021-12-07 19:4				
TNZIZEU9	journalArticle	2015	Macías-Duarte, C	Spatial patterns in hydrogen isotope ratios in feather		0004-8038, 1938	10.1642/AUK-13	https://academic.oup.com/auk/article/132/1/25-36/5149172		2015-01	2021-10-12 2:38	2021-10-12 2:38	25-36	1	132	en
ZUR4TIK7	journalArticle	2015	Rocca-Serra, A.,	Modeling a microbial community and biodiversity as		1758-0463	10.1093/databas	https://academic.oup.com/database/article-lookup/doi/10.1093/database/bau132		2015-01-28	2021-10-12 2:38	2021-12-07 19:5	bau132-bau132	0	2015	en
FW9Y6CP8	journalArticle	2015	Deans, Paula, Ar	Finding Our Way through Phenotypes		1545-7885	10.1371/journal.p	http://dx.plos.org/10.1371/journal.pbio.1002033		2015-01-06	2021-10-12 2:38	2021-12-07 19:5	e1002033	1	13	en
547SWR62	conferencePaper	2015	Brusslan, Judy A	Large Dataset Analysis for Undergraduates: Discovery Environment, RNA-Seq and Lea				https://pag.confex.com/pag/xxiii/webprogram/Paper17290.html		2015	2021-10-12 2:38	2021-10-12 2:38				
WDQCWC2F	conferencePaper	2015	Burnette, James	Transposable Elements, Gene Discovery, and DNA Barcoding (Yellow and Blue Lines)				https://pag.confex.com/pag/xxiii/webprogram/Paper17292.html		2015	2021-10-12 2:38	2021-10-12 2:38				
YEYB35YJ	conferencePaper	2015	Dvorak, Jan et al	BAC-Based Sequencing of the Aegilops tauschii Genome				https://pag.confex.com/pag/xxiii/webprogram/Paper13960.html		2015	2021-10-12 2:39	2021-10-12 2:39				
T3I6ACJU	conferencePaper	2015	Makarevitch, Irina	Bringing Authentic Genomics Research into the Classroom: Analysis of Maize Stress Re				https://pag.confex.com/pag/xxiii/webprogram/Paper17287.html		2015	2021-10-12 2:39	2021-10-12 2:39				
ZYTQS8AQ	conferencePaper	2015	Micklos, Dave	Simplifying Genome Annotation and Examples of Large-Scale Projects in DNA Barcodin				https://pag.confex.com/pag/xxiii/webprogram/Paper17295.html		2015	2021-10-12 2:39	2021-10-12 2:39				
I9HTB59W	conferencePaper	2015	Subramaniam, S	Management and Visualization of Ianimal Quantitative Data Using iPlant and EPIC-CoG				https://pag.confex.com/pag/xxiii/webprogram/Paper14925.html		2015	2021-10-12 2:39	2021-10-12 2:39				
3QIVW986	conferencePaper	2015	Buckner, Brent	Introducing Undergraduate Students to Gene and Genome Structure through Gene Ann				https://pag.confex.com/pag/xxiii/webprogram/Paper17143.html		2015	2021-10-12 2:39	2021-10-12 2:39				
AXZPZSS2	conferencePaper	2015	Buonaccorsi, Vin	Undergraduate Genomics Research through GCAT-SEEK: The Genome Consortium fo				https://pag.confex.com/pag/xxiii/webprogram/Paper17140.html		2015	2021-10-12 2:39	2021-10-12 2:39				
7SVB3Z4K	conferencePaper	2015	Micklos, Dave	Using Students' Own DNA Sequences to Explore Human Evolution				https://pag.confex.com/pag/xxiii/webprogram/Paper17141.html		2015	2021-10-12 2:39	2021-10-12 2:39				
RITJJ9BR	conferencePaper	2015	Bailey, Donovan	Using iPlant Tools and Plastome Sequencing As a Springboard into Comparative Genor				https://pag.confex.com/pag/xxiii/webprogram/Paper17194.html		2015	2021-10-12 2:39	2021-10-12 2:39				
2NZMIVU2	conferencePaper	2015	Burnette, James	DNA Subway Yellow and Blue Lines: TEs, Gene Discovery and DNA Barcoding				https://pag.confex.com/pag/xxiii/webprogram/Paper17293.html		2015	2021-10-12 2:39	2021-10-12 2:39				
NSKSXLPT	conferencePaper	2015	Carson, James e	Leveraging iPlant Cyberinfrastructure for a New Data-Driven Research Community				https://pag.confex.com/pag/xxiii/webprogram/Paper15848.html		2015	2021-10-12 2:39	2021-10-12 2:39				
MH57K9G9	conferencePaper	2015	Cheng, Chia-Yi; I	Data Integration for the Plant Research Community: The Arabidopsis Information Portal				https://pag.confe	The Arabidopsis Information Portal (AIP; http://www.araport.org) is a new on-line resource for plant biology researc	2015	2021-10-12 2:39	2021-10-12 2:39				
WNI52NNE	conferencePaper	2015	Chougule, Kapee	Flash Demo: Atmosphere Cloud Computing - Applied to Visualizing RNA-Seq Data				https://pag.confex.com/pag/xxiii/webprogram/Paper17300.html		2015	2021-10-12 2:39	2021-10-12 2:39				
W54ITCFP	conferencePaper	2015	Cottingham, Rob	The DOE Systems Biology Knowledgebase: Progress Toward a System for Collaborativ				https://pag.confex.com/pag/xxiii/webprogram/Paper16317.html		2015	2021-10-12 2:39	2021-10-12 2:39				
XL2H5K8E	conferencePaper	2015	DeBarry, Jermey	Flash Demo: Secure, Collaborative, Management of Your Life Sciences Data with the D				https://pag.confex.com/pag/xxiii/webprogram/Paper17299.html		2015	2021-10-12 2:39	2021-10-12 2:39				
RQV833RC	conferencePaper	2015	Devisetty, Upend	A Hybrid Approach to Assemble and Annotate the Brassica rapa Transcriptome in the C				https://pag.confex.com/pag/xxiii/webprogram/Paper17195.html		2015	2021-10-12 2:39	2021-10-12 2:39				
NXK43GEJ	conferencePaper	2015	Gardiner, Jack; D	Identification and Development of Data Standards for Maize Field Phenotypic Measurer				https://pag.confex.com/pag/xxiii/webprogram/Paper15771.html		2015	2021-10-12 2:39	2021-10-12 2:39				
6JKU4FWU	conferencePaper	2015	Harper, Lisa et al	An Ontology Approach to Comparative Phenomics in the Plants: Arabidopsis, Tomato, M				https://pag.confex.com/pag/xxiii/webprogram/Paper15793.html		2015	2021-10-12 2:39	2021-10-12 2:39				
NINHQEMU	conferencePaper	2015	Haug-Baltzell, Er	Receptors DRD1a and DRD1b Arise from an Ancient Vertebrate Whole Genome Duplic				https://pag.confex.com/pag/xxiii/webprogram/Paper16214.html		2015	2021-10-12 2:39	2021-10-12 2:39				
X72YDCQ6	conferencePaper	2015	Huala, Eva et al	Flash Talk: An Ontology Approach to Comparative Phenomics in Plants				https://pag.confex.com/pag/xxiii/webprogram/Paper17309.html		2015	2021-10-12 2:39	2021-10-12 2:39				
TRA74G9P	conferencePaper	2015	McCarthy, Fiona	Functional Analysis of Your RNAseq Data				https://pag.confex.com/pag/xxiii/webprogram/Paper16866.html		2015	2021-10-12 2:39	2021-10-12 2:39				
ZI5PXHHC	conferencePaper	2015	Nelson, Andrew I	Utilizing iPlant to Unearth Long Non-Coding RNAs and Characterize Their Evolution in t				https://pag.confex.com/pag/xxiii/webprogram/Paper16865.html		2015	2021-10-12 2:39	2021-10-12 2:39				
BCM3TH2D	conferencePaper	2015	Subramaniam, S	Flash Demo: Annotating genomes with MAKER-P and the iPlant Discovery Environmen				https://pag.confex.com/pag/xxiii/webprogram/Paper17298.html		2015	2021-10-12 2:39	2021-10-12 2:39				
BYGBGEAE	conferencePaper	2015	Taylor, Daniel B	Life Sciences Cyberinfrastructure at Your Fingertips: Using Internet2 to Power Scientific				https://pag.confex.com/pag/xxiii/webprogram/Paper17271.html		2015	2021-10-12 2:39	2021-10-12 2:39				
KBXDNSDN	conferencePaper	2015	Vaughn, Matt et a	Developing Content for the Arabidopsis Information Portal				https://pag.confex.com/pag/xxiii/webprogram/Paper16142.html		2015	2021-10-12 2:39	2021-10-12 2:39				
JXNY5LUT	conferencePaper	2015	Vaughn, journal=	What's Talking?						2015	2021-10-12 2:39	2021-10-12 2:39	59			
QXV2MLM7	journalArticle	2015	Ohyanagi, Kenta	Editorial: Plant and Cell Physiology's 2015 Database		1471-9053, 0032	10.1093/pcp/pcu	https://academic.oup.com/pcp/article-lookup/doi/10.1093/pcp/pcu206		2015-01	2021-10-12 2:39	2021-10-12 2:39	6-Apr	1	56	en
97PLKWIS	journalArticle	2015	Engemann, Jens	Limited sampling hampers "big data" estimation of s		20457758	10.1002/ece3.14	http://doi.wiley.com/10.1002/ece3.1405		2015-02	2021-10-12 2:39	2021-10-12 2:39	807-820	3	5	en
W5ASHETB	journalArticle	2015	Parmentier, Mark	Using multi-timescale methods and satellite-derived		8998418	10.1002/joc.4251	http://doi.wiley.com/10.1002/joc.4251		2015-11-15	2021-10-12 2:39	2021-12-07 19:5	3862-3878	13	35	en
4X3FT5PJ	journalArticle	2015	Darwish, Nadim	Re-annotation of the woodland strawberry (Fragaria		1471-2164	10.1186/s12864-	http://www.biomedcentral.com/1471-2164/16/29		2015	2021-10-12 2:39	2021-10-12 2:39	29	1	16	en
CNKR67XF	journalArticle	2015	Albert, Christoph	Using basic plant traits to predict ungulate seed disp		9067590	10.1111/ecog.007	http://doi.wiley.com/10.1111/ecog.00709		2015-05	2021-10-12 2:39	2021-10-12 2:39	440-449	5	38	en
YBMF7STK	journalArticle	2015	Deng, Douglas E	Phylogeny, divergence times, and historical biogeog		10557903	10.1016/j.ympev.	https://linkinghub.elsevier.com/retrieve/pii/S1055790314003959		2015-02	2021-10-12 2:39	2021-10-12 2:39	86-98		83	en
NF25NIHQ	journalArticle	2015	Huang, Jason H,	Big data - a 21st century science Maginot Line? No-		1756-0381	10.1186/s13040-	https://biodatamining.biomedcentral.com/articles/10.1186/s13040-015-0037-5		2015-06	2021-10-12 2:39	2021-10-12 2:39	7	1	8	en
R9PWEAQM	journalArticle	2015	Wells, Douglas G	A genome-wide analysis of MADS-box genes in pea		1471-2229	10.1186/s12870-	http://www.biomedcentral.com/1471-2229/15/41		2015	2021-10-12 2:39	2021-10-12 2:39	41	1	15	en
TLE957EX	journalArticle	2015	Tang, Christophe	Optical mapping in plant comparative genomics		2047-217X	10.1186/s13742-	https://academic.oup.com/gigascience/article-lookup/doi/10.1186/s13742-015-0044-y		2015-12	2021-10-12 2:39	2021-10-12 2:39	3	1	4	en
APLWZHUV	journalArticle	2015	Yang, James M.,	Gene Co-Expression Network Analysis Provides No		1932-6203	10.1371/journal.p	https://dx.plos.org/10.1371/journal.pone.0117607		2015-02-19	2021-10-12 2:39	2021-12-07 19:5	e0117607	2	10	en
7EUCLYYA	journalArticle	2015	Oellrich, Eva, An	An ontology approach to comparative phenomics in		1746-4811	10.1186/s13007-	http://www.plantmethods.com/content/11/1/10		2015	2021-10-12 2:39	2021-10-12 2:39	10	1	11	en
TJN3CW4E	journalArticle	2015	Horvath, Sharon	RNAseq reveals weed-induced PIF3-like as a candi		0028646X	10.1111/nph.133	http://doi.wiley.com/10.1111/nph.13351		2015-07	2021-10-12 2:39	2021-10-12 2:39	196-210	1	207	en
PSJTZISX	journalArticle	2015	Fahlgren, Ivan, N	Lights, camera, action: high-throughput plant pheno		13695266	10.1016/j.pbi.201	https://linkinghub.elsevier.com/retrieve/pii/S1369526615000266		2015-04	2021-10-12 2:39	2021-10-12 2:39	93-99		24	en
DFW2IYKJ	journalArticle	2015	Macías-Duarte, C	Distributional Changes in the Western Burrowing Ov		0892-1016, 2162	10.3356/JRR-14-	http://www.bioone.org/doi/abs/10.3356/JRR-14-00004.1		2015-03	2021-10-12 2:39	2021-10-12 2:39	75-83	1	49	en
JSAAY2AL	journalArticle	2015	Shipunov, Jason,	Flora of North Dakota project						2015	2021-10-12 2:39	2021-10-12 2:39	1–10		16	
IGBB5JWX	journalArticle	2015	Zulfiqar, Qurban,	Role of genetic studies towards solving problems of human society				http://www.sciencepub.net/nature/ns130415/003_28241ns130415_15_19.pdf		2015	2021-10-12 2:39	2021-12-03 18:5		14	13	
EUGEHSRF	journalArticle	2015	Madduri, Ian, Ra	The Globus Galaxies platform: delivering science gateways as a service						2015	2021-10-12 2:39	2021-10-12 2:39	4344–4360	16	27	
242XHGQ4	journalArticle	2015	Dhanapal, Mahal	Unlimited thirst for genome sequencing, data interpretation, and database usage in genomic era: the road towards fast-track crop plant improvement						2015	2021-10-12 2:39	2021-10-12 2:39			2015	
RQGWA2JD	journalArticle	2015	Batllori, Max A, E	A minimal model of fire-vegetation feedbacks and di		1748-9326	10.1088/1748-93	http://stacks.iop.org/1748-9326/10/i=3/a=034018?key=crossref.8ec0002e5ea92d165adc69409bd95a14		2015-03-01	2021-10-12 2:39	2021-12-07 19:5	34018	3	10	
PFWVVGAM	journalArticle	2015	Goggin, Christop	Applying high-throughput phenotyping to plant–insec		22145745	10.1016/j.cois.20	https://linkinghub.elsevier.com/retrieve/pii/S2214574515000334		2015-06	2021-10-12 2:39	2021-10-12 2:39	69-76		9	en
7477ZKCD	journalArticle	2015	Goring, John W.,	neotoma: A Programmatic Interface to the Neotoma		2055-298X	10.5334/oq.ab	http://www.openquaternary.com/articles/10.5334/oq.ab/		2015-03-09	2021-10-12 2:39	2021-12-07 19:5			1	en
QFZGULXZ	journalArticle	2015	Thompson, Gran	Genomes of ' <i>Candidatus</i> Liberibacter solana		0031-949X	10.1094/PHYTO-	http://apsjournals.apsnet.org/doi/10.1094/PHYTO-12-14-0363-FI		2015-07	2021-10-12 2:39	2021-10-12 2:39	863-871	7	105	en
I4P5NXPL	journalArticle	2015	Albert, Christoph	Seed dispersal by ungulates as an ecological filter: a		301299	10.1111/oik.0251	http://doi.wiley.com/10.1111/oik.02512		2015-09	2021-10-12 2:39	2021-10-12 2:39	1109-1120	9	124	en
2HB5MIR3	journalArticle	2015	Chamala, Pamela	MarkerMiner 1.0: A New Application for Phylogenetic		2168-0450	10.3732/apps.14	http://doi.wiley.com/10.3732/apps.1400115		2015-04	2021-10-12 2:39	2021-10-12 22:4	1400115	4	3	en
NP2P6UUF	journalArticle	2015	Guralnick, Roder	Community Next Steps for Making Globally Unique I		1313-2970, 1313	10.3897/zookeys	http://zookeys.pensoft.net/articles.php?id=5042		2015-04-06	2021-10-12 2:39	2021-12-07 19:5	133-154		494	
A7P8YND7	journalArticle	2015	Köhl, Jürgen, Ka	A software tool for the input and management of phe		1746-4811	10.1186/s13007-	http://www.plantmethods.com/content/11/1/25		2015-12	2021-10-12 2:39	2021-10-12 2:39	25	1	11	en
GUS78SUG	journalArticle	2015	Sadler, Jeffrey M	Hydrologic Data Sharing Using Open Source Software and Low-Cost Electronics						2015	2021-10-12 2:39	2021-10-12 2:39				
4HLAZBHX	journalArticle	2015	Eastmond, Julian	Arabidopsis uses two gluconeogenic gateways for o		2041-1723	10.1038/ncomms	http://www.nature.com/articles/ncomms7659		2015-05	2021-10-12 2:39	2021-10-12 2:39	6659	1	6	en
U6YJSUWJ	conferencePaper	2015	Fischer, Craig A,	Jetstream: A distributed cloud infrastructure for underresourced higher education communities						2015	2021-10-12 2:39	2021-10-12 2:39	53–61			
GH8LM8TY	journalArticle	2015	Li, Nathan M., Qi	Examining the Causes and Consequences of Conte		0032-0889, 1532	10.1104/pp.15.00	http://www.plantphysiol.org/lookup/doi/10.1104/pp.15.00052		2015-08	2021-10-12 2:39	2021-10-12 2:39	1262-1274	4	168	en
X8LKIMRH	journalArticle	2015	Blonder, Brian J.,	Linking environmental filtering and disequilibrium to		0012-9658	10.1890/14-0589	http://doi.wiley.com/10.1890/14-0589.1		2015-04	2021-10-12 2:39	2021-10-12 2:39	972-985	4	96	en
B43Z7BBD	journalArticle	2015	Twyford, Jannice	Adaptive divergence in the monkey flower <i>Mimulu		143820	10.1111/evo.126	http://doi.wiley.com/10.1111/evo.12663		2015-06	2021-10-12 2:39	2021-10-12 2:39	1476-1486	6	69	en
CKU5II58	journalArticle	2015	Li, Jianlin, Jilong;	From Gigabyte to Kilobyte: A Bioinformatics Protoco		1932-6203	10.1371/journal.p	https://dx.plos.org/10.1371/journal.pone.0125000		2015-04-22	2021-10-12 2:39	2021-12-07 19:5	e0125000	4	10	en
GSSAP5WT	journalArticle	2015	Taylor, John M., I	Transport Function of Rice Amino Acid Permeases (		0032-0781, 1471	10.1093/pcp/pcv	https://academic.oup.com/pcp/article-lookup/doi/10.1093/pcp/pcv053		2015-07	2021-10-12 2:39	2021-10-12 2:39	1355-1363	7	56	en
X6UTVJ99	journalArticle	2015	Liu, Andrew, Lijun	A resource for characterizing genome-wide binding a		9607412	10.1111/tpj.1285	http://doi.wiley.com/10.1111/tpj.12850		2015-06	2021-10-12 2:39	2021-10-12 2:39	887-898	5	82	en
E2EDEN8F	bookSection	2015	González, César	Physiological Responses of N2-Fi	978-3-319-06211-2 978-3-319-06212-9			http://link.springer.com/10.1007/978-3-319-06212-9_2		2015	2021-10-12 2:39	2021-10-12 2:39	May-33			en
ZWULWFBE	journalArticle	2015	Noutsos, Doreen	Metabolomic Profiling of the Nectars of Aquilegia pu		1932-6203	10.1371/journal.p	https://dx.plos.org/10.1371/journal.pone.0124501		2015-05-01	2021-10-12 2:39	2021-12-07 19:5	e0124501	5	10	en
YJ6UBZD6	conferencePaper	2015	Fox, Andre, Geof	HPC-ABDS High Performance Co	978-1-4799-8006-2		10.1109/CCGrid.	http://ieeexplore.ieee.org/document/7152592/		2015-05	2021-10-12 2:39	2021-10-12 2:39	1057-1066			
TS6BKQBA	journalArticle	2015	Townsley, Neelim	BrAD-seq: Breath Adapter Directional sequencing: a		1664-462X	10.3389/fpls.201	http://www.frontiersin.org/Plant_Evolution_and_Development/10.3389/fpls.2015.00366/abstract		2015-05-22	2021-10-12 2:39	2021-12-07 19:5			6	
A5GFGZW3	journalArticle	2015	Deck, John, John	Meeting report: Identifying practical applications of o		1944-3277	10.1186/s40793-	https://standardsingenomics.biomedcentral.com/articles/10.1186/s40793-015-0014-0		2015-12	2021-10-12 2:39	2021-10-12 2:39	25	1	10	en
46NN8JLA	journalArticle	2015	Hanlon, Eleanor,	Araport: an application platform for data discovery: A		15320626	10.1002/cpe.354	http://doi.wiley.com/10.1002/cpe.3542		2015-11	2021-10-12 2:39	2021-10-12 2:39	4412-4422	16	27	en
PBMNR3UF	journalArticle	2015	Roux, Matthew B	VirSorter: mining viral signal from microbial genomic		2167-8359	10.7717/peerj.98	https://peerj.com/articles/985		2015-05-28	2021-10-12 2:39	2021-12-07 19:5	e985		3	en
A3XDR98R	journalArticle	2015	Zhu, Jian, Yongn	PathRings: a web-based tool for exploration of ortho		1471-2105	10.1186/s12859-	http://bmcbioinformatics.biomedcentral.com/articles/10.1186/s12859-015-0585-1		2015-12	2021-10-12 2:39	2021-10-12 2:39	165	1	16	en
KH9DSFPV	journalArticle	2015	Richards, Stephe	It's more than stamp collecting: how genome sequer		1689525	10.1016/j.tig.201	https://linkinghub.elsevier.com/retrieve/pii/S0168952515000888		2015-07	2021-10-12 2:39	2021-10-12 2:39	411-421	7	31	en
XRYDCGVH	journalArticle	2015	Peterson, J, M; M	Teaching RNAseq at undergraduate institutions: a tutorial and R package from the Genome Consortium for Active Teaching						2015	2021-10-12 2:39	2021-10-12 2:39				
E6HQI5IB	journalArticle	2015	Minervini, Sotiris	Image Analysis: The New Bottleneck in Plant Pheno		1053-5888	10.1109/MSP.201	http://ieeexplore.ieee.org/document/7123050/		2015-07	2021-10-12 2:39	2021-10-12 2:39	126-131	4	32	
JZVLAQFT	conferencePaper	2015	Heiland, Von, Ra	Authentication and Authorization C	978-1-4503-3566-9		10.1145/2753524	http://dl.acm.org/citation.cfm?doid=2753524.2753534		2015	2021-10-12 2:39	2021-10-12 2:39	29-35			en
WF3JCV9E	journalArticle	2015	Lyu, Xin-Guang,	RNA-Seq based phylogeny recapitulates previous pl		1471-2148	10.1186/s12862-	http://www.biomedcentral.com/1471-2148/15/116		2015-12	2021-10-12 2:39	2021-10-12 2:39	116	1	15	en
AGUWSMKD	journalArticle	2015	Nwakanma, Mn,	and www.biotechsocietynigeria.org DOI: http://dx.do		1891731	10.4314/njb.v29i	http://www.ajol.info/index.php/njb/article/view/120853		2015-08-12	2021-10-12 2:39	2021-12-07 19:5	27	1	29	
DIGILWLW	journalArticle	2015	Campbell, Harka	Integrating Image-Based Phenomics and Associatio		0032-0889, 1532	10.1104/pp.15.00	http://www.plantphysiol.org/lookup/doi/10.1104/pp.15.00450		2015-08	2021-10-12 2:39	2021-10-12 2:39	1476-1489	4	168	en
3PIE2IDC	conferencePaper	2015	Marru, Chathuri,	Apache Airavata as a Laboratory:	978-1-4503-3566-9		10.1145/2753524	http://dl.acm.org/citation.cfm?doid=2753524.2753529		2015	2021-10-12 2:39	2021-10-12 2:39	19-26			en
IXKDQKYV	bookSection	2015	Sivasubramanian	Arabidopsis thaliana: A Model for F	978-81-322-2282-8 978-81-322-2283-5			http://link.springer.com/10.1007/978-81-322-2283-5_1		2015	2021-10-12 2:39	2021-10-12 2:39	26-Jan			en
6VIK9AKH	conferencePaper	2015	Jonquet, Pierre,	AgroPortal: a proposition for ontology-based services in the agronomic domain						2015	2021-10-12 2:39	2021-10-12 2:39				
6QRX3CIJ	journalArticle	2015	Zheng, Eric C., Z	Wild-Type N-Ras, Overexpressed in Basal-like Brea		22111247	10.1016/j.celrep.	https://linkinghub.elsevier.com/retrieve/pii/S2211124715006567		2015-07	2021-10-12 2:39	2021-10-12 2:39	511-524	3	12	en
WX6R275L	conferencePaper	2015	Lewis, John H, R	Accordion: multi-scale recipes for adaptive detection of duplication						2015	2021-10-12 2:39	2021-10-12 2:39				
BT7MBIMK	journalArticle	2015	Allahverdiyeva,	Integration of photosynthesis, development and stre		0028646X	10.1111/nph.135	http://doi.wiley.com/10.1111/nph.13549		2015-11	2021-10-12 2:39	2021-10-12 2:39	647-655	3	208	en
PNTF8RQ6	book	2015	Council, National	Enhancing the effectiveness of team science						2015	2021-10-12 2:39	2021-10-12 2:39				
ZCYN5TQ4	journalArticle	2015	Charnigo R, Thor	Parallel Computing in Genome-Wide Association Stu		21556180	10.4172/2155-61	https://www.omicsonline.org/open-access/parallel-computing-in-genomewide-association-studies-2155-6180-1000e131.php?aid=54		2015	2021-10-12 2:39	2021-10-12 2:39		1	6	
8F5M62BI	bookSection	2015	Horvath, Münevv	Leafy Spurge Genomics: A Model Perennial Weed to Investigate Development, Stress Responses, and Invasiveness						2015	2021-10-12 2:39	2021-10-12 2:39	63–78			
2LLL38FT	journalArticle	2015	Roux, Matthew B	Viral dark matter and virus–host interactions resolve		2050-084X	10.7554/eLife.08	https://elifescienc	The ecological importance of viruses is now widely recognized, yet our limited knowledge of viral sequence space a	2015-07-22	2021-10-12 2:39	2021-12-07 19:5	e08490		4	en
SWWKB9ZQ	conferencePaper	2015	Gesing, Alexande	Science gateways - leveraging mo	978-1-4673-7812-3 978-1-4673-78		10.1109/HPCSim	http://ieeexplore.ieee.org/document/7237017/		2015-07	2021-10-12 2:39	2021-10-12 2:39	19-26			
IGUFK5GH	journalArticle	2015	Krampis, Claudia	A Review of Cloud Computing Bioinformatics Solutic		2084-7173	10.1515/mngs-20	http://access.port	Abstract Research in biology has entered a digital era, where next-generation sequencing instruments generate mu	2015-07-27	2021-10-12 2:39	2021-12-07 19:5		1	2	
VDKSYFE7	thesis	2015	Spannagl, Manue	The genomic repertoire of complex and polyploid cereal genomes						2015	2021-10-12 2:39	2021-10-12 2:39				
GZ3A3A5X	conferencePaper	2015	Stewart, Steven,	Jetstream: a self-provisioned, scal	978-1-4503-3720-5		10.1145/279274	http://dl.acm.org/citation.cfm?doid=2792745.2792774		2015	2021-10-12 2:39	2021-10-12 2:39	8-Jan			en
V4E78UGL	journalArticle	2015	Gray, Kasey; oth	Protocol for the generation of a transcription factor open reading frame collection (TFome)						2015	2021-10-12 2:39	2021-10-12 2:39	e15	15	5	
SNUY6X85	bookSection	2015	Yu, Dorrie, Jing;	Role of Bioinformatic Tools and Da	978-0-89118-626-7			https://dl.sciencesocieties.org/publications/books/abstracts/agronomymonogra/agronmonogr57/agronmonogr57.2013.0043		2015	2021-10-12 2:39	2021-10-12 2:39				en
ZFHJIXRX	conferencePaper	2015	Goscinski, Philip,	Clouds for Masses - Toward Ubiqu	978-1-4673-7287-9		10.1109/CLOUD.	http://ieeexplore.ieee.org/document/7214178/		2015-06	2021-10-12 2:39	2021-10-12 2:39	1141-1148			
874P5GDZ	conferencePaper	2015	McGregor, J., A.;	A Cloud-Based Platform for Suppo	978-1-4673-7287-9		10.1109/CLOUD.	http://ieeexplore.ieee.org/document/7214172/		2015-06	2021-10-12 2:39	2021-10-12 2:39	1107-1110			
VCT85CJ4	journalArticle	2015	Das, Alexander, A	Digital imaging of root traits (DIRT): a high-throughp		1746-4811	10.1186/s13007-	http://www.plantmethods.com/content/11/1/51		2015-12	2021-10-12 2:39	2021-10-12 2:39	51	1	11	en
7UZXASVP	journalArticle	2015	Antoni, PA, Ange	A Bioinformatics Approach to Systemic Lupus Erythematosus and Autoimmunity						2015	2021-10-12 2:39	2021-12-03 18:5				
CDB22M6J	book	2015	Walley, Jonathan	Biotechnology and genomics: exploiting the potential of CWR						2015	2021-10-12 2:39	2021-10-12 2:39				
RV2PZSXQ	journalArticle	2015	Lapatas, Maria V	Data integration in biological research: an overview		2241-5793	10.1186/s40709-	http://www.jbiolres.com/content/22/1/9		2015-12	2021-10-12 2:39	2021-10-12 2:39	9	1	22	en
U7US42XX	journalArticle	2015	Lin, David J., Tia	Transcriptional analysis of phloem-associated cells		1471-2164	10.1186/s12864-	http://www.biomedcentral.com/1471-2164/16/665		2015-12	2021-10-12 2:39	2021-10-12 2:39	665	1	16	en

XZ2UJEWB	conferencePaper	2015	Chard, Ian, Ryan	Cost-Aware Cloud Provisioning	978-1-4673-9325-6		10.1109/eScienc	http://ieeexplore.ieee.org/document/7304284/		2015-08	2021-10-12 2:39	2021-10-12 2:39	136-144			
HNCNUQZ7	conferencePaper	2015	Huck, Kristin, Pat	A Community Contribution Framew	978-1-4673-9325-6		10.1109/eScienc	http://ieeexplore.ieee.org/document/7304338/		2015-08	2021-10-12 2:39	2021-10-12 2:39	535-541			
7L9FJMHS	journalArticle	2015	Wiens, John J.	Faster diversification on land than sea helps explain		1461023X	10.1111/ele.1250	http://doi.wiley.com/10.1111/ele.12503		2015-11	2021-10-12 2:39	2021-10-12 2:39	1234-1241	11	18	en
GAV9QXL7	report	2015	Mikheyev, Jigyas	Using experimental evolution and next-generation sequencing to teach bench and bioin				https://peerj.com	Advances in sequencing technology have exponentially increased data-generating capabilities, and data analysis h	2015-09-09	2021-10-12 2:39	2021-12-07 19:5				en
3FX9BHI8	journalArticle	2015	Haug-Baltzell, Er	Identification of dopamine receptors across the extai		1662-453X	10.3389/fnins.20	http://journal.frontiersin.org/Article/10.3389/fnins.2015.00361/abstract		2015-10-07	2021-10-12 2:39	2021-12-07 19:5			9	
9BBV4KD7	journalArticle	2015	da Silva, Miron, F	Online Task Resource Consumption Prediction for S		0129-6264, 1793	10.1142/S012962	https://www.world	Estimates of task runtime, disk space usage, and memory consumption, are commonly used by scheduling and res	2015-09	2021-10-12 2:39	2021-10-12 2:39	1541003	3	25	en
L7CMRVFV	journalArticle	2015	Flis, Andrew J., A	Defining the robust behaviour of the plant clock gene		2046-2441, 2046	10.1098/rsob.150	https://royalsocietypublishing.org/doi/10.1098/rsob.150042		2015-10	2021-10-12 2:39	2021-10-12 2:39	150042	10	5	en
LX78RCW2	journalArticle	2015	Michaletz, Brian .	Plant Thermoregulation: Energetics, Trait–Environm		1695347	10.1016/j.tree.20	https://linkinghub.elsevier.com/retrieve/pii/S0169534715002396		2015-12	2021-10-12 2:39	2021-10-12 2:39	714-724	12	30	en
FMW78XTZ	bookSection	2015	Villanueva-Rosal	Semantic Bridges for Biodiversity S	978-3-319-25009-0 978-3-319-25010-6			http://link.springer.com/10.1007/978-3-319-25010-6_20		2015	2021-10-12 2:39	2021-10-12 2:39	310-317		9367	
LNEFPYIZ	conferencePaper	2015	Etemadpour, Ang	Designing and evaluating scientific workflows for big data interactions						2015	2021-10-12 2:39	2021-10-12 2:39	1–8			
EE3V2TVI	journalArticle	2015	Thorp, A.N., K.R.	Proximal hyperspectral sensing and data analysis ap		1681699	10.1016/j.compa	https://linkinghub.elsevier.com/retrieve/pii/S016816991500280X		2015-10	2021-10-12 2:39	2021-10-12 2:39	225-236		118	en
GEZRWJQP	conferencePaper	2015	Jordan, Dan, Chr	Wrangler's user environment: A so	978-1-4799-9926-2		10.1109/BigData	http://ieeexplore.ieee.org/document/7364043/		2015-10	2021-10-12 2:39	2021-10-12 2:39	2479-2486			
GZR7DVVQ	journalArticle	2015	Ming, Qingyi, Ray	The pineapple genome and the evolution of CAM ph		1061-4036, 1546	10.1038/ng.3435	http://www.nature.com/articles/ng.3435		2015-12	2021-10-12 2:39	2021-10-12 2:39	1435-1442	12	47	en
3YMS5ICU	journalArticle	2015	Marques, Andrea	Holocentromeres in <i>Rhynchospora</i> are assoc		0027-8424, 1091	10.1073/pnas.15	http://www.pnas.org/lookup/doi/10.1073/pnas.1512255112		2015-11-03	2021-10-12 2:39	2021-12-07 19:5	13633-13638	44	112	en
Q7RREPPB	journalArticle	2015	Docherty, Rachel	Key Edaphic Properties Largely Explain Temporal ar		1932-6203	10.1371/journal.p	https://dx.plos.org/10.1371/journal.pone.0135352		2015-11-04	2021-10-12 2:39	2021-12-07 19:5	e0135352	11	10	en
ELDWQ8DK	journalArticle	2015	VanBuren, Todd	Single-molecule sequencing of the desiccation-toler		0028-0836, 1476	10.1038/nature15	http://www.nature.com/articles/nature15714		2015-11	2021-10-12 2:39	2021-10-12 2:39	508-511	7579	527	en
CYST7N54	conferencePaper	2015	Boyd, Catherine,	Finding function in the unknown	978-1-4673-6799-8		10.1109/BIBM.20	http://ieeexplore.ieee.org/document/7359834/		2015-11	2021-10-12 2:39	2021-10-12 2:39	1098-1099			
76DJTPZD	journalArticle	2015	Gesing, Ian, San	Genomics and Biological Big Data: Facing Current and Future Challenges around Data				http://arxiv.org/ab	Novel technologies in genomics allow creating data in exascale dimension with relatively minor effort of human and	2015-11-09	2021-10-12 2:39	2021-12-07 19:5				
8DCYSAHI	journalArticle	2015	Tang, Eric, Haiba	SynFind: compiling syntenic regions across any set of genomes on demand						2015	2021-10-12 2:39	2021-10-12 2:39	3286–3298	12	7	
DDG7ZGCQ	conferencePaper	2015	James, Mark, Do	Tales from the trenches: can user s	978-1-4503-4000-7		10.1145/2834996	http://dl.acm.org/citation.cfm?doid=2834996.2834998		2015	2021-10-12 2:39	2021-10-12 2:39	11-Jan			en
H2DYZGIG	journalArticle	2015	Dudhagara, Raje	Web Resources for Metagenomics Studies		16720229	10.1016/j.gpb.20	https://linkinghub.elsevier.com/retrieve/pii/S1672022915001424		2015-10	2021-10-12 2:39	2021-10-12 2:39	296-303	5	13	en
EZ35RIX8	journalArticle	2015	Gardiner, Anthon	A genome-wide survey of DNA methylation in hexap		1474-760X	10.1186/s13059-	https://genomebiology.biomedcentral.com/articles/10.1186/s13059-015-0838-3		2015-12	2021-10-12 2:39	2021-10-12 2:39	273	1	16	en
RAV4YUJT	journalArticle	2015	Reed, Zeresenay	Digital data collection in paleoanthropology: Digital D		10601538	10.1002/evan.21	http://doi.wiley.com/10.1002/evan.21466		2015-11	2021-10-12 2:39	2021-10-12 2:39	238-249	6	24	en
MWLCPIUU	journalArticle	2015	Thessen, Paula M	Emerging semantics to link phenotype and environm		2167-8359	10.7717/peerj.14	https://peerj.com	Understanding the interplay between environmental conditions and phenotypes is a fundamental goal of biology. U	2015-12-14	2021-10-12 2:39	2021-12-07 19:5	e1470		3	en
WWTFWWP7	report	2015	Wang, Doreen, L	Architecting a distributed bioinformatics platform with iRODS and iPlant Agave API				http://biorxiv.org/	Over the past few years, cloud-based platforms have been proposed to address storage, management, and compu	2015-12-15	2021-10-12 2:39	2021-12-07 19:5				en
R768YXY5	journalArticle	2015	Liu, Guangtang,	An ultra-high-density map as a community resource		1471-2164	10.1186/s12864-	http://www.biomedcentral.com/1471-2164/16/1078		2015-12	2021-10-12 2:39	2021-10-12 2:39	1078	1	16	en
RYTM4N4Q	journalArticle	2015	Morajkar, Smitha	A DNA Barcode for stenochlaena palustris: an edible medicinal fern						2015	2021-10-12 2:39	2021-10-12 2:39	124-131		32	
DNIXD7ZC	journalArticle	2015	Shanahan, Sean	Teaching Data Science and Cloud Computing in Low		23791764	10.4172/2379-17	http://www.esciencecentral.org/journals/teaching-data-science-and-cloud-computing-in-low-and-middle-incomecountries-2379-1764-		2015	2021-10-12 2:39	2021-10-12 2:39		3	3	
6IZG8KL8	conferencePaper	2015	Hart, Douglas, M	Resolved observations of geostationary satellites from the 6.5 m MMT						2015	2021-10-12 2:39	2021-10-12 2:39				
J58ICH25	journalArticle	2015	Alvi, Angela V, Za	Genomic and expression analysis of transition prote		2156-5562	10.1080/2156556	http://www.tandfonline.com/doi/full/10.1080/21565562.2016.1178518		2015-11-02	2021-10-12 2:51	2021-12-07 19:5	e1178518	3	5	en
FG3JUWB4	conferencePaper	2015	Orcutt, F, JA; Raj	Workflow-Oriented Cyberinfrastructure for Sensor Data Analytics						2015	2021-10-12 2:55	2021-10-12 2:55				
6EQIYIJP	journalArticle	2014	Minervini, Sotirio	Image-based plant phenotyping with incremental lea		15749541	10.1016/j.ecoinf.	https://linkinghub.elsevier.com/retrieve/pii/S1574954113000691		2014-09	2021-10-12 2:33	2021-10-12 2:33	35-48		23	en
TSBD2CNQ	journalArticle	2014	Van Gorp, Louis,	Guest editors' introduction to the first issue on Exper		1676423	10.1016/j.scico.2	https://linkinghub.elsevier.com/retrieve/pii/S0167642313003523		2014-06	2021-10-12 2:33	2021-10-12 2:33	4-Jan		85	en
SX42H72M	journalArticle	2014	Barton, Solon P.,	Extending alignments with k -mismatches and ℓ -gap		3043975	10.1016/j.tcs.201	https://linkinghub.elsevier.com/retrieve/pii/S0304397513004660		2014-03	2021-10-12 2:33	2021-10-12 2:33	80-88		525	en
TXPB7MF7	journalArticle	2014	Rhee, Marek, Se	Towards revealing the functions of all genes in plant		13601385	10.1016/j.tplants.	https://linkinghub.elsevier.com/retrieve/pii/S1360138513002343		2014-04	2021-10-12 2:33	2021-10-12 2:33	212-221	4	19	en
DXHDHJX9	journalArticle	2014	Numa, T., H.; Itoh	MEGANTE: A Web-Based System for Integrated Pla		0032-0781, 1471	10.1093/pcp/pct1	https://academic.oup.com/pcp/article-lookup/doi/10.1093/pcp/pct157		2014-01-01	2021-10-12 2:33	2021-12-07 19:3	e2-e2	1	55	en
ZBKD9QER	journalArticle	2014	Campbell, Mark,	MAKER-P: A Tool Kit for the Rapid Creation, Manag		0032-0889, 1532	10.1104/pp.113.2	http://www.plantphysiol.org/lookup/doi/10.1104/pp.113.230144		2014-02	2021-10-12 2:33	2021-10-12 2:33	513-524	2	164	en
43UGVVBH	journalArticle	2014	Gehring, Thomas	Plant genetics and interspecific competitive interacti		9621083	10.1111/mec.125	http://doi.wiley.com/10.1111/mec.12503		2014-03	2021-10-12 2:33	2021-10-12 2:33	1379-1391	6	23	en
XHVQGHMW	journalArticle	2014	LeDuc, William, F	Leveraging the national cyberinfrastructure for biome		1067-5027, 1527	10.1136/amiajnl-2	https://academic.oup.com/jamia/article-lookup/doi/10.1136/amiajnl-2013-002059		2014-03	2021-10-12 2:33	2021-10-12 2:33	195-199	2	21	en
WN5FWBBB	journalArticle	2014	Gorton, Ian	Cyberinfrastructures: Bridging the Divide between S		0018-9162	10.1109/MC.201	http://ieeexplore.ieee.org/document/6617631/		2014-08	2021-10-12 2:33	2021-10-12 22:4	48-55	8	47	
IU22A8RA	bookSection	2014	Campbell, Mark,	Genome Annotation and Curation	978-0-471-25095-1			http://doi.wiley.com/10.1002/0471250953.bi0411s48		2014-12-12	2021-10-12 2:35	2021-12-07 19:4	4.11.1-4.11.39			en
DQE6236I	journalArticle	2014	Mohan, Tanzima,	Batchsubmit: a high-volume batch submission syste		15320626	10.1002/cpe.323	http://doi.wiley.com/10.1002/cpe.3234		2014-09-10	2021-10-12 2:35	2021-12-07 19:4	2240-2252	13	26	en
WHRN9B6V	journalArticle	2014	Koesterke, Dan,	Discovery of biological networks using an optimized		15320626	10.1002/cpe.325	http://doi.wiley.com/10.1002/cpe.3252		2014-09-10	2021-10-12 2:35	2021-12-07 19:4	2178-2190	13	26	en
B4X9XB93	journalArticle	2014	Ningthoujam, Anu	NoSQL Data Model for Semi-automatic Integration c		9580344	10.1002/pca.252	http://doi.wiley.com/10.1002/pca.2520		2014-11	2021-10-12 2:35	2021-10-12 2:35	495-507	6	25	en
3XQE7UXU	bookSection	2014	Verborgh, Rik, Ru	Survey of Semantic Description of	978-1-4614-9298-6 978-1-4614-9299-3			http://link.springer.com/10.1007/978-1-4614-9299-3_5		2014	2021-10-12 2:35	2021-10-12 2:35	69-89			en
FLP4XY4C	book	2014	Chen, Victor C.M	Big Data	978-3-319-06244-0 978-3-319-06245-7			http://link.springer.com/10.1007/978-3-319-06245-7		2014	2021-10-12 2:35	2021-10-12 2:35				
IQR6QQK5	journalArticle	2014	Goodrich, Ruth E	Conducting a Microbiome Study		928674	10.1016/j.cell.20	https://linkinghub.elsevier.com/retrieve/pii/S0092867414008642		2014-07	2021-10-12 2:35	2021-10-12 2:35	250-262	2	158	en
HV4ICSLK	journalArticle	2014	Robinson, Rober	EarthEnv-DEM90: A nearly-global, void-free, multi-sc		9242716	10.1016/j.isprsjpr	https://linkinghub.elsevier.com/retrieve/pii/S0924271613002360		2014-01	2021-10-12 2:35	2021-10-12 2:35	57-67		87	en
S9UPAJUL	journalArticle	2014	Feltus, F. Alex	Systems genetics: A paradigm to improve discovery		1689452	10.1016/j.plantsc	https://linkinghub.elsevier.com/retrieve/pii/S0168945214000478		2014-06	2021-10-12 2:35	2021-10-12 2:35	45-48		223	en
7CHDDVP9	journalArticle	2014	Wilson, Walter, A	Systematic land cover bias in Collection 5 MODIS cl		344257	10.1016/j.rse.201	https://linkinghub.elsevier.com/retrieve/pii/S0034425713003933		2014-02	2021-10-12 2:35	2021-10-12 2:35	149-154		141	en
8V2LQUSG	journalArticle	2014	Ma, Xiangfeng, C	Machine learning for Big Data analytics in plants		13601385	10.1016/j.tplants.	https://linkinghub.elsevier.com/retrieve/pii/S1360138514002192		2014-12	2021-10-12 2:35	2021-10-12 2:35	798-808	12	19	en
YTRX5LJN	journalArticle	2014	Michaletz, Brian .	Convergence of terrestrial plant production across g		0028-0836, 1476	10.1038/nature13	http://www.nature.com/articles/nature13470		2014-08	2021-10-12 2:35	2021-10-12 2:35	39-43	7512	512	en
ATN52DN4	journalArticle	2014	Werner, E. Toby,	A single evolutionary innovation drives the deep evo		2041-1723	10.1038/ncomms	http://www.nature.com/articles/ncomms5087		2014-09	2021-10-12 2:35	2021-10-12 2:35	4087	1	5	en
YSU2EK64	journalArticle	2014	Nezhad, Amir Sa	Future of portable devices for plant pathogen diagno		1473-0197, 1473	10.1039/C4LC00	http://xlink.rsc.org	This paper reviews the current progress of on-spot detection of plant pathogens and predicts the future of portable	2014	2021-10-12 2:35	2021-10-12 2:35	2887-2904	16	14	en
P7R2TNUM	journalArticle	2014	Hurwitz, M. B., B	Modeling ecological drivers in marine viral communit		0027-8424, 1091	10.1073/pnas.13	http://www.pnas.org/cgi/doi/10.1073/pnas.1319778111		2014-07-22	2021-10-12 2:35	2021-12-07 19:4	10714-10719	29	111	en
BJB2JJVU	journalArticle	2014	Wickett, James, I	Phylotranscriptomic analysis of the origin and early c		0027-8424, 1091	10.1073/pnas.13	http://www.pnas.org/lookup/doi/10.1073/pnas.1323926111		2014-11-11	2021-10-12 2:35	2021-12-07 19:4	E4859-E4868	45	111	en
AMYRZKEJ	journalArticle	2014	Ichihashi, N. R., Y	Evolutionary developmental transcriptomics reveals		0027-8424, 1091	10.1073/pnas.14	http://www.pnas.org/cgi/doi/10.1073/pnas.1402835111		2014-06-24	2021-10-12 2:35	2021-12-07 19:4	E2616-E2621	25	111	en
TCLBEVRW	journalArticle	2014	Mirarab, T., S.; R	ASTRAL: genome-scale coalescent-based species t		1460-2059, 1367	10.1093/bioinfor	https://academic.oup.com/bioinformatics/article-lookup/doi/10.1093/bioinformatics/btu462		2014-09-01	2021-10-12 2:35	2021-12-07 19:4	i541-i548	17	30	en
G6NQTGB9	journalArticle	2014	Brunbjerg, J.-C.,	Multi-scale phylogenetic structure in coastal dune pl		1752-9921, 1752	10.1093/jpe/rtt06	https://academic.oup.com/jpe/article-lookup/doi/10.1093/jpe/rtt069		2014-04-01	2021-10-12 2:35	2021-12-07 19:4	101-114	2	7	en
LSL7QSX6	journalArticle	2014	Parry, Geraint	Components of the Arabidopsis nuclear pore comple		1460-2431, 0022	10.1093/jxb/eru3	https://academic.oup.com/jxb/article-lookup/doi/10.1093/jxb/eru346		2014-11	2021-10-12 2:35	2021-10-12 2:35	6057-6067	20	65	en
F9TN4DFL	journalArticle	2014	Stobbe, U., A. H.	Screening Metagenomic Data for Viruses Using the		0031-949X	10.1094/PHYTO-	http://apsjournals.apsnet.org/doi/10.1094/PHYTO-11-13-0310-R		2014-10	2021-10-12 2:35	2021-10-12 2:35	1125-1129	10	104	en
UQAH2B8R	journalArticle	2014	Aronson, Marten	A global analysis of the impacts of urbanization on b		0962-8452, 1471	10.1098/rspb.20	https://royalsocietypublishing.org/doi/10.1098/rspb.2013.3330		2014-04-07	2021-10-12 2:35	2021-12-07 19:4	20133330	1780	281	en
Q364FV4Y	report	2014	Wang, Lincoln, Li	A GWAS platform built on iPlant cyber-infrastructure				http://biorxiv.org/	We demonstrated a flexible Genome-Wide Association Study (GWAS) platform built upon the iPlant Collaborative (	2014-02-20	2021-10-12 2:35	2021-12-07 19:3				en
WZ28RZ42	report	2014	Schatz, William F	New whole genome de novo assemblies of three divergent strains of rice (O. sativa) doc				http://biorxiv.org/	The use of high throughput genome-sequencing technologies has uncovered a large extent of structural variation i	2014-04-02	2021-10-12 2:35	2021-12-07 19:4				en
9C87ITZF	journalArticle	2014	Ranjan, N. R., A.	De Novo Assembly and Characterization of the Tran		0032-0889, 1532	10.1104/pp.113.2	http://www.plantphysiol.org/cgi/doi/10.1104/pp.113.234864		2014-11-01	2021-10-12 2:35	2021-12-07 19:4	1186-1199	3	166	en
5F4FJADE	journalArticle	2014	Li, Han Hu; Yong	Toward Scalable Systems for Big Data Analytics: A T		2169-3536	10.1109/ACCESS	http://ieeexplore.ieee.org/document/6842585/		2014	2021-10-12 2:35	2021-10-12 2:35	652-687		2	
J7YGVS6A	conferencePaper	2014	Kanewala, Marlo	A Credential Store for Multi-tenant	978-1-4799-2784-5		10.1109/CCGrid.	http://ieeexplore.ieee.org/document/6846480/		2014-05	2021-10-12 2:35	2021-10-12 2:35	445-454			
WKA97BGE	conferencePaper	2014	Hanlon, Eleanor,	The Arabidopsis Information Portal	978-1-4799-7030-8		10.1109/GCE.20	http://ieeexplore.ieee.org/document/7021848/		2014-11	2021-10-12 2:35	2021-10-12 2:35	38-41			
J54BJQRS	conferencePaper	2014	Lawrence, Sures	Who Cares about Science Gatewa	978-1-4799-7030-8		10.1109/GCE.20	http://ieeexplore.ieee.org/document/7021839/		2014-11	2021-10-12 2:35	2021-10-12 2:35	4-Jan			
ULCGRHSY	conferencePaper	2014	Cholia, Terence,	The NEWT Platform: An Extensible	978-1-4799-7030-8		10.1109/GCE.20	http://ieeexplore.ieee.org/document/7021843/		2014-11	2021-10-12 2:35	2021-10-12 2:35	17-20			
QVGS8WVX	conferencePaper	2014	Cuccuru, Gianlui	An automated infrastructure to sup	978-1-4799-5313-4 978-1-4799-53		10.1109/HPCSim	http://ieeexplore.ieee.org/document/6903742/		2014-07	2021-10-12 2:35	2021-10-12 2:35	600-607			
XD6ZNVUG	journalArticle	2014	Holmgren, Jerem	Evidence against a Pleistocene desert refugium in th		3050270	10.1111/jbi.12337	http://doi.wiley.com/10.1111/jbi.12337		2014-09	2021-10-12 2:35	2021-10-12 2:35	1769-1780	9	41	en
AD5WHPLT	journalArticle	2014	Huang, Elizabeth	Population genetics of <i>Setaria viridis</i> , a new		9621083	10.1111/mec.129	http://doi.wiley.com/10.1111/mec.12907		2014-10	2021-10-12 2:35	2021-10-12 2:35	4912-4925	20	23	en
7NSADBIF	journalArticle	2014	Wilson-Sánchez,	Leaf phenomics: a systematic reverse genetic scree		9607412	10.1111/tpj.1259	http://doi.wiley.com/10.1111/tpj.12595		2014-09	2021-10-12 2:35	2021-10-12 2:35	878-891	5	79	en
BRH3VMZC	journalArticle	2014	Gardiner, Anthon	Using genic sequence capture in combination with a		9607412	10.1111/tpj.1266	http://doi.wiley.com/10.1111/tpj.12660		2014-12	2021-10-12 2:35	2021-10-12 2:35	895-904	5	80	en
HS552HMD	journalArticle	2014	Kim, J. H., G.; Le	Genomic-scale exchange of mRNA between a paras		0036-8075, 1095	10.1126/science.	http://www.sciencemag.org/cgi/doi/10.1126/science.1253122		2014-08-15	2021-10-12 2:35	2021-12-07 19:4	808-811	6198	345	en
XA8ZZQLS	journalArticle	2014	Chalhoub, P., B.;	Early allopolyploid evolution in the post-Neolithic Bra		0036-8075, 1095	10.1126/science.	http://www.sciencemag.org/cgi/doi/10.1126/science.1253435		2014-08-22	2021-10-12 2:35	2021-12-07 19:4	950-953	6199	345	en
SMET42TT	conferencePaper	2014	Xu, Reagan, Hac	Building an extensible file system v	978-1-4503-2912-5		10.1145/2603941	http://dl.acm.org/citation.cfm?doid=2603941.2603943		2014	2021-10-12 2:35	2021-10-12 2:35	18-Nov			en
AYF9LN9I	conferencePaper	2014	Dooley, Joe, Rior	Dynamically Provisioning Portable	978-1-4503-2893-7		10.1145/2616498	http://dl.acm.org/citation.cfm?doid=2616498.2616561		2014	2021-10-12 2:35	2021-10-12 2:35	2-Jan			en
597FITCC	conferencePaper	2014	Hart, Matthew, D	XRAS: Allocations software as a se	978-1-4503-2893-7		10.1145/2616498	http://dl.acm.org/citation.cfm?doid=2616498.2616562		2014	2021-10-12 2:35	2021-10-12 2:35	8-Jan			en
IIPF9GN8	conferencePaper	2014	Hilgert, David, Uv	DNA Subway: Making Genome An	978-1-4503-2893-7		10.1145/2616498	http://dl.acm.org/citation.cfm?doid=2616498.2616575		2014	2021-10-12 2:35	2021-10-12 2:35	3-Jan			en
ZEXDJMU5	conferencePaper	2014	Gnimpieba, Caro	RNA-seq gene and transcript expr	978-1-4503-2894-4		10.1145/2649387	http://dl.acm.org/citation.cfm?doid=2649387.2660822		2014	2021-10-12 2:35	2021-10-12 2:35	661-669			en
3LTESYX7	conferencePaper	2014	Thavappiragasar	Heuristic parallelizable algorithm fc	978-1-4503-2894-4		10.1145/2649387	http://dl.acm.org/citation.cfm?doid=2649387.2660849		2014	2021-10-12 2:35	2021-10-12 2:35	782-789			en
CHX4SWQW	conferencePaper	2014	Weinandt, Carol,	Pheno2GRN: a workflow for phenc	978-1-4503-2894-4		10.1145/2649387	http://dl.acm.org/citation.cfm?doid=2649387.2660851		2014	2021-10-12 2:35	2021-10-12 2:35	797-804			en
DXP8VABB	journalArticle	2014	Merelli, Daniele,	High-Performance Computing and Big Data in Omic		2314-6133, 2314	10.1155/2014/82	http://www.hindawi.com/journals/bmri/2014/825649/		2014	2021-10-12 2:35	2021-10-12 2:35	2-Jan		2014	en
FTG9GPTH	journalArticle	2014	Lammers, Barba	The HTS barcode checker pipeline, a tool for automa		1471-2105	10.1186/1471-21	http://bmcbioinformatics.biomedcentral.com/articles/10.1186/1471-2105-15-44		2014	2021-10-12 2:35	2021-10-12 2:35	44	1	15	en
7S38R8LV	journalArticle	2014	Ruhfel, J, Brad R	From algae to angiosperms–inferring the phylogeny		1471-2148	10.1186/1471-21	http://bmcevolbiol.biomedcentral.com/articles/10.1186/1471-2148-14-23		2014	2021-10-12 2:35	2021-10-12 2:35	23	1	14	en
BVNSXFMH	journalArticle	2014	Melicher, Julia H,	A pipeline for the de novo assembly of the Themira b		1471-2164	10.1186/1471-21	http://bmcgenomics.biomedcentral.com/articles/10.1186/1471-2164-15-188		2014	2021-10-12 2:35	2021-10-12 2:35	188	1	15	en
WSJ7A3MV	journalArticle	2014	Belamkar, Steve	Comprehensive characterization and RNA-Seq profi		1471-2164	10.1186/1471-21	http://bmcgenomics.biomedcentral.com/articles/10.1186/1471-2164-15-950		2014	2021-10-12 2:35	2021-10-12 2:35	950	1	15	en
K7P9VENC	journalArticle	2014	Zhao, Laura E, K	Comparative genomic analysis of the R2R3 MYB se		1471-2229	10.1186/1471-22	http://bmcplantbiol.biomedcentral.com/articles/10.1186/1471-2229-14-135		2014	2021-10-12 2:35	2021-10-12 2:35	135	1	14	en
FW5L5IY9	journalArticle	2014	Das, Joshua S, A	ClearedLeavesDB: an online database of cleared pla		1746-4811	10.1186/1746-48	http://plantmethods.biomedcentral.com/articles/10.1186/1746-4811-10-8		2014	2021-10-12 2:35	2021-10-12 2:35	8	1	10	en
XCRKM8L2	journalArticle	2014	Walls, Jie, Ramo	Meeting report: advancing practical applications of b		1944-3277	10.1186/1944-32	https://standardsingenomics.biomedcentral.com/articles/10.1186/1944-3277-9-17		2014-12	2021-10-12 2:35	2021-10-12 2:35	17	1	9	en
4CHYE3WL	journalArticle	2014	Dahdul, Melissa	Nose to tail, roots to shoots: spatial descriptors for p		2041-1480	10.1186/2041-14	http://jbiomedsem.biomedcentral.com/articles/10.1186/2041-1480-5-34		2014	2021-10-12 2:35	2021-10-12 2:35	34	1	5	en
IZB2UNSQ	journalArticle	2014	Matasci, Gane K	Data access for the 1,000 Plants (1KP) project		2047-217X	10.1186/2047-21	https://academic.oup.com/gigascience/article-lookup/doi/10.1186/2047-217X-3-17		2014-12	2021-10-12 2:35	2021-10-12 2:35	17	1	3	en
8VB5ST9R	journalArticle	2014	Shaffer, Sarah C.	A Course-Based Research Experience: How Benefit		1931-7913	10.1187/cbe-13-0	https://www.lifesc	There is widespread agreement that science, technology, engineering, and mathematics programs should provide	2014-03	2021-10-12 2:35	2021-10-12 2:35	111-130	1	13	en
MY3HIHTW	journalArticle	2014	Amrine, David H.	tRNA Signatures Reveal a Polyphyletic Origin of SAI		1553-7358	10.1371/journal.p	https://dx.plos.org/10.1371/journal.pcbi.1003454		2014-02-27	2021-10-12 2:35	2021-12-07 19:3	e1003454	2	10	en
SGYMZ7FR	journalArticle	2014	Walls, John, Ram	Semantics in Support of Biodiversity Knowledge Dis		1932-6203	10.1371/journal.p	https://dx.plos.org/10.1371/journal.pone.0089606		2014-03-03	2021-10-12 2:35	2021-12-07 19:3	e89606	3	9	en
2K567Z7H	journalArticle	2014	West, Nathan M.	Genomic Distribution of H3K9me2 and DNA Methyla		1932-6203	10.1371/journal.p	http://dx.plos.org/10.1371/journal.pone.0105267		2014-08-14	2021-10-12 2:35	2021-12-07 19:4	e105267	8	9	en
V2343FJC	journalArticle	2014	Staff, The PLOS	Correction: De Novo Transcriptome Assembly and A		1932-6203	10.1371/journal.p	https://dx.plos.org/10.1371/journal.pone.0105275		2014-08-01	2021-10-12 2:35	2021-12-07 19:4	e105275	8	9	en
6H2MLNAK	journalArticle	2014	Rees, Tony	Taxamatch, an Algorithm for Near ('Fuzzy') Matching		1932-6203	10.1371/journal.p	http://dx.plos.org/10.1371/journal.pone.0107510		2014-09-23	2021-10-12 2:35	2021-12-07 19:4	e107510	9	9	en
63EFDVU2	journalArticle	2014	Soderlund, Steph	Allele Workbench: Transcriptome Pipeline and Intera		1932-6203	10.1371/journal.p	https://dx.plos.org/10.1371/journal.pone.0115740		2014-12-26	2021-10-12 2:35	2021-12-07 19:4	e115740	12	9	en
SLYFC3VM	journalArticle	2014	Wang, Peiya, Fu	Metadata based management and sharing of distribu		1744-2621, 1744	10.1504/IJMSO.2	http://www.inderscience.com/link.php?id=59126		2014	2021-10-12 2:35	2021-10-12 2:35	42	1	9	en
EBG8PY7L	journalArticle	2014	Jamann, Rebecc	Unraveling Genomic Complexity at a Quantitative Di		0016-6731, 1943	10.1534/genetics	http://www.genetics.org/lookup/doi/10.1534/genetics.114.167486		2014-09	2021-10-12 2:35	2021-10-12 2:35	333-344	1	198	en
I4N3E997	journalArticle	2014	Esa	The 2014 Annual Reports to the Governing Council:		0012-9623	10.1890/2014.co	http://doi.wiley.com/10.1890/2014.councilreport		2014-10	2021-10-12 2:35	2021-10-12 2:35	S1-S69	4	95	en
TZH9PRF3	journalArticle	2014	Uribe-Convers, D	A Long PCR–Based Approach for DNA Enrichment F		2168-0450	10.3732/apps.13	http://doi.wiley.com/10.3732/apps.1300063		2014-01	2021-10-12 2:35	2021-10-12 2:35	1300063	1	2	en
4LQQWVD2	journalArticle	2014	Wofford, Ann, Au	A Set of Plastid Loci for Use in Multiplex Fragment L		2168-0450	10.3732/apps.14	http://doi.wiley.com/10.3732/apps.1400002		2014-05	2021-10-12 2:35	2021-10-12 2:35	1400002	5	2	en

KDU9VK26	journalArticle	2014	Smith, Tessa L, H	Using Flatbed Scanners to Collect High-resolution T	1940-087X	10.3791/50878	http://www.jove.com/video/50878/using-flatbed-scanners-to-collect-high-resolution-time-lapsed-images		2014-01-25	2021-10-12 2:35:	2021-12-07 19:3		50878		83		en
KKWFZ9A2	journalArticle	2014	Poelchau, Peter,	An Experimental and Bioinformatics Protocol for RN	1940-087X	10.3791/51961	http://www.jove.com/video/51961/an-experimental-bioinformatics-protocol-for-rna-seq-analyses		2014-11-30	2021-10-12 2:35:	2021-12-07 19:4		51961		93		en
U6IKYRTV	journalArticle	2014	Parr, Robert, Cyr	The Encyclopedia of Life v2: Providing Global Acces	1314-2828, 1314	10.3897/BDJ.2.e	http://bdj.pensoft.net/articles.php?id=1079		2014-04-29	2021-10-12 2:36:	2021-12-07 19:4		e1079			2	
EDFLE6JY	journalArticle	2014	Tukamuhabwa, L	Application of Bioinformatics in Crop Improvement: A	0974276X	10.4172/jpb.100	http://omicsonline.org/application-of-bioinformatics-in-crop-improvement-annotating-the-putative-soybean-rust-resistance-gene-rpp-f		2014	2021-10-12 2:36:	2021-10-12 2:36:				1	7	
6FAGEMDB	journalArticle	2014	Rajasekar, Reag	Note on Three Classes of Data Grid Operations	2327-5219, 2327	10.4236/jcc.2014	http://www.scirp.org/journal/doi.aspx?DOI=10.4236/jcc.2014.26001		2014	2021-10-12 2:36:	2021-10-12 2:36:		6-Jan		6	2	
AFJLIUB7	journalArticle	2014	Yuvaraja, C, R; R	DESIGN AND DEVELOPMENT OF SUBJECT GATEWAY WITH SPECIAL REFERENCE TO CHEMISTRY					2014	2021-10-12 2:36:	2021-10-12 2:36:						
2EPWUDVP	journalArticle	2014	Oaks, Jamie R.	An Improved Approximate-Bayesian Model-choice M	1471-2148	10.1186/1471-21	http://arxiv.org/ab	To understand biological diversification, it is important to account for large-scale processes that affect the evolution	2014	2021-10-12 2:36:	2021-10-12 2:36:		150		1	14	
ZKVXKLT3	journalArticle	2014	Oaks, Jeet, Jami	Implications of uniformly distributed, empirically infor	143820	10.1111/evo.1252	http://arxiv.org/ab	Establishing that a set of population-splitting events occurred at the same time can be a potentially persuasive argu	2014-12	2021-10-12 2:36:	2021-10-12 2:36:		3607-3617		12	68	
A5FZYZGG	journalArticle	2014	Wang, Doreen, L	The relationships among GC content, nucleosome occupancy, and exon size			http://arxiv.org/ab	The average size of internal translated exons, ranging from 120 to 165 nt across metazoans, is approximately the s	2014-05-27	2021-10-12 2:36:	2021-12-07 19:4						
6BJGXDHE	journalArticle	2014	Katz, Colin, Dani	Summary of the First Workshop on Sustainable Soft	2049-9647	10.5334/jors.an	http://arxiv.org/ab	Challenges related to development, deployment, and maintenance of reusable software for science are becoming a	2014-07-09	2021-10-12 2:36:	2021-12-07 19:4		e6		1	2	
8RQZBQWV	journalArticle	2014	Makinde, Diran	African Orphan Crops Consortium: a NEPAD-led initiative					2014	2021-10-12 2:36:	2021-10-12 2:36:		57				
BBL9FPDB	journalArticle	2014	Izquierdo-Carras	PUmPER: phylogenies updated perpetually					2014	2021-10-12 2:36:	2021-10-12 2:36:		1476–1477		10	30	
XIQT2ST8	conferencePaper	2014	Tong, Xiu, Qiuhui	Cyberinfrastructure: Applications and challenges					2014	2021-10-12 2:36:	2021-10-12 2:36:		74–80				
KAQT9AUK	conferencePaper	2014	Walls, Ramona; J	A Specimen-based View of the World: Using the Biological Collections Ontology to Mod			http://ceur-ws.org/Vol-1309/paper8.pdf		2014	2021-10-12 2:36:	2021-12-03 18:5						
DQ7T2LZS	journalArticle	2014	Merelli, Daniele,	Managing, analysing, and integrating big data in medical bioinformatics: open problems and future perspectives					2014	2021-10-12 2:36:	2021-10-12 2:36:					2014	
ZY9792WY	journalArticle	2014	Mentewab, Brian	RNA-seq analysis of the effect of kanamycin and the ABC transporter AtWBC19 on Arabidopsis thaliana seedlings reveals changes in metal content					2014	2021-10-12 2:36:	2021-10-12 2:36:		e109310		10	9	
Y9V485JG	journalArticle	2014	Stewart, Craig A	Serving national scientific communities: Genome analysis as an example					2014	2021-10-12 2:36:	2021-10-12 2:36:						
N2XPKSRH	conferencePaper	2014	Rajasekar, Arcot.	The Librarian & the Big Data: Bridging the Gap					2014	2021-10-12 2:36:	2021-12-03 18:5						
CC65YWWW	journalArticle	2014	Brauer, Sorina C.	Next-generation plant science: putting big data to wo	1474-760X	10.1186/gb4149	https://doi.org/10	A Report on the Plant Genomes & Biotechnology: From Genes to Networks meeting, held at the Cold Spring Harbo	2014-01-15	2021-10-12 2:36:	2021-12-07 19:3		301		1	15	
EP4X5CCF	journalArticle	2014	Snoderly-Foster,	Characterization of a plant gene family expanded in glycine max					2014	2021-10-12 2:36:	2021-10-12 2:36:						
Y5IRJCFM	thesis	2014	Scott, Taylor	Identification of GCN4 homologues in maize using a bioinformatics approach					2014	2021-10-12 2:36:	2021-10-12 2:36:						
A6JUTTGS	journalArticle	2014	Mirarab, Tandy, S	Binning Code for Mirarab et. al.					2014	2021-10-12 2:36:	2021-10-12 2:36:						
JJDSEV9T	journalArticle	2014	Mirarab, Tandy, S	Avian Model Species Trees for 1X Model Condition for Mirarab et. al.					2014	2021-10-12 2:36:	2021-10-12 2:36:						
6W65GSAB	journalArticle	2014	Mirarab, Tandy, S	Sequence Alignments and Trees for Avian 1X for Mirarab et. al.					2014	2021-10-12 2:36:	2021-10-12 2:36:						
PDCLE5WI	journalArticle	2014	Mirarab, Tandy, S	Bin Definition for Super Gene Trees for Mirarab et. al.					2014	2021-10-12 2:36:	2021-10-12 2:36:						
6EKABFXE	journalArticle	2014	Sachdeva, Ben, I	Report of the final external review of the Generation Challenge Programme					2014	2021-10-12 2:36:	2021-10-12 2:36:						
S45DXPBH	journalArticle	2014	Chen, Yunhao, M	Big data: A survey					2014	2021-10-12 2:36:	2021-10-12 2:36:		171–209		2	19	
6SPKAER7	journalArticle	2014	Caciula, Adrian	Optimization techniques for next-generation sequencing data analysis					2014	2021-10-12 2:36:	2021-10-12 2:36:						
66P2WCJW	conferencePaper	2014	Thavappiragasar	SBMLChecker, a Semantic approach for SBML model reliability evaluation					2014	2021-10-12 2:36:	2021-10-12 2:36:		1				
5CZXCN65	conferencePaper	2014	Mirarab, Tandy, S	PASTA: ultra-large multiple sequence alignment					2014	2021-10-12 2:36:	2021-10-12 2:36:		177–191				
48PALVEM	journalArticle	2014	Maia, Douglas E,	Angiosperm phylogeny based on 18S/26S rDNA sequence data: constructing a large data set using next-generation sequence data					2014	2021-10-12 2:36:	2021-10-12 2:36:		613–650		6	175	
JGZ7CBE9	journalArticle	2014	Carnaby, S, Penr	Lincoln Hub data and information architecture project: DATA^2: Data architecture transforming access & analysis					2014	2021-10-12 2:36:	2021-10-12 2:36:						
BHF6PVNU	journalArticle	2014	Aronson, Bruce;	A global analysis of the impacts of urbanization on bird and plant diversity reveals key anthropogenic drivers					2014	2021-10-12 2:36:	2021-10-12 2:36:		20133330		1780	281	
2UA73UU6	conferencePaper	2014	Williams, D, J; M	DNA Subway–An educational bioinformatics platform for gene and genome analysis: DNA barcoding, and RNA-Seq					2014	2021-10-12 2:36:	2021-10-12 2:36:						
4K47DW22	conferencePaper	2014	Reecy, James; C	Computational resources to facilitate variant discovery and analysis					2014-03-18	2021-10-12 2:36:	2021-12-07 19:3					23	
J64WT9Y4	conferencePaper	2014	Der, Joshua P.	A unified global gene family classification resource for plants and its utility for comparative genomics, genome annotation, and gene family studies					2014-01-14	2021-10-12 2:36:	2021-12-07 19:3						
9UG6VYS7	conferencePaper	2014	Duvick, Jon	Genome annotation in the cloud through xGDBvm virtual server instances deployed at iPlant					2014-01-14	2021-10-12 2:36:	2021-12-07 19:3						
VE8YJEKM	conferencePaper	2014	Hurwitz, Bonnie	iMicrobe: Advancing Clinical and Environmental Microbial Research using the iPlant Cyberinfrastructure					2014-01-14	2021-10-12 2:36:	2021-12-07 19:3						
AFUBLND3	conferencePaper	2014	Buckner, Brent	Introducing Genome Browsers, Genome Structure, and Gene Annotation					2014-01-11	2021-10-12 2:36:	2021-12-07 19:3						
PW6UHCS3	conferencePaper	2014	Vaughn, Matt	The Arabidopsis Information Portal: A First Glimpse					2014-01-13	2021-10-12 2:36:	2021-12-07 19:3						
42JQUAMG	conferencePaper	2014	Micklos, Dave	A Simple Bioinformatics Workflow for RNA-Seq Analysis and Distributed Genome Annotation					2014-01-11	2021-10-12 2:36:	2021-12-07 19:3						
8AYDPSY6	conferencePaper	2014	Vasquez-Gross, I	Case Study: Enabling Forest Tree Genomics through Association Studies with the use of SSWAP					2014-01-14	2021-10-12 2:36:	2021-12-07 19:3						
FF9QLSH6	conferencePaper	2014	Horvath, David; J	Progress in Sequencing the Genome of an Invasive Polyploid Weed (Leafy Spurge)					2014-01-14	2021-10-12 2:36:	2021-12-07 19:3						
JMWE3AFN	conferencePaper	2014	Mandadi, Kranthi	Transcriptomic Analyses and Alternative Splicing Landscapes of Brachypodium Infected with Panicum Mosaic Virus					2014-01-14	2021-10-12 2:36:	2021-12-07 19:3						
9EX5NNLK	conferencePaper	2014	Xu, Dong	Soybean knowledge base (SoyKB): a web resource for integration of soybean translational genomics and molecular breeding					2014-01-14	2021-10-12 2:36:	2021-12-07 19:3						
X5C2LS25	conferencePaper	2014	Reecy, J.; J.P. Ca	Cyberinfrastructure for Life Sciences - iAnimal resources for genomics and other data driven biology					2014-08-22	2021-10-12 2:36:	2021-12-07 19:4					9911	
Z8FUB3LH	report	2014	Hanssen, A.M., F	Nordic LifeWatch Cooperation. Final report.				The main goal of the present report is to outline the possibilities for an enhanced cooperation between the Nordic c	2014	2021-10-12 2:38:	2021-10-12 2:38:						
JTUC794X	journalArticle	2014	Jazayeri, Hernán	RNA-SEQ: A GLANCE AT TECHNOLOGIES AND M	1900-1649, 0120	10.15446/abc.v2	http://www.revistas.unal.edu.co/index.php/actabiol/article/view/43639		2014-11-04	2021-10-12 2:38:	2021-12-07 19:5				2	20	
MLASDBKU	journalArticle	2014	Towns, Nancy, Jo	XSEDE: Accelerating Scientific Discovery	1521-9615, 1558	10.1109/MCSE.2	https://ieeexplore.ieee.org/document/6866038/		2014-09	2021-10-12 2:39:	2021-10-12 2:39:		62-74		5	16	
2SYZQPMI	journalArticle	2013	Frousios, Stilianc	Transcriptome map of mouse isochores in embryoni	8887543	10.1016/j.ygeno.	https://linkinghub.elsevier.com/retrieve/pii/S0888754312002261		2013-02	2021-10-12 2:30:	2021-10-12 2:30:		120-124		2	101	en
UCL63749	journalArticle	2013	Lisch, Damon	How important are transposons for plant evolution?	1471-0056, 1471	10.1038/nrg3374	http://www.nature.com/articles/nrg3374		2013-01	2021-10-12 2:30:	2021-10-12 2:30:		49-61		1	14	en
WHCULF7H	journalArticle	2013	Oliver, Sheldon J	Using the iPlant Collaborative Discovery Environmen	19343396	10.1002/047125	http://doi.wiley.com/10.1002/0471250953.bi0122s42		2013-06	2021-10-12 2:33:	2021-10-12 2:33:		1.22.1-1.22.26		1	42	en
SB4F9PW2	journalArticle	2013	Matasci, Sheldon	Phylogenetic Analysis with the iPlant Discovery Envi	19343396	10.1002/047125	http://doi.wiley.com/10.1002/0471250953.bi0613s42		2013-06	2021-10-12 2:33:	2021-10-12 2:33:		6.13.1-6.13.13		1	42	en
TVN4FE2R	bookSection	2013	McKay, Christos,	Cloud Computing with iPlant Atmo:	978-0-471-25095-1		http://doi.wiley.com/10.1002/0471250953.bi0915s43		2013-10-15	2021-10-12 2:33:	2021-12-07 19:3		9.15.1-9.15.20				en
68NY4ZMZ	journalArticle	2013	Koehbach, Christ	Cyclotide discovery in Gentianales revisited-identific	63525	10.1002/bip.2232	http://doi.wiley.com/10.1002/bip.22328		2013-09	2021-10-12 2:33:	2021-10-12 2:33:		438-452		5	100	en
EEPQR9A9	journalArticle	2013	Kluyver, Colin P.,	Taxonome: a software package for linking biological	20457758	10.1002/ece3.52	http://doi.wiley.com/10.1002/ece3.529		2013-05	2021-10-12 2:33:	2021-10-12 2:33:		1262-1265		5	3	en
MSDLE5ST	bookSection	2013	Soltis, Douglas E	Angiosperm Phylogeny: A Framew	978-3-7091-1159-8 978-3-7091-1160-4		http://www.springerlink.com/index/10.1007/978-3-7091-1160-4_1		2013	2021-10-12 2:33:	2021-10-12 2:33:		11-Jan				en
8Y6NC38L	journalArticle	2013	López, J.F., R.; S	an e-Science project in Astrodynamics and Celestial	104655	10.1016/j.cpc.20	https://linkinghub.elsevier.com/retrieve/pii/S0010465513000088		2013-05	2021-10-12 2:33:	2021-10-12 2:33:		1381-1386		5	184	en
ETLTVRHV	journalArticle	2013	Christou, Jan, Mi	Tree template matching in unranked ordered trees	15708667	10.1016/j.jda.201	https://linkinghub.elsevier.com/retrieve/pii/S1570866713000026		2013-05	2021-10-12 2:33:	2021-10-12 2:33:		51-60			20	en
GK4IRIE3	journalArticle	2013	Goff, Qifa, Steph	Heterosis in elite hybrid rice: speculation on the gen	13695266	10.1016/j.pbi.201	https://linkinghub.elsevier.com/retrieve/pii/S1369526613000447		2013-05	2021-10-12 2:33:	2021-10-12 2:33:		221-227		2	16	en
5UIW695E	journalArticle	2013	Ragupathy, Sylvi	Arguments for standardizing transposable element a	13601385	10.1016/j.tplants.	https://linkinghub.elsevier.com/retrieve/pii/S1360138513000587		2013-07	2021-10-12 2:33:	2021-10-12 2:33:		367-376		7	18	en
Y6W2SJVA	journalArticle	2013	Dhondt, Dirk, Stij	Cell to whole-plant phenotyping: the best is yet to co	13601385	10.1016/j.tplants.	https://linkinghub.elsevier.com/retrieve/pii/S1360138513000812		2013-08	2021-10-12 2:33:	2021-10-12 2:33:		428-439		8	18	en
NT5298AN	journalArticle	2013	Thibault, Thomas	iBIOMES: Managing and Sharing Biomolecular Simu	1549-9596, 1549	10.1021/ci30052	https://pubs.acs.org/doi/10.1021/ci300524j		2013-03-25	2021-10-12 2:33:	2021-12-07 19:3		726-736		3	53	en
3GS6B9BH	journalArticle	2013	Ibarra-Laclette, L	Architecture and evolution of a minute plant genome	0028-0836, 1476	10.1038/nature1	http://www.nature.com/articles/nature12132		2013-06	2021-10-12 2:33:	2021-10-12 2:33:		94-98		7452	498	en
JJZYCA7H	journalArticle	2013	Waters, N. M., A.	Comprehensive analysis of imprinted genes in maiz	0027-8424, 1091	10.1073/pnas.13	http://www.pnas.org/cgi/doi/10.1073/pnas.1309182110		2013-11-26	2021-10-12 2:33:	2021-12-07 19:3		19639-19644		48	110	en
9FD3NMUD	journalArticle	2013	Koenig, J. N., D.;	Comparative transcriptomics reveals patterns of sele	0027-8424, 1091	10.1073/pnas.13	http://www.pnas.org/cgi/doi/10.1073/pnas.1309606110		2013-07-09	2021-10-12 2:33:	2021-12-07 19:3		E2655-E2662		28	110	en
ATGL9U4R	journalArticle	2013	Bayzid, Tandy, M	Naive binning improves phylogenomic analyses	1460-2059, 1367	10.1093/bioinfor	https://academic.oup.com/bioinformatics/article-lookup/doi/10.1093/bioinformatics/btt394		2013-09-15	2021-10-12 2:33:	2021-12-07 19:3		2277-2284		18	29	en
99F3QXRC	journalArticle	2013	Hofberger, M., Jo	Whole Genome and Tandem Duplicate Retention Fa	1759-6653	10.1093/gbe/evt1	https://academic.oup.com/gbe/article-lookup/doi/10.1093/gbe/evt162		2013-11	2021-10-12 2:33:	2021-10-12 2:33:		2155-2173		11	5	en
RIZW46CZ	journalArticle	2013	Cheung, Wei-Cai	Pollen and Plant Reproduction Biology: Blooming fro	16742052	10.1093/mp/sst1	https://linkinghub.elsevier.com/retrieve/pii/S1674205214608972		2013-07	2021-10-12 2:33:	2021-10-12 2:33:		995-997		4	6	en
IXIPUJZL	journalArticle	2013	Beaulieu, Michae	Identifying Hidden Rate Changes in the Evolution of	1076-836X, 1063	10.1093/sysbio/s	https://academic.oup.com/sysbio/article/62/5/725/1685276		2013-09-01	2021-10-12 2:33:	2021-12-07 19:3		725-737		5	62	en
4XUJMLSU	journalArticle	2013	Chen, Xiangfeng	CrusView: A Java-Based Visualization Platform for C	0032-0889, 1532	10.1104/pp.113.2	http://www.plantphysiol.org/lookup/doi/10.1104/pp.113.219444		2013-09	2021-10-12 2:33:	2021-10-12 2:33:		354-362		1	163	en
TBIXPNQP	conferencePaper	2013	Joshi, Dong, Tru	Soybean knowledge base (SoyKB	978-1-4799-1309-1	10.1109/BIBM.20	http://ieeexplore.ieee.org/document/6732755/		2013-12	2021-10-12 2:33:	2021-10-12 2:33:		42-44				
WMHZXEW3	conferencePaper	2013	Fick, B.S., R.H.J.	Simultaneous cell tracking and ima	978-1-4673-6455-3 978-1-4673-64	10.1109/ISBI.201	http://ieeexplore.ieee.org/document/6556624/		2013-04	2021-10-12 2:33:	2021-10-12 2:33:		914-917				
69G64BNV	journalArticle	2013	Tan, Schahram, \	Social-Network-Sourced Big Data Analytics	1089-7801	10.1109/MIC.201	http://ieeexplore.ieee.org/document/6596496/		2013-09	2021-10-12 2:33:	2021-10-12 2:33:		62-69		5	17	
XRKZSYFK	journalArticle	2013	Vasquez-Gross, J	CartograTree: connecting tree genomes, phenotype:	1755098X	10.1111/1755-09	http://doi.wiley.com/10.1111/1755-0998.12067		2013-05	2021-10-12 2:33:	2021-10-12 2:33:		528-537		3	13	en
XICSYEXR	journalArticle	2013	Morueta-Holme,	Habitat area and climate stability determine geograp	1461023X	10.1111/ele.1218	http://doi.wiley.com/10.1111/ele.12184		2013-12	2021-10-12 2:33:	2021-10-12 2:33:		1446-1454		12	16	en
EDJCNFMJ	journalArticle	2013	Beaulieu, Michae	FRUIT EVOLUTION AND DIVERSIFICATION IN CA	143820	10.1111/evo.1218	http://doi.wiley.com/10.1111/evo.12180		2013-11	2021-10-12 2:33:	2021-10-12 2:33:		3132-3144		11	67	en
IUA5YYZD	journalArticle	2013	Moreau, Sorina, I	The Arabidopsis oligopeptidases TOP1 and TOP2 a	9607412	10.1111/tpj.1232	http://doi.wiley.com/10.1111/tpj.12320		2013-11	2021-10-12 2:33:	2021-10-12 2:33:		603-614		4	76	en
KABWWKCK	journalArticle	2013	Harris, M., S. E.;	DNA Barcoding from NYC to Belize	0036-8075, 1095	10.1126/science.	http://www.sciencemag.org/cgi/doi/10.1126/science.1230006		2013-12-20	2021-10-12 2:33:	2021-12-07 19:3		1462-1463		6165	342	en
DJPKVTRC	journalArticle	2013	Amborella Genor	The Amborella Genome and the Evolution of Flower	0036-8075, 1095	10.1126/science.	http://www.sciencemag.org/cgi/doi/10.1126/science.1241089		2013-12-20	2021-10-12 2:33:	2021-12-07 19:3		1241089-1241089		6165	342	en
DDICLZUW	journalArticle	2013	ter Steege, M. R.	Hyperdominance in the Amazonian Tree Flora	0036-8075, 1095	10.1126/science.	http://www.sciencemag.org/cgi/doi/10.1126/science.1243092		2013-10-18	2021-10-12 2:33:	2021-12-07 19:3		1243092-1243092		6156	342	en
S6JKECCR	journalArticle	2013	Service, R. F.	Biology's Dry Future	0036-8075, 1095	10.1126/science.	http://www.sciencemag.org/cgi/doi/10.1126/science.342.6155.186		2013-10-11	2021-10-12 2:33:	2021-12-07 19:3		186-189		6155	342	en
GV85PI2Y	conferencePaper	2013	Gorton, Kerstin, I	Build less code deliver more scien	978-1-4503-2122-8	10.1145/246544	http://dl.acm.org/citation.cfm?doid=2465449.2465452		2013	2021-10-12 2:33:	2021-10-12 2:33:		159				en
9FEG2VJS	conferencePaper	2013	Mohan, Gregory	Developing a high-volume batch s	978-1-4503-2170-9	10.1145/248476	http://dl.acm.org/citation.cfm?doid=2484762.2484784		2013	2021-10-12 2:33:	2021-10-12 2:33:		1				en
P2M8NBT4	conferencePaper	2013	Koesterke, J. M.,	Optimizing the PCIT algorithm on s	978-1-4503-2170-9	10.1145/248476	http://dl.acm.org/citation.cfm?doid=2484762.2484794		2013	2021-10-12 2:33:	2021-10-12 2:33:		1				en
Z9XYTHK4	conferencePaper	2013	Miller, Wayne, M	Embedding CIPRES science gate	978-1-4503-2170-9	10.1145/248476	http://dl.acm.org/citation.cfm?doid=2484762.2484806		2013	2021-10-12 2:33:	2021-10-12 2:33:		1				en
R3ZX936Y	journalArticle	2013	Wollbrett, Manue	Clever generation of rich SPARQL queries from ann	1471-2105	10.1186/1471-21	https://bmcbioinformatics.biomedcentral.com/articles/10.1186/1471-2105-14-126		2013-12	2021-10-12 2:33:	2021-10-12 2:33:		126		1	14	en
HBSLZ3FJ	journalArticle	2013	Stoltzfus, Greg, A	Phylotastic! Making tree-of-life knowledge accessibl	1471-2105	10.1186/1471-21	https://bmcbioinformatics.biomedcentral.com/articles/10.1186/1471-2105-14-158		2013-12	2021-10-12 2:33:	2021-10-12 2:33:		158		1	14	en
LR8RH73N	journalArticle	2013	Boyle, Brian J, B	The taxonomic name resolution service: an online to	1471-2105	10.1186/1471-21	https://bmcbioinformatics.biomedcentral.com/articles/10.1186/1471-2105-14-16		2013-12	2021-10-12 2:33:	2021-10-12 2:33:		16		1	14	en
2Z3JW6P2	journalArticle	2013	Wang, Lincoln D,	Modeling the evolution dynamics of exon-intron stru	1471-2148	10.1186/1471-21	http://bmcevolbiol.biomedcentral.com/articles/10.1186/1471-2148-13-57		2013	2021-10-12 2:33:	2021-10-12 2:33:		57		1	13	en
Q3375BJ5	journalArticle	2013	Liseron-Monfils, I	Promzea: a pipeline for discovery of co-regulatory m	1471-2229	10.1186/1471-22	http://bmcplantbiol.biomedcentral.com/articles/10.1186/1471-2229-13-42		2013	2021-10-12 2:33:	2021-10-12 2:33:		42		1	13	en
333D6EWN	journalArticle	2013	Ming, Jane, Ray;	Genome of the long-living sacred lotus (Nelumbo nu	1465-6906	10.1186/gb-2013	http://genomebiology.biomedcentral.com/articles/10.1186/gb-2013-14-5-r41		2013	2021-10-12 2:33:	2021-10-12 2:33:		R41		5	14	en
ELK59T94	journalArticle	2013	Chamberlain, Ed	taxize: taxonomic search and retrieval in R	2046-1402	10.12688/f1000re	https://f1000rese	All species are hierarchically related to one another, and we use taxonomic names to label the nodes in this hierarc	2013-10-28	2021-10-12 2:33:	2021-12-07 19:3		191			2	en
44LCMC8Y	journalArticle	2013	Burleigh, Mengjie	Next-generation phenomics for the Tree of Life	2157-3999	10.1371/currents	http://currents.plos.org/treeoflife/index.html%3Fp=2583.html		2013	2021-10-12 2:33:	2021-10-12 2:33:						en
JYQR3FSX	journalArticle	2013	Rodrigo, Brian, A	Science Incubators: Synthesis Centers and Their Ro	1545-7885	10.1371/journal.p	http://dx.plos.org/10.1371/journal.pbio.1001468		2013-01-15	2021-10-12 2:33:	2021-12-07 19:3		e1001468		1	11	en
7TCKKKAY	journalArticle	2013	Soderlund, David	TCW: Transcriptome Computational Workbench	1932-6203	10.1371/journal.p	https://dx.plos.org/10.1371/journal.pone.0069401		2013-07-17	2021-10-12 2:33:	2021-12-07 19:3		e69401		7	8	en
HPLXZA2V	journalArticle	2013	Ramamoorthy, A	The Putative C2H2 Transcription Factor MtfA Is a No	1932-6203	10.1371/journal.p	https://dx.plos.org/10.1371/journal.pone.0074122		2013-09-16	2021-10-12 2:33:	2021-12-07 19:3		e74122		9	8	en
ZQ35JSGM	journalArticle	2013	Rothfels, Kathlee	Transcriptome-Mining for Single-Copy Nuclear Mark	1932-6203	10.1371/journal.p	https://dx.plos.org/10.1371/journal.pone.0076957		2013-10-08	2021-10-12 2:33:	2021-12-07 19:3		e76957		10	8	en
BDI5N8Y7	journalArticle	2013	Miller, Sean, Allis	Vitis Phylogenomics: Hybridization Intensities from a	1932-6203	10.1371/journal.p	https://dx.plos.org/10.1371/journal.pone.0078680		2013-11-13	2021-10-12 2:33:	2021-12-07 19:3		e78680		11	8	en
CAV3ICD9	journalArticle	2013	Kültz, Anne E., D	New Frontiers for Organismal Biology	1525-3244, 0006	10.1525/bio.2013	https://academic.oup.com/bioscience/article-lookup/doi/10.1525/bio.2013.63.6.8		2013-06	2021-10-12 2:33:	2021-10-12 2:33:		464-471		6	63	en
9Q596D69	journalArticle	2013	von Mogel, Karl H	Taking the into the Field	1529-9163	10.2134/csa2013	https://www.agronomy.org/publications/csa/abstracts/58/3/4		2013	2021-10-12 2:33:	2021-10-12 2:33:		4		3	58	en
ZU8ZXD5V	journalArticle	2013	Khoury, Paul C.,	An Inventory of Crop Wild Relatives of the United St	0011-183X	10.2135/cropsci2	https://www.crops.org/publications/cs/abstracts/53/4/1496		2013	2021-10-12 2:33:	2021-10-12 2:33:		1496		4	53	en
V7DNF2JW	journalArticle	2013	McCarthy, E., F. I	From data to function: Functional modeling of poultr	0032-5791, 1525	10.3382/ps.2012	https://academic.oup.com/ps/article-lookup/doi/10.3382/ps.2012-02808		2013-09-01	2021-10-12 2:33:	2021-12-07 19:3		2519-2529		9	92	en
S7B7ID8M	journalArticle	2013	Lehan, Bethany A	Accidental introductions are an important source of i	29122	10.3732/ajb.130	http://doi.wiley.com/10.3732/ajb.1300061		2013-07	2021-10-12 2:33:	2021-10-12 2:33:		1287-1293		7	100	en
ACIL2WER	journalArticle	2013	Stull, Matthew A.	A Targeted Enrichment Strategy for Massively Parall	2168-0450	10.3732/apps.12	http://doi.wiley.com/10.3732/apps.1200497		2013-02	2021-10-12 2:33:	2021-10-12 2:33:		1200497		2	1	en
J7VF5E32	journalArticle	2013	Eichten, Nathan I	Variation in DNA Methylation Patterns is More Comn	1940-3372	10.3835/plantger	https://www.crops.org/publications/tpg/abstracts/6/2/plantgenome2012.06.0009		2013	2021-10-12 2:33:	2021-10-12 2:33:		0		2	6	en

554NQZ88	journalArticle	2013	Deck, John, Johr	Clarifying Concepts and Terms in Biodiversity Inform			1944-3277	10.4056/sigs.390	http://www.standardsingenomics.org/index.php/sigen/article/view/sigs.3907833		2013-05-31	2021-10-12 2:33	2021-12-07 19:3		352-359		2	8										en
946FW4RZ	journalArticle	2013	Swenson, Richar	Sustainable Software Development for Next-Gen Sequencing (NGS) Bioinformatics on					http://arxiv.org/ab	DNA sequence analysis is fundamental to life science research. The rapid development of next generation sequen	2013-10-26	2021-10-12 2:33	2021-12-07 19:3															
IAVLWMLN	journalArticle	2013	Moore, Reagan V	Extensible Generic Data Management Software					http://arxiv.org/ab	Extensibility mechanisms constitute a form of knowledge capture that is essential for software re-use. The Data Int	2013-08-30	2021-10-12 2:33	2021-12-07 19:3															
PIQRWXDE	journalArticle	2013	Gnimpieba, Caro	Bioinformatics Knowledge Transmission (training, learning, and teaching): overview anc					http://arxiv.org/ab	The merger of computer science, mathematics, and life sciences has brought about the discipline known as bioinfo	2013-10-31	2021-10-12 2:33	2021-12-07 19:3															
5L8XMSPG	journalArticle	2013	Crochemore, Ton	Order-Preserving Suffix Trees and Their Algorithmic Applications					http://arxiv.org/ab	Recently Kubica et al. (Inf. Process. Let., 2013) and Kim et al. (submitted to Theor. Comp. Sci.) introduced order-pr	2013-03-27	2021-10-12 2:33	2021-12-07 19:3															
YTE4CA8E	journalArticle	2013	Waters, Nathan M	Comprehensive analysis of imprinted genes in maize reveals limited conservation with c					http://arxiv.org/ab	In plants, a subset of genes exhibit imprinting in endosperm tissue such that expression is primarily from the mater	2013-07-29	2021-10-12 2:33	2021-12-07 19:3															
3QARSUY3	journalArticle	2013	Gessler, Jill, Dam	iPlant SSWAP (Simple Semantic Web Architecture and Protocol) enables semantic pipelines for biodiversity							2013	2021-10-12 2:33	2021-10-12 2:33		101													
4UMWFBM5	journalArticle	2013	Eichten, Steven F	Identification and characterization of DNA methylation variation within maize							2013	2021-10-12 2:33	2021-10-12 2:33															
PVI7VVTW	book	2013		2013 International Conference on :		978-0-7695-5137-1 978-1-4799-1519-4					2013	2021-10-12 2:33	2021-10-12 2:33															eng
W2D9PRIW	journalArticle	2013	Rajasekar, Sharli	Design of the DataBridge: A System for Sociometric Analysis of Long tail Science Data							2013	2021-10-12 2:33	2021-12-03 18:5															
WQD2ZVS7	journalArticle	2013	Eichten, Peter; ol	Epigenetic and genetic influences on DNA methylation variation in maize populations							2013	2021-10-12 2:33	2021-10-12 2:33		2783–2797		8	25										
ET2W8CWW	journalArticle	2013	Busov, Victor	The Promise of Genetics and Beyond						This review aims at providing a short synopsis of the history, advances and future of genom- ics sciences in variou	2013-09-24	2021-10-12 2:33	2021-12-07 19:3		107-128		2	19										
4WMYH97K	thesis	2013	Oaks, Jamie Rich	Islands and Integrals: Processes of Diversification in an Island Archipelago and Bayesian Methods of Comparative Phylogeographical Model Choice							2013	2021-10-12 2:33	2021-10-12 2:33															
66ZSSEEP	conferencePaper	2013	Predoehl, Kobus	A statistical model for recreational trails in aerial images							2013	2021-10-12 2:33	2021-10-12 22:4		337–344													
ZQJZJZSW	journalArticle	2013	Narjala, Lakshmi	Color based classification of circular markers for the identification of experimental units							2013	2021-10-12 2:33	2021-10-12 2:33															
BQFDAWUC	journalArticle	2013	Rajasekar, Justin	The databridge							2013	2021-10-12 2:33	2021-10-12 2:33		1–14		1	2										
P28J5HDS	conferencePaper	2013	Chen, David, Eric	The dynamics of functional classes of plant genes in rediploidized ancient polyploids							2013	2021-10-12 2:33	2021-10-12 2:33		S19			14										
JNE7GA54	conferencePaper	2013	Izquierdo-Carras	A generic vectorization scheme and a gpu kernel for the phylogenetic likelihood library							2013	2021-10-12 2:33	2021-10-12 2:33		530–538													
9IR96HZN	conferencePaper	2013	Flouri, Alexandro	An optimal algorithm for computing all subtree repeats in trees							2013	2021-10-12 2:33	2021-10-12 2:33		269–282													
HDSPP743	conferencePaper	2013	Crochemore, Ton	Order-preserving incomplete suffix trees and order-preserving indexes							2013	2021-10-12 2:33	2021-10-12 2:33		84–95													
4TDQ2VKP	conferencePaper	2013	Gnimpieba, Caro	Multidisciplinarity in Knowledge Transmision Management System (KTMS) evaluation							2013	2021-10-12 2:33	2021-10-12 2:33		238													
478BGWDP	conferencePaper	2013	Del Rio, CJ, Nich	Elseweb meets sadi: Supporting data-to-model integration for biodiversity forecasting							2013	2021-10-12 2:33	2021-10-12 2:33															
GAPGLBDK	journalArticle	2013	Jordan, Sivakum	Responses to Data Management Requirements at the National Scale							2013	2021-10-12 2:33	2021-10-12 2:33		64													
JXQID4YE	journalArticle	2013	Gnimpieba, Caro	RESTful API in life science research systems and data integration challenges: linking metabolic pathway, metabolic network, gene and publication.							2013	2021-10-12 2:33	2021-10-12 2:33		1196–1199			10										
I7CHC45A	journalArticle	2013	Kim, Jeonghyun	Data sharing and its implications for academic libraries							2013	2021-10-12 2:33	2021-10-12 2:33		494–506		12-Nov	114										
6SKXGH78	conferencePaper	2013	Stanzione, Danie	Keynote: The iPlant Collaborative: A Life Sciences Cyberinfrastructure for the 21st Century						The iPlant Collaborative (iPlant) is a large scale United States National Science Foundation (NSF) funded project tl	2013-05-21	2021-10-12 2:33	2021-12-07 19:3															
XTA5XXJ7	journalArticle	2013	Soltis, W Brad, D	The potential of genomics in plant systematics							2013	2021-10-12 2:33	2021-10-12 2:33		886–898		5	62										
HE8R947W	conferencePaper	2013	Lee, Robert K, M	VegBank: a permanent online repository for international plot and relevé data							2013	2021-10-12 2:33	2021-10-12 2:33		66													
FF97QSTQ	journalArticle	2013	Flouri, Wojciech,	Enhanced string covering							2013	2021-10-12 2:33	2021-10-12 2:33		102–114			506										
CL34EF2K	journalArticle	2013	Gessler, T, Damia	A posteriori ontology engineering for data-driven science							2013	2021-10-12 2:33	2021-10-12 2:33															
DW3MEZ3S	conferencePaper	2013	Fritz, J.M., E.R.;	Identification of SNPs in Water Buffalo using next generation sequencing data							2013	2021-10-12 2:33	2021-10-12 2:33															
DYYDCLS8	conferencePaper	2013	Vasquez-Gross,	CartograTree: Connection Forest Tree Genomes, Phenotypes, and Environment							2013	2021-10-12 2:33	2021-10-12 2:33															
YKBUUK76	conferencePaper	2013	Stein, Joshua; Je	Biological Computing Using the iPlant Discovery Environment							2013	2021-10-12 2:33	2021-10-12 2:33															
IC46DXU8	conferencePaper	2013	Spalding, Nirav M	Enabling Cloud and GRid Powered Image Phenotyping							2013	2021-10-12 2:33	2021-10-12 2:33															
J4P7PJ5F	conferencePaper	2013	Galbraith, Stephe	Gene Exression in Inbred and Hybrid Mice, Young and Old							2013	2021-10-12 2:33	2021-10-12 2:33															
YYEVZUN4	conferencePaper	2013	Gregory, Eric Lyc	EPIC CoGE- a unified browser for Epigenetics							2013	2021-10-12 2:33	2021-10-12 2:33															
TU2CY9CE	book	2013	Micklos, David A.	Genome science: a practical and c		978-0-87969-859-1					2013	2021-12-06 19:3	2021-12-06 19:3			692					Genome science					Cold Spring Harb	Cold Spring Harbor, N.Y	
L4K5SGLK	journalArticle	2012	Schnable, Eric, J	Genome-Wide Analysis of Syntenic Gene Deletion ir			1759-6653	10.1093/gbe/evs	https://academic.oup.com/gbe/article-lookup/doi/10.1093/gbe/evs009		2012	2021-10-12 2:30	2021-10-12 2:30		265-277		3	4										en
IW25AULP	journalArticle	2012	Egan, David M.,	Applications of next-generation sequencing in plant l			29122	10.3732/ajb.120	http://doi.wiley.com/10.3732/ajb.1200020		2012-02	2021-10-12 2:30	2021-10-12 2:30		175-185		2	99										en
GKIIYX3H	journalArticle	2012	Jiao, Claude W,	A genome triplication associated with early diversific			1465-6906	10.1186/gb-2012	http://genomebiology.biomedcentral.com/articles/10.1186/gb-2012-13-1-r3		2012	2021-10-12 2:30	2021-10-12 2:30		R3		1	13										en
C7UQDUNF	journalArticle	2012	Parr, Roderic D.M	Evolutionary informatics: unifying knowledge about t			1695347	10.1016/j.tree.20	https://linkinghub.elsevier.com/retrieve/pii/S0169534711003247		2012-02	2021-10-12 2:30	2021-10-12 2:30		94-103		2	27										en
8ETGZ57W	journalArticle	2012	Reddy, Asa, Anire	Deciphering the Plant Splicing Code: Experimental a			1664-462X	10.3389/fpls.201	http://journal.frontiersin.org/article/10.3389/fpls.2012.00018/abstract		2012	2021-10-12 2:30	2021-10-12 2:30					3										
2EYWASGX	journalArticle	2012	Reinders, Anke	Evolution of plant sucrose uptake transporters			1664462X	10.3389/fpls.201	http://journal.frontiersin.org/article/10.3389/fpls.2012.00022/abstract		2012	2021-10-12 2:30	2021-10-12 2:30					3										
PTRIF8SU	journalArticle	2012	Kolker, Vural, Eu	Opportunities and Challenges for the Life Sciences (			1536-2310, 1557	10.1089/omi.201	http://www.liebertpub.com/doi/10.1089/omi.2011.0152		2012-03	2021-10-12 2:30	2021-10-12 2:30		138-147		3	16										en
KGHAGUY7	journalArticle	2012	Miller, Dmitry V.,	Structure-Forming Corals and Sponges and Their U			1932-6203	10.1371/journal.p	https://dx.plos.org/10.1371/journal.pone.0033885		2012-03-21	2021-10-12 2:30	2021-12-07 19:2		e33885		3	7										en
F4DN6LBW	journalArticle	2012	Ram, Jun, Sudha	A Semantic Foundation for Provenance Managemer			1861-2032, 1861	10.1007/s13740-	http://link.springer.com/10.1007/s13740-012-0002-0		2012-05	2021-10-12 2:30	2021-10-12 2:30		17-Nov		1	1										en
ZXX3EX8A	journalArticle	2012	Zhou, Ruth, Lecc	Mining for Meaning: Visualization Approaches to De			1536-2310, 1557	10.1089/omi.201	http://www.liebertpub.com/doi/10.1089/omi.2011.0111		2012-04	2021-10-12 2:30	2021-10-12 2:30		208-228		4	16										en
68298I8L	journalArticle	2012	Schatz, W Richa	Current challenges in de novo plant genome sequer			1465-6906	10.1186/gb-2012	http://genomebiology.biomedcentral.com/articles/10.1186/gb-2012-13-4-243		2012	2021-10-12 2:30	2021-10-12 2:30		243		4	13										en
YRLWG5M2	journalArticle	2012	Beck, Carsten F.,	What's on the horizon for macroecology?			9067590	10.1111/j.1600-0	http://doi.wiley.com/10.1111/j.1600-0587.2012.07364.x		2012-08	2021-10-12 2:30	2021-10-12 2:30		673-683		8	35										en
PJUE9VB9	journalArticle	2012	Stamatakis, F., A	RAxML-Light: a tool for computing terabyte phyloger			1367-4803, 1460	10.1093/bioinforr	https://academic.oup.com/bioinformatics/article-lookup/doi/10.1093/bioinformatics/bts309		2012-08-01	2021-10-12 2:30	2021-12-07 19:2		2064-2066		15	28										en
VR9QDVXF	journalArticle	2012	Alakonya, Neelim	Interspecific RNA Interference of <i>SHOOT MERIS			1040-4651, 1532	10.1105/tpc.112.0	http://www.plantcell.org/lookup/doi/10.1105/tpc.112.099994		2012-07	2021-10-12 2:30	2021-10-12 2:30		3153-3166		7	24										en
KQFEGLNW	journalArticle	2012	Schneider, Todd	Ontology for Big Systems: The Ontology Summit 20			1570-5838	10.3233/AO-201	http://www.medra	Ali, Hashemi; Mike, Bennett; Mary, Brady; Cory, Casanave; Henson, Graves; Michael, Gruninger; Nicola, Guarino;	2012	2021-10-12 2:30	2021-12-06 19:2		357–371		3											
5DMIQ4W8	journalArticle	2012	D'Hont, Patrick,	The banana (Musa acuminata) genome and the evo			0028-0836, 1476	10.1038/nature1	http://www.nature.com/articles/nature11241		2012-08	2021-10-12 2:30	2021-10-12 2:30		213-217		7410	488										en
3RV3HLAD	journalArticle	2012	Kakumanu, Andy	Effects of Drought on Gene Expression in Maize Re			0032-0889, 1532	10.1104/pp.112.2	http://www.plantphysiol.org/lookup/doi/10.1104/pp.112.200444		2012-10	2021-10-12 2:30	2021-10-12 2:30		846-867		2	160										en
26PUPLZL	journalArticle	2012	Tang, Haibo; Lyo	Unleashing the Genome of Brassica Rapa			1664-462X	10.3389/fpls.201	http://journal.frontiersin.org/article/10.3389/fpls.2012.00172/abstract		2012	2021-10-12 2:30	2021-12-06 19:4					3										
FDXKPBKW	journalArticle	2012	Smith, Brian C.,	treePL: divergence time estimation using penalized l			1460-2059, 1367	10.1093/bioinforr	https://academic.oup.com/bioinformatics/article-lookup/doi/10.1093/bioinformatics/bts492		2012-10-15	2021-10-12 2:30	2021-12-07 19:2		2689-2690		20	28										en
U3MLACC2	journalArticle	2012	O'Meara, Brian C	Evolutionary Inferences from Phylogenies: A Review			1543-592X, 154	10.1146/annurev	http://www.annualreviews.org/doi/10.1146/annurev-ecolsys-110411-160331		2012-12	2021-10-12 2:30	2021-10-12 2:30		267-285		1	43										en
9JCRF3DK	journalArticle	2012	Claros, Noé, Mar	Why Assembling Plant Genome Sequences Is So Cl			2079-7737	10.3390/biology1	http://www.mdpi.com/2079-7737/1/2/439		2012-09-18	2021-10-12 2:30	2021-12-07 19:2		439-459		2	1										en
5BHRPLPZ	journalArticle	2012	Huang, Barbara	Association between the geographic distribution duri			29122	10.3732/ajb.120	http://doi.wiley.com/10.3732/ajb.1200348		2012-11	2021-10-12 2:30	2021-10-12 2:30		1866-1874		11	99										en
NC6UZ8K7	journalArticle	2012	Blonder, Brian J.,	The leaf-area shrinkage effect can bias paleoclimate			29122	10.3732/ajb.120	http://doi.wiley.com/10.3732/ajb.1200062		2012-11	2021-10-12 2:30	2021-10-12 2:30		1756-1763		11	99										en
YS4TSTP8	journalArticle	2012	Johnson, Gane K	Evaluating Methods for Isolating Total RNA and Prec			1932-6203	10.1371/journal.p	http://dx.plos.org/10.1371/journal.pone.0050226		2012-11-21	2021-10-12 2:30	2021-12-07 19:2		e50226		11	7										en
IWLPTZXR	journalArticle	2012	Eichten, Nathan	Spreading of Heterochromatin Is Limited to Specific			1553-7404	10.1371/journal.p	https://dx.plos.org/10.1371/journal.pgen.1003127		2012-12-13	2021-10-12 2:30	2021-12-07 19:2		e1003127		12	8										en
G2P2AVJH	conferencePaper	2012	Lyons, James C.	Whole Genome Comparisons with Oryza and Brachypodium using CoGe and SynMap					https://pag.confe	Of the more than 30 plant genomes currently available, the Poaceae represents the most heavily sample family of	2012-01-14	2021-10-12 2:30	2021-12-07 19:2		W267													
FP4UR3TG	conferencePaper	2012	Eichten, Steven F	Contribution of Epigenetic Variation to Expression Changes Among Tissues and Genoty					https://pag.confe	Epigenetic variation describes heritable differences that are not attributable to changes in DNA sequence. In particu	2012-01-17	2021-10-12 2:30	2021-12-07 19:2		W417													
8GQKALUG	conferencePaper	2012	McKay, Sheldon	How to Use the iPlant's Discovery Environment for Your Research					https://pag.confe	The iPlant Discovery Environment (DE) is a system for managing and running tools, analyses, and workflows. The	2012-01-17	2021-10-12 2:30	2021-12-07 19:2		W420													
5L53HXQS	conferencePaper	2012	McKay, Matt, She	The iPlant Collaborative					https://pag.confe	The iPlant Collaborative's mission is to create a comprehensive set of cyberinfrastructure to support plant research	2012-01-17	2021-10-12 2:30	2021-12-07 19:2		W416													
G5G2RYEJ	conferencePaper	2012	Miller, Nathan	Enabling Phenotypic Image Analysis Using Shared Cyberinfrastructure					https://pag.confe	Understanding how an organism's genotype and environment influence its growth, development and physiology re	2012-01-17	2021-10-12 2:30	2021-12-07 19:2		W418													
LLVNL7NU	conferencePaper	2012	Tang, Konstantin	Leverage iPlant Computing Resources for Collaborative Genomic Research					https://pag.confe	The ability to sequence novel genomes using high-throughput methods has tremendously enabled the decoding of	2012-01-17	2021-10-12 2:30	2021-12-07 19:2		W419													
MZ997I33	thesis	2012	Kakumanu, Aksh	Effects of Drought on Gene Expression in Maize Reproductive and Leaf Meristem Tissu					http://hdl.handle.	Drought is a major environmental stress factor that poses a serious threat to food security. The effects of drought o	2012-06-28	2021-10-12 2:30	2021-12-07 19:2															
XWAR9FHZ	conferencePaper	2012	Klinov, Thomas, I	Incrementally Updateable and Persistent Decomposition of OWL Ontologies					http://ceur-ws.org/Vol-849/paper_7.pdf		2012	2021-10-12 2:30	2021-10-12 2:30		7			849										
A7NR2EPY	conferencePaper	2012	Fox, Dennis, Ge	Using Clouds for Technical Computing.							2012	2021-10-12 2:33	2021-10-12 2:33		81–102													
5WB7BBKB	journalArticle	2012	Das, Joshua S, A	Infrastructure for managing and analyzing biological networks derived from collections of plant images							2012	2021-10-12 2:36	2021-12-03 18:5															
VSTBIAB3	journalArticle	2012	McDonald, Steve	Latest Technology in Irrigation Management in Nursery Production\copyright							2012	2021-10-12 2:36	2021-10-12 2:36		89–93													
QDPYTUBC	bookSection	2012	Council, National	Implementing the Research Strategy and Evaluating Progress							2012	2021-10-12 2:36	2021-10-12 2:36															
R5DNEVRW	bookSection	2012	Cole, Benjamin J	Image-Based An	High-Throughput	978-1-61779-994-5 978-1-61779-995-2			http://link.springer.com/10.1007/978-1-61779-995-2_1		2012	2021-12-06 19:2	2021-12-06 19:2	2021-12-06 19:2	1-7			918								Humana Press	Totowa, NJ	
MLJC2MY2	journalArticle	2012	Gessler, Damian	iPlant Semantic Web Platform uses SSWAP (Simple Semantic Web Architecture and Protocol) to enable Semantic Pipelines across Distributed Web and High Performance Computing Resources							2012	2021-12-06 19:3	2021-12-06 19:3															
V56HKSU3	conferencePaper	2012	Dragut, Eduard C	Lonomics Atlas: a	Proceedings of th	978-1-4503-1156-4		10.1145/2396761	http://dl.acm.org/citation.cfm?doid=2396761.2398724		2012	2021-12-06 19:3	2021-12-06 19:3	2021-12-06 19:3	2680						Lonomics Atlas					ACM Press	Maui, Hawaii, US	en
R3VUMJGG	journalArticle	2012	Geser, Guntram;	Building blocks fo	AGRIS on-line Papers in Economics and Informatics						2012	2021-12-06 19:3	2021-12-06 19:3		11–18		665-2016-44926	4										
RXMLM2LJ	journalArticle	2012	Tang, H; Lyons, E	The Evolution of	Int J Evol 1						2012	2021-12-06 19:4	2021-12-06 19:4		2			1										
8AMPNVZS	journalArticle	2012	White, Jeffrey W.	Field-based pher	Field Crops Research		3784290	10.1016/j.fcr.201	https://linkinghub.elsevier.com/retrieve/pii/S037842901200130X		2012-07	2021-12-06 19:4	2021-12-07 19:2	2021-12-06 19:4	101-112			133		Field Crops Research								en
L8NWXIY8	journalArticle	2012	Beaulieu, Jeremy	Synthesizing phy	Ecology		0012-9658	10.1890/11-0638	http://doi.wiley.com/10.1890/11-0638.1		2012-08	2021-10-12 2:28	2021-12-07 19:2	2021-12-06 19:4	S4-S13		sp8	93		Ecology								en
3YI9S455	journalArticle	2012	Kvilekval, Kristiar	Bisque: Advance	IEEE Data Eng. Bull.						2012	2021-12-06 19:4	2021-12-06 19:4		56–64		3	35										
8W2KU296	conferencePaper	2012	Miller, Mark A.; P	The CIPRES scie	Proceedings of th	978-1-4503-1602-6		10.1145/2335755	http://dl.acm.org/citation.cfm?doid=2335755.2335836		2012	2021-12-06 19:4	2021-12-06 19:4	2021-12-06 19:4	1						The CIPRES science gateway					ACM Press	Chicago, Illinois	en
6MD3DNC9	journalArticle	2012	Christou, Michalis	Computing all su	Information Processing Letters		200190	10.1016/j.ipl.201	https://linkinghub.elsevier.com/retrieve/pii/S0020019012002505		2012-12	2021-12-06 19:4	2021-12-07 19:2	2021-12-06 19:4	958-962		24	112		Information Processing Letters								en
S4II5PZ4	conferencePaper	2012	Woodward, Nicho	On automatically	Proceedings of th	978-1-4503-1472-5		10.1145/2348283	http://dl.acm.org/citation.cfm?doid=2348283.2348494		2012	2021-12-06 19:4	2021-12-06 19:4	2021-12-06 19:4	1111											ACM Press	Portland, Oregon	en
E4RBQCTP	conferencePaper	2012	Dooley, Rion; Va	Software-as-a-se	5th IEEE Workshop on Many-Task Computing on Grids and Supercomputers (MTAGS)						2012	2021-12-06 19:5	2021-12-06 19:5													Citeseer		
X8K5NZCW	conferencePaper	2012	Dooley, R; Vaugh	Your data, your w	Proceedings of The Third International Workshop on Data Intensive C				http://www.cse.buffalo.edu/faculty/tkosar/DataCloud2012/papers/datacloud2012_paper_3.pdf		2012	2021-12-06 19:5	2021-12-06 19:5													DataCloud		
3FTE9AWA	conferencePaper	2012	Simmerman, Sco	Exploring similari	Proceedings of th	978-1-4503-1602-6		10.1145/2335755	http://dl.acm.org/citation.cfm?doid=2335755.2335835		2012	2021-12-06 19:5	2021-12-06 19:5	2021-12-06 19:5	1											ACM Press	Chicago, Illinois	en
GGJFSRFH	journalArticle	2012	Straub, Shannon	Navigating the tip	American Journal of Botany		29122	10.3732/ajb.110	http://doi.wiley.com/10.3732/ajb.1100335		2012-02	2021-10-12 2:28	2021-12-07 19:2	2021-12-06 19:5	349-364		2	99		American Journa	Navigating the tip of the genomic iceberg							en
7LTB6EAL	journalArticle	2012	Strickler, Susan F	Designing a trans	American Journal of Botany		29122	10.3732/ajb.110	http://doi.wiley.com/10.3732/ajb.1100292		2012-02	2021-10-12 2:28	2021-12-07 19:2	2021-12-06 19:5	257-266		2	99		American Journal of Botany								en
KSAI8BC7	conferencePaper	2012	Flouri, Tomás; Ili	New and Efficien	Stringology	978-80-01-05095-8			http://www.stringology.org/papers/PSC2012.pdf#page=83		2012	2021-12-06 19:5	2021-12-06 19:5		75–88													
5UMT6MI9	bookSection	2012	Flouri, Tomáš; Ili	GapMis-OMP: Pa	Artificial Intelliger	978-3-642-33411-5 978-3-642-33412-2			http://link.springer.com/10.1007/978-3-642-33412-2_61		2012	2021-12-06 19:5	2021-12-06 19:5	2021-12-06 19:5	593-601			382			GapMis-OMP					Springer Berlin H	Berlin, Heidelberg	
4UT2AQPC	conferencePaper	2012	Kharitonova, Yek	Client-side backp	Proceedings of th	978-1-4503-1089-5		10.1145/2393347	http://dl.acm.org/citation.cfm?doid=2393347.2396368		2012	2021-12-06 19:5	2021-12-06 19:5	2021-12-06 19:5	1005											ACM Press	Nara, Japan	en
T2HJ5PHY	journalArticle	2011	Wang, Thomas F	Regulatory mechanisms underlying C4 photosynthe			0028646X	10.1111/j.1469-8	http://doi.wiley.com/10.1111/j.1469-8137.2011.03649.x		2011-04	2021-10-12 2:28	2021-10-12 2:28		20-Sep		1	190										en
VHE4MN9J	journalArticle	2011	Smith, M. J., S. A	Understanding angiosperm diversification using sma			0002-9122, 1537	10.3732/ajb.100	http://doi.wiley.com/10.3732/ajb.1000481		2011-03-01	2021-10-12 2:28	2021-12-07 19:2		404-414		3	98										en
BTGKLPD4	conferencePaper	2011	Rogowitz, Naim,	Metadata Mapper: a web service for mapping data between independ				10.1117/12.8817	http://proceedings.spiedigitallibrary.org/proceeding.aspx?doi=10.1117/12.881734		2011-02-10	2021-10-12 2:28	2021-12-07 19:2		78650I													
EIWPBS5V	journalArticle	2011	Macilwain, Colin	Systems Biology: Evolving into the Mainstream			928674	10.1016/j.cell.20	https://linkinghub.elsevier.com/retrieve/pii/S0092867411002340		2011-03	2021-10-12 2:28	2021-10-12 2:28		839-841		6	144										en
Y68EVMMK	journalArticle	2011	Nodine, Frans E.	A few standing for many: embryo receptor-like kinas			13601385	10.1016/j.tplants.	https://linkinghub.elsevier.com/retrieve/pii/S1360138511000082		2011-04	2021-10-12 2:28	2021-10-12 2:28		211-217		4	16										en
NR7RWFBI	journalArticle	2011	Banks, I. V., J. A.	The Selaginella Genome Identifies Genetic Changes			0036-8075, 1095	10.1126/science.	http://www.sciencemag.org/cgi/doi/10.1126/science.1203810		2011-05-20	2021-10-12 2:28	2021-12-07 19:2		960-963		6032	332										en
TTFIPTLQ	journalArticle	2011	Tang, Michael, H	Screening synteny blocks in pairwise genome comp			1471-2105	10.1186/1471-21	https://bmcbioinformatics.biomedcentral.com/articles/10.1186/1471-2105-12-102		2011-12	2021-10-12 2:28	2021-10-12 2:28		102		1	12										en
FHJ9E77Y	journalArticle	2011	Feng, Lincoln, Xi	PeakRanger: A cloud-enabled peak caller for ChIP-s			1471-2105	10.1186/1471-21	https://bmcbioinformatics.biomedcentral.com/articles/10.1186/1471-2105-12-139		2011-12	2021-10-12 2:28	2021-10-12 2:28		139		1	12										en
RW3M76Y8	journalArticle	2011	Schaeffer, C. J., I	MaizeGDB: curation and outreach go hand-in-hand			1758-0463	10.1093/databas	https://academic.oup.com/database/article-lookup/doi/10.1093/database/bar022		2011-05-29	2021-10-12 2:28	2021-12-07 19:2		bar022-bar022		0	2011										en
JRD2PSZK	journalArticle	2011	Liu, Sudha, Jun;	Who does what: Collaboration patterns in the wikipe			2158656X	10.1145/1985347	http://dl.acm.org/citation.cfm?doid=1985347.1985352		2011-06-01	2021-10-12 2:28	2021-12-07 19:2		23-Jan		2	2										en
AY33YQL3	journalArticle	2011	Stillman, B., J. H.	Grand Opportunities: Strategies for Addressing Grar			1540-7063, 1557	10.1093/icb/icr05	https://academic.oup.com/icb/article-lookup/doi/10.1093/icb/icr052		2011-07-01	2021-10-12 2:28	2021-12-07 19:2		13-Jul		1	51										en
FLLVPDCV	journalArticle	2011	Wang, Fei-Yue	From AI to SciTS: Team Science and Research Intel			1541-1672	10.1109/MIS.201	http://ieeexplore.ieee.org/document/5898445/		2011-05	2021-10-12 2:28	2021-10-12 2:28		4-Feb		3	26										
9A9UWGX2	journalArticle	2011	Goff, Dan, Steph	The iPlant Collaborative: Cyberinfrastructure for Plar			1664-462X	10.3389/fpls.201	http://journal.frontiersin.org/article/10.3389/fpls.2011.00034/abstract		2011	2021-10-12 2:28	2021-10-12 2:28					2										

KXGRHX24	journalArticle	2011	Galbraith, Pamel	The Genomes of All Angiosperms: A Call for a Coord			2090-0120, 2090	10.1155/2011/64	https://www.hinda	Recent advances in biological instrumentation and associated experimental technologies now permit an unprecede	2011-11-17	2021-10-12 2:28	2021-12-07 19:2		10-Jan			2011										en
7T3GBLTS	journalArticle	2011	Stanzione, Dan	The iPlant Collaborative: Cyberinfrastructure to Fee			0018-9162	10.1109/MC.2011	http://ieeexplore.ieee.org/document/6025336/		2011-11	2021-10-12 2:28	2021-10-12 2:28		44-52		11	44										
72IWHKSU	journalArticle	2011	Kimbrel, Jeff H.,	RNA-Seq for Plant Pathogenic Bacteria			2073-4425	10.3390/genes20	http://www.mdpi.com/2073-4425/2/4/689		2011-10-13	2021-10-12 2:28	2021-12-07 19:2		689-705		4	2										en
WTJNLRH9	journalArticle	2011	Wang, Thomas F	A Low-Cost Library Construction Protocol and Data			1932-6203	10.1371/journal.p	https://dx.plos.org/10.1371/journal.pone.0026426		2011-10-19	2021-10-12 2:28	2021-12-07 19:2		e26426		10	6										en
BIJT4Y2M	journalArticle	2011	Barthelson, Sara	Plantagora: Modeling Whole Genome Sequencing a			1932-6203	10.1371/journal.p	http://dx.plos.org/10.1371/journal.pone.0028436		2011-12-12	2021-10-12 2:28	2021-12-07 19:2		e28436		12	6										en
ELY4QLG3	journalArticle	2011	Micklos, A., D.; L	Lessons from a Science Education Portal			0036-8075, 1095	10.1126/science.	http://www.sciencemag.org/cgi/doi/10.1126/science.1197074		2011-12-23	2021-10-12 2:28	2021-12-07 19:2		1657-1658		6063	334										en
CZ8DMWZG	conferencePaper	2011	McLay, Travis, R	A scalable parallel implementation of the neighbor joining algorithm for phylogenetic trees							2011	2021-10-12 2:28	2021-10-12 2:28															
L242ZCZJ	thesis	2011	Liu, Jun	W7 model of provenance and its use in the context of wikipedia					https://repository.	Data provenance refers to the lineage or pedigree of data, including information such as its origin and key events t	2011	2021-10-12 2:28	2021-10-12 2:28															
9X9AVS7W	journalArticle	2011	Schnable, Eric, J	Comparative gen	Maydica				https://journals-c	Of all the major plant groups, the grasses, with the complete genomes of five species, are the best positioned to ta	2011	2021-10-12 2:30	2021-12-07 0:37				2	56										
86UEQU8G	journalArticle	2011	Boerma, Ed, Rog	Soybean genomics research program strategic plan							2011	2021-10-12 2:51	2021-10-12 2:51		1–11		1	4										
SFQQZCE5	conferencePaper	2011	Del Vescovo, Chi	DeMoSt: a tool fo	Proceedings of the 10th International Semantic Web			10.1007/978-3-642- 25073-6_9			2011	2021-12-06 19:3	2021-12-07 0:33		191													
8BKPZCDH	bookSection	2011	Zheng, Chunfang	OMG! Orthologs	Algorithms in Bio	978-3-642-23037-0 978-3-642-23038-7			http://link.springer.com/10.1007/978-3-642-23038-7_30		2011	2021-12-06 19:3	2021-12-06 19:3	2021-12-06 19:3	364-375			6833								Springer Berlin H	Berlin, Heidelberg	
NS3D42AD	journalArticle	2011	Carey, Hannah V	Grand challenges	The Physiologist		0031-9376				2011-10	2021-12-06 19:4	2021-12-06 19:4		169, 171-172		5	54		Physiologist								eng
KNFQSY4S	journalArticle	2011	Zamer, William E	A Cohesive Biolo	BioScience		00063568, 1525	10.1525/bio.2011	https://academic.oup.com/bioscience/article-lookup/doi/10.1525/bio.2011.61.11.3		2011-11	2021-12-06 19:5	2021-12-07 19:2	2021-12-06 19:5	848-849		11	61		BioScience								en
PFWUJYYR	conferencePaper	2011	Basney, Jim; Doc	Distributed web s	Proceedings of the 2011 ACM workshop on Gateway computing environments						2011	2021-12-07 0:32	2021-12-07 0:32		13–20													
JT824PCS	conferencePaper	2011	Brown, Susan A;	Factors Influencin	PACIS						2011	2021-12-07 0:32	2021-12-07 0:32		31											Citeseer		
8SCS9SMG	conferencePaper	2011	Koesterke, Lars;	An efficient and s	2011 IEEE International Symposium on Parallel and Distributed Processing Workshops and Phd Forum						2011	2021-12-07 0:34	2021-12-07 0:34		523–530											IEEE		
TZPZGIM9	conferencePaper	2011	Lenards, Andrew	Building an envir	Proceedings of the 2011 ACM workshop on Gateway computing environments						2011	2021-12-07 0:34	2021-12-07 0:34		51–58													
CAWUTNAC	journalArticle	2011	Makumburage, G	Phenotype Unifo	Frontiers in Plant Science		1664-462X	10.3389/fpls.201	http://journal.frontiersin.org/article/10.3389/fpls.2011.00012/abstract		2011	2021-12-07 0:35	2021-12-07 0:35	2021-12-07 0:35				2		Front. Plant Sci.								
YN9R5PEU	conferencePaper	2011	Matasci, Naim; B	Correcting and S	TDWG 2011 Annual Conference						2011	2021-12-07 0:35	2021-12-07 0:35															
8XYEWBYN	conferencePaper	2011	Skidmore, Edwin	iPlant atmospher	Proceedings of the 2011 ACM workshop on Gateway computing environments						2011	2021-12-07 0:38	2021-12-07 0:38		59–64													
UTWLYFWQ	journalArticle	2010	Nelson, Damian	Applications and methods utilizing the Simple Sema			1756-0381	10.1186/1756-03	http://biodatamining.biomedcentral.com/articles/10.1186/1756-0381-3-3		2010-12	2021-10-12 2:24	2021-10-12 2:24		3		1	3										en
A2YIJQ44	journalArticle	2010	Moore, William L	ASIS&T research data access and preservation sum			954403	10.1002/bult.201	http://doi.wiley.com/10.1002/bult.2010.1720360611		2010-08	2021-10-12 2:24	2021-10-12 2:24		42-45		6	36										en
CNGE7JPC	journalArticle	2010	Wang, Lincoln D,	Localizing triplet periodicity in DNA and cDNA seque			1471-2105	10.1186/1471-21	https://bmcbioinformatics.biomedcentral.com/articles/10.1186/1471-2105-11-550		2010-12	2021-10-12 2:24	2021-10-12 2:24		550		1	11										en
NIHCSAV9	conferencePaper	2010	Ram, Wei, Sudha	How social is social bookmarking?		978-1-4244-8999-2		10.1109/BASNA.	http://ieeexplore.ieee.org/document/5730302/		2010-12	2021-10-12 2:24	2021-10-12 2:24		8-Jan													
QLDLINSN	bookSection	2010	McKay, Jason E.	Using the Generic Synteny Browse		978-0-471-25095-1			http://doi.wiley.com/10.1002/0471250953.bi0912s31		2010-09	2021-10-12 2:26	2021-10-12 2:26		bi0912s31													en
V6CZ4HT5	conferencePaper	2010	Perianayagam, S	Rex: A toolset for	2010 IEEE Intern	978-1-4244-8306-8		10.1109/BIBM.20	http://ieeexplore.ieee.org/document/5706639/		2010-12	2021-12-03 17:1	2021-12-06 17:4	2021-12-03 17:1	613-617						Rex					IEEE	Hong Kong, China	
IIG7ZBYL	conferencePaper	2010	Hanlon, Matthew	My-Plant.org: A p	2010 Gateway C	978-1-4244-9751-5		10.1109/GCE.20	http://ieeexplore.ieee.org/document/5676118/		2010-11	2021-12-06 17:2	2021-12-06 17:4	2021-12-06 17:2	1-8						My-Plant.org					IEEE	New Orleans, LA, USA	
JT2929PQ	conferencePaper	2010	Brown, Susan A;	Managing knowle	2010 43rd Hawaii International Conference on System Sciences						2010	2021-12-06 20:3	2021-12-06 20:3		1–10											IEEE		
7Z2FJXFZ	journalArticle	2010	Macías-Duarte, A	Novel microsatell	Conservation Genetics Resources						2010	2021-12-06 20:4	2021-12-06 20:4		67–69		1	2										
LYRJHGJQ	journalArticle	2009	Gessler, Rex T, D	SSWAP: A Simple Semantic Web Architecture and F			1471-2105	10.1186/1471-21	https://bmcbioinformatics.biomedcentral.com/articles/10.1186/1471-2105-10-309		2009-12	2021-10-12 2:19	2021-10-12 2:19		309		1	10										en
CADT826E	journalArticle	2009	Merchant, Edwin	Leveraging Cloud Infrastructure for Life Sciences Research Laboratories: A Generalized View							2009	2021-10-12 2:19	2021-10-12 2:19		53													
4YZPIEQK	conferencePaper	2009	Soltis, P. S., D. E	Advances in the use of next-generation sequence data in plant systematics and evolutic					http://www.actahort.org/books/859/859_23.htm		2009-12-31	2021-10-12 2:19	2021-10-12 2:19		193-206			859										
MDS8XYN2	journalArticle	2009	Han, Yujun; Burn	TARGeT: a web-b	Nucleic Acids Research		1362-4962, 0305	10.1093/nar/gkp2	https://academic.oup.com/nar/article-lookup/doi/10.1093/nar/gkp295		2009-06	2021-12-06 20:3	2021-12-07 19:2	2021-12-06 20:3	e78-e78		11	37			TARGeT							en
FUW9KVFR	conferencePaper	2009	Liu, Jun; Ram, S	Who does what:	19th Workshop on Information Technologies and Systems						2009	2021-12-06 20:3	2021-12-06 20:3		175–180													
EZ7Y3NSP	journalArticle	2008	Holuigue, Rodrig	Crops in all shapes and sizes			1474-760X	10.1186/gb-2008	https://static.tdwg.org/conferences/2008/tdwg_2008_proceedings.pdf		2008-10-24	2021-10-12 1:57	2021-12-03 18:4															
63RLCBI5	journalArticle	2008	Stein, Lincoln D	Towards a cyberinfrastructure for the biological sciences: progress, visions and challenges							2008	2021-10-12 2:09	2021-10-12 2:09															
ZHVKVCCN	conferencePaper	2008	Piel, William	Obstacles and Avenues Facing Phyloinformatic Research					https://static.tdwg.org/conferences/2008/tdwg_2008_proceedings.pdf		2008-10-24	2021-10-12 2:09	2021-12-03 18:4													Biodiversity Information Standards (TDWG) and the Missouri Botanical Garden		
YFGHAFZR	bookSection	2008	Ram, Sudha; Zha	Linking Biologica	Advances in Con	978-3-540-87990-9 978-3-540-87991-6			http://link.springer.com/10.1007/978-3-540-87991-6_4		2008	2021-12-03 17:1	2021-12-03 17:1	2021-12-03 17:1	22-32			5232								Springer Berlin H	Berlin, Heidelberg	
