# Supplementary material for: CyVerse: Cyberinfrastructure for open science
Source: PLoS Comput Biol. 2024 Feb 7;20(2):e1011270. doi: 10.1371/journal.pcbi.1011270 (PMC10878509; doi:10.1371/journal.pcbi.1011270)
Supplement: Attachment — Submitted filename: PCOMPBIOL-D-23-00939_Reviewer_Responses.pdf [file pcbi.1011270.s011.pdf]

THE UNIVERSITY OF ARIZONA

Institute for Computation and Data-Enabled Insight (ICDI)
University of Arizona

Biological Sciences Research Laboratories
Tucson, Arizona 85721
(520) 626-9104
tswetnam@arizona.edu

Thank you, Reviewer 1 and the Editor for these considerate remarks and for your suggestions.

We provide our responses to the comments and edits suggested below in ***red***

The original copy (pre-print) PDF has been compared to the final copy (submission) PDF. Besides the editorial responses, we have updated the number of CyVerse registered users (110,000 is now 124,000), and the number of attendees in our workshops and webinars through 2023 to-date.

Another change: we added Dr. Dan Stanzione, Executive Director of the Texas Advanced Computing Center (TACC) and a former iPlant Collaborative Principal Investigator to the list of co-authors. Dr. Stanzione has recently reassumed responsibilities for supervision of CyVerse resources at TACC. Dr. Stanzione has agreed to be added as a co-author.

Reviewer #1: Comments and significant suggested edits

1) I commend the authors and their team for providing a valuable resource to the scientific community, and for their dedication to enabling its use through innovation and training.
Thank you.

2) The web re-design (not new I know) is an attractive improvement in form and functionality, especially the DE.
Thank you.

3) Supplemental - Are the metadata AVU triplets indexed for searching? If so, this would be good to include. If not, this is necessary to address specifically. Also, where do these metadata 'fit' in the description of metadata on page 20 of the supplemental material?

Yes, the AVUs are used in two ways, (1) the indexing internally for our ElasticSearch (OpenSearch) and (2) when the data are shared `anonymous` as read-only they are visible through the Data Commons (https://datacommons.cyverse.org). These AVUs are subsequently crawled by schema.org and appear in web searches on Google / Bing

We have added statements (emphasis on **bold**) to the metadata descriptions on pages 12 and 15 of the Supplemental Materials:

The iRODS Data Store uses a conventional three-tiered Linux permission system (i.e., 'read', 'write', and 'own') for files and folders. Data is shared internally with iRODS by adding permissions to individual objects or collections with other CyVerse users' private names, or with 'teams' made up of grouped usernames administered by a project owner. ***Data can also be shared with the entire CyVerse user base by adding the 'public' username group, or the open internet by adding the 'anonymous' username group.*** *The CyVerse metadata database is the primary repository for DE metadata storage. Based on AVU-triples (Attributes-Values-Units) foundation of iRODS metadata database, the CyVerse metadata database allows an unlimited number of AVU combinations. For example, users can use the same attribute with more than one value or more than one type of unit, significantly expanding the degree of metadata that can be stored for data analysis and retrieval.* ***AVUs are exposed to web crawlers, like schema.org when they are shared with the 'anonymous' username group. These metadata allow both files and folders to become searchable using common search engines, i.e., Google or Bing. Metadata templates for common metadata standards, e.g., the Dublin Core (https://www.dublincore.org/) and DataCite [140, 141] provide pre-configured formats for users to apply standard AVUs to their data files and folders in the Discovery Environment. The AVUs are visible publicly via the Data Commons website and programmatically through iRODS and its APIs***

4) Supplemental - Page 15 - Data Commons - What is the process for curating datasets? Is there expert curation? Who performs this and how are curations quality controlled? A brief explanation would be helpful.

THE UNIVERSITY OF ARIZONA

Institute for Computation and Data-Enabled Insight (ICDI)
University of Arizona

Biological Sciences Research Laboratories
Tucson, Arizona 85721
(520) 626-9104
tswetnam@arizona.edu

The Data Commons are curated by an internal expert review team who communicate with the authors of the data. Edits to metadata fields may be suggested and the data must meet all of the internal requirements for DataCite publication before they are submitted for DOI.

User owned Community Released data do not have expert curation, and are treated as living project data that are subject to change or removal by their owners.

We have added a brief explanation statement to the DataCommons section which describes the process in more detail. The exact steps are defined in our public documentation: https://learning.cyverse.org/ds/doi/

New text on pages 15-16:

Data Commons hosts both curated and community released data. The Data Commons can be used to publish (DataCite DOI) data for which there are no other existing canonical repositories or for which hosting on other research data services would not be feasible, such as very large datasets or data that need to be linked to analysis tools on CyVerse. Where appropriate, CyVerse encourages publication to canonical data repositories such as the National Center for Biotechnology Information (NCBI) [135 – 137]. CyVerse also provides tools for publishing sequence data directly to NCBI's Sequence Read Archive (SRA) [138, 139]. Curated data in the Data Commons are published via DataCite [140, 141] and receive a DOI. In addition to publication of static datasets with a DOI, shared data can exist as writable (editable) archives that can be modified by their owners in the Data Store's 'Community Released' projects. Community released data folders can be shared publicly as 'read-only' files over WebDav (https://data.cyverse.org) and the Data Commons (https://datacommons.cyverse.org) when shared with the 'anonymous' username group. Users can request DataCite DOI publication through the User Portal or by 'Publishing' their data in the Discovery Environment. Metadata are applied to the community released and curated folders using the Discovery Environment's metadata templates and AVUs. ***The steps for requesting both a Community Released folder and a DataCite DOI folder are described in the user documentation (https://learning.cyverse.org/ds/doi/). Every DOI requestor must complete the DataCite metadata template with required fields in the Discovery Environment. The template is submitted for review, where CyVerse DOI experts communicates with the authors, ensuring that all fields are complete and meet the DataCite standards. Once the DOI has been granted all write and own permissions are and own permissions are removed from the folder and it is transferred to the '/curated' folder space as 'read-only'.***

5) Is there a process to quality control public Apps supplied by users?

Yes, there is a process whereby a CyVerse employee inspects the Dockerfile and the container image before and after copying the image to the harbor.cyverse.org container registry (explained on pages 16 and 17 of the Supplemental Materials.

We have added additional text to the Discovery Environment section as a sub-section on "Publishing custom Tools and Apps". The longer details are available in our user documentation https://learning.cyverse.org/de/create_apps/

New text on page 20 of the Supplemental Materials:

Publishing custom Tools and Apps
The only prerequisite to user's publishing their own private container apps is that the container image for the app must reside in a Docker configured to be trusted by the Discovery Environment, i.e., Harbor (https://harbor.cyverse.org). If the app publishing request container image is not in a trusted registry then the request is sent to Discovery Environment administrators as a notification. This process allows administrators to review the container image before allowing the app to be published. If the container used by the app already resides in one of the registries that are trusted by the Discovery Environment, then the app is made available immediately. Forcing apps to be in a trusted registry provides a few important benefits. First, it allows administrators to inspect any container images that aren't already in one of the trusted registries for vulnerabilities. Second, it allows administrators to ensure that different image tags are used for different versions of the same container image, which helps to ensure reproducibility. Third, it can help to avoid errors caused by rate limiting from third party registries. Whether or not administrator intervention is required to publish the app, all newly published apps are tagged as Beta apps. Only administrators can remove this tag, so users of the app can request an inspection before using the app if they want.

THE UNIVERSITY OF ARIZONA

Institute for Computation and Data-Enabled Insight (ICDI)
University of Arizona

Biological Sciences Research Laboratories
Tucson, Arizona 85721
(520) 626-9104
tswetnam@arizona.edu

6) Fig S10 - What is 'porklock'? Is a citation needed? As written this is mysteriously uninformative.

Porklock is the name of the file utility that we use to transfer files to and from the data store for non-interactive analyses in the Discovery Environment. This utility was developed here at CyVerse, so no citation is needed. You can find the source code in the https://github.com/cyverse-de/porklock We apologize for the confusion; that name was intended to be internal. We have added the link to the porklock repository in the figure caption.

7) Line 80 - Deployment in a matter of hours is only possible with the necessary experience and expertise. Could this statement be reworded to clarify that fast deployment does require necessary knowledge and skills and is not an 'out of the box' possibility for the majority of users? For instance, not all users can access all layers of the 'cake' stack as specified in the following section.

Yes, we agree that this needs re-wording, and was an oversight of ours. We have re-worded the statements around line 76-93 to read:

CyVerse utilizes HPC resources at Texas Advanced Computing Center (TACC) and HTC through the OpenScienceGrid, each of which are connected over Internet2 [45] (S1 Fig). CyVerse is also connected to and manages commercial resources on AWS, Google Cloud, and Azure through its external partnership programs. ***By managing itself as SaaS and IaC, replicated versions of CyVerse can be deployed by experienced CyVerse staff in a matter of hours.*** *Managing both SaaS and IaC has benefits from an economy of scale perspective and reduces more costly and time-consuming manual deployment processes on physical hardware.* ***New CyVerse deployments can be undertaken by anyone using the public developer documentation at https://docs.cyverse.org.***

8) Line 147 - Are there any data (counts) available to include for the number of workshop attendees who used CyVerse as part of successful proposals and the other examples listed?

Unfortunately, no. We have not been able to ascertain totals on how many workshop participants have used CyVerse in their proposals. We do have the list of letters of support we have written.

9) Scientific Impacts - an impressive section!

Thank you.

Minor suggested edits

1) Supplemental - Page 9 - User Portal - It may be good to specify that users must request and be approved to be powered by CyVerse. As written, it appears as though the choice is entirely in the hands of the users.

We have updated the text:

User Portal
Through the User Portal (Fig 4), new users can create and manage their account, request access to featured platforms, and request to schedule workshops. The User Portal interface also provides hyperlinks to all the CyVerse platform's featured Services.
Requests for access to featured services, platforms, workshops, and community data released folders are reviewed by CyVerse staff. Requests for federated CyVerse services, 'Powered by CyVerse' as a 3rd party platform projects, or replicated CyVerse deployments, are sent through the ticketing system (Intercom.io). Requestors are contacted directly by CyVerse leadership to begin discussions and contracting agreements.

2) Line 38 - 'compliments' can work, but 'components' may be easier for a broad audience to understand

We have changed the word 'compliments' to 'components'

THE UNIVERSITY OF ARIZONA

Institute for Computation and Data-Enabled Insight (ICDI)
University of Arizona

Biological Sciences Research Laboratories
Tucson, Arizona 85721
(520) 626-9104
tswetnam@arizona.edu

(3) Line 51 - fame is less of a factor than capability - suggest changing to say something like, '...best equipped and well funded...'

Yes, thank you. We have changed the text to say 'best equipped and well-funded'

4) Line 96 - Should 'CyVerse' be possessive here? 'CyVerse's featured software stack...'?

Yes, we have made it possessive (CyVerse')

5) Line 155 - perhaps just say 'outside' or 'beyond' life sciences?

Edited to say: "...over 23% of users beyond life science"

6) Be consistent with use of 'R1' and 'Research 1'

Removed 'R1' and kept 'Research 1' throughout.

7) Supplemental - Page 20 - How challenges are met - Should this section be moved to the main manuscript? Especially the first paragraph. The last 2 paragraphs appear a little awkwardly fitted in.

Originally, we had that section and the 'Migration to Cloud..." sections in the main text but moved it to the Supplementary Materials in-order to stay within the PLOS Comp Bio word-limit for a Software manuscript.

We have removed the last two paragraphs of the 'How Challenges" section as they are digressive to the rest of the material. We do intend to submit a commentary / opinion in the future about how CyVerse has met and failed to meet these grand challenges where those last two paragraphs will have more space to elaborate upon their meanings.
